# Straightforward Synthesis of α-Chloromethylketimines Catalyzed by Gold(I). A Clean Way to Building Blocks

**DOI:** 10.1021/acs.joc.1c02877

**Published:** 2022-02-22

**Authors:** Jeymy T. Sarmiento, María Cárcel, Carmen Ramírez de Arellano, Teresa Varea, Gregorio Asensio, Andrea Olmos

**Affiliations:** Departamento de Química Orgánica, Universidad de Valencia, Av. Vicente Andrés Estellés S/N, 46100 Burjassot, Spain

## Abstract

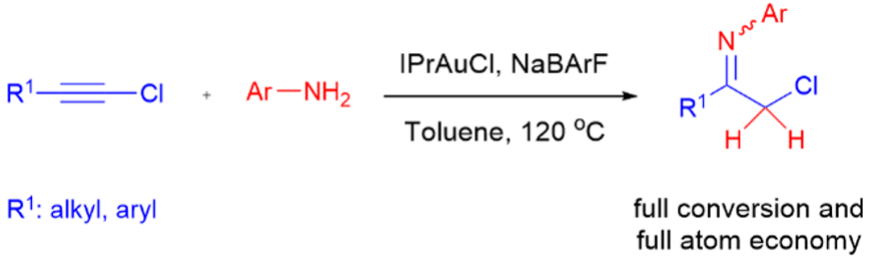

α-Chloromethylketimines have been obtained through a gold-catalyzed hydroamination of aromatic and aliphatic 1-chloroalkynes with aromatic amines by using equimolar amounts of both reagents. This procedure has allowed the preparation and spectroscopic characterization of α-chloromethylketimines for the first time with a high degree of purity, complete conversion, and atom economy. The synthetic usefulness of the methodology has been demonstrated with the preparation of β-chloroamines and indoles.

## Introduction

α-Monohalogenated imines are dielectrophilic compounds in which there has long been interest.^1^ The reduction of these compounds provides β-haloamino derivatives,^2^ the main entrance route for the synthesis of aziridines,^3^ substances with interesting biological properties that are widely used as synthetic intermediates.^4^ In addition, halogenated imines can be subjected to in situ cyclization to afford indoles,^5^ piperidines, and other nitrogen-containing heterocycles.^6^

**Scheme 1:**
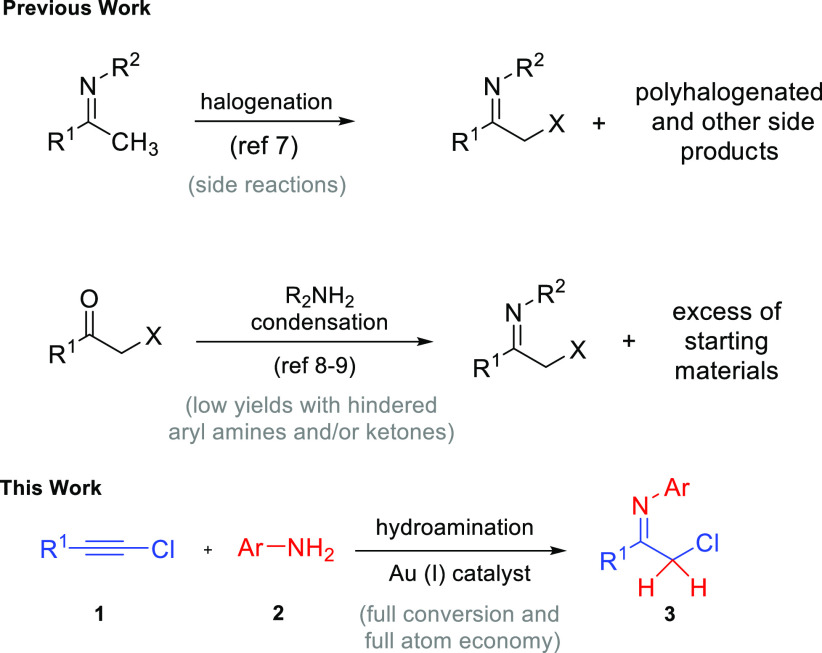
Syntheses of Halomethylketimines

Two strategies have been applied for the synthesis of halogenated ketimines: the electrophilic halogenation^7^ of the corresponding imine in a process similar to the halogenation of ketones and the condensation of a monohalogenated ketone with an amine (Scheme 1). In the first case, the low regioselectivity and the formation of per-halogenated or unstable compounds make this procedure unsuitable for the monochlorination of simple ketimines. The second route, usually catalyzed by molecular sieves^8^ or TiCl4,^9^ is less effective in the case of hindered amines or when ketones are used instead of aldehydes. In addition, the condensation reaction requires in most cases the use of a large excess of one of the reactants. Due to their facile hydrolysis, purification is a major problem and prevents the isolation of α-halogenated ketimines. For this reason, they have never been properly characterized by NMR. Thus, these species are used in situ in the presence of large amounts of starting materials and side products that complicate subsequent transformations.^5,6,8,9^

The synthetic routes for α-chloroketimines described so far cannot be considered general or satisfactory in any instance. Therefore, the design of a general and clean way to prepare these versatile building blocks is necessary. With this aim, we have developed a new strategy by using 1-chloroalkynes as precursors and Au(I) catalysis (Scheme 1). 1-Chloroalkynes are readily available compounds whose usefulness in synthesis has been widely demonstrated.^10^ In this context, Xiang, He, and co-workers^11^ reported the synthesis of α-halomethylketones in the gold-catalyzed hydration of 1-haloalkynes. On the contrary, since the pioneering work of Tanaka,^12^ the intermolecular hydroamination of alkynes has been highlighted as one of the best alternatives for the synthesis of conventional imines and enamines.^13^ Interesting advantages of organic gold derivatives are the compatibility with the presence of different functional groups, the very low air sensitivity, and the stability to β-elimination of these compounds.^14^ Different Au complexes and nanoparticles have been used in the reaction of alkynes with amines^15^ and other N-nucleophiles^16^ with excellent results, but to the best of our knowledge, none of the methodologies described has been used to address the preparation of halogenated imines in spite of their synthetic value.

In this paper, we describe the regioselective Markovnikov hydroamination of 1-chloroalkynes to afford α-chloroimines in high yields under mild reaction conditions.

## Results and Discussion

The conditions were first optimized for the gold-catalyzed reaction between equimolecular amounts of 1-chloroethynylbenzene (**1a**) and aniline (**2a**) (see Table 1). The consumption of **1a** was followed by GC (gas chromatography). Chloroimines suffer several transformations under these high-temperature conditions. Therefore, the analysis and yield of **3aa** were determined by NMR. The use of 1 mol % 1,3-bis(2,6-diisopropylphenyl-imidazol-2-ylidene)gold(I) chloride (IPrAuCl) activated by 1.5 mol % sodium tetrakis[3,5-bis(trifluoromethyl)phenyl]-borate (NaBArF) as the catalyst in toluene (0.42 M) at 120 °C for 0.5 h gave α-chloroimine **3aa** in excellent yield (Table 1, entry 4). We selected these conditions to explore the reaction scope.

**Table 1:** Optimization of the Reaction Conditions of the Hydroamination of **1a** with **2a**^a^

entry	catalyst (mol %)	solvent	temp (°C)	time (h)	yield of **3aa** (%)^b^
1	IPrAuCl (3)/AgSbF_6_ (4)	CH_2_Cl_2_	rt	22	90
2	IPrAuCl (1)/NaBArF (1.5)	CHCl_3_	75	3	92
3	IPrAuCl (1)/AgSbF_6_ (1.5)	CHCl_3_	75	3	65
4	IPrAuCl (1)/NaBArF (1.5)	toluene	120	0.5	93
5	IPrAuCl (0.3)/NaBArF (0.45)	toluene	120	15	89
6	IPrAuCl (2)/AgNTf_2_ (3)	toluene	120	1.5	15
7	PPh_3_AuCl (2)/AgSbF_6_ (3)	toluene	120	1.5	40

a Reaction conditions: **1a** (0.5 mmol), **2a** (0.5 mmol), solvent (1.2 mL).

b Yield determined by ^1^H NMR using 1,1,2,2-tetrachloroethane as the internal standard.

These selected conditions were used for the reaction of alkyne **1a** with a series of primary amines **2a–h** to yield the corresponding chlorinated imines **3aa–ah**, respectively. Reaction mixtures were heated until complete conversion of chloroalkyne **1a** had been achieved followed by GC analysis. Yields determined by NMR and reaction times are shown in Table 2.

**Table 2:**
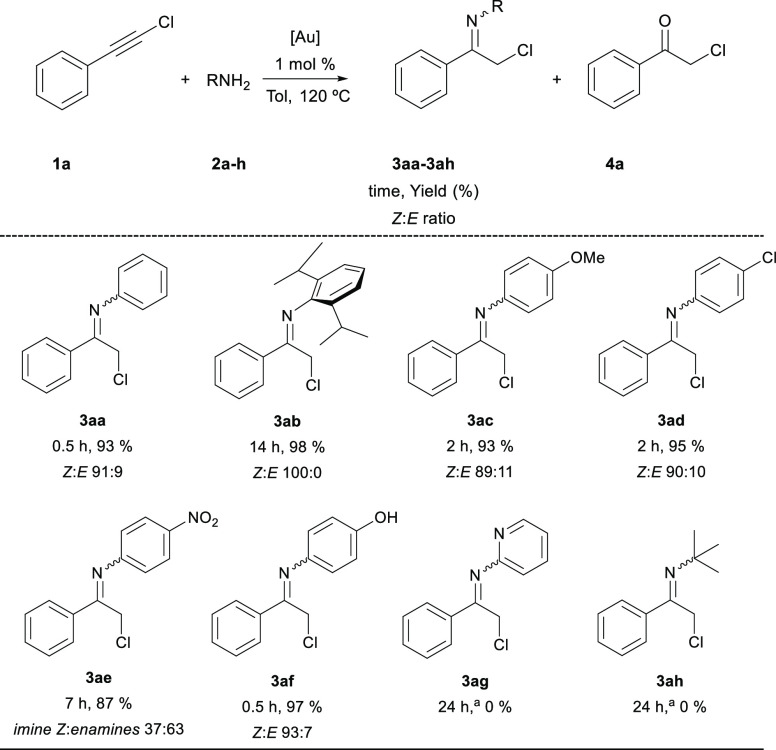
Scope of the Reaction of **1a** with Amines **2a–h**^a^

a Reaction conditions: **1a** (0.5 mmol), **2a–h** (0.5 mmol), IPrAuCl (5 μmol), NaBArF (7.5 μmol), toluene (1.2 mL). Yields determined by ^1^H NMR using 1,1,2,2-tetrachloroethane as the internal standard. In the case of amines **2g** and **2h**, chloroalkyne **1a** was recovered unreacted.

Reactions proceeded smoothly to the formation of both *Z* and *E* imines together with trace amounts of the corresponding enamines. Manipulation of reaction crudes was limited to toluene evaporation and addition of the deuterated solvent and standard. Any other purification procedure led to extensive hydrolysis of reaction products. Moreover, even substitution of a solvent for deuterated chloroform under inert conditions provoked the partial (2–7%) hydrolysis of cloromimines **3** into α-chloromethylketone **4a**. Yields were determined by ^1^H NMR using 1,1,2,2-tetrachloroethane as the internal standard. The NMR spectra corresponding to the crude reaction products can be found in the Supporting Information.

The noteworthy efficiency of this procedure is demonstrated by the impressive result obtained with the strongly hindered 2,6-diisopropylaniline (**2b**). It must be pointed out that imine **3ab** can be hardly prepared through condensation reactions. The hydroamination reaction also tolerates the presence of both electron-donating (**3ac**) and electron-withdrawing (**3ad**) groups on the aniline aromatic ring. In the case of the strong withdrawing substituent nitro group (**3ae**), the product was formed in high yield, although the corresponding chloroenamines were obtained preferentially over the imine tautomers. Additionally, the yield is not affected by the presence of free hydroxy groups on the substrates as observed in **3af**. Unfortunately, the presence of more basic nitrogen atoms in the amine counterpart like those of pyridine **2g** or aliphatic amine **2h** inhibits the transformation, and chloroalkyne **1a** was recovered unreacted probably due to the strong coordination of the amine with the gold catalyst.

During a second step, we explored the reactivity of different 1-chloroalkynes (**1b–f**) with aniline (**2a**), and the results are summarized in Table 3. Excellent to very good yields were obtained in all cases, except for chloroalkyne **2f**, for which the presence of a pyridine again inhibited the reaction. The addition of 1 equiv of TfOH to avoid catalyst poisoning^17^ resulted in the formation of a complex reaction mixture. Chloroalkyne **2b** with the bulky mesityl substituent needed 15 h to complete the reaction, and the elusive imine **3ba** could be obtained in 97% yield.

**Table 3:**
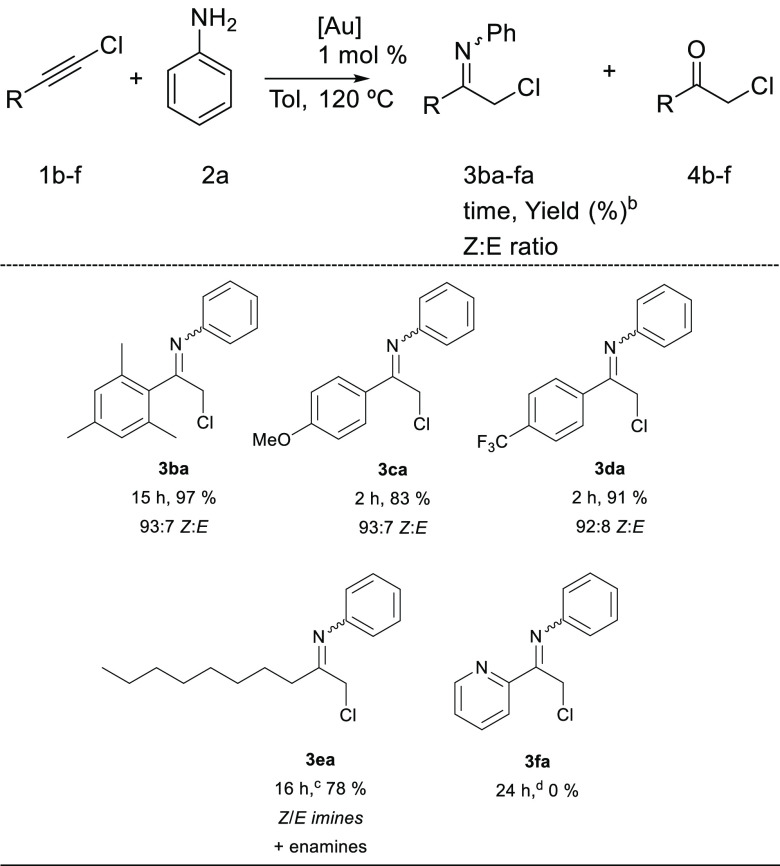
Scope of the Reaction of **2a** with Chloroalkynes **1b–f**^a^

a Reaction conditions: **1b–f** (0.5 mmol), **2a** (0.5 mmol), IPrAuCl (5 μmol), NaBArF (7.5 μmol), toluene (1.2 mL).

b Yields determined by ^1^H NMR using 1,1,2,2-tetrachloroethane as the internal standard.

c To achieve complete conversion of **1e**, 2 mol % IPrAuCl and 3 mol % NaBArF had to be used.

d In the case of alkyne **1f**, starting materials were recovered unreacted.

**Scheme 2:**
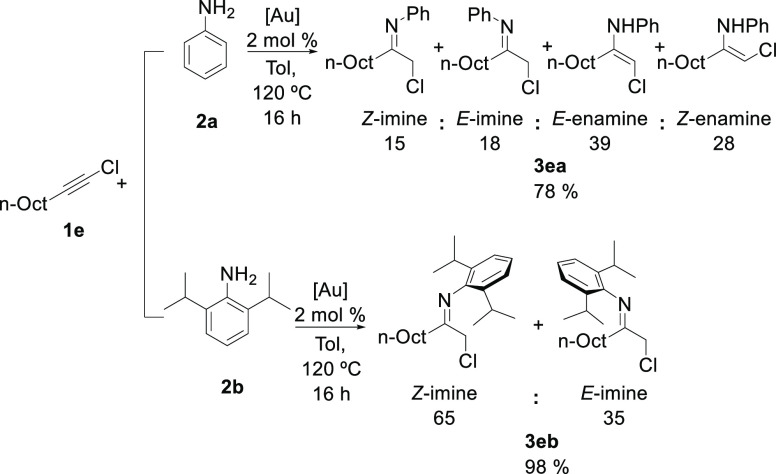
Synthesis of α-Chloroimine Tautomers **3ea** and **3eb**

Aliphatic chloroalkyne **1e** gave the expected product as a mixture of different imines and the corresponding enamines (see the Supporting Information). In this case, 22% hydrolysis of the product could not be avoided due to the higher sensitivity of **3ea** to traces of water. When hindered amine **2b** was used for the hydroamination reaction of aliphatic alkyne **1e**, the expected chloroimine **3eb** was obtained in 98% yield as a 65:35 *Z*/*E* mixture of diastereomers (Scheme 2). In this case, enamines were observed only in traces.

**Figure 1:**
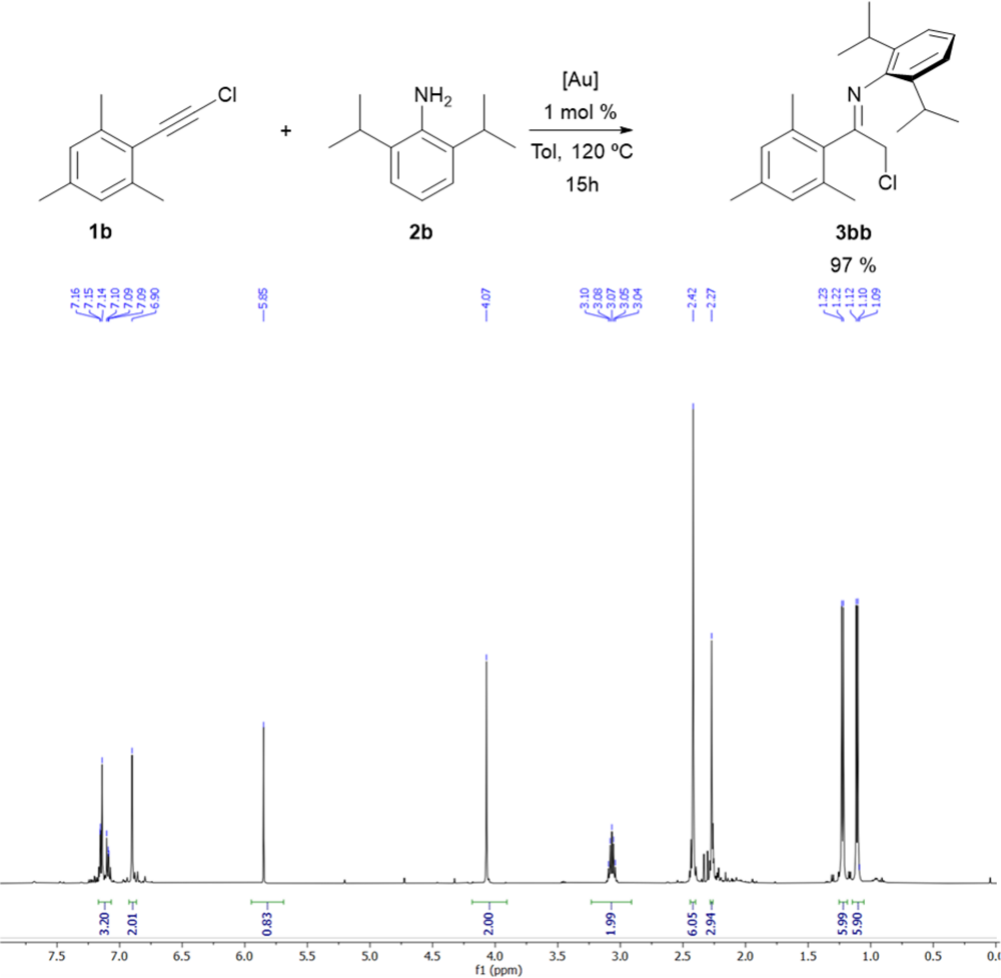
Synthesis and ^1^H NMR spectrum of chloroimine **3bb**.

Encouraged by the remarkable results obtained for encumbered chloroimines **3ab**, **3ba**, and **3eb**, we attempted to synthesize the challenging chloroimine **3bb** under our standard conditions. To our delight, the expected chloroimine **3bb** was formed in 98% yield after 15 h at 120 °C with just 1 mol % activated IPrAuCl catalyst, as one can see on the ^1^H NMR spectrum of the crude reaction mixture (Figure 1).

Additionally, we checked the viability of the gold-catalyzed hydroamination reaction for all possible combinations of electron-withdrawing and electron-donating substituents at the *para* position of the chloroalkyne and amine counterparts. As one can interpret from the results shown in Table 4, catalyst effectiveness is maintained in any case. The presence of electron-donating groups on the aniline moiety (**2c**) increases the nucleophilicity of the nitrogen atom but also the coordinating ability to the catalyst, leading to results similar to those of aniline **2a**. In fact, a longer reaction time was required for the preparation of chloroimine **3cc** that joins electron-donating groups in both starting materials. In addition, it was observed that products derived from chloroalkyne **1c** are more prone to hydrolysis.

**Table 4:** Influence of the Electronic Character of the *para* Substitution on the Reaction Efficiency^a^

chloroalkyne **1c** or **1d** (R^1^)	amine **2c** or **2d** (R^2^)	imine **3**	time (h)	yield (%)^b^	*Z*:*E* ratio
**1c** (OMe)	**2c** (OMe)	**3cc**	4	85	93:7
**1c** (OMe)	**2d** (Cl)	**3cd**	2	86	93:7
**1d** (CF_3_)	**2c** (OMe)	**3dc**	2	93	91:9
**1d** (CF_3_)	**2d** (Cl)	**3dd**	2	93	93:7

a Reaction conditions: **1c** or **1d** (0.5 mmol), **2c** or **2d** (0.5 mmol), IPrAuCl (5 μmol), NaBArF (7.5 μmol), toluene (1.2 mL), 120 °C.

b Yields determined by ^1^H NMR using 1,1,2,2-tetrachloroethane as the internal standard.

**Figure 2:**
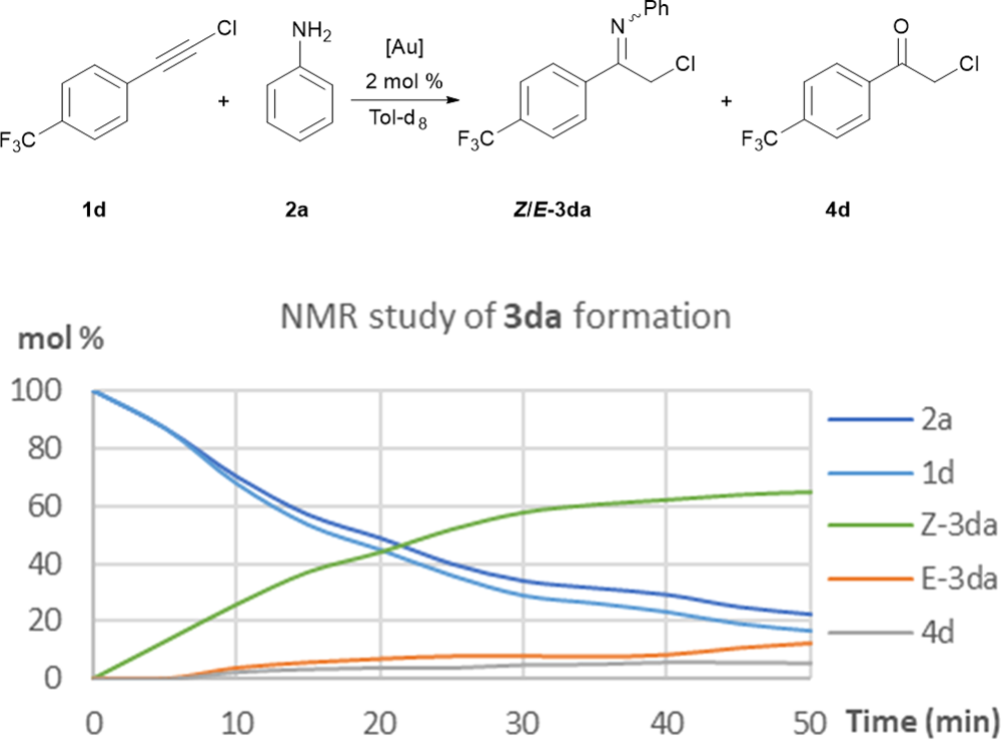
NMR monitoring of gold-catalyzed **3da** formation.

We studied the formation of chloroimine **3da** through NMR spectroscopy to assess if the *Z*/*E* stereoselectivity observed for the gold-catalyzed hydroamination reactions is a consequence of the catalyst activity or is due to tautomeric equilibrium. For safety reasons, the temperature of the reaction was decreased to 80 °C. Consequently, the catalyst amount was doubled to maintain the reaction rate. Figure 2 shows the evolution of the concentration of all reagents and products during the reaction time. Initially, only chloroimine **3da** with a *Z* configuration can be observed. Only after an important accumulation of this species is the *E* diastereomer formed. This result points to a highly stereoselective formation of the *Z* isomer in the gold catalysis that it is tautomerized by the corresponding *E/Z* equilibrium. The equilibrium on different solvents and temperatures was not further investigated.

Chloroenamines are highly reactive interesting synthetic intermediates that have been prepared through TiCl_4_-catalyzed condensation of the corresponding α-chloroketone with an excess of secondary amine.^18^ Due to the limitations associated with this procedure, only chloroenamines derived from aliphatic amines have been synthesized. We tested the performance of our catalytic system on the hydroamination reaction of chloroalkyne **1a** with the secondary aromatic amines **5a–e** under our standard conditions (Table 5). The expected chloroenamine **6aa** from *N*-methylaniline was formed in excellent 90% yield exclusively in the *E* configuration (see the Supporting Information). The reaction tolerates the presence of electron-donating (**6ab**) and electron-withdrawing (**6ac**) groups on the aromatic ring of the amine and on the alkyne (**6de**), although for electron rich secondary amine **5b** some chloroalkyne remained unreacted under the standard reaction conditions. The methyl group on the nitrogen atom can be replaced by a longer alkyl chain (**6ad**) or a second aromatic ring (**6de**) without an important loss of yield. These results open a way to the synthesis of a new family of aromatic chloroenamines that have not been accessible to date.

**Table 5:**
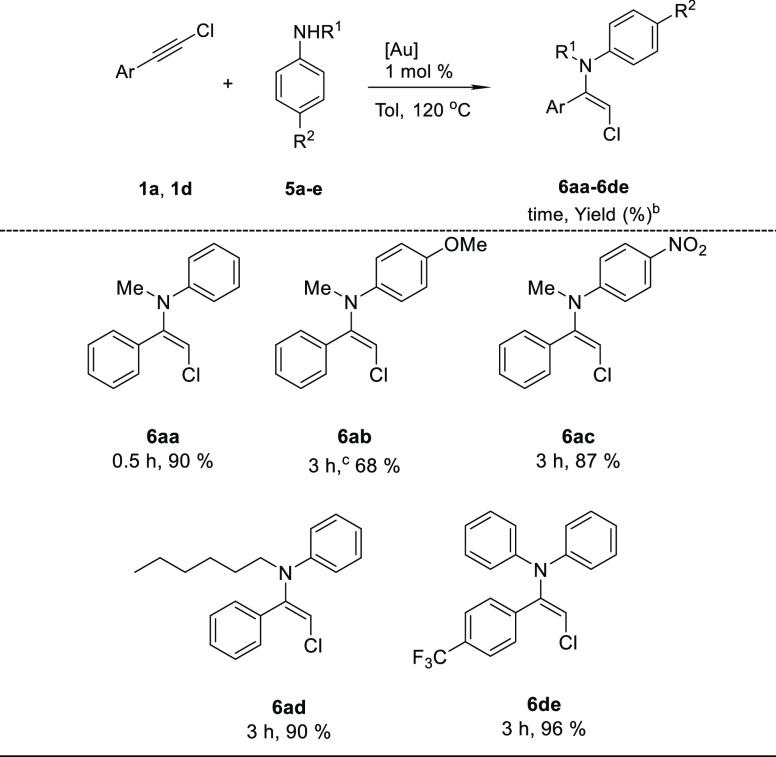
Gold-Catalyzed Chloroenamine Synthesis^a^

a Reaction conditions: **1a** or **1e** (0.5 mmol), **5a–e** (0.5 mmol), IPrAuCl (5 μmol), NaBArF (7.5 μmol), toluene (1.2 mL).

b Yields determined by ^1^H NMR using 1,1,2,2-tetrachloroethane as the internal standard.

c 19% of unreacted chloroalkyne **1a** was recovered.

The synthetic usefulness of this procedure was evaluated through the development of two derivatization procedures based on one-pot, two-step syntheses. First, crude chloroimines **3aa**, **3ba**, and **3ea** were directly subjected to palladium-catalyzed cyclization to afford indoles **7** (Table 6). The results obtained with this selection of chloroimines show the power of this methodology for preparing indoles presenting bulky or aliphatic substituents, arduous to synthesize through other pathways such as condensation^5^ or cross-coupling.^19^

**Table 6:**
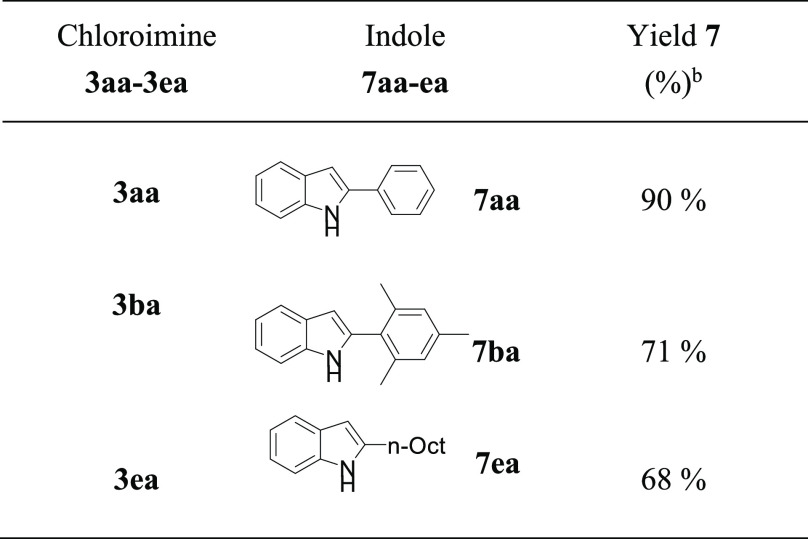
Pd-Catalyzed Cyclization of α-Chloroimines to Indoles^a^

a Reaction conditions: (1) **1a–e** (1.0 mmol), **2a** (1.0 mmol), IPrAuCl (10 μmol), NaBArF (15 μmol), toluene (2.4 mL), 120 °C; (2) Pd(OAc)_2_ (0.05 mmol), P(*o*-tolyl)_3_ (0.3 mmol), CsF (3 mmol), THF 10 mL, 65 °C.

b Isolated yields.

Additionally, a procedure for the direct reduction to β-chloroamines has been assayed. These interesting compounds are important intermediates for the preparation of aziridines.^3^ Their syntheses are especially complicated in the case of substrates with bulky substituents. Once complete conversion of the corresponding chloroalkyne into the imine was achieved, toluene was replaced by methanol and NaBH_3_CN added as the reductant. This procedure was applied to a selection of examples. The yields obtained of the crude chloroamines (**8**) are listed in Table 7.

**Table 7:**
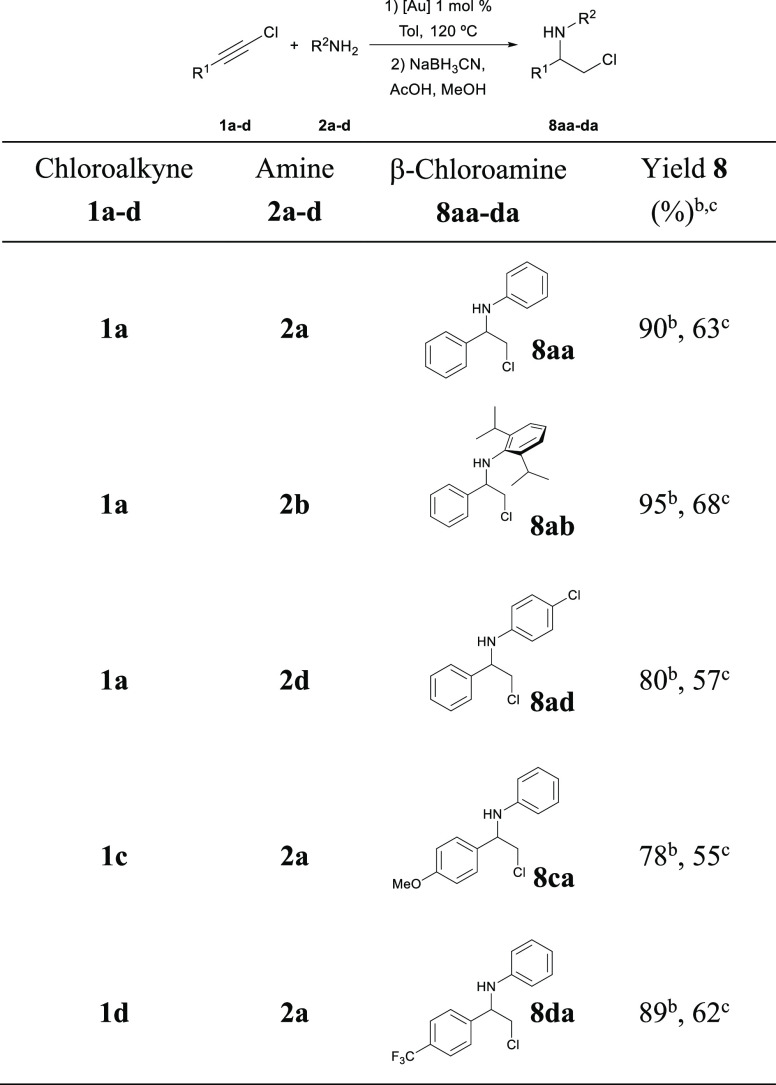
Obtaining β-Chloroamines through a One-Pot, Two-Step Procedure^a^

a Reaction conditions: (1) **1a–d** (1.0 mmol), **2a–d** (1.0 mmol), IPrAuCl (10 μmol), NaBArF (15 μmol), toluene (2.4 mL), 120 °C; (2) AcOH (1.0 mmol), NaBH_3_CN (1.1 mmol), MeOH (6 mL), rt.

b Determined by NMR analysis of reaction crudes.

c Isolated yield.

In conclusion, we have developed a new catalytic procedure for the synthesis of α-chloromethylketimines with full atom economy. The hydroamination reaction takes place with equimolar amounts of chloroalkynes and aromatic amines. The appropriate gold catalyst at just 1 mol % is enough to reach complete conversions and allows the preparation of these interesting synthetic intermediates in a degree of purity not yet achieved. The scope of this method has been extended to the use of secondary amines for the preparation of the elusive chloroenamines. To assess the usefulness of this synthetic procedure, two significant kinds of building blocks, β-chloroamines and indoles, have been cleanly prepared through direct methods.

## Experimental Section

### General Experimental Details

Chloromethylimines are products that are very sensitive to hydrolysis. All transformations have been performed using common Schlenk techniques or in a glovebox. Toluene, toluene-*d*_8_, and THF have been dried with sodium prior to use. Deuterated chloroform and liquid amines have been distilled from CaH_2_ prior to use. NMR analyses have been performed in a Bruker AvanceIII 300 spectrometer, a Bruker AV400 spectrometer, or a Bruker Neo500 spectrometer. NMR data have been processed using MestReNova or TopSpin. Residual signals of deuterated solvents have been used as internal references (CDCl_3_ at 7.26 ppm in ^1^H NMR and 77.16 ppm in ^13^C NMR and toluene-*d*_8_ at 2.08 and 20.43 ppm for methyl group in ^1^H NMR and ^13^C NMR, respectively). IR spectra have been recorded on a Thermo Scientific Nicolet iS10 instrument and processed with Omnic. IR band frequencies have been rounded to 1 cm^–1^. GC-MS analyses have been performed with an Agilent 5977A instrument equipped with a 30 m × 0.25 m × 0.25 μm HP-5ms ui column. HRMS (+ESI) analyses have been performed in an AB SCIEX TripleTOF 5600 LC/MS/MS system, and data have been processed using PeakView. Elemental analyses have been performed in a Thermofisher Flashmart Eager 200 instrument.

### General Chloroalkyne Synthesis

A 2.5 M solution of *n*-butyllithium in hexanes (2.6 mL, 6.5 mmol, 1.3 equiv) was slowly added to the corresponding alkyne (5 mmol, 1 equiv) dissolved in 12 mL of THF at −78 °C. After the mixture had been stirred for 1 h at that temperature, a suspension of *N*-chlorosuccinimide (868 mg, 6.5 mmol, 1.3 equiv) in 5 mL of THF was added via cannula. The cooling bath was removed, and the mixture was stirred for an additional 5 h at room temperature. The reaction mixture was hydrolyzed with 20 mL of water, and the aqueous phase extracted with Et_2_O (2 × 20 mL). The organic phases were mixed, dried with anhydrous magnesium sulfate, and concentrated. The crude product was purified through column chromatography using *n*-hexane as the eluent. Chloroalkynes **1a**, **1c**, **1d**, and **1f** were obtained in 60–80% yields, and their NMR data were consistent with literature values.^10b,17^

#### 2-(Chloroethynyl)-1,3,5-trimethylbenzene (**1b**)

Eluent *n*-hexane; 732 mg as a white solid; 82% yield; ^1^H NMR (500 MHz, CDCl_3_) δ 6.85 (s, 2H), 2.39 (s, 6H), 2.28 (s, 3H); ^13^C{^1^H} NMR (125 MHz, CDCl_3_) δ 141.0, 138.1, 127.7, 119.1, 74.3, 67.6, 21.4, 21.0; IR (cm^–1^) 2942, 2914, 2212, 1478. Elemental analysis calcd for C_11_H_11_Cl: C, 73.95; H, 6.21. Found: C, 73.05; H, 6.05.

#### 1-Chloro-1-decyne (**1e**)

Eluent *n*-hexane; 536 mg as a colorless oil; 62% yield; ^1^H NMR (300 MHz, CDCl_3_) δ 2.16 (t, *J* = 7.0 Hz, 2H), 1.55–1.45 (m, 2H), 1.39–1.26 (m, 10H), 0.91–0.86 (m, 3H); ^13^C{^1^H} NMR (75 MHz, CDCl_3_) δ 70.0, 57.1, 32.0, 29.3, 29.2, 29.0, 28.5, 22.8, 18.9, 14.2; IR (cm^–1^) 2928, 2856, 2243, 2214, 1464, 851. Elemental analysis calcd for C_10_H_17_Cl: C, 69.55; H, 9.92. Found: C, 68.95; H, 9.37.

### Gold-Catalyzed Hydroamination Reactions

IPrAuCl (3 mg, 5 μmol, 1 mol %) and NaBArF (6.6 mg, 7.5 μmol, 1.5 mol %) were weighed in a glovebox and introduced into an ampule provided with a J. Young valve. Once at the Schlenk line, the mixture of solids was suspended in 1.2 mL of toluene and stirred for 10 min at room temperature before the addition of amine (0.5 mmol) followed by chloroalkyne (0.5 mmol). The ampule was closed and heated to 120 °C for the indicated time in an oil bath (Tables 2–4). After the mixture had cooled to room temperature, the solvent was evaporated, the crude mixture was dissolved in 1.5 mL of CDCl_3_, and 1,1,2,2-tetrachloroethane was added as the internal standard. The resulting solution was analyzed via NMR and HRMS (+ESI).

#### (*Z*)-2-Chloro-*N*,1-diphenylethan-1-imine (**3aa**)

NMR yield of 92%, 84% chloroimine *Z*, 8% chloroimine *E*, *Z*:*E* ratio of 91:9, 8% α-chloroketone. Imine *Z*: ^1^H NMR (400 MHz, CDCl_3_) δ 7.97–7.95 (m, 2H), 7.43–7.40 (m, 3H), 7.33–7.29 (m, 2H), 7.09–7.04 (m, 1H), 6.86–6.83 (m, 2H), 4.25 (s, 2H); ^13^C{^1^H} NMR (100 MHz, CDCl_3_) δ 162.0, 149.9, 136.0, 131.2, 129.3, 128.7, 128.0, 124.3, 119.4, 35.4; HRMS (+ESI) for (M + H)^+^ C_14_H_13_ClN^+^ (*m*/*z*) calcd 230.0737, found 230.0731.

#### (*Z*)-2-Chloro-*N*-1-(2,6-diisopropylphenyl)-1-phenylethan-1-imine (**3ab**)

NMR Yield of 98%, 98% chloroimine *Z*, *Z*:*E* ratio of 100:0, 2% α-chloroketone. Imine *Z*: ^1^H NMR (500 MHz, CDCl_3_) δ 8.06–8.04 (m, 1H), 7.48–7.47 (m, 3H), 7.15–7.14 (m, 3H), 4.18 (s, 2H), 2.74–2.68 (m, 2H), 1.17 (d, *J* = 7.0 Hz, 6H), 1.12 (d, *J* = 6.8 Hz, 6 H); ^13^C{^1^H} NMR (125 MHz, CDCl_3_) δ 161.2, 145.2, 138.0, 136.0, 135.6, 131.1, 129.2, 128.7, 128.4, 128.1, 125.4, 124.3, 123.2, 35.9, 28.5, 23.5, 22.6; HRMS (+ESI) for (M + H)^+^ C_20_H_25_ClN^+^ (*m*/*z*) calcd 314.1670, found 314.1664.

#### (*Z*)-2-Chloro-*N*-(4-methoxyphenyl)-1-phenylethan-1-imine (**3ac**)

NMR yield of 93%. 83% chloroimine *Z*, 10% chloroimine *E*, *Z*:*E* ratio of 89:11, 7% α-chloroketone. Imine *Z*: ^1^H NMR (500 MHz, CDCl_3_) δ 7.95–7.94 (m, 2H), 7.40–7.38 (m, 3H), 6.87–6.82 (m, 4H), 4.28 (s, 2H), 3.74 (s, 3H); ^13^C{^1^H} NMR (125 MHz, CDCl_3_) δ 161.9, 156.8, 143.0, 136.3, 131.1, 128.7, 127.8, 121.0, 114.5, 55.6, 35.3; HRMS (+ESI) for (M + H)^+^ C_15_H_15_ClNO^+^ (*m*/*z*) calcd 260.0837, found 260.0834.

#### (*Z*)-2-Chloro-*N*-(4-chlorophenyl)-1-phenylethan-1-imine (**3ad**)

NMR yield of 95%, 86% chloroimine *Z*, 9% chloroimine *E*, *Z*:*E* ratio of 90:10, 5% α-chloroketone. Imine *Z*: ^1^H NMR (400 MHz, CDCl_3_) δ 7.96–7.93 (m, 2H), 7.44–7.38 (m, 3H), 7.27 (d, *J* = 8.6 Hz, 2H), 6.79 (d, *J* = 8.6 Hz, 2H), 4.22 (s, 2H); ^13^C{^1^H} NMR (100 MHz, CDCl_3_) δ 162.7, 148.4, 135.8, 131.5, 129.4, 128.8, 128.3, 128.0, 120.9, 35.2; HRMS (+ESI) for (M + H)^+^ C_14_H_12_Cl_2_N^+^ (*m*/*z*) calcd 264.0341, found 264.0340.

#### (*Z*)-2-Chloro-*N*-(4-nitrophenyl)-1-phenylethan-1-imine (**3ae**)

NMR yield of 87%, 32% chloroimine *Z*, 55% enamines *Z*/*E*, imine:enamine ratio of 37:63, 13% α-chloroketone. Due to the overlap of the signals corresponding to three obtained products, only the main signals are listed: ^1^H NMR (500 MHz, CDCl_3_) δ chloroimine *Z* (CCH_2_Cl) 4.18, chlorenamine *Z* (=CHCl) 5.80, chloroenamine *E* (=CHCl) 5.98, α-chloroketone (COCH_2_Cl) 4.62; ^13^C{^1^H} NMR (125 MHz, CDCl_3_) δ chloroimine *Z* (CCH_2_Cl) 46.4, chloroenamine *Z* (=CHCl) 104.8, chloroenamine *E* (=CHCl) 111.2, α-chloroketone (COCH_2_Cl) 35.5; HRMS (+ESI) for (M + H)^+^ C_14_H_12_ClN_2_O_2_^+^ (*m*/*z*) calcd 275.0558, found 275.0585.

#### (*Z*)-2-Chloro-*N*-(4-hydroxyphenyl)-1-phenylethan-1-imine (**3af**)

NMR yield of 97%, 84% chloroimine *Z*, 13% chloroimine *E*, *Z*:*E* ratio of 93:7, 3% α-chloroketone. Imine *Z*: ^1^H NMR (400 MHz, CDCl_3_) δ 7.92–7.89 (m, 2H), 7.41–7.34 (m, 3H), 6.77–6.71 (m, 4H), 4.27 (s, 2H); ^13^C{^1^H} NMR (100 MHz, CDCl_3_) δ 162.9, 153.2, 142.6, 129.2, 128.8, 128.4, 127.9, 121.3, 116.3, 35.6; HRMS (+ESI) for (M + H)^+^ C_14_H_13_ClNO^+^ (*m*/*z*) calcd 246.0680, found 246.0683.

#### (*Z*)-2-Chloro-1-mesityl-*N*-phenylethan-1-imine (**3ba**)

NMR yield of 97%, 90% chloroimine *Z*, 7% chloroimine *E*, *Z*:*E* ratio of 93:7, 3% α-chloroketone. Imine *Z*: ^1^H NMR (500 MHz, CDCl_3_) δ 7.03 (dd, *J* = 8.4, 7.3 Hz, 2H), 6.90–6.81 (m, 2H), 6.66 (s, 3H), 4.40 (s, 2H), 2.12 (s, 3H), 2.07 (s, 6H); ^13^C{^1^H} NMR (125 MHz, CDCl_3_) δ 167.4, 148.7, 138.7, 134.4, 129.4, 128.6, 128.5, 124.9, 120.4, 48.8, 21.2, 20.4; HRMS (+ESI) for (M + H)^+^ C_17_H_19_ClN^+^ (*m*/*z*) calcd 272.1201, found 272.1200.

#### (*Z*)-2-Chloro-*N*-phenyl-1-(4-methoxyphenyl)ethan-1-imine (**3ca**)

NMR yield of 87%, 81% chloroimine *Z*, 6% chloroimine *E*, *Z*:*E* ratio of 93:7, 13% α-chloroketone. Imine *Z*: ^1^H NMR (500 MHz, CDCl_3_) δ 7.92 (d, *J* = 8.9 Hz, 2H), 7.29 (t, *J* = 7.9 Hz, 2H), 6.90 (d, *J* = 8.9 Hz, 2H), 6.83 (d, *J* = 7.9 Hz, 2H), 4.21 (s, 2H), 3.78 (s, 3H); ^13^C{^1^H} NMR (125 MHz, CDCl_3_) δ 162.1, 161.1, 150.1, 129.7, 129.2, 129.2, 124.1, 119.6, 114.0, 55.5, 35.3; HRMS (+ESI) for (M + H)^+^ C_15_H_15_ClNO^+^ (*m*/*z*) calcd 260.0837, found 260.0836.

#### (*Z*)-2-Chloro-*N*-phenyl-1-[4-(trifluoromethyl)phenyl]ethan-1-imine (**3da**)

NMR yield of 91%, 84% chloroimine *Z*, 7% chloroimine *E*, *Z*:*E* ratio of 92:8, 9% α-chloroketone. Imine *Z*: ^1^H NMR (400 MHz, CDCl_3_) δ 8.06 (d, *J* = 8.2 Hz, 2H), 7.66 (dt, *J* = 8.3, 0.7 Hz, 2H), 7.32 (dd, *J* = 8.2, 7.5 Hz, 2H), 7.09 (ddd, *J* = 7.5, 6.3, 1.2 Hz, 1H), 6.85–6.83 (m, 2H), 4.26 (s, 2H); ^19^F NMR (470 MHz, CDCl_3_) δ −63.29; ^13^C{^1^H} NMR (100 MHz, CDCl_3_) δ 160.9, 149.4, 139.2, 132.8 (q, *J* = 32 Hz), 129.4, 128.4, 124.4. 125.7 (q, *J* = 3.1 Hz), 121.6 (q, *J* = 210 Hz), 119.3, 35.3; HRMS (+ESI) for (M + H)^+^ C_15_H_12_ClF_3_N^+^ (*m*/*z*) calcd 298.0605, found 298.0609.

#### 1-Chloro-*N*-phenyldecan-2-imine (**3ea**)

NMR yield of 78%, 12% chloroimine *Z*, 16% chloroimine *E*, 19% chloroenamine *Z*, 31% chloroenamine *E*, 22% α-chloroketone. Due to the overlap of the signals corresponding to the five obtained products, only the main signals are listed. ^1^H NMR (500 MHz, CDCl_3_) δ chloroimine *Z* (CCH_2_Cl) 3.75, chloroimine *E* (CCH_2_Cl) 4.14, chloroenamine *Z* (=CHCl) 6.03, chloroenamine *E* (=CHCl) 6.35, α-chloroketone (COCH_2_Cl) 3.97; ^13^C{^1^H} NMR (125 MHz, CDCl_3_) δ chloroimine *Z* (CCH_2_Cl) 39.9, chloroimine *E* (CCH_2_Cl) 47.5, chloroenamine *Z* (=CHCl) 113.5, chloroenamine *E* (=CHCl) 117.7, α-chloroketone (COCH_2_Cl) 48.3; HRMS (+ESI) for (M + H)^+^ C_16_H_25_ClN^+^ (*m*/*z*) calcd 266.1670, found 266.1671.

#### (*Z*)-2-Chloro-1-mesityl-*N*-1-(2,6-diisopropylphenyl)ethan-1-imine (**3bb**)

NMR yield of 98%, 96% chloroimine *Z*, 2% chloroimine *E*, *Z*:*E* ratio of 98:2, 2% α-chloroketone. Imine *Z*: ^1^H NMR (500 MHz, CDCl_3_) δ 7.16–7.09 (m, 3H), 6.90 (s, 2H), 4.07 (s, 2H), 3.10–3.04 (m, 2H), 2.42 (s, 6H), 2.27 (s, 3H), 1.22 (d, *J* = 7.0 Hz, 6H), 1.11 (d, *J* = 6.8 Hz, 6H); ^13^C{^1^H} NMR (125 MHz, CDCl_3_) δ 167.9, 144.0, 138.4, 136.5, 135.4, 135.0, 129.4, 124.7, 123.5, 41.5, 28.0, 24.0, 23.4, 21.1, 20.6; HRMS (+ESI) for (M + H)^+^ C_23_H_31_ClN^+^ (*m*/*z*) calcd 356.2140, found 356.2138.

#### (*Z*)-2-Chloro-*N*,1-bis(4-methoxyphenyl)ethan-1-imine (**3cc**)

Yield of 85%, 78% chloroimine *Z*, 2% chloroimine *E*, *Z*:*E* ratio of 93:7, 15% α-chloroketone. Imine *Z*: ^1^H NMR (300 MHz, CDCl_3_) δ 7.92 (d, *J* = 9.0 Hz, 2H), 6.91 (d, *J* = 9.0 Hz, 2H), 6.86–6.83 (m, 4H), 4.25 (s, 2H), 3.79 (s, 3H), 3.74 (s, 3H); ^13^C{^1^H} NMR (75 MHz, CDCl_3_) δ 162.0, 161.1, 156.6, 143.3, 129.6, 125.4, 121.1, 114.5, 114.0, 55.5, 55.5, 35.2; HRMS (+ESI) for (M + H)^+^ C_16_H_17_ClNO_2_^+^ (*m*/*z*) calcd 290.0942, found 290.0940.

#### (*Z*)-2-Chloro-*N*-1-(4-chlorophenyl)-1-(4-methoxyphenyl)ethan-1-imine (**3cd**)

Yield of 86%, 80% chloroimine *Z*, 6% chloroimine *E*, *Z*:*E* ratio of 93:7, 14% α-chloroketone. Imine *Z*: ^1^H NMR (500 MHz, CDCl_3_) δ 7.89 (d, *J* = 9.0 Hz, 2H), 7.24 (d, *J* = 8.6 Hz, 2H), 6.89 (d, *J* = 9.0 Hz, 2H), 6.76 (d, *J* = 8.6 Hz, 2H), 4.18 (s, 2H), 3.78 (s, 3H); ^13^C{^1^H} NMR (125 MHz, CDCl_3_) δ 162.3, 161.8, 148.6, 129.8, 129.3, 129.2, 128.4, 121.1, 114.1, 55.6, 35.2; HRMS (+ESI) for (M + H)^+^ C_15_H_14_Cl_2_NO^+^ (*m*/*z*) calcd 294.0447, found 294.0448.

#### (*Z*)-2-Chloro-*N*-(4-methoxyphenyl)-1-[4-(trifluoromethyl)phenyl]ethan-1-imine (**3dc**)

NMR yield of 93%, 85% chloroimine *Z*, 8% chloroimine *E*, *Z*:*E* ratio of 91:9, 7% α-chloroketone. Imine *Z*: ^1^H NMR (500 MHz, CDCl_3_) δ 8.05 (d, *J* = 8.2 Hz, 2 H), 7.63 (d, *J* = 8.2 Hz, 2H), 6.87–6.82 (m, 4H), 4.29 (s, 2H), 3.73 (s, 3H); ^19^F NMR (470 MHz, CDCl_3_) δ −62.83; ^13^C{^1^H} NMR (125 MHz, CDCl_3_) δ 160.5, 157.2, 142.5, 139.6, 132.5 (q, *J* = 32.6 Hz), 128.2, 125.6 (q, *J* = 3.9 Hz), 124.1 (q, *J* = 272.3 Hz), 121.1, 114.6, 55.6, 35.3; HRMS (+ESI) for (M + H)^+^ C_16_H_14_ClF_3_NO^+^ (*m*/*z*) calcd 328.0711, found 328.0711.

#### (*Z*)-2-Chloro-*N*-(4-chlorophenyl)-1-[4-(trifluoromethyl)phenyl]ethan-1-imine (**3dd**)

NMR yield of 96%, 89% chloroimine *Z*, 7% chloroimine *E*, *Z*:*E* ratio of 93:7, 4% α-chloroketone. Imine *Z*: ^1^H NMR (400 MHz, CDCl_3_) δ 8.04 (d, *J* = 8.2 Hz, 2H), 7.64 (d, *J* = 8.2 Hz, 2H), 7.28 (d, *J* = 8.6 Hz, 2H), 6.78 (d, *J* = 8.6 Hz, 2H), 4.22 (s, 2H); ^19^F NMR (470 MHz, CDCl_3_) δ −62.92; ^13^C{^1^H} NMR (100 MHz, CDCl_3_) δ 161.5, 147.8, 133.0 (q, *J* = 32.6 Hz), 130.3, 129.5, 128.4, 125.7 (q, *J* = 3.8 Hz), 124.0 (d, *J* = 269.0 Hz), 120.8, 116.3 35.1; HRMS (+ESI) for (M + H)^+^ C_15_H_11_Cl_2_F_3_N^+^ (*m*/*z*) calcd 332.0215, found 332.0214.

#### (*Z*/*E*)-1-Chlorodecan-*N*-1-(2,6-disopropylphenyl)-2-imine (**3eb**)

NMR yield of 98%, 64% chloroimine *Z*, 34% chloroimine *E*, 2% α-chloroketone. Due to the overlap of the signals corresponding to two obtained products, only the main signals are listed: ^1^H NMR (500 MHz, CDCl_3_) δ chloroimine *Z* (CCH_2_Cl) 3.65, chloroimine *E* (CCH_2_Cl) 4.24, α-chloroketone (COCH_2_Cl) 3.97; ^13^C{^1^H} NMR (125 MHz, CDCl_3_) δ chloroimine *Z* (CCH_2_Cl) 39.9, chloroimine *E* (CCH_2_Cl) 46.9, α-chloroketone (COCH_2_Cl) 48.3; HRMS (+ESI) for (M + H)^+^ C_22_H_37_ClN^+^ (*m*/*z*) calcd 350.2609, found 350.2617.

#### (*E*)-*N*-(2-Chloro-1-phenylvinyl)-*N*-methylaniline (**6aa**)

Yield of 90%, 90% enamine *E*, 10% α-chloroketone. Enamine *E*: ^1^H NMR (500 MHz, CDCl_3_) δ 7.31–7.29 (m, 2H), 7.26–7.24 (m, 3H), 7.15–7.12 (m, 2H), 6.70–6.68 (m, 3H), 6.28 (s, 1H), 3.15 (s, 3H); ^13^C{^1^H} NMR (125 MHz, CDCl_3_) δ 146.8, 146.0, 136.5, 129.5, 129.1, 128.9, 126.7, 118.1, 113.9, 113.0, 37.9; HRMS (+ESI) for (M + H)^+^ C_15_H_15_ClN^+^ (*m*/*z*) calcd 244.0888, found 244.0885.

#### (*E*)-*N*-(2-Chloro-1-phenylvinyl)-4-methoxy-*N*-methylaniline (**6ab**)

Yield of 68%, 68% enamine *E*, 13% α-chloroketone, 19% chloroalkyne. Enamine *E*: ^1^H NMR (500 MHz, CDCl_3_) δ 7.34–7.32 (m, 2H), 7.27–7.24 (m, 3H), 6.69 (d, *J* = 9.2 Hz, 2H), 6.62 (d, *J* = 9.2 Hz, 2H), 6.10 (s, 1H), 3.64 (s, 3H), 3.12 (s, 3H); ^13^C{^1^H} NMR (125 MHz, CDCl_3_) δ 152.4, 146.4, 141.1, 136.9, 132.0, 128.8, 127.0, 115.6, 114.5, 111.1, 55.6, 38.3; HRMS (+ESI) for (M + H)^+^ C_16_H_17_ClNO^+^ (*m*/*z*) calcd 274.0999, found 274.0996.

#### (*E*)-*N*-(2-Chloro-1-phenylvinyl)-*N*-methyl-4-nitroaniline (**6ac**)

Yield of 87%, 87% enamine *E*, 13% α-chloroketone. Enamine *E*: ^1^H NMR (500 MHz, CDCl_3_) δ 8.00–7.97 (m, 2H), 7.27–7.21 (m, 5H), 6.62–6.59 (m, 2H), 6.70–6.68 (m, 3H), 6.51 (s, 1H), 3.16 (s, 3H); ^13^C{^1^H} NMR (125 MHz, CDCl_3_) δ 152.0, 144.8, 138.9, 134.5, 129.7, 129.4, 126.5, 126.1, 116.0, 112.2, 38.0; HRMS (+ESI) for (M + H)^+^ C_15_H_14_ClN_2_O_2_^+^ (*m*/*z*) calcd 289.0744, found 289.0742.

#### (*E*)-*N*-(2-Chloro-1-phenylvinyl)-*N*-hexylaniline (**6ad**)

Yield of 90%, 90% enamine *E*, 10% α-chloroketone; ^1^H NMR (400 MHz, CDCl_3_) δ 7.39–7.33 (m, 2H), 7.28–7.24 (m, 4H), 7.17 (t, *J* = 7.8 Hz, 2H), 6.77 (d, *J* = 8.3 Hz, 2H), 6.26 (s, 1H), 3.37 (t, *J* = 7.8 Hz, 2H), 1.66–1.62 (m, 2H), 1.34–1.17 (m, 6H), 0.84–0.82 (m, 3H); ^13^C{^1^H} NMR (75 MHz, CDCl_3_) δ 146.7, 136.8, 132.1, 129.2, 128.9, 128.8, 127.1, 118.1, 114.2, 113.3, 50.1, 31.7, 28.4, 26.8, 22.7, 14.1; HRMS (+ESI) for (M + H)^+^ C_20_H_25_ClN^+^ (*m*/*z*) calcd 314.1670, found 314.1665.

#### (*E*)-*N*-{2-Chloro-1-[4-(trifluoromethyl)phenyl]vinyl}-*N*-phenylaniline (**6de**)

Yield of 96%, 74% enamine *E*, 22% enamine *Z*, 4% α-chloroketone. Enamine *E*: ^1^H NMR (500 MHz, CDCl_3_) δ 7.51–7.44 (m, 4H), 7.21–7.14 (m, 2H), 7.04 (d, *J* = 8.0 Hz, 4H), 7.00 (d, *J* = 8.0 Hz, 4H), 6.92–6.85 (m, 2H), 6,32 (s, 1H); ^19^F NMR (470 MHz, CDCl_3_) δ −62.6; ^13^C{^1^H} NMR (75 MHz, CDCl_3_) δ 145.2, 143.2, 131.2, 130.6 (q, *J* = 33 Hz), 129.5, 129.3, 127.5, 127.0, 125.8 (q, *J* = 4 Hz), 124.2 (q, *J* = 235 Hz), 122.8, 122.0, 121.1, 117.9, 115.5; HRMS (+ESI) for (M + H)^+^ C_21_H_16_ClF_3_N^+^ (*m*/*z*) calcd 374.0923, found 374.0923.

### Cyclization Conditions for Indole Derivatives (**7**)

Crude reaction mixtures of chloroimine synthesis were evaporated to dryness and dissolved in 10 mL of dry THF. Pd(OAc)_2_ (11 mg, 0.05 mmol), P(*o*-tolyl)_3_ (46 mg, 0.15 mmol), and CsF (225 mg, 1.5 mmol) were added. The mixture was stirred at 65 °C for 24 h in an oil bath. After complete conversion, the mixture was diluted with Et_2_O and washed with saturated aqueous NH_4_Cl, water, and brine. The organic phase was dried with MgSO_4_ and evaporated. Crude indoles were purified through column chromatography using a 50:1 *n*-hexane/AcOEt mixture as the eluent.

#### 2-Phenyl-1*H*-indole (**7aa**)

Eluent 50:1 *n*-hexane/AcOEt; 154 mg as a white solid; isolated yield of 80%. NMR data were consistent with literature values:^5^^1^H NMR (300 MHz, CDCl_3_) δ 8.32 (s, 1H), 7.66–7.60 (m, 3H), 7.46–7.37 (m, 3H), 7.31 (t, *J* = 7.5 Hz, 1H), 7.21–7.08 (m, 2H), 6.81 (bs, 1H); ^13^C{^1^H} NMR (75 MHz, CDCl_3_) δ 138.1, 137.0, 130.2, 129.5, 129.3, 127.9, 125.4, 122.6, 120.9, 120.5, 111.1, 100.2.

#### 2-Mesityl-1*H*-indole (**7ba**)

Eluent 50:1 *n*-hexane/AcOEt; 167 mg as a white solid; isolated yield of 71%. NMR data were consistent with literature values:^19c^^1^H NMR (500 MHz, CDCl_3_) δ 7.85 (s, 1H), 7.69–7.59 (m, 1H), 7.40–7.34 (m, 1H), 7.20–7.15 (m, 1H), 7.12 (td, *J* = 7.5, 1.1 Hz, 1H), 6.95 (s, 2H), 6.37 (d, *J* = 2.1 Hz, 1H), 2.33 (s, 3H), 2.13 (s, 6H); ^13^C{^1^H} NMR (125 MHz, CDCl_3_) δ 138.4, 138.3, 136.4, 136.1, 130.2, 129.0, 128.3, 121.6, 120.4, 119.9, 110.8, 102.7, 21.2, 20.6.

#### 2-Octyl-1*H*-indole (**7ea**)

Eluent 50:1 *n*-hexane/AcOEt; 156 mg as a thick colorless oil; isolated yield of 68%. NMR data were consistent with literature values:^5^^1^H NMR (500 MHz, CDCl_3_) δ 7.75 (s, 1H), 7.45 (d, *J* = 7.7 Hz, 1H), 7.20 (d, *J* = 7.7 Hz, 1H), 7.06–6.96 (m, 2H), 6.22–6.09 (m, 1H), 2.66 (t, *J* = 7.6 Hz, 2H), 1.63 (p, *J* = 7.6 Hz, 2H), 1.35–1.14 (m, 12H), 0.81 (t, *J* = 6.8 Hz, 3H); ^13^C{^1^H} NMR (125 MHz, CDCl_3_) δ 140.2, 136.0, 129.0, 121.0, 119.9, 119.7, 110.4, 99.6, 32.0, 29.6, 29.5, 29.4, 29.3, 28.4, 22.8, 14.2.

### General Reduction of Chloroimine to β-Chloroamine (**8**)

Crude reaction mixtures of chloroimine synthesis were evaporated to dryness, dissolved in 12 mL of dry MeOH, and cooled to 0 °C. AcOH (56 μL, 1.0 mmol) and NaBH_3_CN (75 mg, 1.1 mmol) were added. The mixture was stirred at rt for 15 h. After complete conversion, the mixture was evaporated, and the residue dissolved in Et_2_O and washed with water and brine. The organic phase was dried with MgSO_4_ and evaporated. Crude amines were purified through column chromatography using a 50:1 *n*-hexane/AcOEt mixture as the eluent. Due to adsorption of the product onto the stationary phase, some loss of yield was observed.

#### *N*-(2-Chloro-1-phenylethyl)aniline (**8aa**)

Eluent 50:1 *n*-hexane/AcOEt; 146 mg as a colorless oil; isolated yield of 63%. NMR data were consistent with literature values:^8b^^1^H NMR (500 MHz, CDCl_3_) δ 7.43 (dd, *J* = 7.5, 2.1 Hz, 2H), 7.38 (td, *J* = 7.5, 2.2 Hz, 2H), 7.35–7.28 (m, 1H), 7.14 (td, *J* = 8.0, 2.9 Hz, 2H), 6.73 (td, *J* = 7.5, 3.0 Hz, 1H), 6.58 (dd, *J* = 8.3, 2.8 Hz, 2H), 4.64 (dt, *J* = 7.4, 3.7 Hz, 1H), 4.46 (bs, 1H), 3.89 (ddd, *J* = 11.4, 3.7, 1.8 Hz, 1H), 3.74 (ddd, *J* = 11.4, 7.5, 1.8 Hz, 1H); ^13^C{^1^H} NMR (125 MHz, CDCl_3_) δ 146.9, 140.1, 129.3, 129.0, 128.2, 126.9, 118.4, 114.0, 59.5, 49.2.

#### *N*-(2-Chloro-1-phenylethyl)-2,6-diisopropylaniline (**8ab**)

Eluent 50:1 *n*-hexane/AcOEt; 215 mg as a colorless oil; isolated yield of 68%; ^1^H NMR (300 MHz, CDCl_3_) δ 7.30–7.22 (m, 5H), 7.01–6.97 (m, 3H), 4.17 (dd, *J* = 6.6, 4.6 Hz, 1H), 3.84 (dd, *J* = 10.9, 4.6 Hz, 1H), 3.78 (dd, *J* = 10.9, 6.6 Hz, 1H), 3.49 (br s, 1H), 3.11 (m, 2H), 1.13 (d, *J* = 6.8 Hz, 6H), 1.01 (d, *J* = 6.8 Hz, 6H); ^13^C{^1^H} NMR (75 MHz, CDCl_3_) δ 142.8, 141.0, 140.5, 128.7, 127.9, 127.1, 124.1, 123.8, 65.3, 48.1, 27.9, 24.3; HRMS (+ESI) for (M + H)^+^ C_20_H_27_ClN^+^ (*m*/*z*) calcd 316.1827, found 316.1818.

#### 4-Chloro-*N*-(2-chloro-1-phenylethyl)aniline (**8ad**)

Eluent 50:1 *n*-hexane/AcOEt; 151 mg as a colorless oil; isolated yield of 57%. NMR data were consistent with literature values:^8b^^1^H NMR (500 MHz, CDCl_3_) δ 7.45–7.33 (m, 4H), 7.31 (dd, *J* = 5.9, 2.9 Hz, 1H), 7.10–7.01 (m, 2H), 6.51–6.43 (m, 2H), 4.57 (dd, *J* = 7.9, 4.2 Hz, 1H), 4.47 (bs, 1H), 3.87 (dd, *J* = 11.4, 4.2 Hz, 1H), 3.69 (dd, *J* = 11.4, 7.9 Hz, 1H); ^13^C{^1^H} NMR (125 MHz, CDCl_3_) δ 145.4, 139.6, 129.1, 129.1, 128.3, 126.8, 123.0, 115.1, 59.5, 49.1.

#### *N*-[2-Chloro-1-(4-methoxyphenyl)ethyl]aniline (**8ca**)

Eluent 50:1 *n*-hexane/AcOEt; 142 mg as a colorless oil; isolated yield of 55%; ^1^H NMR (300 MHz, CDCl_3_) δ 7.28–7.21 (m, 2H), 7.08–7.00 (m, 2H), 6.84–6.78 (m, 2H), 6.68–6.60 (m, 1H), 6.51–6.45 (m, 2H), 4.49 (dd, *J* = 7.8, 4.5 Hz, 1H), 4.31 (s, 1H), 3.76 (dd, *J* = 11.3, 4.4 Hz, 1H), 3.71 (s, 3H), 3.62 (dd, *J* = 11.3, 7.8 Hz, 1H); ^13^C{^1^H} NMR (75 MHz, CDCl_3_) δ 159.8, 147.3, 132.3, 129.6, 128.2, 118.6, 114.7, 114.3, 59.2, 55.7, 49.5; HRMS (+ESI) for (M + H)^+^ C_15_H_17_ClNO^+^ (*m*/*z*) calcd 262.0982, found 262.0975.

#### *N*-{2-Chloro-1-[4-(trifluoromethyl)phenyl]ethyl}aniline (**8da**)

Eluent 50:1 *n*-hexane/AcOEt; 186 mg as a colorless oil; isolated yield of 62%; ^1^H NMR (300 MHz, CDCl_3_) δ 7.55 (d, *J* = 8.4 Hz, 2H), 7.46 (d, *J* = 8.4 Hz, 2H), 7.09–7.02 (m, 2H), 6.70–6.63 (m, 1H), 6.48–643 (m, 2H), 4.63 (dd, *J* = 7.2, 4.2 Hz, 1H), 4.4 (br s, 1H), 3.83 (dd, *J* = 11.4, 4.2 Hz, 1H), 3.66 (dd, *J* = 11.4, 7.2 Hz, 1H); ^19^F (282 MHz, CDCl_3_) δ −63.00; ^13^C{^1^H} NMR (125 MHz, CDCl_3_) δ 146.4, 144.4, 130.7 (q, *J* = 32.5 Hz), 129.4, 127.4, 126.0 (q, *J* = 3.7 Hz), 124.1 (q, *J* = 272 Hz), 118.8, 114.0, 59.0, 48.9; HRMS (+ESI) for (M + H)^+^ C_15_H_14_ClF_3_N^+^ (*m*/*z*) calcd 300.0761, found 300.0752.

## Supporting Information Available

Experimental procedures, product characterization data, and NMR, IR, and HRMS spectra (PDF)
FAIR data, including the primary NMR FID files, for compounds **1b**, **1e**, **3aa–ae**, **3ba**, **3bb**, **3ca**, **3cc**, **3cd**, **3da**, **3dc**, **3dd**, **3ea**, **3eb**, **6aa–ad**, **6de**, **7aa**, **7ba**, **7ea**, **8aa**, **8ab**, **8ad**, **8ca**, and **8da** (ZIP)

## Dedication

Dedicated to Prof. Joan Bosch on the occasion of his 75th birthday.
