# Supplementary material for: Straightforward Synthesis of α-Chloromethylketimines Catalyzed by Gold(I). A Clean Way to Building Blocks
Source: J Org Chem. 2022 Feb 22;87(5):3114–22. doi: 10.1021/acs.joc.1c02877 (PMC10391627; doi:10.1021/acs.joc.1c02877)
Supplement: Supplementary file 1 — jo1c02877_si_001.pdf [file jo1c02877_si_001.pdf]

Straightforward synthesis of α-chloromethylketimines catalyzed by gold(I). A clean way to building-blocks.

Jeymy T. Sarmiento,[a] María Cárcel,[a] Carmen Ramírez de Arellano,[a] Teresa Varea,[a] Gregorio Asensio[a] and Andrea Olmos[a,]*

[a]Departamento de Química Orgánica, Universidad de Valencia. Av. Vicente Andrés Estellés S/N. 46100 Burjassot (SPAIN).

Supporting Information

Content

1. General information	S3
2. Chloroalkyne syntheses	S3
3. Spectral and analysis data for **1b** and **1e**	S4
4. Gold-catalyzed hydroamination reactions	S4
5. NMR study of **3da** formation in toluene-d_8	S5
6. Chloroimine cyclization to indoles **7**	S5
7. General procedure for chloroimine reduction to chloroamines **8**	S5
8. Spectral and analysis data for compounds **3**, **6**, **7** and **8**	S6
9. NMR, IR and HRMS spectra	S15
10. References	S93

1. General information.

Chloromethylimines are products very sensitive to hydrolysis. All transformations have been performed using common Schlenk techniques or in a glove box. Toluene, toluene-d_8, and THF have been dried with sodium prior to use. Deuterated chloroform and liquid amines have been distilled from CaH_2 prior to use. Methanol was acquired anhydrous and used as received. NMR analysis have been performed in a Bruker AvanceIII 300 spectrometer, a Bruker AV400 spectrometer, or a Bruker Neo500 spectrometer. NMR data have been processed using MestReNova™ or TopSpin™. Residual signals of deuterated solvents have been used as internal reference ($CDCl_3$ at 7.26 ppm in ^{1}H-NMR and 77.16 ppm in ^{13}C-NMR, toluene-d_8 at 2.08 ppm and 20.43 ppm for methyl group in ^{1}H-NMR and ^{13}C-NMR respectively). IR spectra have been recorded on a Thermo Scientific Nicolet iS10 and processed with Omnic™. IR frequencies have been rounded to 1 cm^{-1}. GC-MS analysis were performed in an Agilent 5977A equipped with a 30mx0.25mx0.25μm HP-5ms ui column. HRMS (+ESI) analysis have been performed in AB SCIEX TripleTOF™ 5600 LC/MS/MS System and data have been processed using PeakView™. Elemental analyses have been performed in a Thermofisher Flashmart Eager 200.

2. Chloroalkyne synthesis.

1-chloroalkynes were prepared through a modified procedure of reference 1.

A 2.5M solution of *n*-butyllithium in hexanes (2.6 mL, 6.5 mmol, 1.3 eq.) was slowly added to the corresponding alkyne (5 mmol, 1 eq.) solved in 12 mL of THF at -78 ^oC. After stirring 1 hour at that temperature, a suspension of *N*-chlorosuccinimide (868 mg, 6.5 mmol, 1.3 eq) in 5 mL THF was added via cannula. The cooling bath was removed, and the mixture was stirred for 5 additional hours at room temperature. The reaction was hydrolyzed with 20 mL of water and the aqueous phase extracted with Et_2O (2x20 mL). Organic phases were joined, dried with anhydrous magnesium sulfate, and concentrated. Crude product was purified through column chromatography using *n*-hexane as eluent.

Chloroalkynes **1a**, **1c**, **1d**, and **1f** were obtained in 60-80 % yield and their NMR data were consistent with literature values.[1,2]

2-(chloroethynyl)-1,3,5-trimethylbenzene (**1b**) was obtained as a white solid, 732 mg. Yield 82%.

1-chloro-1-decyne (**1e**) was obtained as a colorless oil, 536 mg. Yield 62 %.

3. Spectral and analysis data for 1b and 1e.

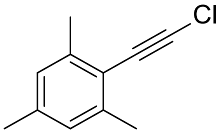<br>**1b**	**2-(chloroethynyl)-1,3,5-trimethylbenzene.** Eluent *n*-hexane. 732 mg as a white solid. Yield 82 %.<br>^{1}H NMR (500 MHz, $CDCl_3$) δ 6.85 (s, 2H), 2.39 (s, 6H), 2.28 (s, 3H); $^{13}C\{^1H\}$ NMR (125 MHz, $CDCl_3$) δ 141.0, 138.1, 127.7, 119.1, 74.3, 67.6, 21.4, 21.0; IR (cm^{-1}) 2942, 2914, 2211, 1478; Elemental analysis calculated for $C_{11}H_{11}Cl$: C, 73.95; H, 6.21; found: 73.05; H, 6.05.
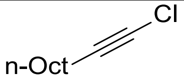<br>**1e**	**1-chloro-1-decyne**. Eluent *n*-hexane. 536 mg as a colorless oil. Yield 62%.<br>^{1}H NMR (300 MHz, $CDCl_3$) δ 2.16 (t, *J* = 7.0 Hz, 2H), 1.55-1.45 (m, 2H), 1.39-1.26 (m, 10H), 0.91-0.86 (m, 3H). $^{13}C\{^1H\}$ NMR (75 MHz, $CDCl_3$) δ 70.0, 57.1, 32.0, 29.3, 29.2, 29.0, 28.5, 22.8, 18.9, 14.2; IR (cm^{-1}): 2928, 2856, 2243, 2214, 1464, 851. Elemental analysis calculated for $C_{10}H_{17}Cl$: C, 69.55; H, 9.92; found: 68.95; H, 9.37.

4. Gold catalyzed hydroamination reactions.

IPrAuCl (3 mg, 5 μmol, 1 mol %) and NaBArF (6.6 mg, 7.5 μmol, 1.5 mol %) were weighted in a glove box and introduced in an ampoule provided with J. Young valve. Once at the Schlenk line the mixture of solids was suspended in 1.2 mL of toluene and stirred for 10 minutes at room temperature before addition of amine (0.5 mmol) followed by chloroalkyne (0.5 mmol). The ampoule was closed and heated to 120 °C for the indicated time in an oil bath (Tables 2, 3 and 4). After cooling to room temperature, solvent was evaporated, and the crude mixture was solved in 1.5 mL of $CDCl_3$ and 1,1,2,2-tetrachloroethane was added as internal standard. The resulting solution was analyzed through NMR and HRMS (+ESI). *Z* and *E* diastereoisomers were determined through NOESY experiments. Species present in the tautomeric equilibria of compounds **3ea** and **3eb** were identified also with the aid of HSQC and GC-MS experiments. Signals appearing on the ^{1}H NMR spectra in the region 1.0-1.5 ppm correspond to isopropyl groups of IPrAuCl catalyst present in the crude mixture.

5. NMR study of 3da formation in toluene-d_8.

IPrAuCl (3 mg, 5 μmol, 2 mol %) and NaBArF (6.6 mg, 7.5 5 μmol, 3 mol %) were weighted in a glove box and introduced in an NMR tube provided with a J. Young valve. toluene-d_8 was added and the tube introduced in the NMR equipment preheated at 80 °C. Lock and shims were fitted and the tube was ejected to add aniline **2a** (50 μL, 0.25 mmol, 1 eq.) followed by 1-(chloroethynyl)-4-(trifluoromethyl)benzene **1d** (27.5 μL, 0.25 mmol, 1 eq.) under Ar. The Young valve was closed, and the tube charged on the equipment. ^{1}H-NMR spectra of 16 scans were recorded each 300 seconds.

6. Chloroimine cyclization to indoles 7.

Crude reaction mixtures of chloroimine synthesis were evaporated to dryness and solved in 10 mL of dry THF. $Pd(OAc)_2$ (22 mg, 0.1 mmol), P(*o*-tolyl) (46, 0.3 mmol) and CsF (225 mg, 3 mmol) were added. The mixture was stirred at 65 °C for 24 h in an oil bath. The mixture was diluted with Et_2O and washed saturated aqueous NH_4Cl, water, and brine. Organic phase was dried with $MgSO_4$ and evaporated. Crude indoles were purified through column chromatography using *n*-hexane:AcOEt 50:1 as eluent.

7. General procedure for chloroimine reduction to chloroamines 8.

Crude reaction mixtures of chloroimine synthesis were evaporated to dryness, solved in 12 mL of dry MeOH and cooled to 0 °C. ACOH (56 μL, 1 mmol) and $NaBH_3CN$ (70 mg, 1.1 mmol) were added. The mixture was stirred at rt for 15 h. After complete conversion, the mixture was evaporated, and the residue solved in Et_2O and washed with water and brine. Organic phase was dried with $MgSO_4$ and evaporated. Crude amines were purified through column chromatography using *n*-hexane:AcOEt 50:1 as eluent. Due to product adsorption onto the stationary phase some yield loss was observed.

8. Spectral and analysis data for compounds 3, 6, 7 and 8.

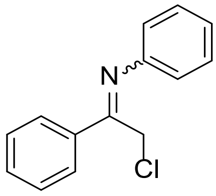 **3aa**	**(*Z*)-2-chloro-*N*,1-diphenylethan-1-imine.** NMR Yield 92 %. Chlorimine *Z*: 84 %, chlorimine *E*: 8 %, ratio *Z:E* 91:9, α-chloroketone: 8 %. Imine *Z*: ^{1}H NMR (400 MHz, $CDCl_3$) δ 7.97-7.95 (m, 2H), 7.43-7.40 (m, 3H), 7.33-7.29 (m, 2H), 7.09-7.04 (m, 1H), 6.86-6.83 (m, 2H), 4.25 (s, 2H); $^{13}C\{^{1}H\}$ NMR (100 MHz, $CDCl_3$) δ 162.0, 149.9, 136.0, 131.2, 129.3, 128.7, 128.0, 124.3, 119.4, 35.4. HRMS (+ESI) for $(M+H)^+$ $C_{14}H_{13}ClN^+$ (*m/z*): calc. 230.0737; found 230.0731.
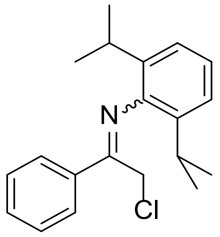 **3ab**	**(*Z*)-2-chloro-*N*-1-(2,6-diisopropylphenyl)-1-phenylethan-1-imine.** NMR Yield 98 %. Chlorimine *Z*: 98 %, ratio *Z:E* 100:0, α-chloroketone: 2 %. Imine *Z*: ^{1}H NMR (500 MHz, $CDCl_3$) δ 8.06-8.04 (m, 1H), 7.48-7.47 (m, 3H), 7.15-7.14 (m, 3H), 4.18 (s, 2H), 2.74-2.68 (m, 2H), 1.17 (d, *J* = 7.0 Hz, 6H), 1.12 (d, *J* = 6.8 Hz, 6 H); $^{13}C\{^{1}H\}$ NMR (125 MHz, $CDCl_3$) δ 161.2, 145.2, 138.0, 136.0, 135.6, 131.1, 129.2, 128.7, 128.4, 128.1, 125.4, 124.3, 123.2, 35.9, 28.5, 23.5, 22.6; HRMS (+ESI) for $(M+H)^+$ $C_{20}H_{25}ClN^+$ (*m/z*): calc. 314.1670; found 314.1664.
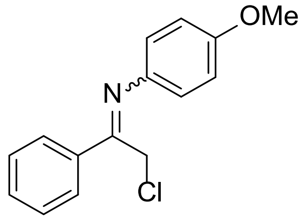 **3ac**	**(*Z*)-2-chloro-*N*-(4-methoxyphenyl)-1-phenylethan-1-imine.** NMR Yield 93 %. Chlorimine *Z*: 83 %, chlorimine *E*: 10 %, ratio *Z:E* 89:11, α-chloroketone: 7 %. Imine *Z*: ^{1}H NMR (500 MHz, $CDCl_3$) δ 7.95-7.94 (m, 2H), 7.40-7.38 (m, 3 H), 6.87-6.82 (m, 4H), 4.28 (s, 2H), 3.74 (s, 3H); $^{13}C\{^{1}H\}$ NMR (125 MHz, $CDCl_3$) δ 161.9, 156.8, 143.0, 136.3, 131.1, 128.7, 127.8, 121.0, 114.5, 55.6, 35.3; HRMS (+ESI) for $(M+H)^+$ $C_{15}H_{15}ClNO^+$ (*m/z*): calc. 260.0837; found 260.0834.
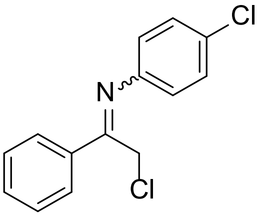 **3ad**	**(*Z*)-2-chloro-*N*-(4-chlorophenyl)-1-phenylethan-1-imine.** NMR Yield 95 %. Chlorimine *Z*: 86 %, chlorimine *E*: 9 %, ratio *Z:E* 90:10, α-chloroketone: 5 %. Imine *Z*: ^{1}H NMR (400 MHz, $CDCl_3$) δ 7.96-7.93 (m, 2H), 7.44-7.38 (m, 3H), 7.27 (d, *J* = 8.6 Hz, 2H), 6.79 (d, *J* = 8.6 Hz) 2H), 4.22 (s, 2H); $^{13}C\{^{1}H\}$ NMR (100 MHz, $CDCl_3$) δ

	162.7, 148.4, 135.8, 131.5, 129.4, 128.8, 128.3, 128.0, 120.9, 35.2; HRMS (+ESI) for $(M+H)^+$ $C_{14}H_{12}Cl_2N^+$ (*m/z*): calc. 264.0341; found 264.0340.
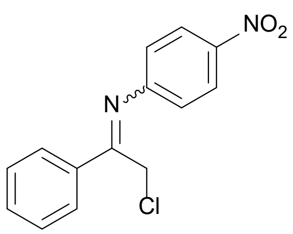<br>**3ae**	**(*Z*)-2-chloro-*N*-(4-nitrophenyl)-1-phenylethan-1-imine.** NMR Yield 87 %. Chloroimine *Z*: 32 %, enamines *Z/E*: 55 %, ratio imine/enamine 37:63, α-chloroketone: 13 %. Due to the overlap of the signals corresponding to three obtained products, only main signals are listed.<br>^{1}H NMR (500 MHz, $CDCl_3$) δ chloroimine *Z* (CCH_2Cl): 4.18, chlorenamine *Z* (=CHCl): 5.80, chloroenamine *E* (=CHCl): 5.98, α-chloroketone ($COCH_2Cl$) 4.62; $^{13}C\{^1H\}$ NMR (125 MHz, $CDCl_3$) δ chloroimine *Z* (CCH_2Cl) 46.4; chloroenamine *Z* (=CHCl) 104.8; chloroenamine *E* (=CHCl) 111.2, α-chloroketone ($COCH_2Cl$) 35.5; HRMS (+ESI) for $(M+H)^+$ $C_{14}H_{12}ClN_2O_2^+$ (m/z): calc. 275.0558; found 275.0585.
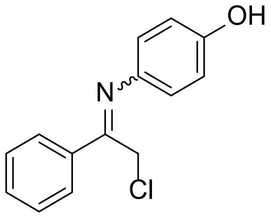<br>**3af**	**(*Z*)-2-chloro-*N*-(-4-hydroxyphenyl)-1-phenylethan-1-imine.** NMR Yield 97 %. Chlorimine *Z*: 84 %, chlorimine *E*: 13 %, ratio *Z:E* 93:7, α-chloroketone: 3 %.<br>Imine *Z*: ^{1}H NMR (400 MHz, $CDCl_3$) δ 7.92-7.89 (m, 2H), 7.41-7.34 (m, 3H); 6.77-6.71 (m, 4H), 4.27 (s, 2H); $^{13}C\{^1H\}$ NMR (100 MHz, $CDCl_3$) δ 162.9, 153.2, 142.6, 129.2, 128.8, 128.4, 127.9, 121.3, 116.3, 35.6; HRMS (+ESI) for $(M+H)^+$ $C_{14}H_{13}ClNO^+$ (*m/z*): calc. 246.0680; found 246.0683.
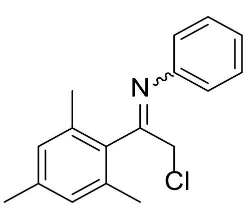<br>**3ba**	**(*Z*)-2-chloro-1-mesityl-*N*-phenylethan-1-imine.** NMR Yield 97 %. Chlorimine *Z*: 90 %, chlorimine *E*: 7 %, ratio *Z:E* 93:7, α-chloroketone: 3 %.<br>Imine *Z*: ^{1}H NMR (500 MHz, $CDCl_3$) δ 7.03 (dd, *J* = 8.4, 7.3 Hz, 2H), 6.90-6.81 (m, 2H), 6.66 (s, 3H), 4.40 (s, 2H), 2.12 (s, 3H), 2.07 (s, 6H); $^{13}C\{^1H\}$ NMR (125 MHz, $CDCl_3$) δ 167.4, 148.7, 138.7, 134.4, 129.4, 128.6, 128.5, 124.9, 120.4, 48.8, 21.2, 20.4; HRMS (+ESI) for $(M+H)^+$ $C_{17}H_{19}ClN^+$ (*m/z*): calc. 272.1201; found 272.1200.

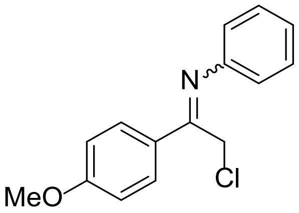 **3ca**	**(*Z*)-2-chloro-*N*-phenyl-1-(4-methoxyphenyl)ethan-1-imine.** NMR Yield 87 %. Chlorimine *Z*: 81 %, chlorimine *E*: 6 %, ratio *Z:E* 93:7, α-chloroketone: 13 %. Imine *Z*: ^{1}H NMR (500 MHz, $CDCl_3$) δ 7.92 (d, *J* = 8.9 Hz, 2H), 7.29 (t, *J* = 7.9 Hz, 2H), 6.90 (d, *J* = 8.9 Hz, 2H), 6.83 (d, *J* = 7.9 Hz, 2H), 4.21 (s, 2H), 3.78 (s, 3H); $^{13}C\{^{1}H\}$ NMR (125 MHz, $CDCl_3$) δ 162.1, 161.1, 150.1, 129.7, 129.2, 129.2, 124.1, 119.6, 114.0, 55.5, 35.3; HRMS (+ESI) for $(M+H)^+$ $C_{15}H_{15}ClNO^+$ (*m/z*): calc. 260.0837; found 260.0836.
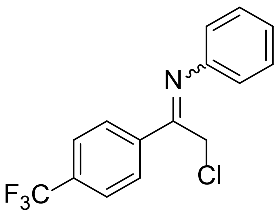 **3da**	**(*Z*)-2-chloro-*N*-phenyl-1-(4-(trifluoromethyl)phenyl)ethan-1-imine.** NMR Yield 91 %. Chlorimine *Z*: 84%, chlorimine *E*: 7 %, ratio *Z:E* 92:8, α-chloroketone: 9 %. Imine *Z*: ^{1}H NMR (400 MHz, $CDCl_3$) δ 8.06 (d, *J* = 8.2 Hz, 2H), 7.66 (dt, *J* = 8.3, 0.7 Hz, 2H), 7.32 (dd, *J* = 8.2, 7.5 Hz, 2H), 7.09 (ddd, *J* = 7.5, 6.3, 1.2 Hz, 1H), 6.85-6.83 (m, 2H), 4.26 (s, 2H); ^{19}F NMR (470 MHz, $CDCl_3$) δ -63.29; $^{13}C\{^{1}H\}$ NMR (100 MHz, CDCl3) δ 160.9, 149.4, 139.2, 132.8 (q, *J* = 32 Hz), 129.4, 128.4, 124.4. 125.7 (q, *J* = 3.1 Hz), 121.6 (q, *J* = 210 Hz), 119.3, 35.3; HRMS (+ESI) for $(M+H)^+$ $C_{15}H_{12}ClF_3N^+$ (*m/z*): calc. 298.0605; found 298.0609.
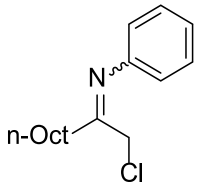 **3ea**	**1-chloro-*N*-phenyldecan-2-imine.** NMR Yield 78%. Chlorimine *Z*: 12%, chlorimine *E*: 16 %, chloroenamine *Z*: 19 %, chloroenamine *E*: 31%, α-chloroketone: 22 %. Due to the overlap of the signals corresponding to the five obtained products, only main signals are listed. ^{1}H NMR (500 MHz, $CDCl_3$) δ chloroimine *Z* ($CC\underline{H}_2Cl$) 3.75; chloroimine *E* ($CC\underline{H}_2Cl$) 4.14; chloroenamine *Z* ($=C\underline{H}Cl$) 6.03; chloroenamine *E* ($=C\underline{H}Cl$) 6.35, α-chloroketone ($COC\underline{H}_2Cl$) 3.97; $^{13}C\{^{1}H\}$ NMR (125 MHz, $CDCl_3$) δ chloroimine *Z* ($C\underline{C}H_2Cl$) 39.9; chloroimine *E* ($C\underline{C}H_2Cl$) 47.5; chloroenamine *Z* ($=\underline{C}HCl$) 113.5; chloroenamine *E* ($=\underline{C}HCl$) 117.7, α-chloroketone ($CO\underline{C}H_2Cl$) 48.3; HRMS (+ESI) for $(M+H)^+$ $C_{16}H_{25}ClN^+$ (*m/z*): calc. 266.1670; found 266.1671.

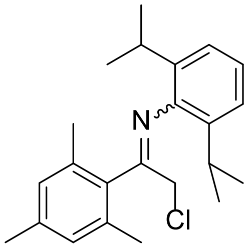<br>**3bb**	**(*Z*)-2-chloro-1-mesityl-*N*-1-(2,6-diisopropylphenyl)ethan-1-imine.** NMR Yield 98 %. Chlorimine *Z*: 96%, chlorimine *E*: 2 %, ratio *Z:E* 98:2, α-chloroketone: 2 %.<br>Imine *Z*: ^{1}H NMR (500 MHz, $CDCl_3$) δ 7.16-7.09 (m, 3H), 6.90 (s, 2H), 4.07 (s, 2H), 3.10-3.04 (m, 2H), 2.42 (s, 6H), 2.27 (s, 3H), 1.22 (d, *J* = 7.0 Hz, 6H), 1.11 (d, *J* = 6.8 Hz, 6H); $^{13}C\{^{1}H\}$ NMR (125 MHz, $CDCl_3$) δ 167.9, 144.0, 138.4, 136.5, 135.4, 135.0, 129.4, 124.7, 123.5, 41.5, 28.0, 24.0, 23.4, 21.1, 20.6; HRMS (+ESI) for $(M+H)^+$ $C_{23}H_{31}ClN^+$ (*m/z*): calc. 356.2140; found 356.2138.
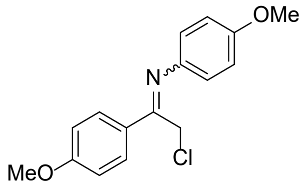<br>**3cc**	**(*Z*)-2-chloro-*N*,1-bis(4-methoxyphenyl)ethan-1-imine.** Yield 85 %. Chlorimine *Z*: 78%, chlorimine *E*: 2 %, ratio *Z:E* 93:7, α-chloroketone: 15 %.<br>Imine *Z*: ^{1}H NMR (300 MHz, $CDCl_3$) δ 7.92 (d, *J* = 9.0 Hz, 2H), 6.91 (d, *J* = 9.0 Hz, 2H), 6.86-6.83 (m, 4H), 4.25 (s, 2H), 3.79 (s, 3H), 3.74 (s, 3H); $^{13}C\{^{1}H\}$ NMR (75 MHz, $CDCl_3$) δ 162.0, 161.1, 156.6, 143.3, 129.6, 125.4, 121.1, 114.5, 114.0, 55.5, 55.5, 35.2; HRMS (+ESI) for $(M+H)^+$ $C_{16}H_{17}ClNO_2^+$ (*m/z*): calc. 290.0942; found 290.0940.
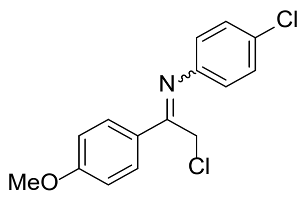<br>**3cd**	**(*Z*)-2-chloro-*N*-1-(4-chlorophenyl)-1-(4-methoxyphenyl)ethan-1-imine.** Yield 86 %. Chlorimine *Z*: 80%, chlorimine *E*: 6 %, ratio *Z:E* 93:7, α-chloroketone: 14 %.<br>Imine *Z*: ^{1}H-NMR (500 MHz, $CDCl_3$) δ 7.89 (d, *J* = 9.0 Hz, 2H), 7.24 (d, *J* = 8.6 Hz, 2H), 6.89 (d, *J* = 9.0 Hz, 2H), 6.76 (d, *J* = 8.6 Hz, 2H), 4.18 (s, 2H), 3.78 (s, 3H); $^{13}C\{^{1}H\}$ NMR (125 MHz, $CDCl_3$) δ 162.3, 161.8, 148.6, 129.8, 129.3, 129.2, 128.4, 121.1, 114.1, 55.6, 35.2; HRMS (+ESI) for $(M+H)^+$ $C_{15}H_{14}Cl_2NO^+$ (*m/z*): calc. 294.0447; found 294.0448.
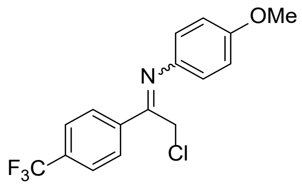	**(*Z*)-2-chloro-*N*-(4-methoxyphenyl)-1-(4-(trifluoromethyl)phenyl)ethan-1-imine.** NMR Yield: 93 %. Chlorimine Z: 85 %, chlorimine E: 8 %, ratio Z:E 91:9, α-chloroketone 7 %.<br>Imine *Z*: ^{1}H NMR (500 MHz, $CDCl_3$) δ 8.05 (d, *J* = 8.2 Hz, 2 H), 7.63 (d, *J* = 8.2 Hz, 2H), 6.87-6.82 (m, 4H), 4.29 (s, 2H), 3.73 (s, 3H); ^{19}F NMR (470 MHz, $CDCl_3$) δ -62.83; $^{13}C\{^{1}H\}$

**3dc**	NMR (125 MHz, $CDCl_3$) δ 160.5, 157.2, 142.5, 139.6, 132.5 (q, *J* = 32.6 Hz), 128.2, 125.6 (q, *J* = 3.9 Hz), 124.1 (q, *J* = 272.3 Hz), 121.1, 114.6, 55.6, 35.3; HRMS (+ESI) for $(M+H)^+$ $C_{16}H_{14}ClF_3NO^+$ (*m/z*): calc. 328.0711; found 328.0711.
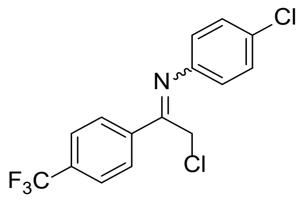 **3dd**	**(*Z*)-2-chloro-*N*-(4-chlorophenyl)-1-(4-(trifluoromethyl)phenyl)ethan-1-imine.** NMR Yield 96 %. Chlorimine Z: 89 %, chlorimine E: 7 %, Ratio Z:E 93:7, α-chloroketone: 4 %.<br>Imine *Z*: ^{1}H NMR (400 MHz, $CDCl_3$) δ 8.04 (d, *J* = 8.2 Hz, 2H), 7.64 (d, *J* = 8.2 Hz, 2H), 7.28 (d, *J* = 8.6 Hz, 2H), 6.78 (d, *J* = 8.6 Hz, 2H), 4.22 (s, 2H); ^{19}F NMR (470 MHz, $CDCl_3$) δ -62.92; ^{13}C{^{1}H} NMR (100 MHz, $CDCl_3$) δ 161.5, 147.8, 133.0 (q, *J* = 32.6 Hz), 130.3, 129.5, 128.4, 125.7 (q, *J* = 3.8 Hz), 124.0 (q, *J* = 269.0 Hz), 120.8, 116.3; 35.2; HRMS (+ESI) for $(M+H)^+$ $C_{15}H_{11}Cl_2F_3N^+$ (*m/z*): calc. 332.0215; found 332.0214.
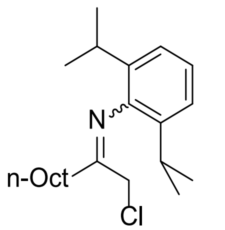 **3eb**	**(*Z*/E)-1-chloro-decan-*N*-1-(2,6-disopropylphenyl)-2-imine.** NMR Yield 98 %. Chlorimine *Z*: 64%, chlorimine *E*: 34 %, α-chloroketone: 2 %. Due to the overlap of the signals corresponding to two obtained products, only main signals are listed.<br>^{1}H NMR (500 MHz, $CDCl_3$) δ chloroimine *Z* (CC$\underline{H}_2$Cl) 3.65; chloroimine *E* (CC$\underline{H}_2$Cl) 4.24, α-chloroketone (COC$\underline{H}_2$Cl) 3.97; ^{13}C{^{1}H} NMR (125 MHz, $CDCl_3$) δ chloroimine *Z* (C$\underline{C}$$H_2$Cl) 39.9; chloroimine *E* (C$\underline{C}$$H_2$Cl) 46.9; α-chloroketone (CO$\underline{C}$$H_2$Cl) 48.3; HRMS (+ESI) for $(M+H)^+$ $C_{22}H_{37}ClN^+$ (*m/z*): calc. 350.2609; found 350.2617.
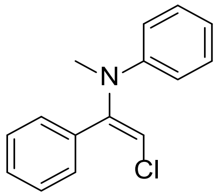 **6aa**	**(*E*)-*N*-(2-chloro-1-phenylvinyl)-*N*-methylaniline.** Yield 90 %. Enamine E 90 %, α-chloroketone: 10 %.<br>Enamine *E*: ^{1}H NMR (500 MHz, $CDCl_3$) δ 7.31-7.29 (m, 2H), 7.26-7.24 (m, 3H), 7.15-7.12 (m, 2H), 6.70-6.68 (m, 3H), 6.28 (s, 1H), 3.15 (s, 3H); ^{13}C{^{1}H} NMR (125 MHz, $CDCl_3$) δ 146.8, 146.0, 136.5, 129.5, 129.1, 128.9, 126.7, 118.1, 113.9, 113.0, 37.9; HRMS (+ESI) for $(M+H)^+$ $C_{15}H_{15}ClN^+$ (*m/z*): calc. 244.0888; found 244.0885.

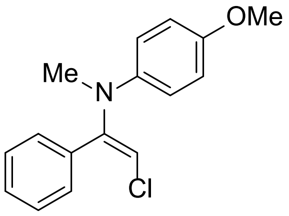<br>**6ab**	**(*E*)-*N*-(2-chloro-1-phenylvinyl)-4-methoxy-*N*-methylaniline.** Yield 68 %. Enamine *E* 68 %, α-chloroketone: 13 %, chloroalkyne 19 %.<br>Enamine *E*: ^{1}H NMR (500 MHz, $CDCl_3$) δ 7.34-7.32 (m, 2H), 7.27-7.24 (m, 3H), 6.69 (d, *J* =9.2 Hz, 2H), 6.62 (d, *J* = 9.2 Hz, 2H), 6.10 (s, 1H), 3.64 (s, 3H), 3.12 (s, 3H); ^{13}C{^{1}H} NMR (125 MHz, $CDCl_3$) δ 152.4, 146.4, 141.1, 136.9, 132.0, 128.8, 127.0, 115.6, 114.5, 111.1, 55.6, 38.3; HRMS (+ESI) for $(M+H)^+$ $C_{16}H_{17}ClNO^+$ (m/z): calc. 274.0999; found 274.0996.
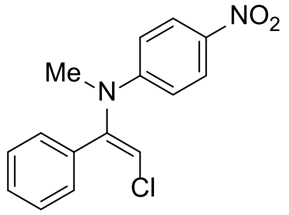<br>**6ac**	**(*E*)-*N*-(2-chloro-1-phenylvinyl)-*N*-methyl-4-nitroaniline**. Yield 87 %. Enamine *E* 87 %, α-chloroketone: 13 %.<br>Enamine *E*: ^{1}H NMR (500 MHz, $CDCl_3$) δ 8.00-7.97 (m, 2H), 7.27-7.21 (m, 5H), 6.62-6.59 (m, 2H), 6.70-6.68 (m, 3H), 6.51 (s, 1H), 3.16 (s, 3H); ^{13}C{^{1}H} NMR (125 MHz, $CDCl_3$) δ 152.0,144.8,138.9, 134.5, 129.7, 129.4, 126.5, 126.1, 116.0, 112.2, 38.0; HRMS (+ESI) for $(M+H)^+$ $C_{15}H_{14}ClN_2O_2^+$ (m/z): calc. 289.0744; found 289.0742.
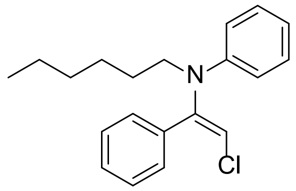<br>**6ad**	**(*E*)-*N*-(2-chloro-1-phenylvinyl)-*N*-hexylaniline.**<br>Yield 90 %. Enamine *E* 90 %, α-chloroketone 10 %.<br>Enamine *E*: ^{1}H NMR (400 MHz, $CDCl_3$) δ 7.39-7.33 (m, 2H), 7.28-7.24 (m, 4H), 7.17 (t, *J* = 7.8 Hz, 2H), 6.77 (d, *J* = 8.3 Hz, 2H), 6.26 (s, 1H), 3.37 (t, *J* = 7.8 Hz, 2H), 1.66-1.62 (m, 2H), 1.34-1.17 (m, 6H), 0.84-0.82 (m, 3H); ^{13}C{^{1}H} NMR (75 MHz, $CDCl_3$) δ 146.7, 136.8, 132.1, 129.2, 128.9, 128.8, 127.1, 118.1, 114.2, 113.3, 50.1, 31.7, 28.4, 26.8, 22.7, 14.1; HRMS (+ESI) for $(M+H)^+$ $C_{20}H_{25}ClN^+$ (m/z): calc. 314.1670; 314.1665.
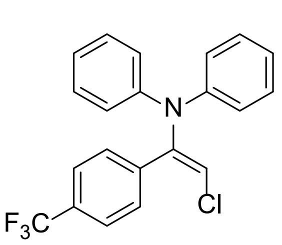<br>**6de**	**(*E*)-*N*-(2-chloro-1-(4-(trifluoromethyl)phenyl)vinyl)-*N*-phenylaniline.**<br>Yield 96 %. Enamine *E* 74 %, Enamine *Z* 22 %, α-chloroketone 4 %.<br>Enamine *E*: ^{1}H NMR (500 MHz, $CDCl_3$) δ 7.51-7.44 (m, 4H), 7.21-7.14 (m, 2H), 7.04 (d, *J* = 8.0 Hz, 4H), 7.00 (d, *J* = 8.0 Hz, 4H), 6.92-6.85 (m, 2H), 6,32 (s, 1H);^{19}F NMR (470 MHz, $CDCl_3$) δ -62.6; ^{13}C{^{1}H} NMR (75 MHz, $CDCl_3$) δ 145.2, 143.2, 131.2, 130.6 (q, *J* = 33 Hz), 129.5, 129.3, 127.5, 127.0,

	125.8 (q, J = 4 Hz), 124.2 (q, J = 235 Hz), 122.8, 122.0, 121.1, 117.9, 115.5; HRMS (+ESI) for $(M+H)^+$ $C_{21}H_{16}ClF_3N^+$ (m/z): calc. 374.0923; found 374.0923.
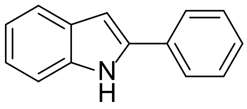 **7aa**	**2-Phenyl-1-*H*_indole.**[3] Eluent *n*-hexane:AcOEt 50:1. 154 mg as a white solid. Isolated yield 80 %. ^{1}H NMR (300 MHz, $CDCl_3$) δ 8.32 (s, 1H), 7.66-7.60 (m, 3H), 7.46-7.37 (m, 3H), 7.31 (t, J = 7.5 Hz, 1H), 7.21-7.08 (m, 2H), 6.81 (bs, 1H); ^{13}C{^{1}H} NMR (75 MHz, $CDCl_3$) δ 138.1, 137.0, 130.2, 129.5, 129.3, 127.9, 125.4, 122.6, 120.9, 120.5, 111.1, 100.2.
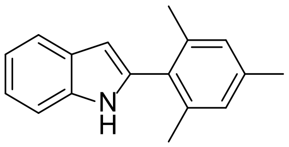 **7ba**	**2-Mesityl-1-*H*-indole.**[4] Eluent *n*-hexane:AcOEt 50:1. 167 mg as a white solid. Isolated yield 71 % ^{1}H NMR (500 MHz, $CDCl_3$) δ 7.85 (s, 1H), 7.69-7.59 (m, 1H), 7.40-7.34 (m, 1H), 7.20-7.15 (m, 1H), 7.12 (td, J = 7.5, 1.1 Hz, 1H), 6.95 (s, 2H), 6.37 (d, J = 2.1 Hz, 1H), 2.33 (s, 3H), 2.13 (s, 6H); ^{13}C{^{1}H} NMR (126 MHz, $CDCl_3$) δ 138.4, 138.3, 136.4, 136.0, 130.2, 129.0, 128.3, 121.5, 120.4, 119.9, 110.8, 102.8, 21.3, 20.6.
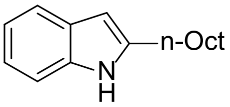 **7ea**	**2-Octyl-1-*H*-indole.**[3] Eluent *n*-hexane:AcOEt 50:1. 156 mg as a thick colorless oil. Isolated yield 68 %. ^{1}H NMR (500 MHz, $CDCl_3$) δ 7.81 (s, 1H), 7.51 (d, J = 7.7 Hz, 1H), 7.27 (d, J = 7.7 Hz, 1H), 7.11-7.03 (m, 2H), 6.22 (bs, 1H), 2.72 (t, J = 7.6 Hz, 2H), 1.70 (p, J = 7.6 Hz, 2H), 1.38-1.25 (m, 12H), 0.87 (t, J = 6.8 Hz, 3H); ^{13}C{^{1}H} NMR (126 MHz, $CDCl_3$) δ 140.2, 136.0, 129.1, 121.1, 119.9, 119.8, 110.5, 99.7, 32.1, 29.6, 29.6, 29.4, 29.4, 28.5, 22.9, 14.3.
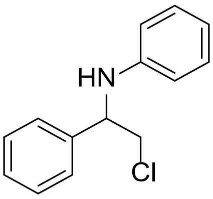 **8aa**	***N*-(2-chloro-1-phenylethyl)aniline.**[5] Eluent *n*-hexane:AcOEt 50:1. 146 mg as a colourless oil. Isolated yield 63 %. ^{1}H NMR (500 MHz, $CDCl_3$) δ 7.43 (dd, J = 7.5, 2.1 Hz, 2H), 7.38 (td, J = 7.5, 2.2 Hz, 2H), 7.35-7.28 (m, 1H), 7.14 (td, J = 8.0, 2.9 Hz, 2H), 6.73 (td, J = 7.5, 3.0 Hz, 1H), 6.58 (dd, J = 8.3, 2.8 Hz, 2H), 4.64 (dt, J = 7.4, 3.7 Hz, 1H), 4.46 (bs, 1H), 3.89 (ddd, J = 11.4, 3.7, 1.8 Hz, 1H), 3.74 (ddd, J = 11.4, 7.5,

	1.8 Hz, 1H); $^{13}C\{^1H\}$ NMR (126 MHz, $CDCl_3$) δ 146.9, 140.1, 129.3, 129.0, 128.2, 126.9, 118.4, 114.0, 59.5, 49.2.
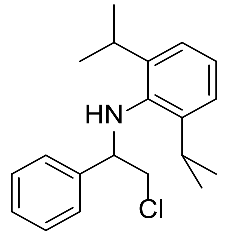 **8ab**	***N*-(2-chloro-1-phenylethyl)-2,6-diisopropylaniline.** Eluent *n*-hexane:AcOEt 50:1. 215 mg as a colorless oil. Isolated yield 68 %.<br>^1H NMR (300 MHz, $CDCl_3$) δ 7.37-7.22 (m, 5H), 7.01-6.97 (m, 3H), 4.17 (dd, *J* = 6.6, 4.6 Hz, 1H), 3.84 (dd, *J* = 10.9, 4.6 Hz, 1H), 3.78 (dd, *J* = 10.9, 6.6 Hz, 1H), 3.49 (br s, 1H), 3.11 (m, 2H), 1.13 (d, *J* = 6.8 Hz, 6H), 1.01 (d, *J* = 6.8 Hz, 6H); $^{13}C\{^1H\}$ NMR (75 MHz, $CDCl_3$) δ 142.8, 141.0, 140.6, 128.7, 128.0, 127.2, 124.2, 123.9, 65.4, 48.2, 27.9, 24.4; HRMS (+ESI) for $(M+H)^+$ $C_{20}H_{27}ClN^+$ (*m/z*): calc. 316.1827; found 316.1818.
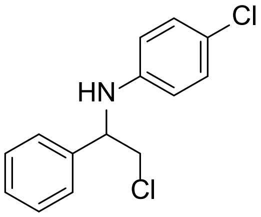 **8ad**	**4-chloro-*N*-(2-chloro-1-phenylethyl)aniline.**[5] Eluent *n*-hexane:AcOEt 50:1. 151 mg as a colorless oil. Isolated yield 57 %.<br>^1H NMR (500 MHz, $CDCl_3$) δ 7.45-7.33 (m, 4H), 7.31 (dd, *J* = 5.9, 2.9 Hz, 1H), 7.10-7.01 (m, 2H), 6.51-6.43 (m, 2H), 4.57 (dd, *J* = 7.9, 4.2 Hz, 1H), 4.47 (bs, 1H), 3.87 (dd, *J* = 11.4, 4.2 Hz, 1H), 3.69 (dd, *J* = 11.4, 7.9 Hz, 1H); $^{13}C\{^1H\}$ NMR (126 MHz, $CDCl_3$) δ 145.4, 139.5, 129.1, 129.1, 128.3, 126.8, 123.0, 115.1, 59.5, 49.1.
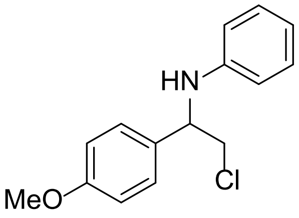 **8ca**	***N*-[2-chloro-1-(4-methoxyphenyl)ethyl]aniline.** Eluent *n*-hexane:AcOEt 50:1. 142 mg as a colorless oil. Isolated yield 55 %.<br>^1H NMR (300 MHz, $CDCl_3$) δ 7.28-7.21 (m, 2H), 7.08-7.00 (m, 2H), 6.84-6.78 (m, 2H), 6.68-6.60 (m, 1H), 6.51-6.45 (m, 2H), 4.49 (dd, *J* = 7.8, 4.5 Hz, 1H), 4.33 (s, 1H), 3.76 (dd, *J* = 11.3, 4.4 Hz, 1H), 3.73 (s, 3H), 3.62 (dd, *J* = 11.3, 7.8 Hz, 1H); $^{13}C\{^1H\}$ NMR (75 MHz, $CDCl_3$) δ 159.5, 147.0, 132.0, 129.3, 128.0, 118.3, 114.4, 114.0, 59.0, 55.4, 49.3; HRMS (+ESI) for $(M+H)^+$ $C_{15}H_{17}ClNO^+$ (*m/z*): calc. 262.0982; found 262.0975.

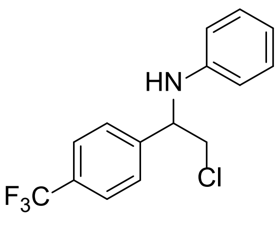<br>**8da**	***N*-[2-chloro-1-(4-(trifluoromethyl)phenyl)ethyl]aniline.** Eluent *n*-hexane:AcOEt 50:1. 186 mg as a colorless oil. Isolated yield 62 %.<br>^{1}H NMR (500 MHz, $CDCl_3$) δ 7.62 (d, *J* = 8.4 Hz, 2H), 7.51 (d, *J* = 8.4 Hz, 2H), 7.13-7.08 (m, 2H), 6.74-6.69 (m, 1H), 6.53-6.49 (m, 2H), 4.68 (dd, *J* = 7.2, 4.2 Hz, 1H), 4.46 (br s, 1H), 3.88 (dd, *J* = 11.4, 4.2 Hz, 1H), 3.71 (dd, *J* = 11.4, 7.2 Hz, 1H); ^{19}F (282 MHz, $CDCl_3$) δ -63.00 ppm; ^{13}C{^{1}H} NMR (126 MHz, $CDCl_3$) δ 146.4, 144.4, 130.7 (q, *J* = 32,5 Hz), 129.4, 127.4, 126.0 (q, *J* = 3.7 Hz), 124.1 (q, *J* = 272 Hz), 118.8, 114.0, 59.0, 48.9; HRMS (+ESI) for $(M+H)^+$ $C_{15}H_{14}ClF_3N^+$ (*m/z*): calc. 300.0761; found 300.0752**.**

9. NMR, IR and HRMS spectra. Kinetic experiments.

^{1}H (500 MHz) and $^{13}C\{^{1}H\}$ (126 MHz) NMR of **1b** in $CDCl_3$

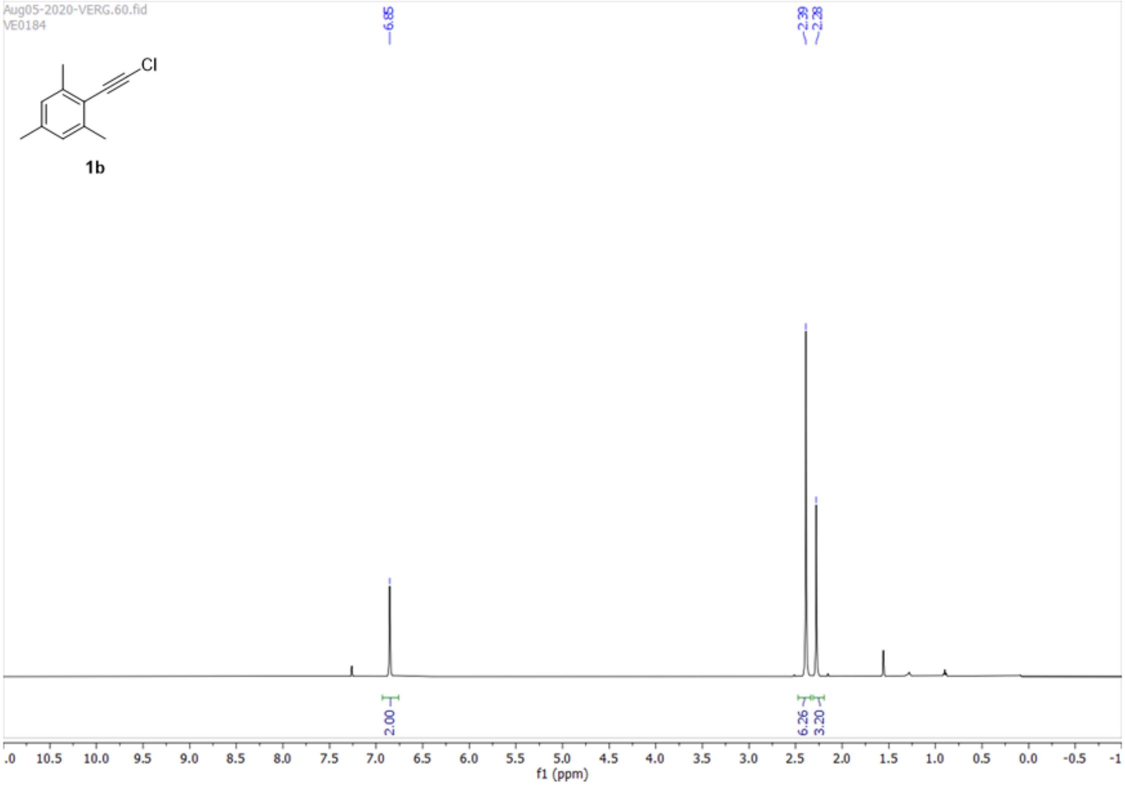

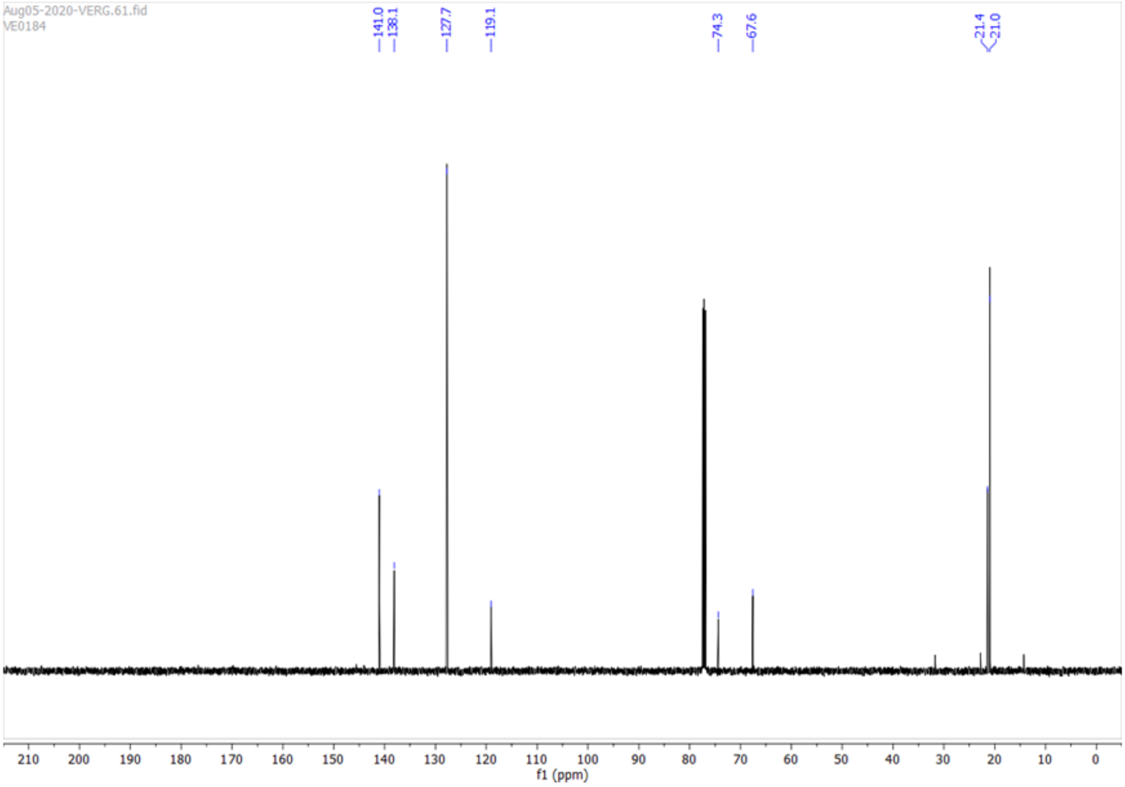

IR spectrum of **1b**:

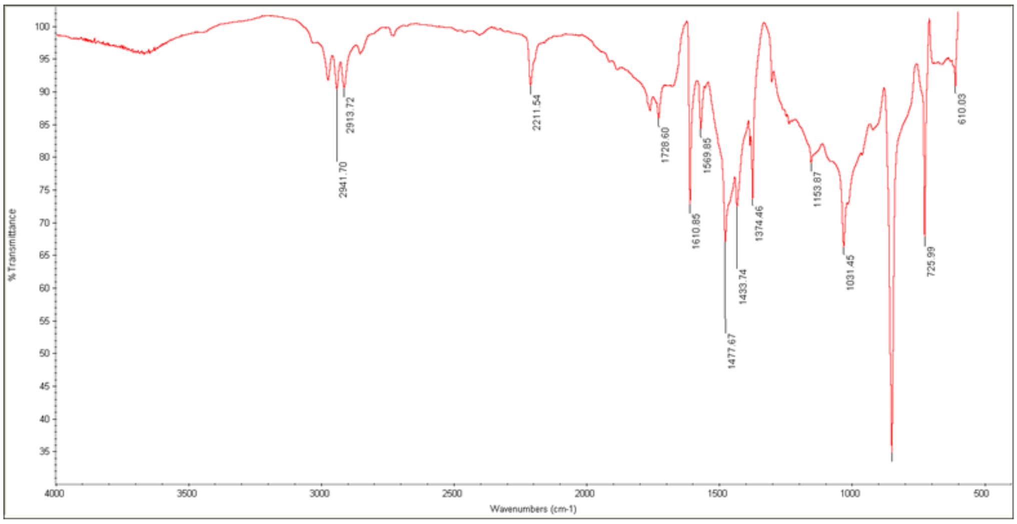

[1]H (300 MHz) and ^{13}C{^{1}H} (75 MHz) NMR of **1e** in $CDCl_3$

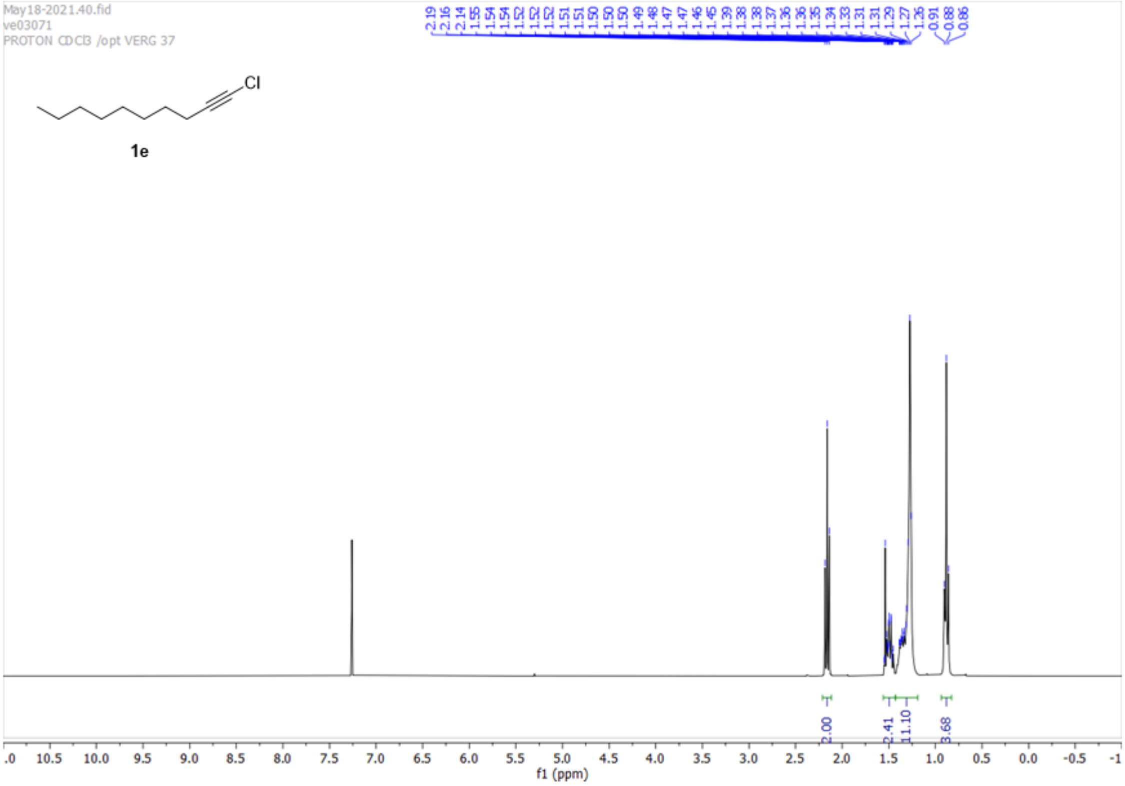

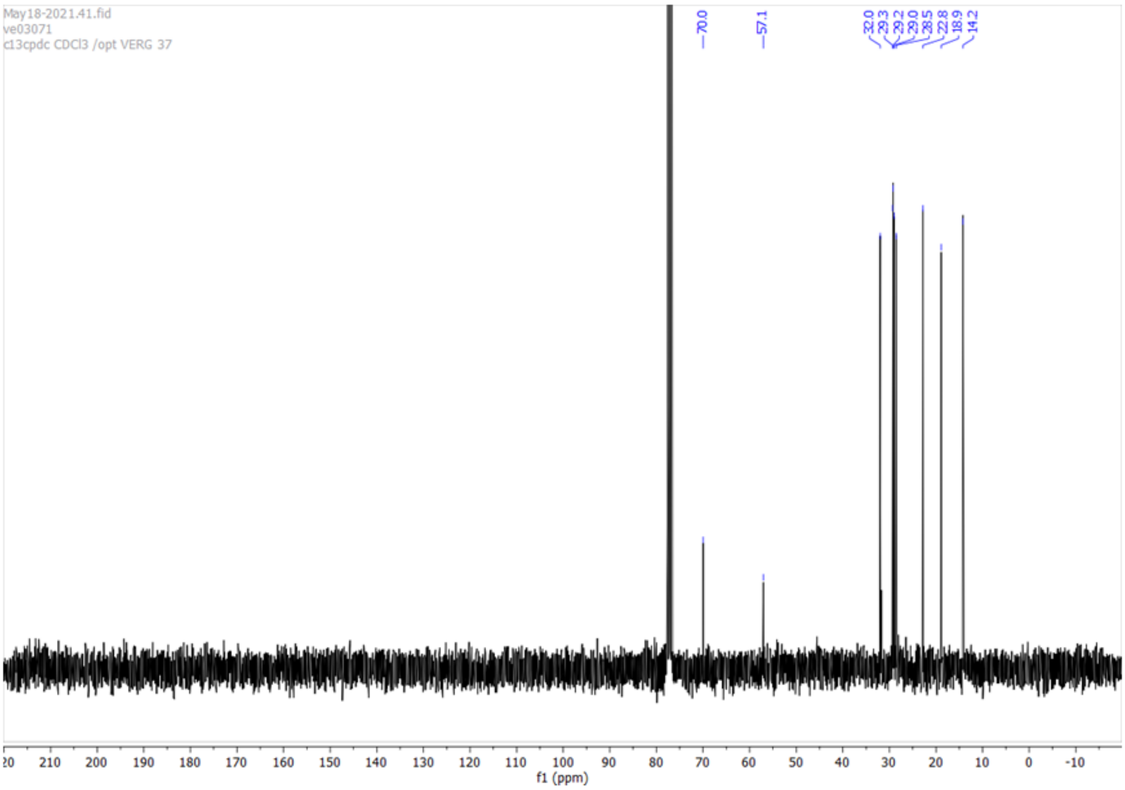

IR spectrum of **1e**:

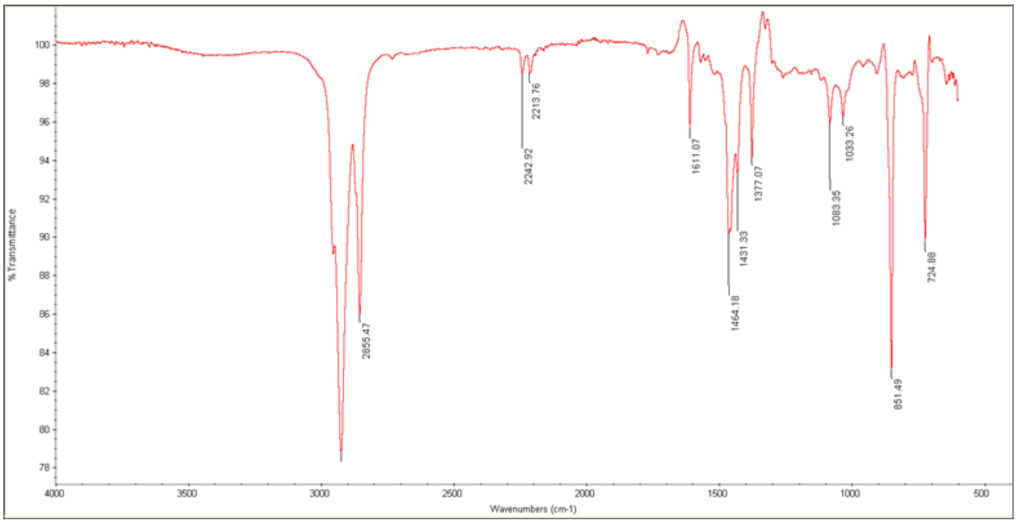

^{1}H (400 MHz) and $^{13}C\{^{1}H\}$ (100 MHz) NMR of crude **3aa** in $CDCl_3$

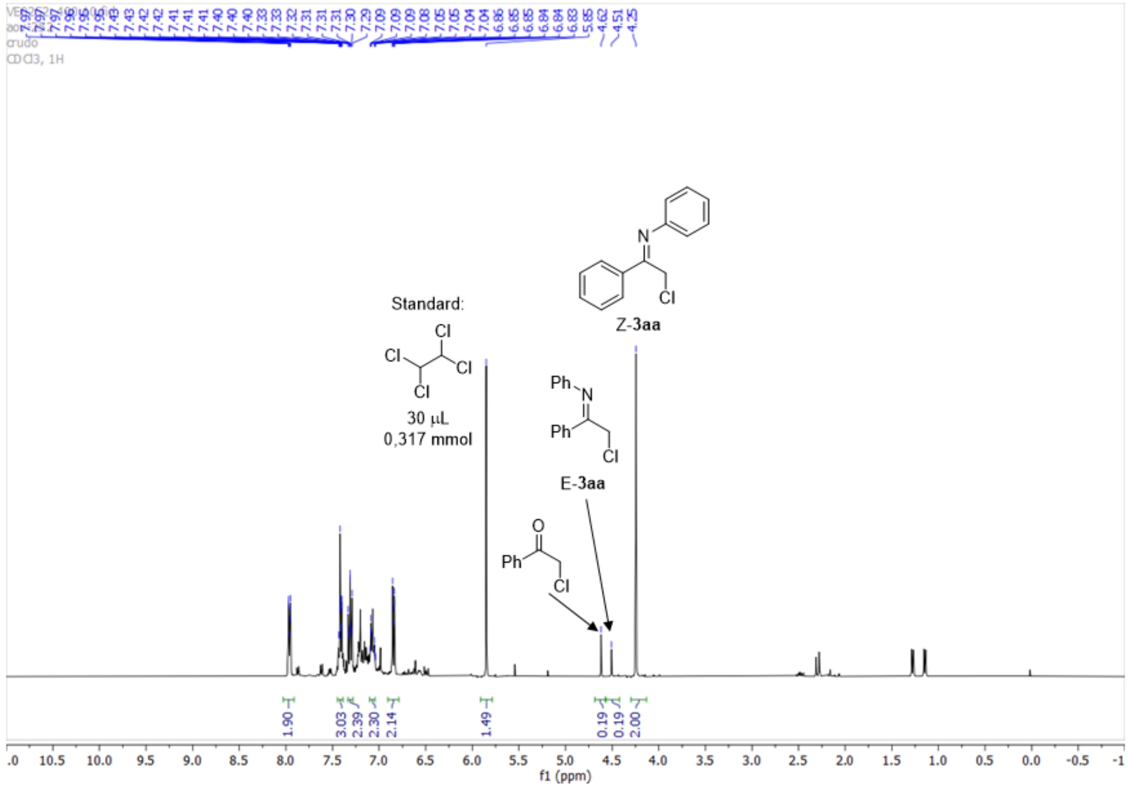

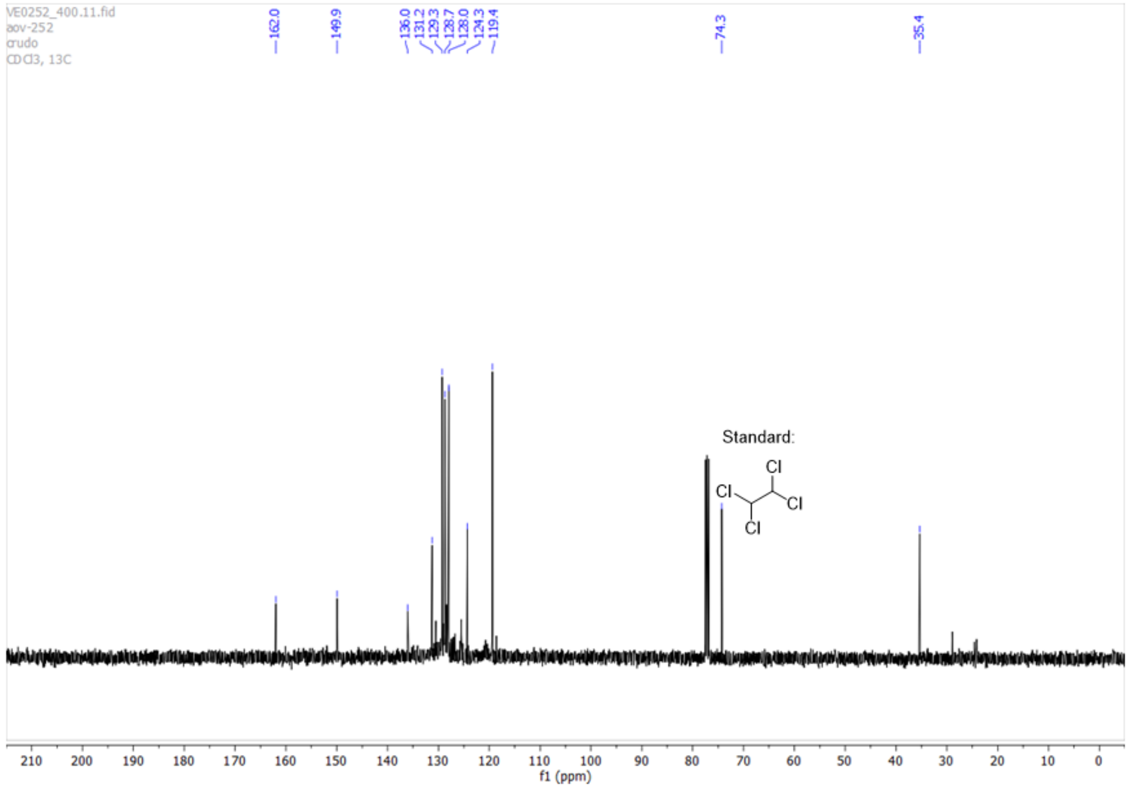

HRMS (+ESI) of crude **3aa**:

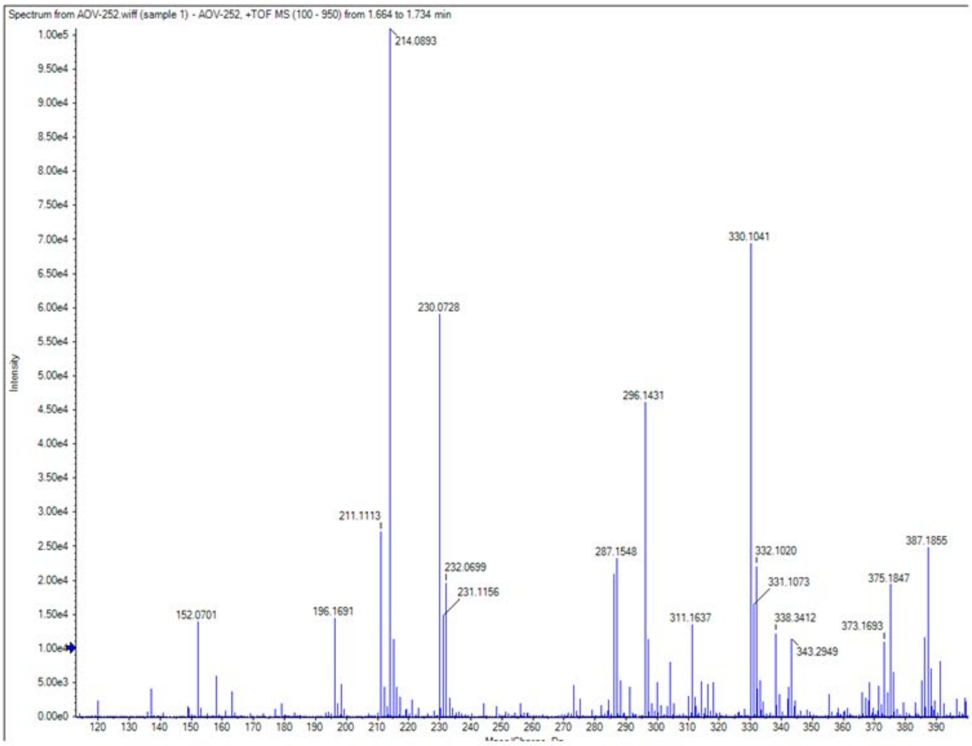

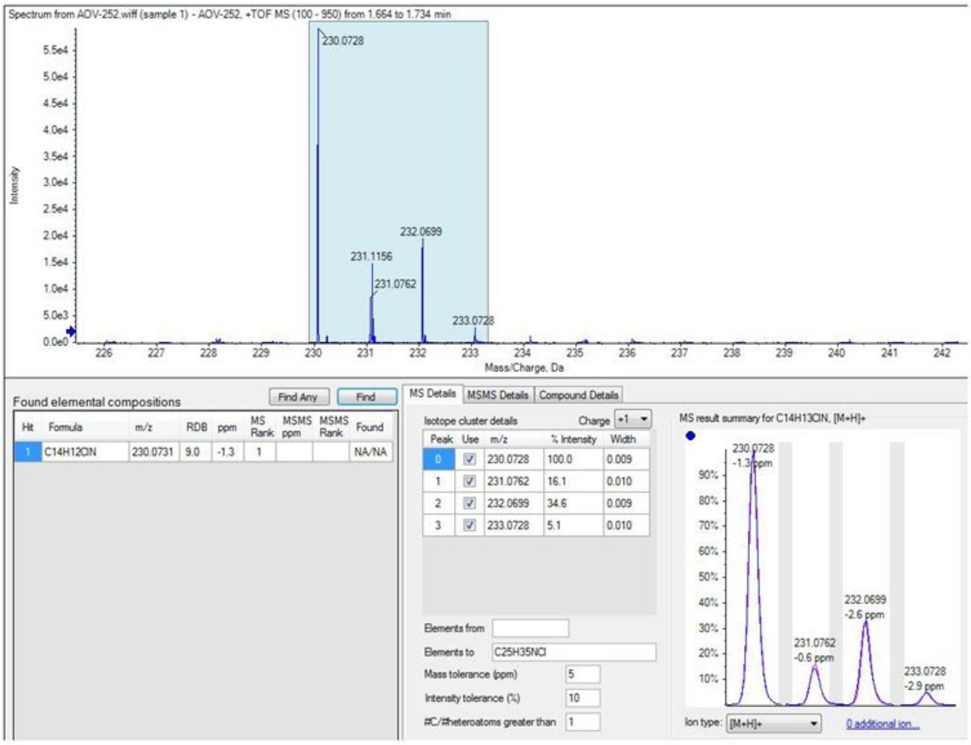

[1]H (500 MHz) and ^{13}C{^{1}H} (126 MHz) NMR of crude **3ab** in $CDCl_3$

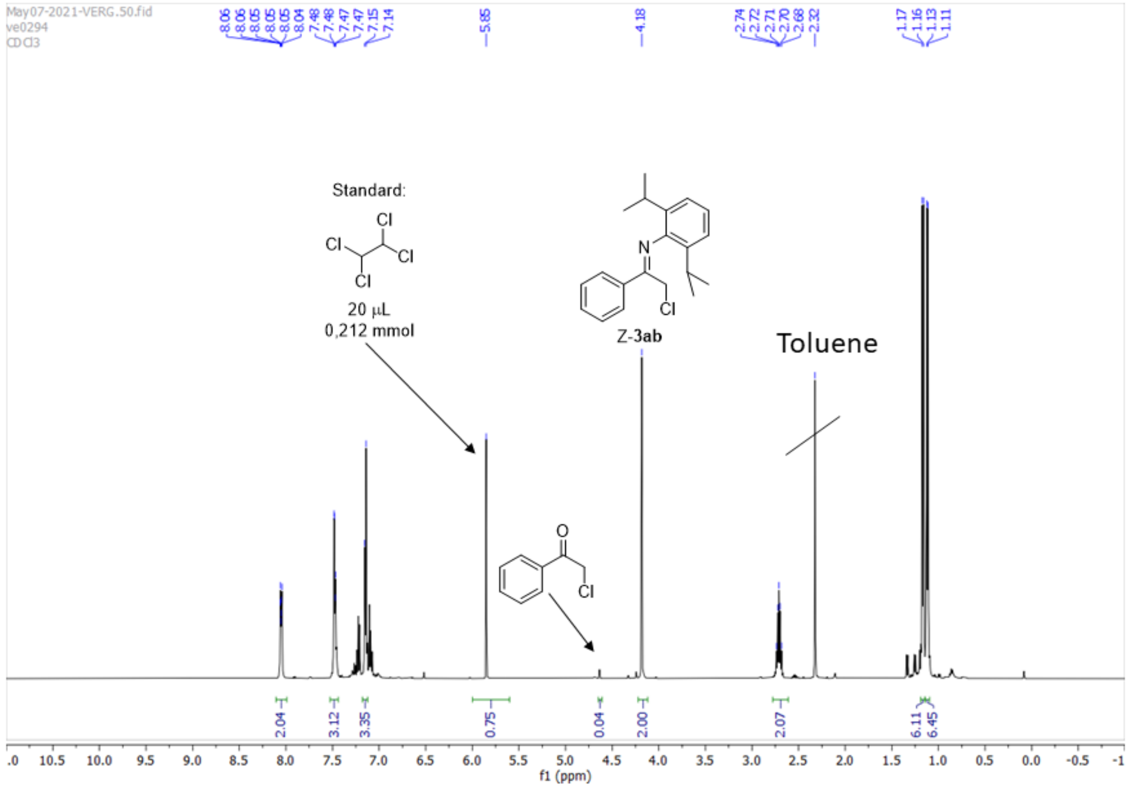

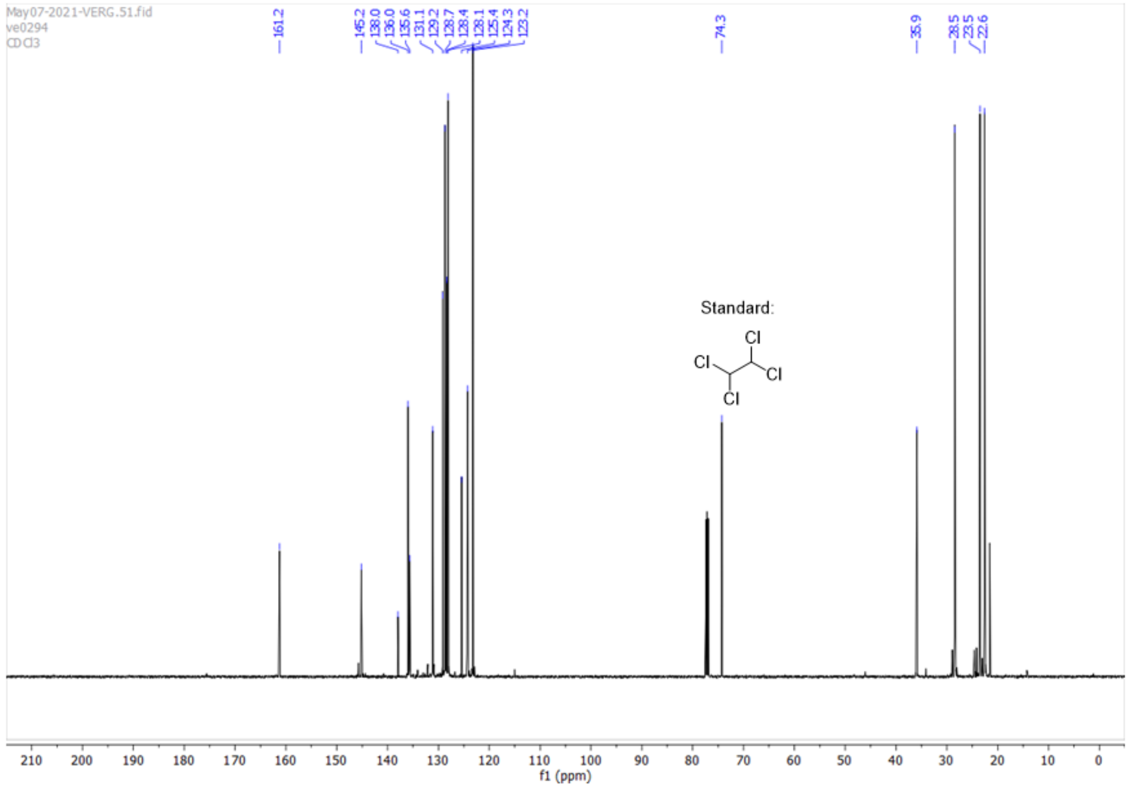

HRMS (+ESI) of crude **3ab**:

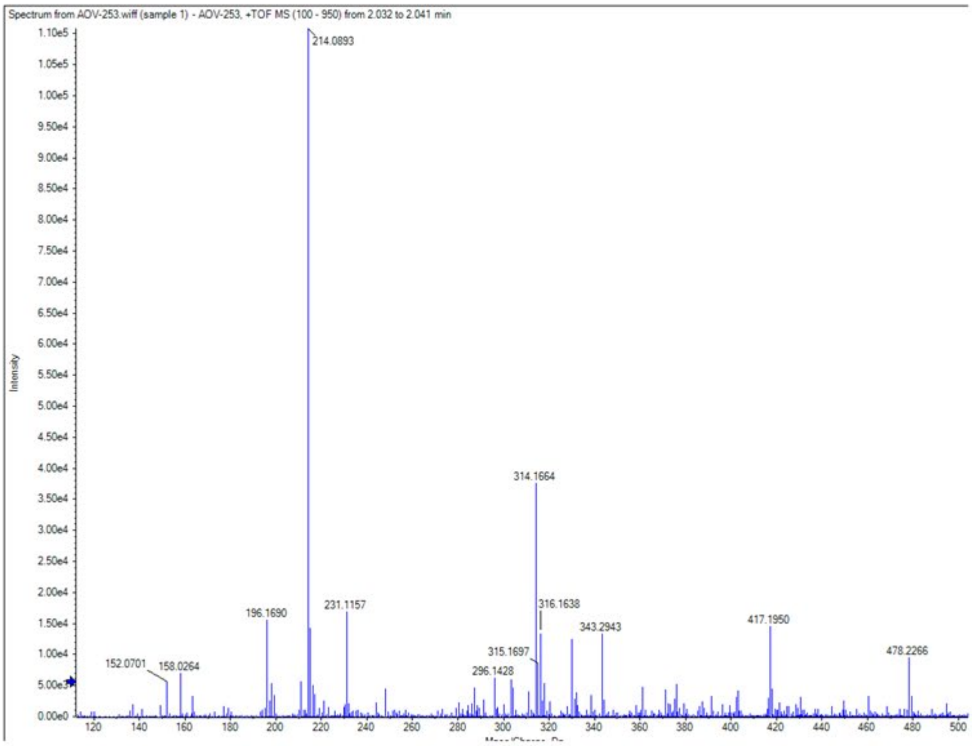

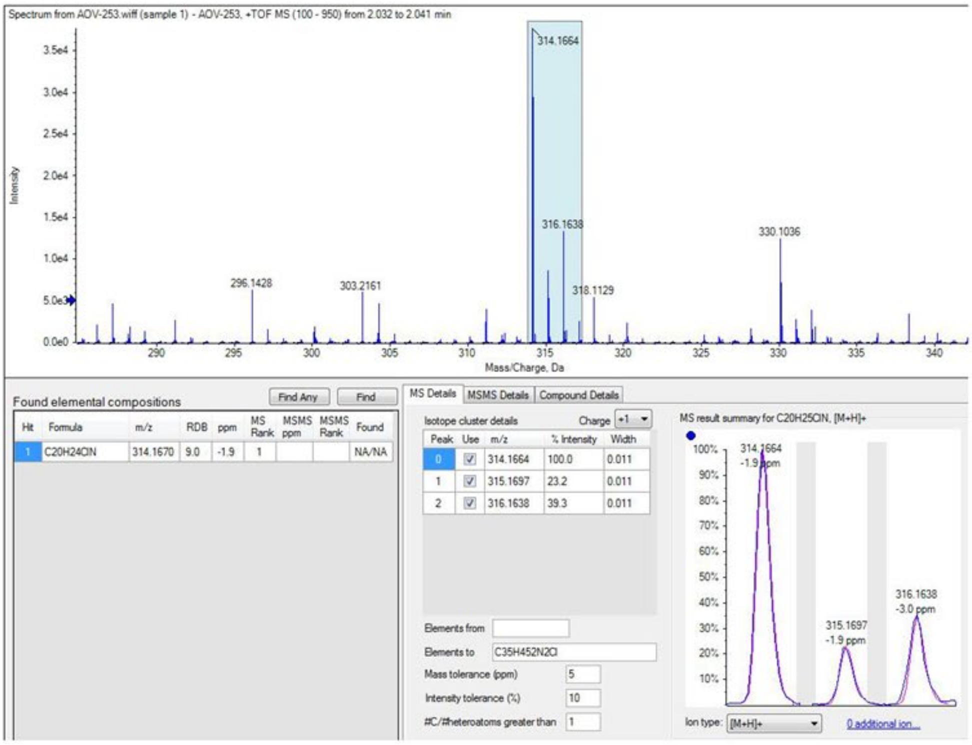

^1H (500 MHz) and $^{13}C\{^1H\}$ (125 MHz) NMR of crude **3ac** in $CDCl_3$

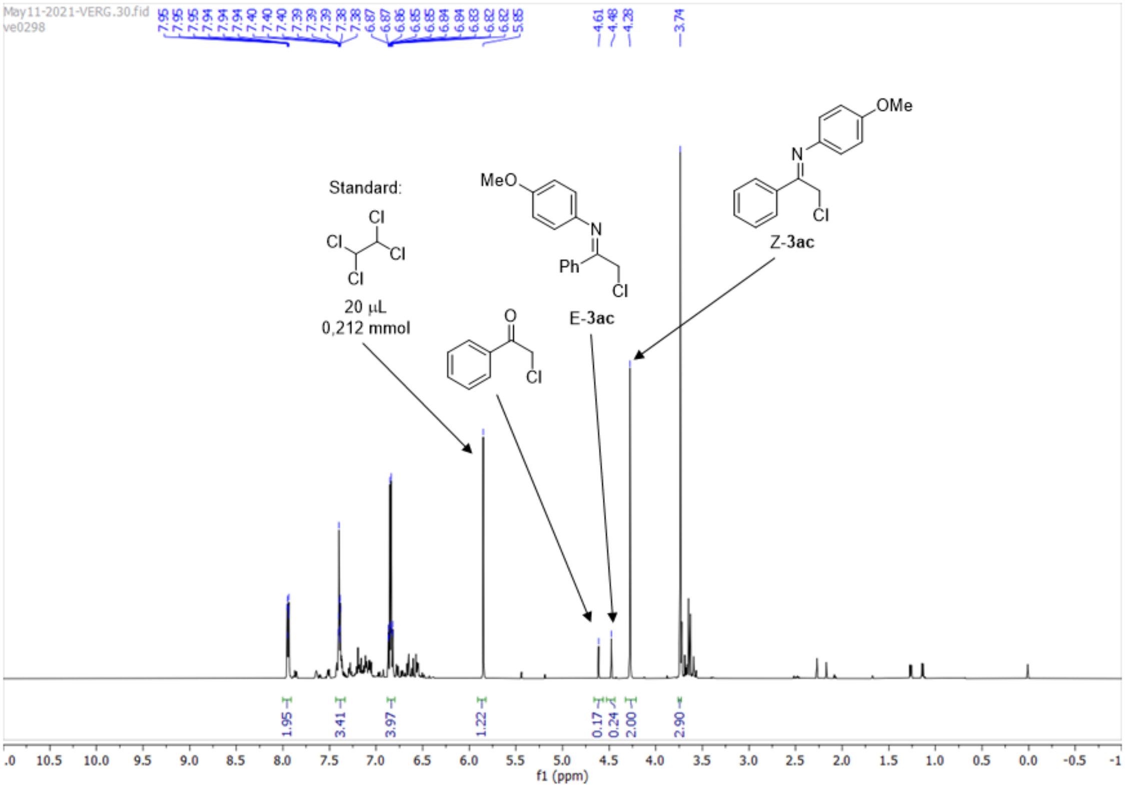

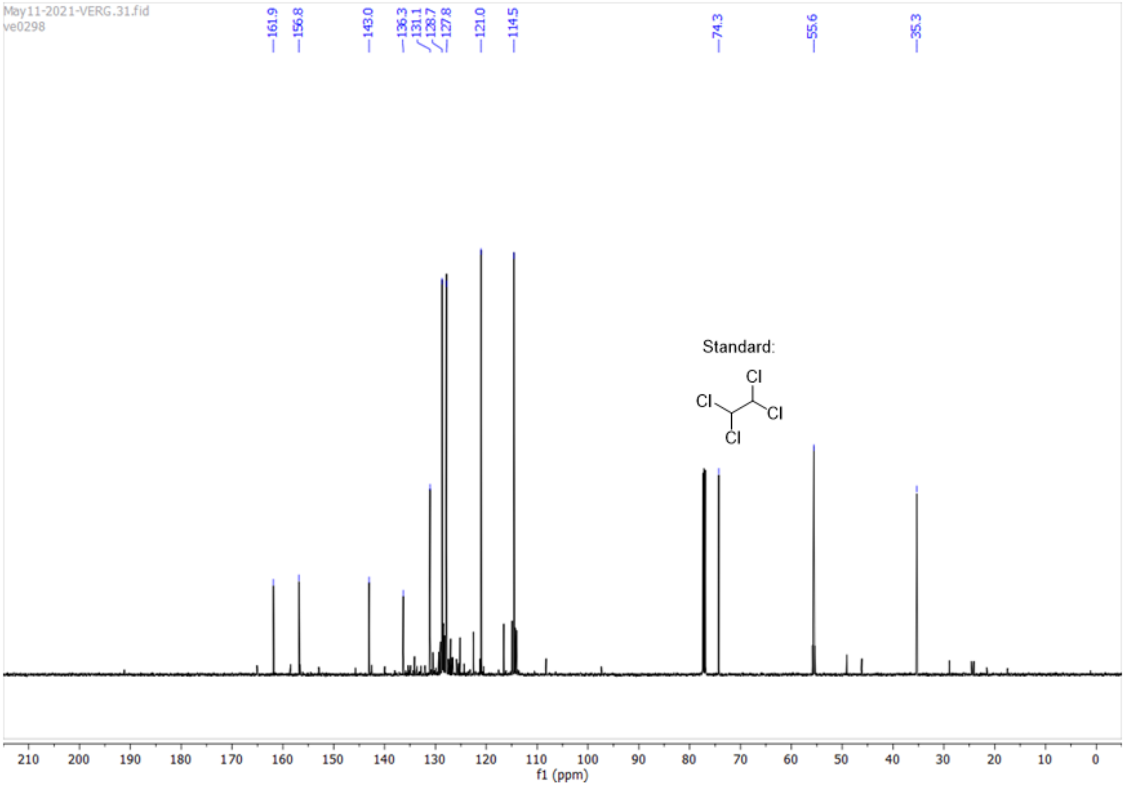

HRMS (+ESI) of crude **3ac**:

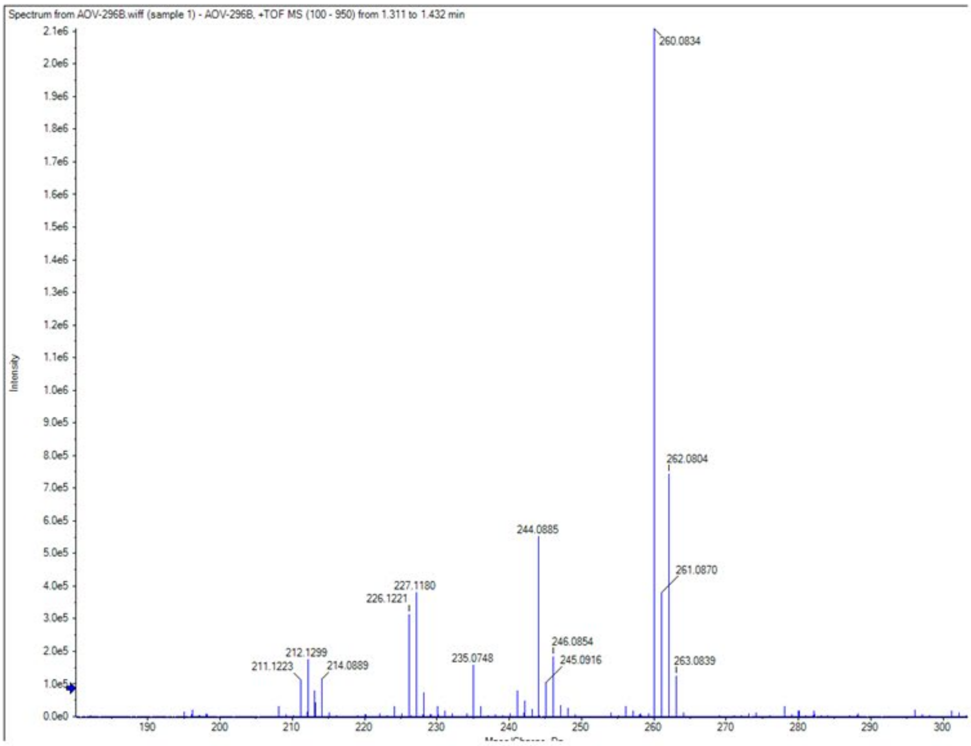

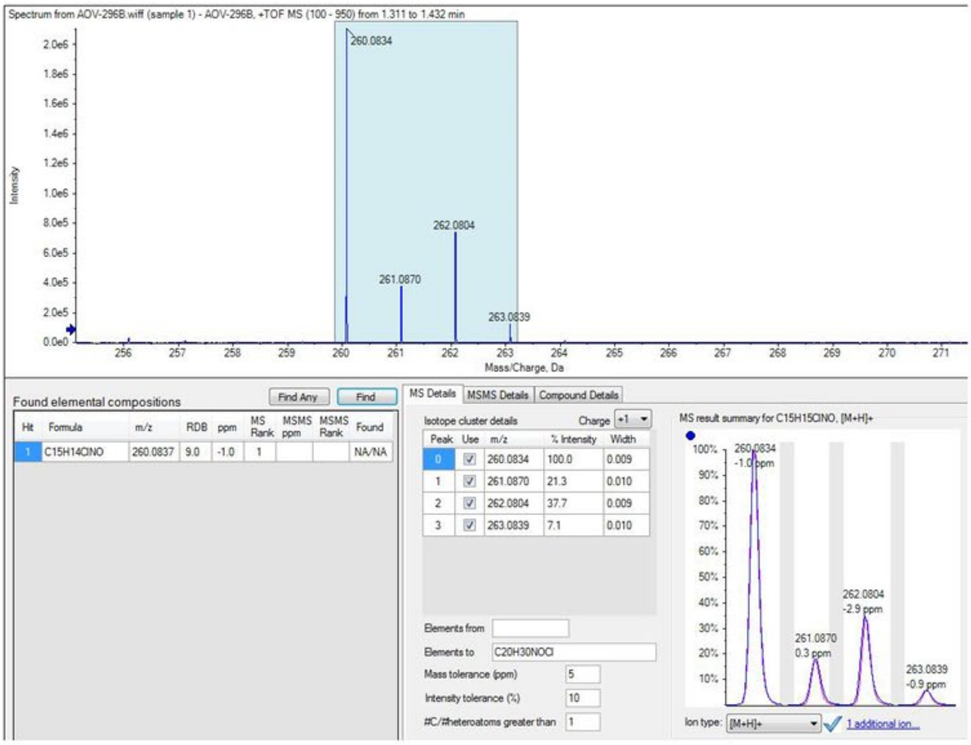

^{1}H (400 MHz) and $^{13}C\{^{1}H\}$ (100 MHz) NMR of crude **3ad** in $CDCl_3$

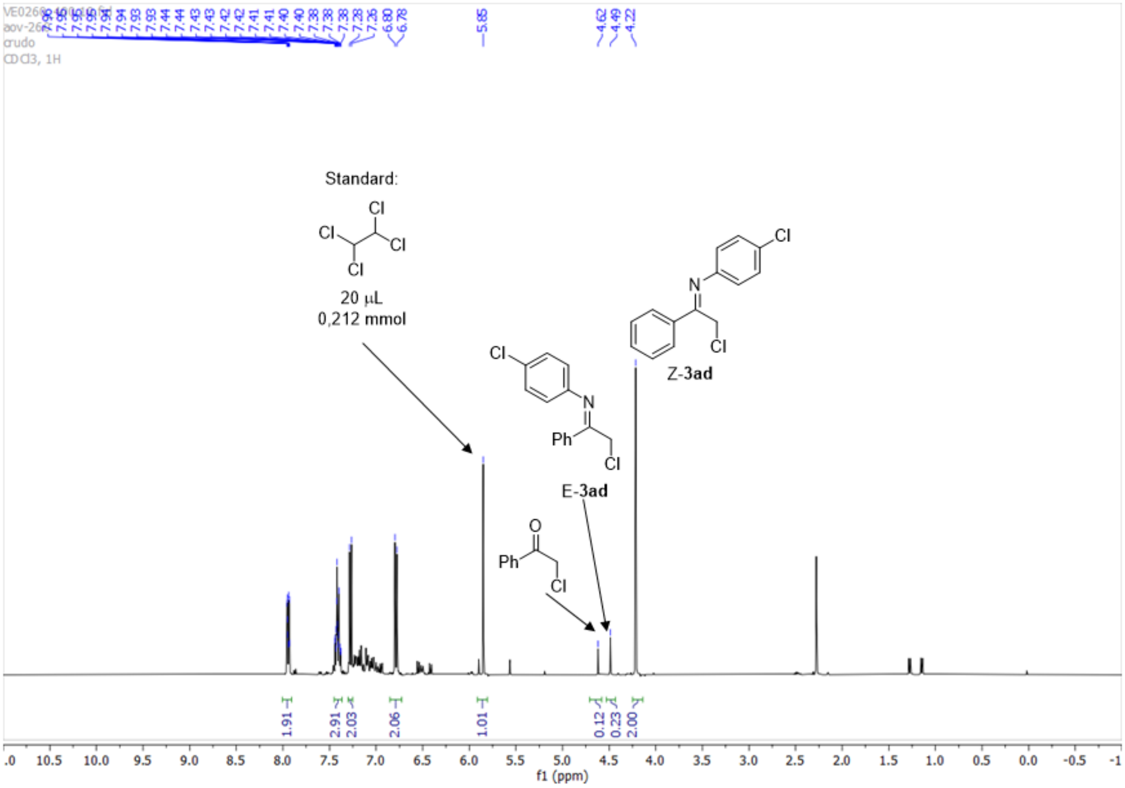

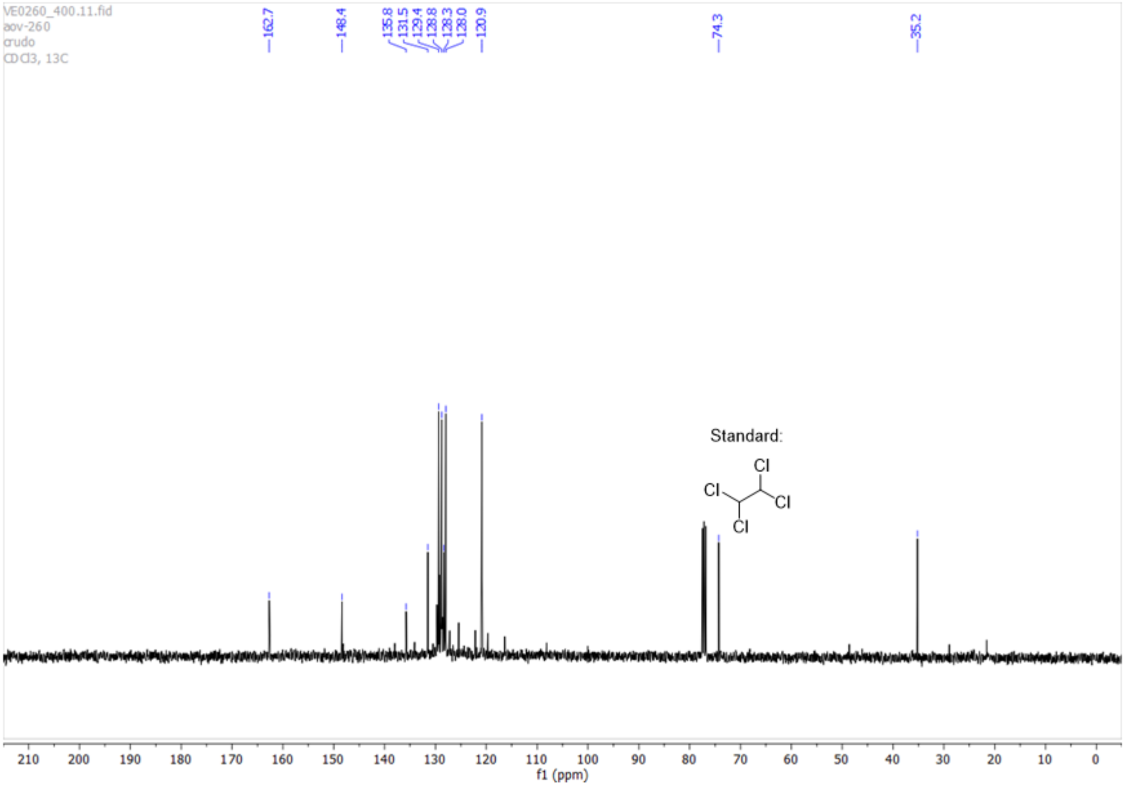

HRMS (+ESI) of crude **3ad**:

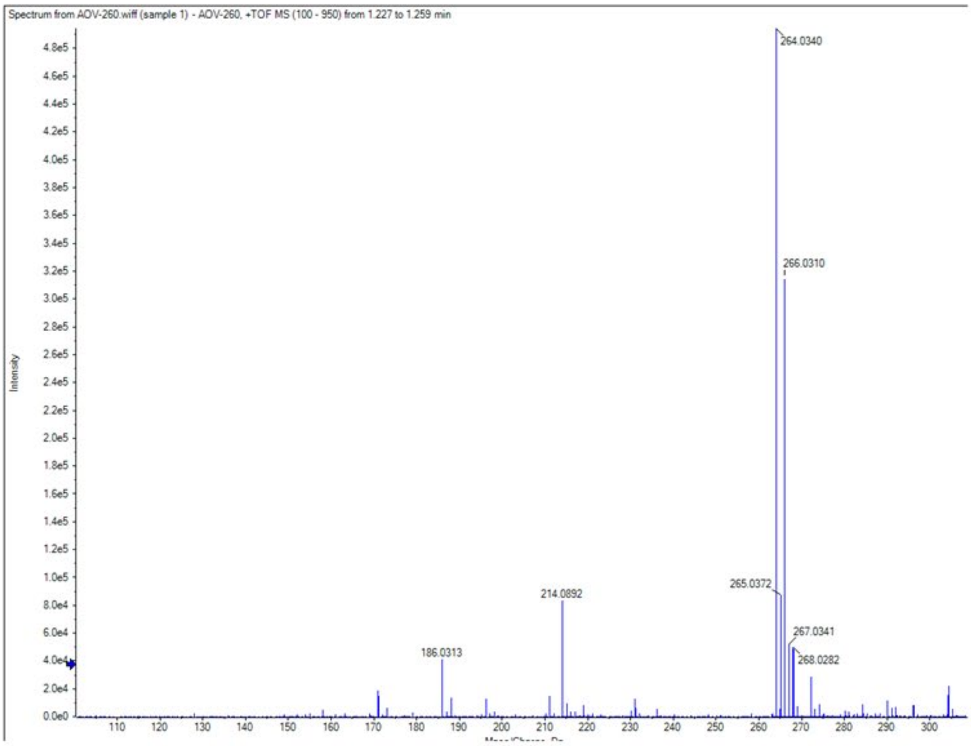

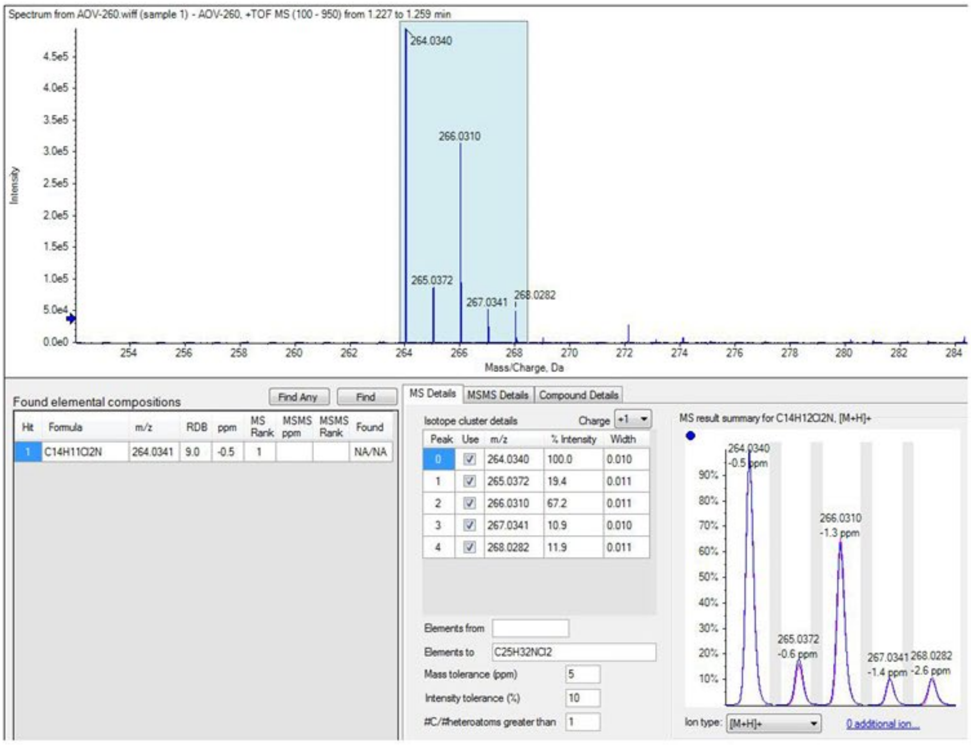

^{1}H (500 MHz) and $^{13}C\{^{1}H\}$ (125 MHz) NMR of crude **3ae** in $CDCl_3$

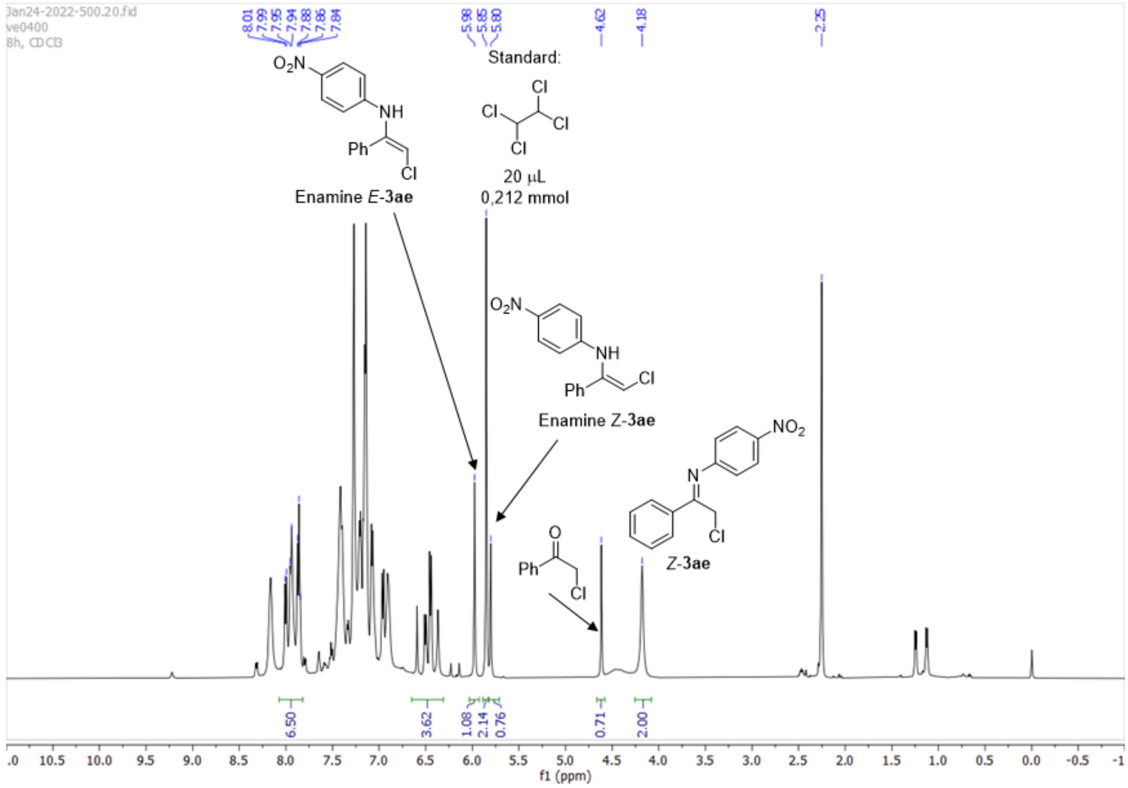

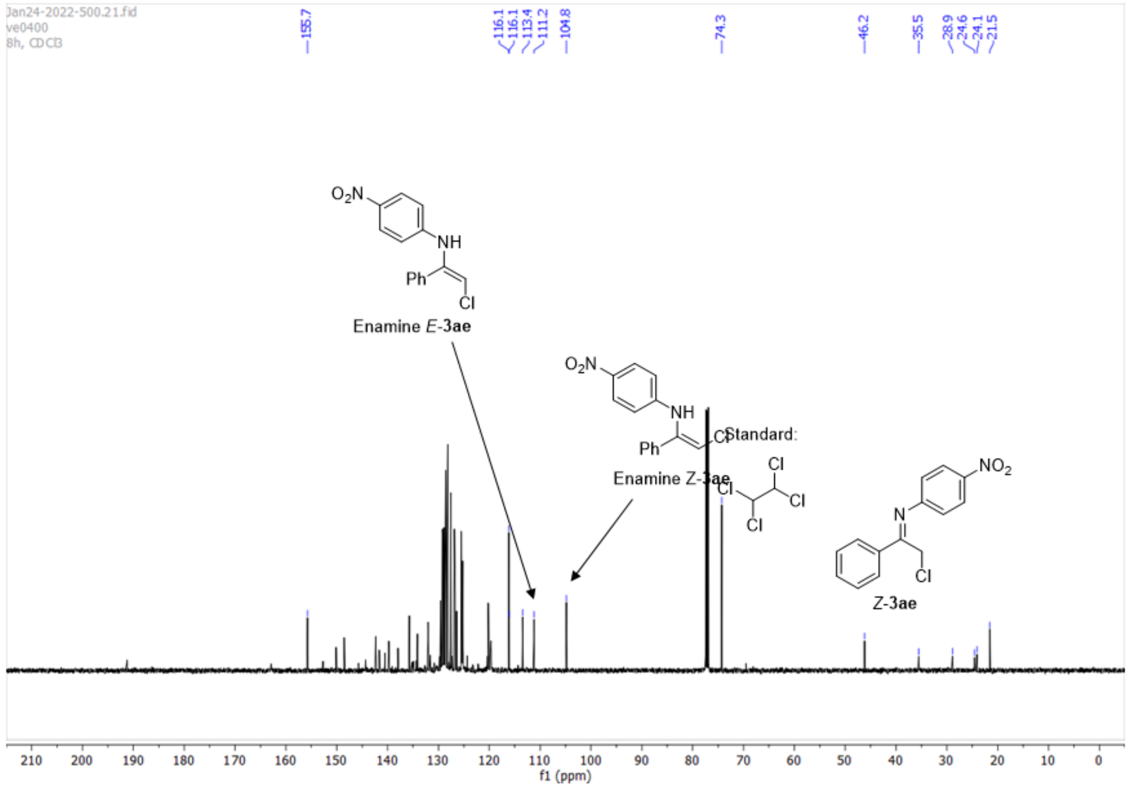

HSQC (500 MHz) of crude **3ae** in $CDCl_3$

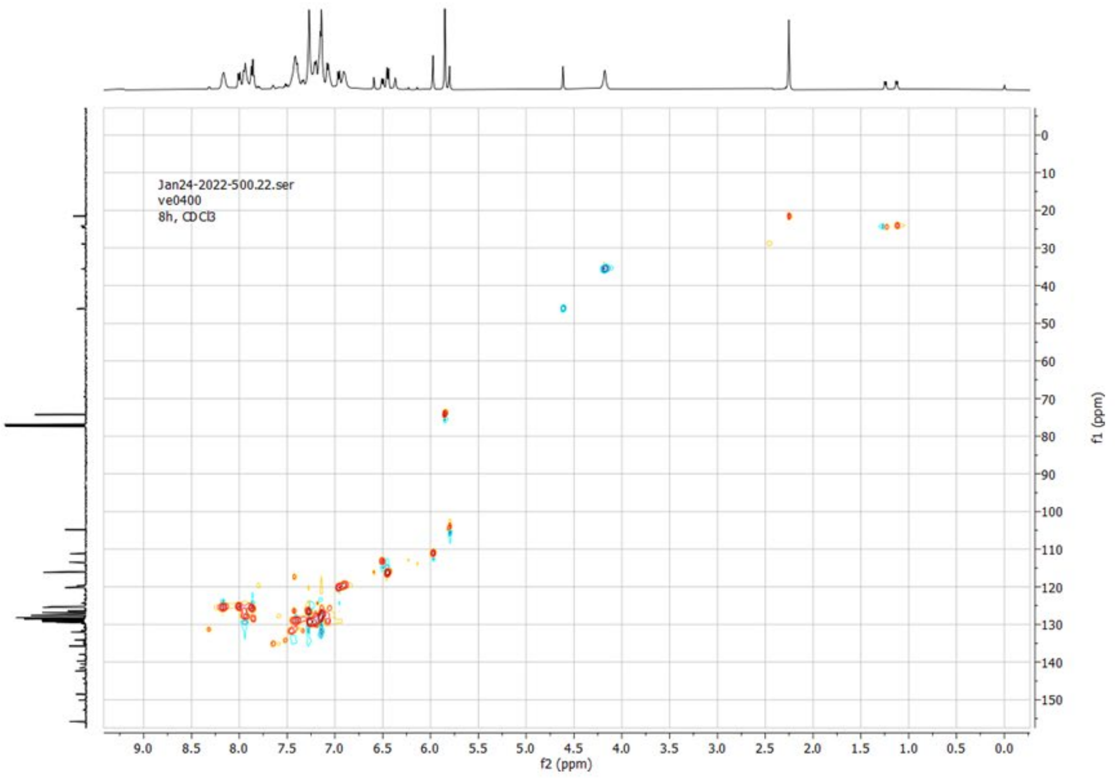

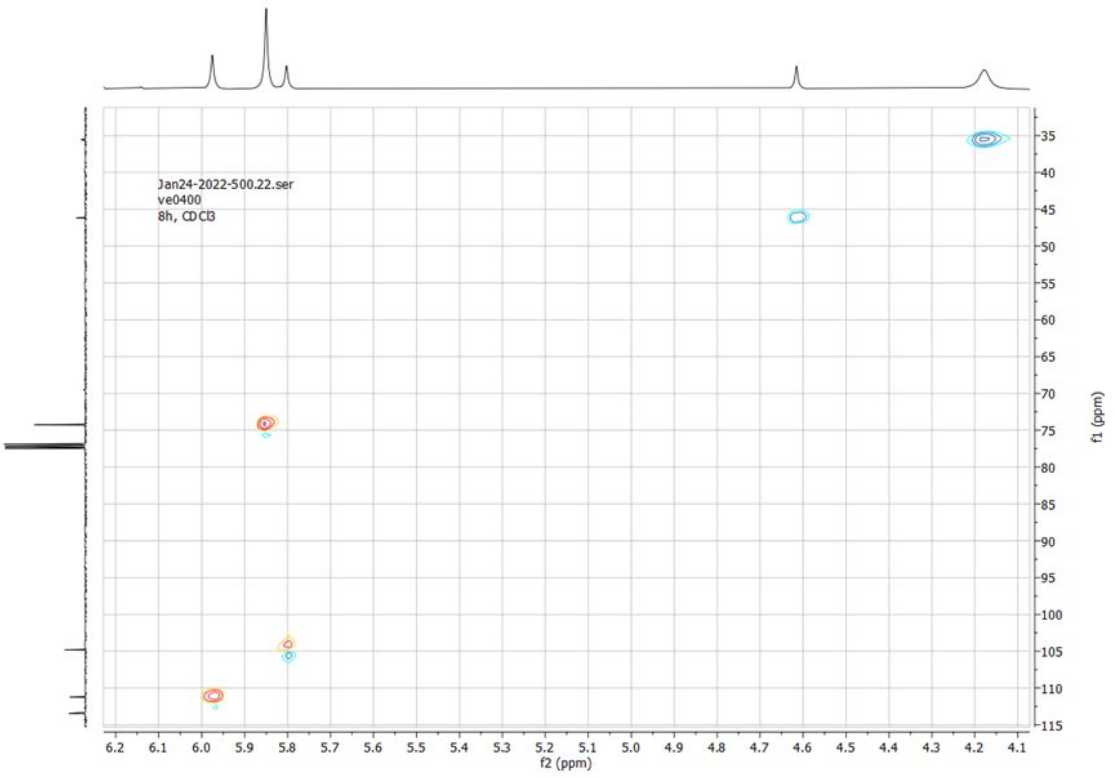

HRMS (+ESI) of crude **3ae**:

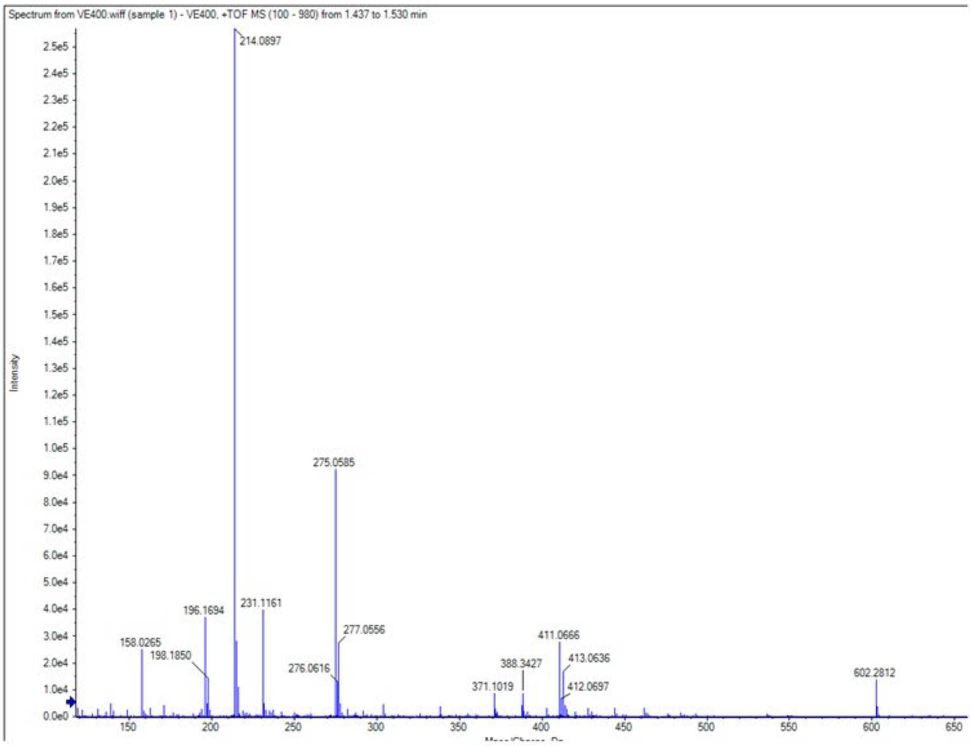

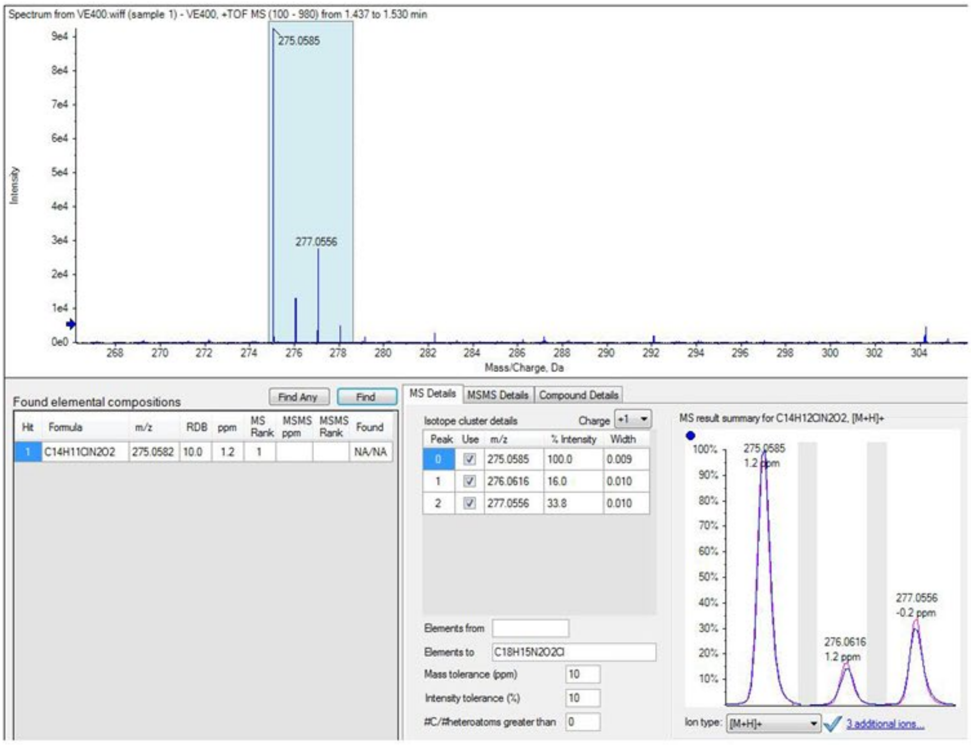

Hit	Formula	m/z	RDB	ppm	MS Rank	MSMS ppm	MSMS Rank	Found
1	C14H11ClN2O2	275.0582	10.0	1.2	1			NA/NA

Peak	Use	m/z	% Intensity	Width
0	☑	275.0585	100.0	0.009
1	☑	276.0616	16.0	0.010
2	☑	277.0556	33.8	0.010

^{1}H (400 MHz) and $^{13}C\{^{1}H\}$ (100 MHz) NMR of crude **3af** in $CDCl_3$

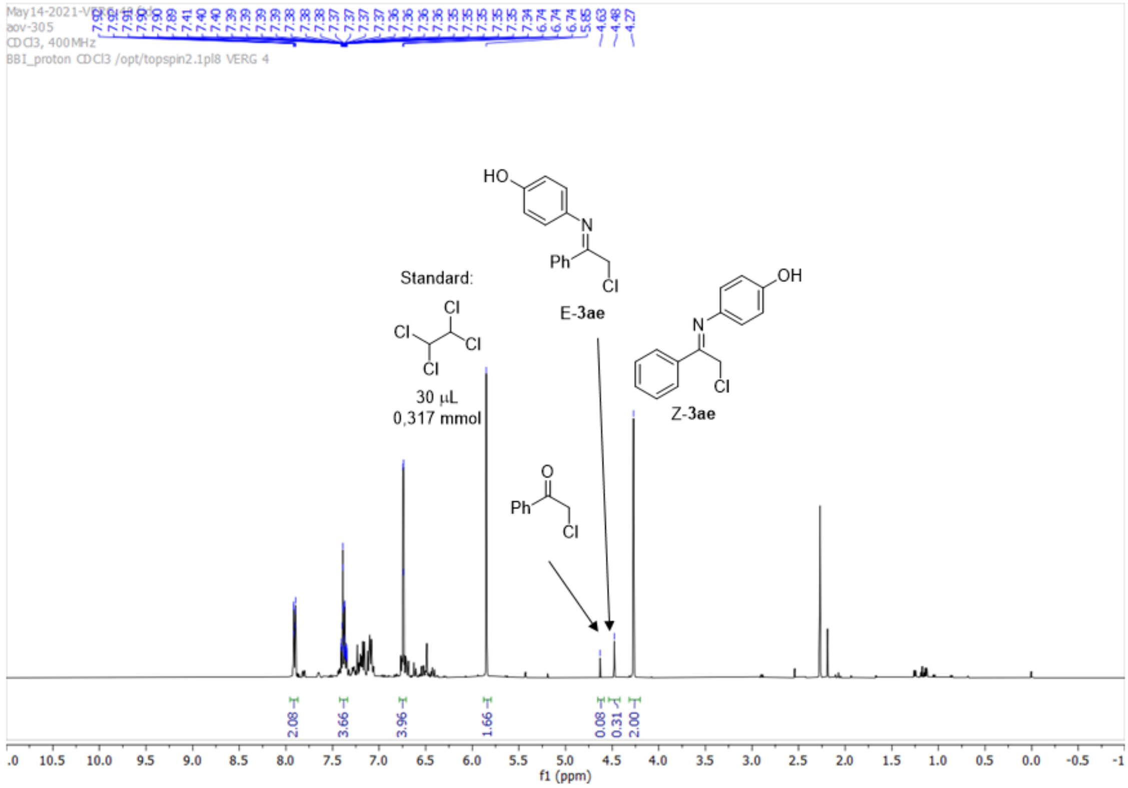

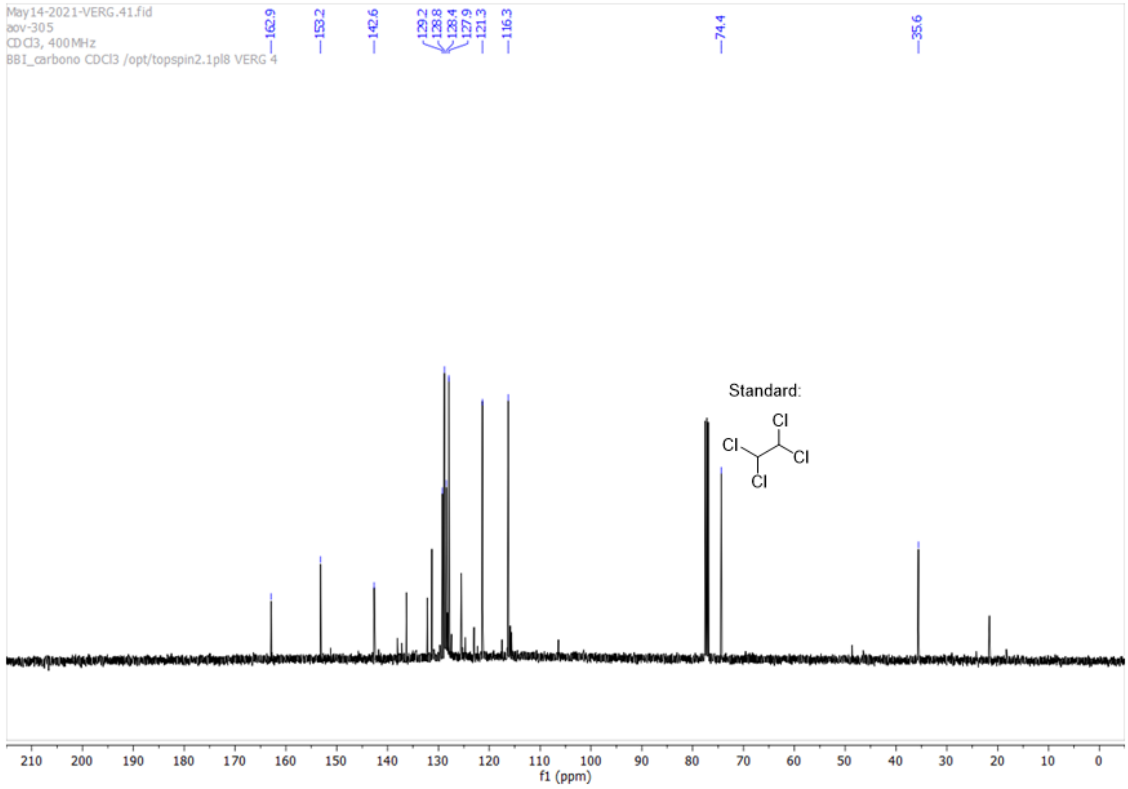

HRMS (+ESI) of crude **3af**:

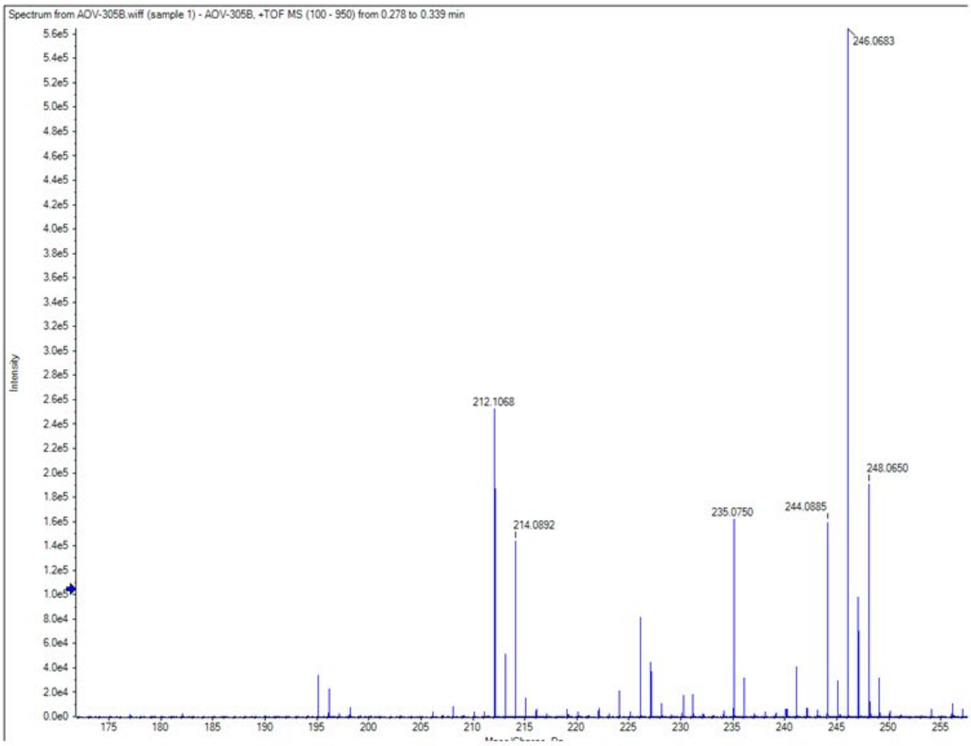

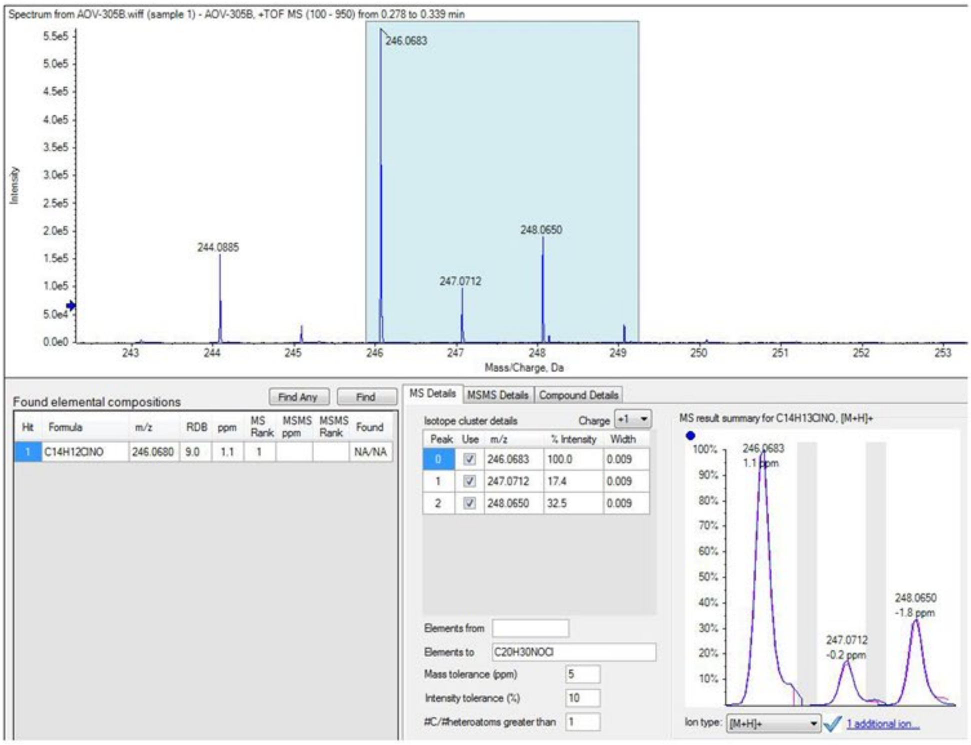

^1H (500 MHz) and $^{13}C\{^1H\}$ (125 MHz) NMR of crude **3ba** in $CDCl_3$

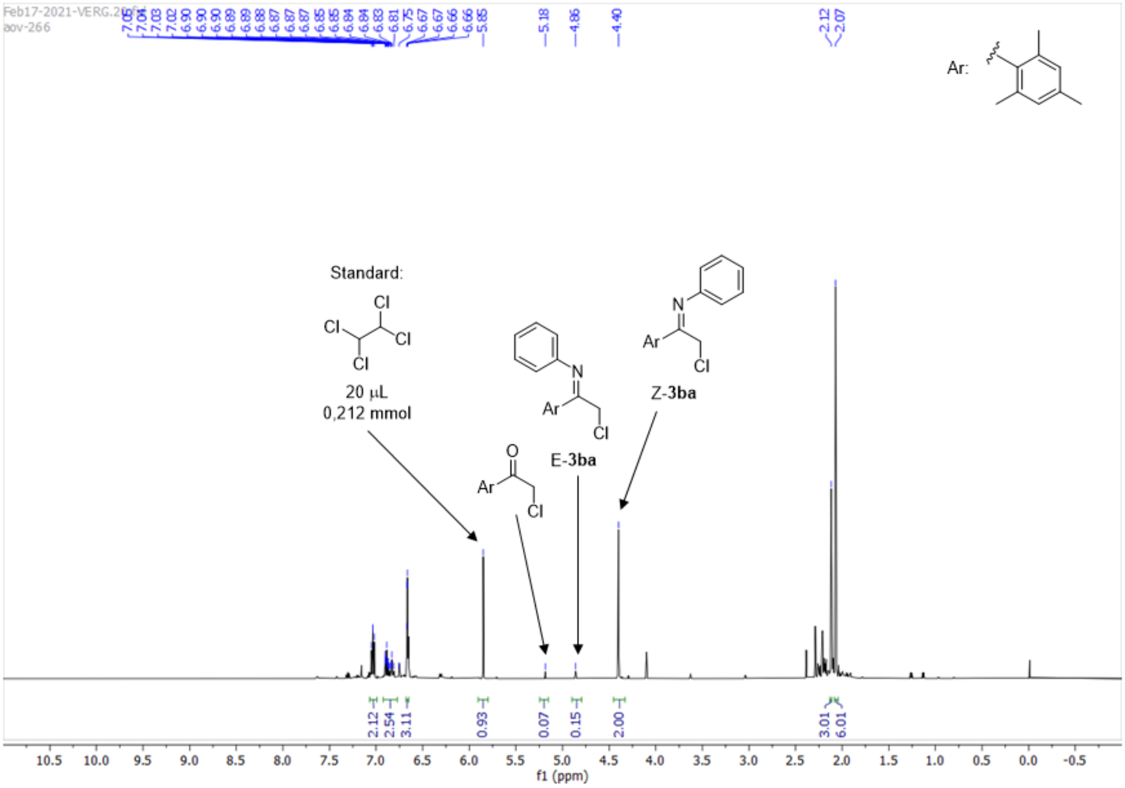

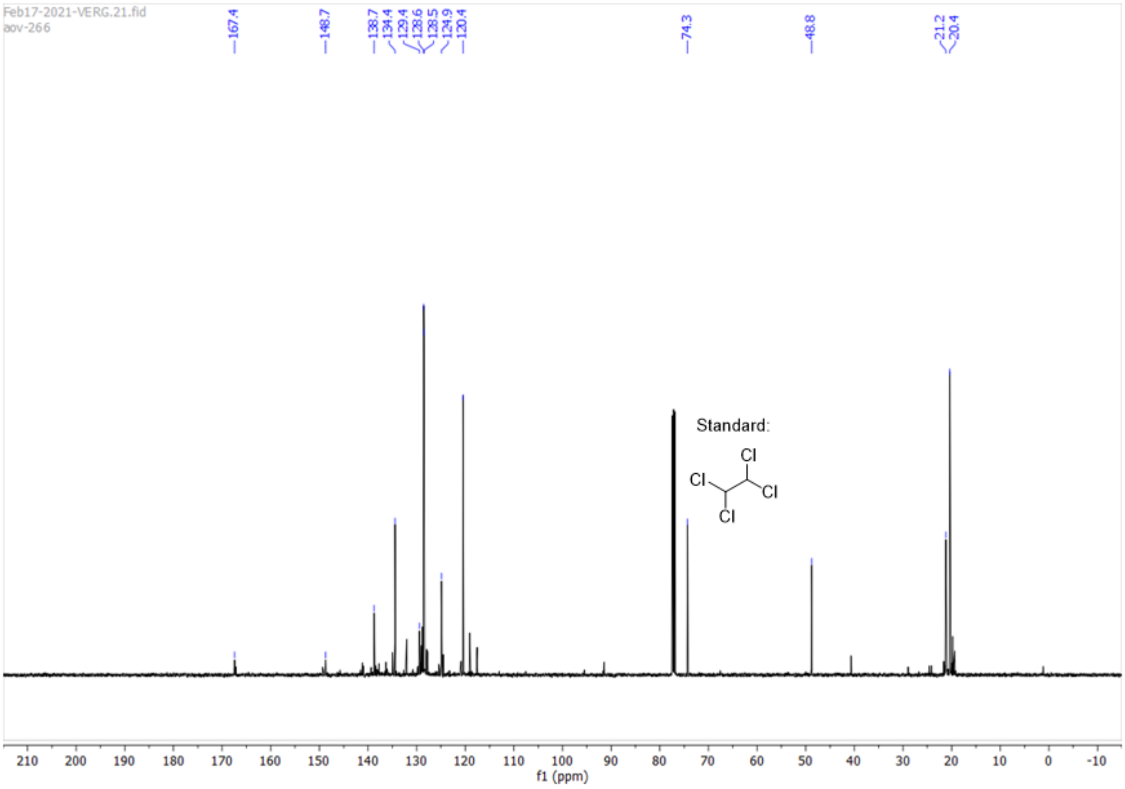

HRMS (+ESI) of crude **3ba**:

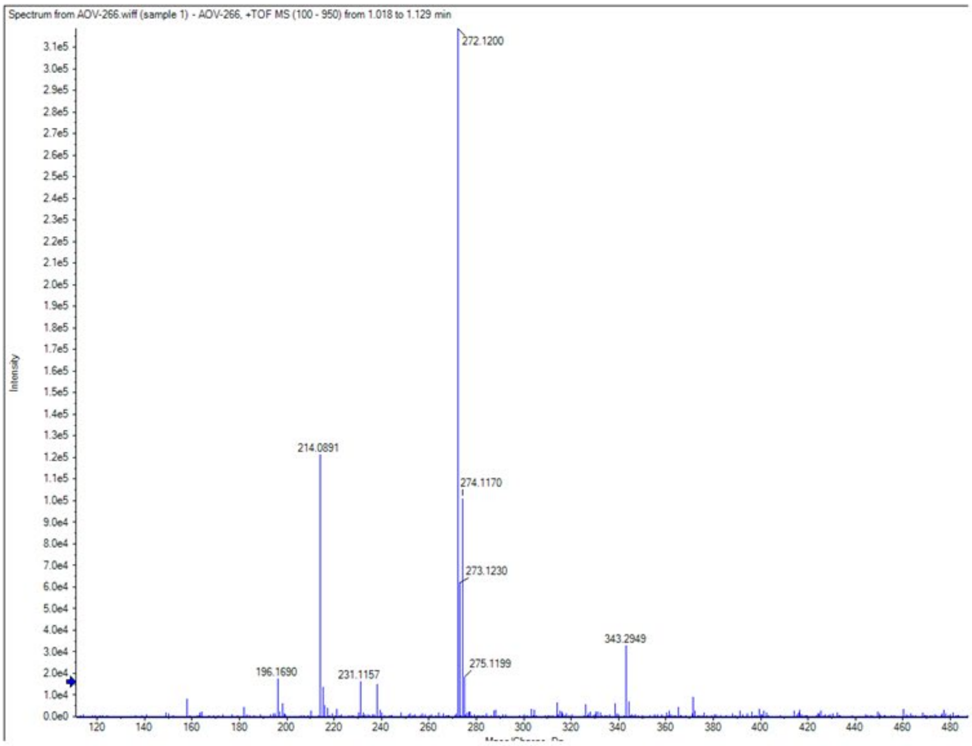

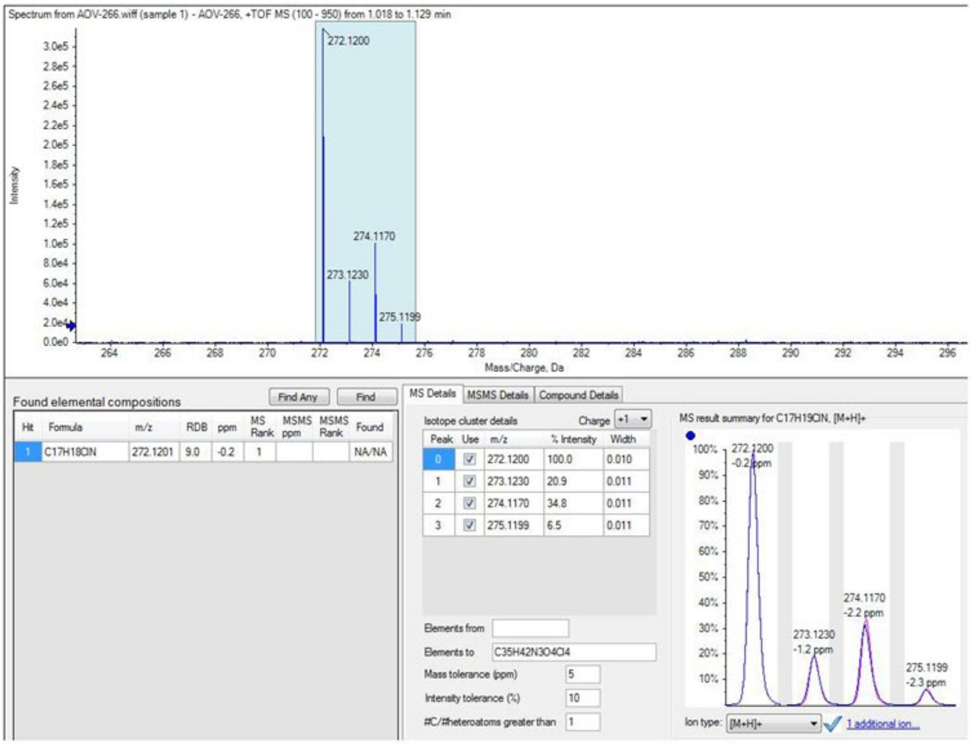

^{1}H (500 MHz) and $^{13}C\{^{1}H\}$ (125 MHz) NMR of crude **3ca** in $CDCl_3$

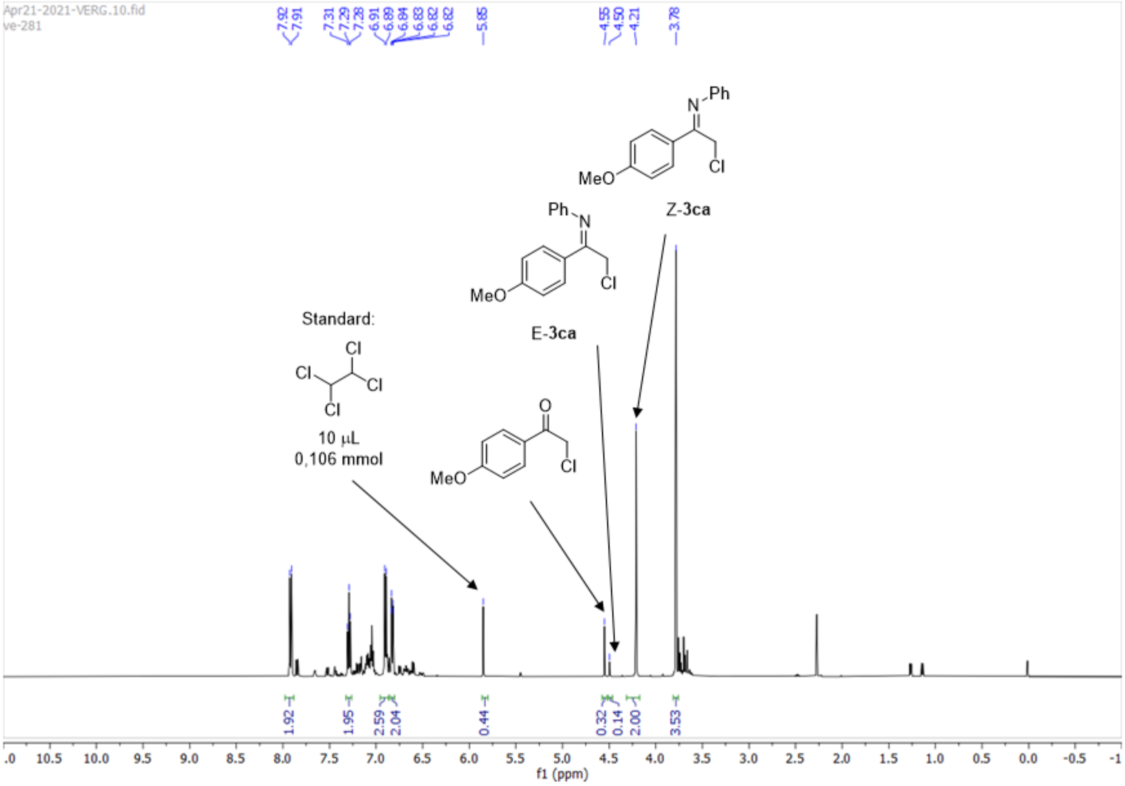

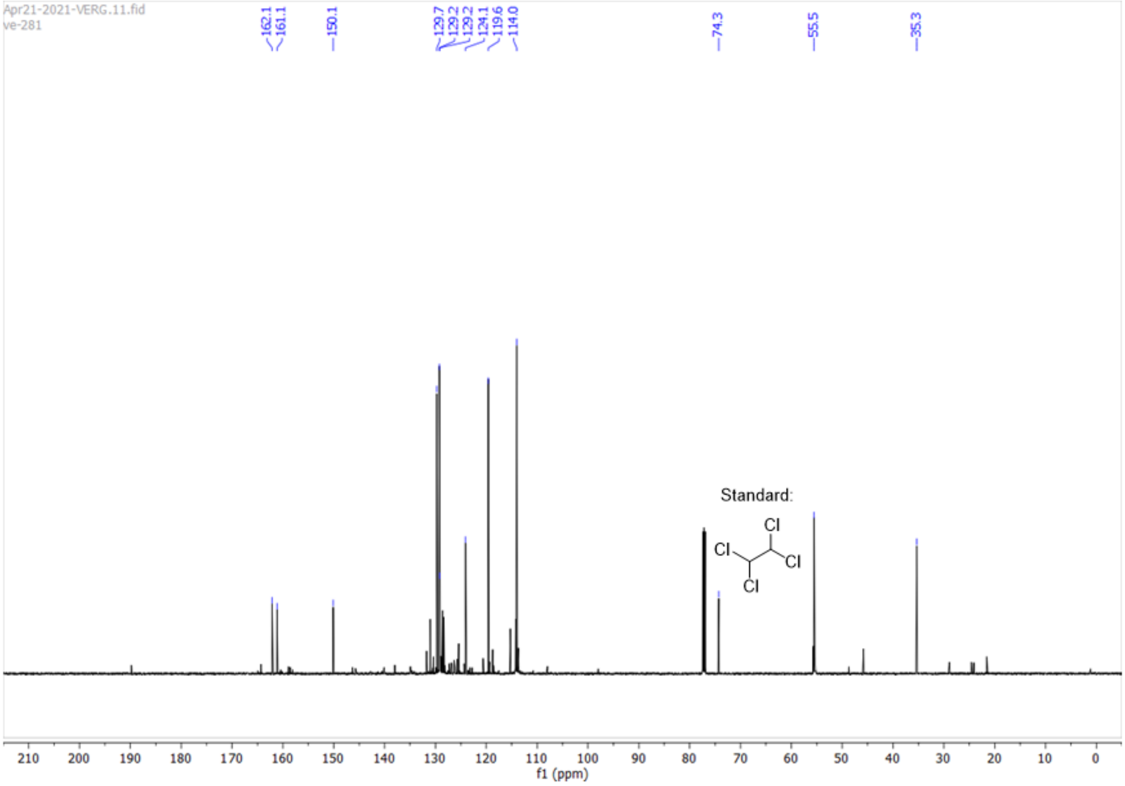

HRMS (+ESI) of crude **3ca**:

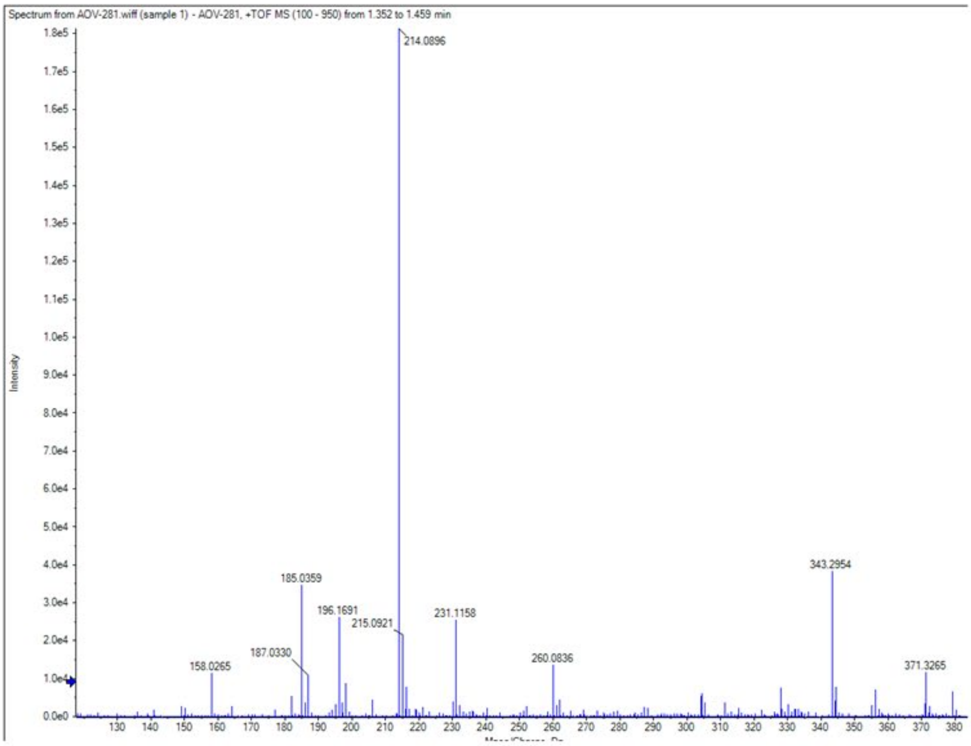

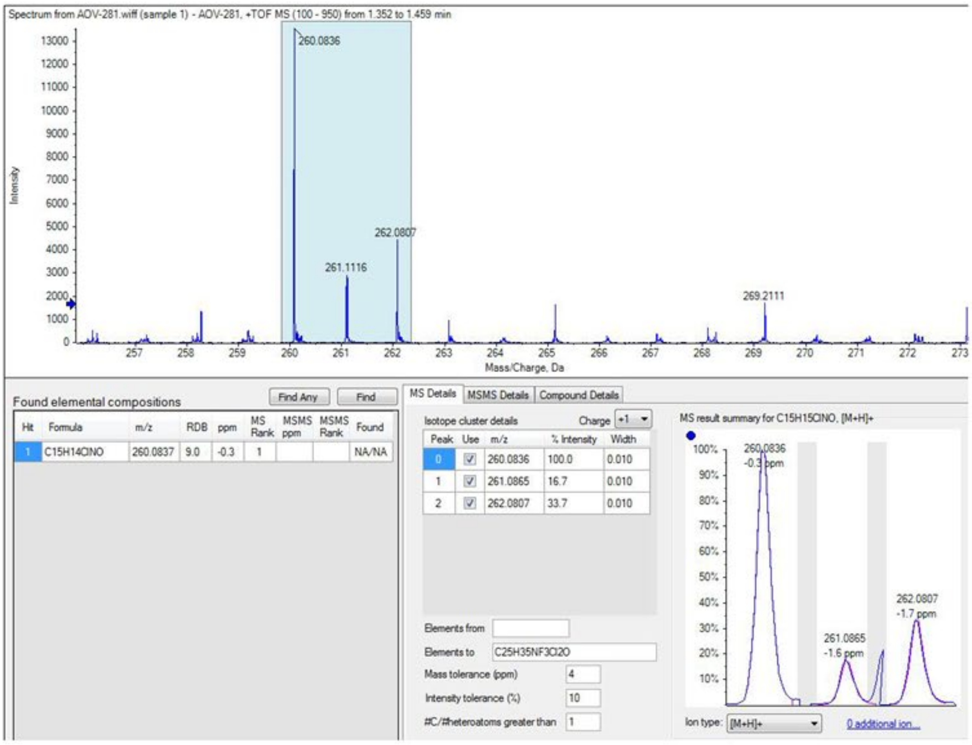

^{1}H (400 MHz) and ^{19}F (470 MHz) NMR of crude **3da** in $CDCl_3$

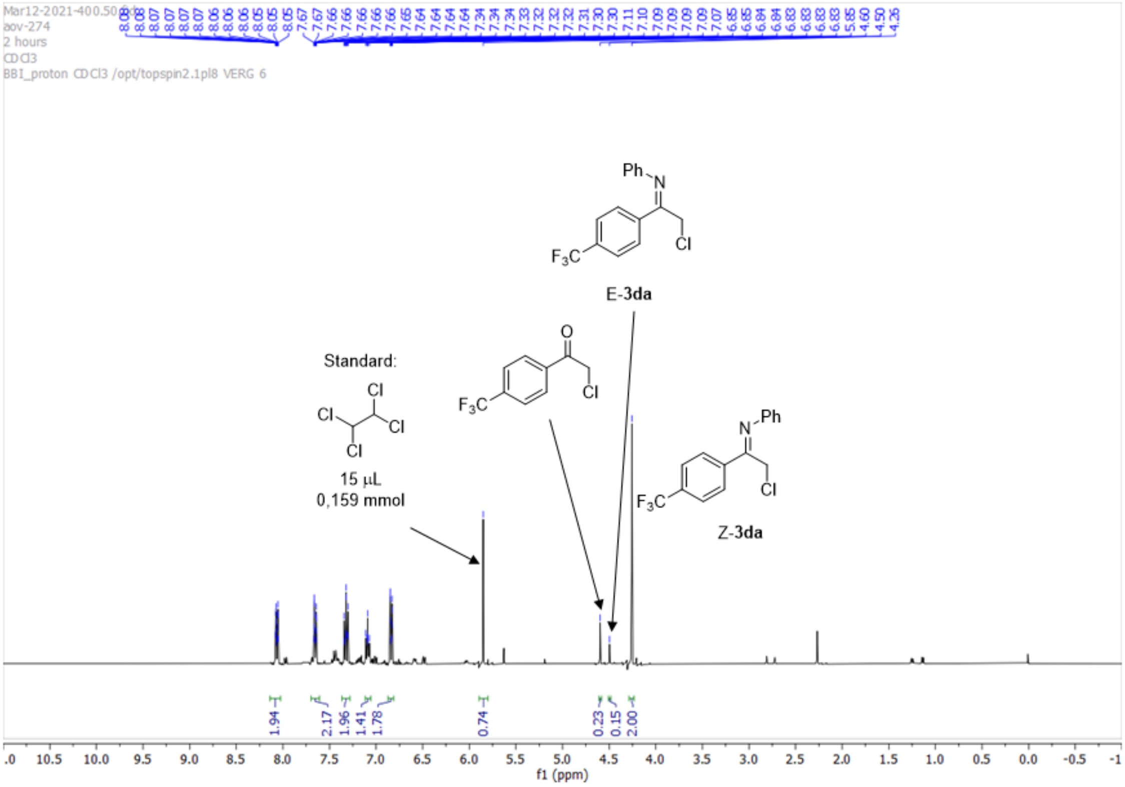

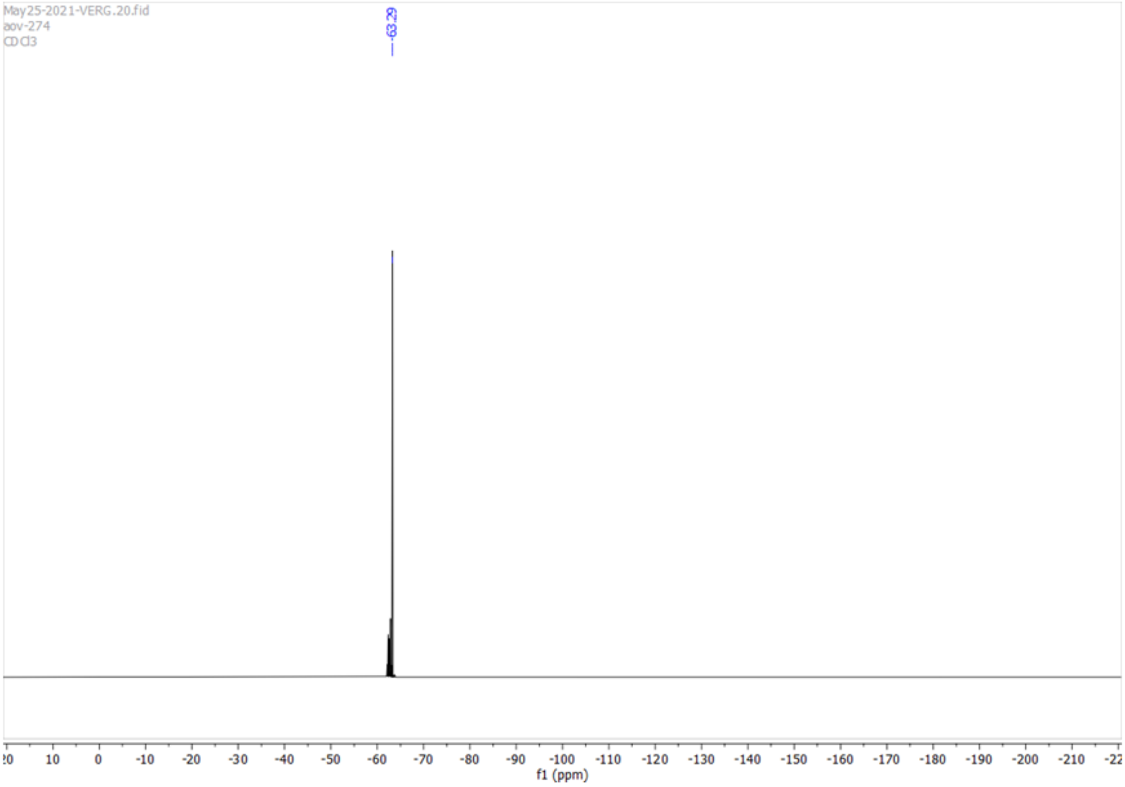

$^{13}C\{^{1}H\}$ (100 MHz) NMR of crude **3da** in $CDCl_3$

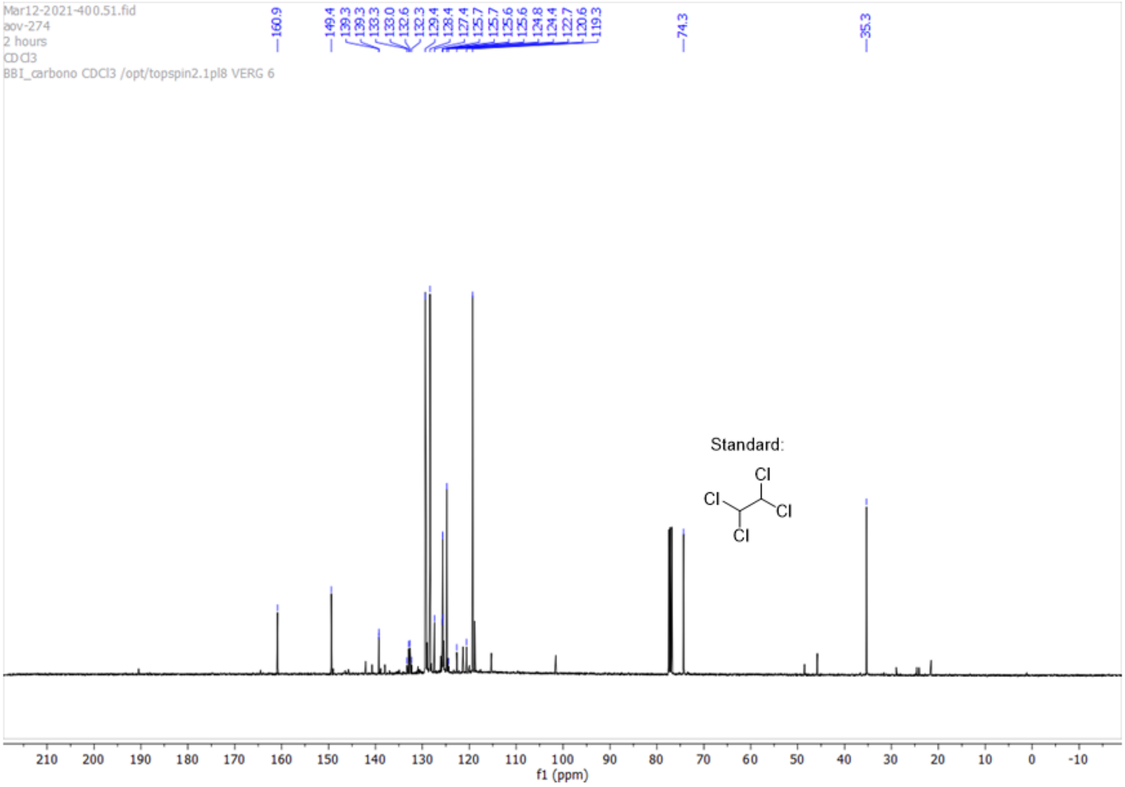

HRMS (+ESI) of crude **3da**:

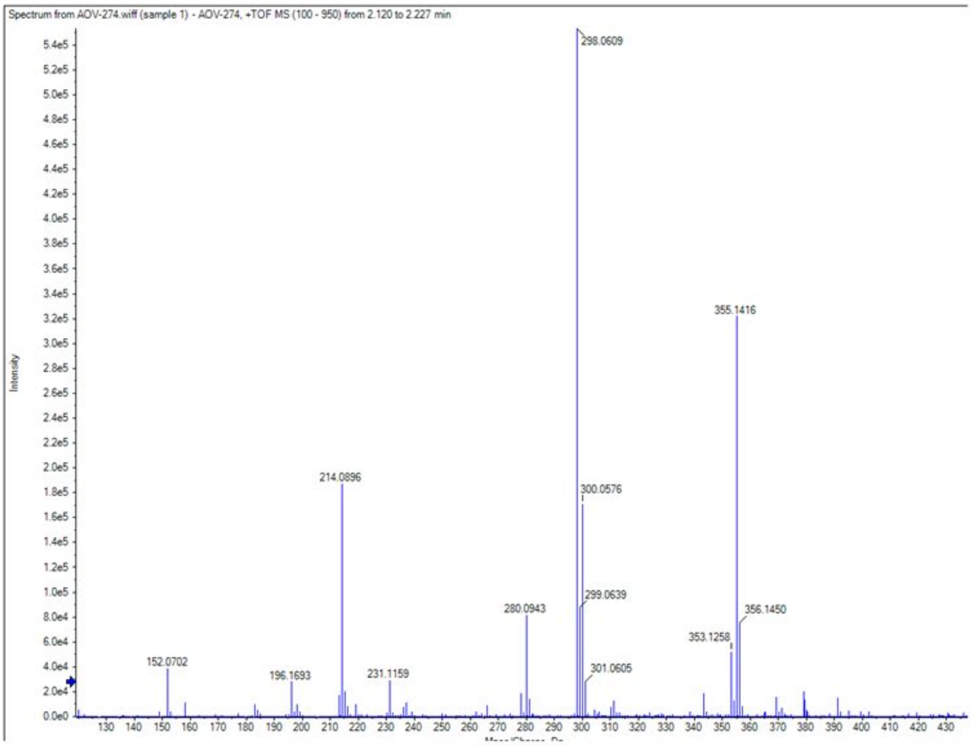

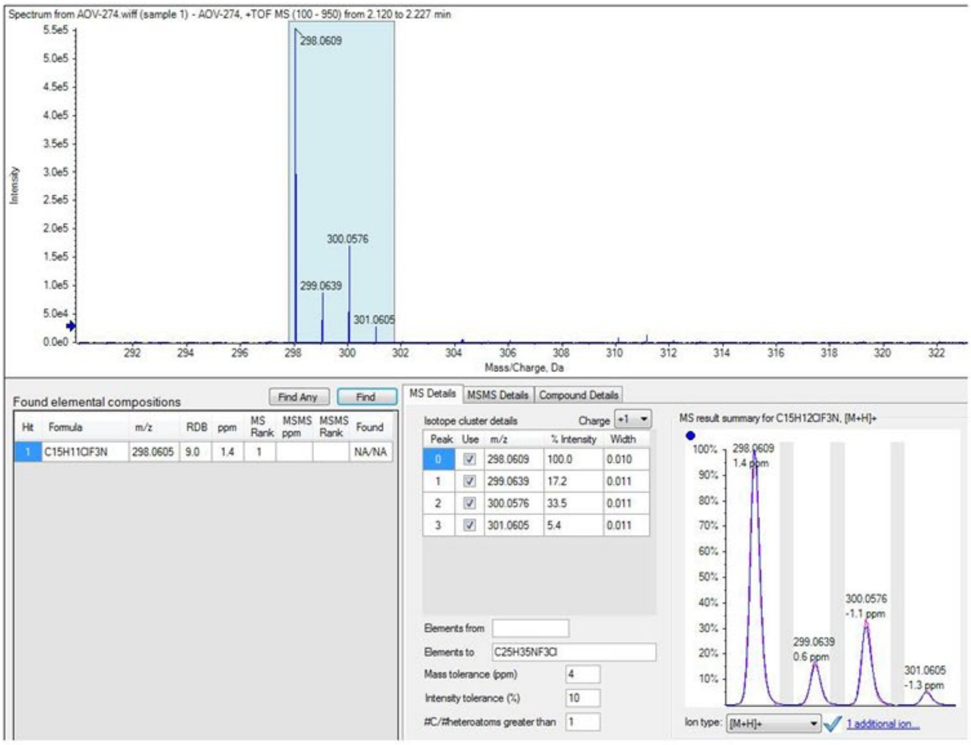

[1]H (500 MHz) and ^{13}C{^{1}H} (125 MHz) NMR of crude **3ea** in $CDCl_3$

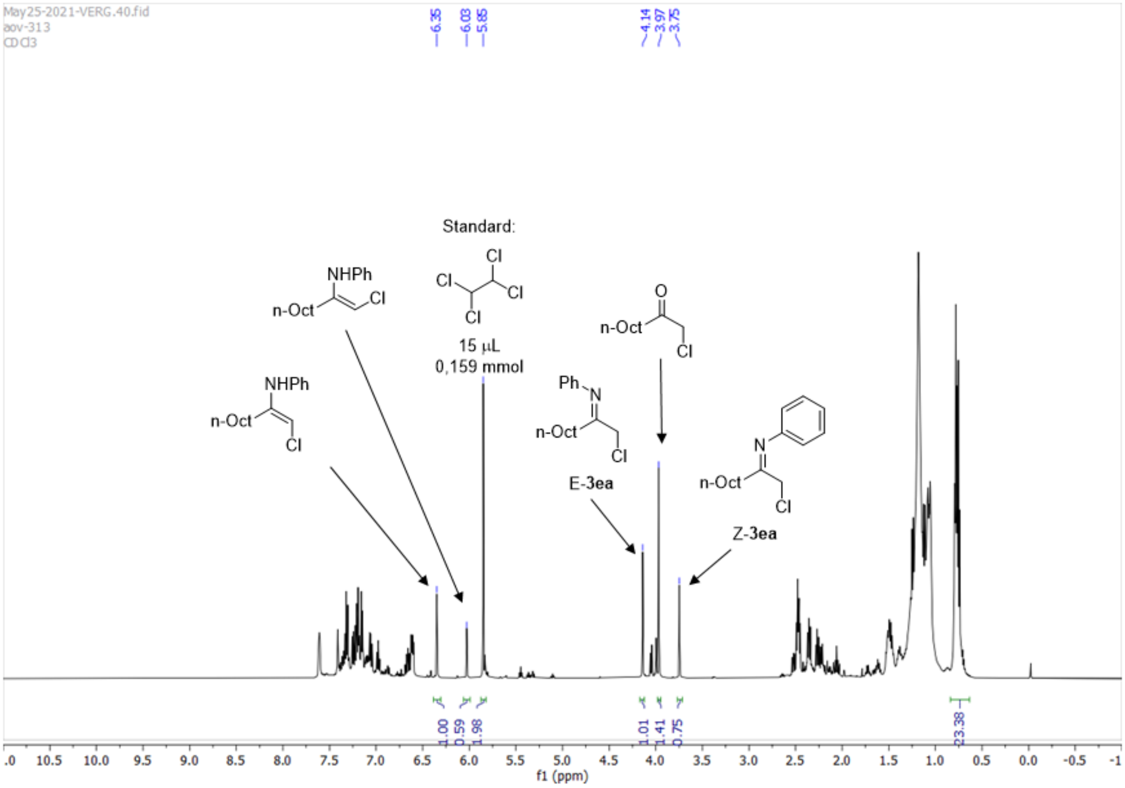

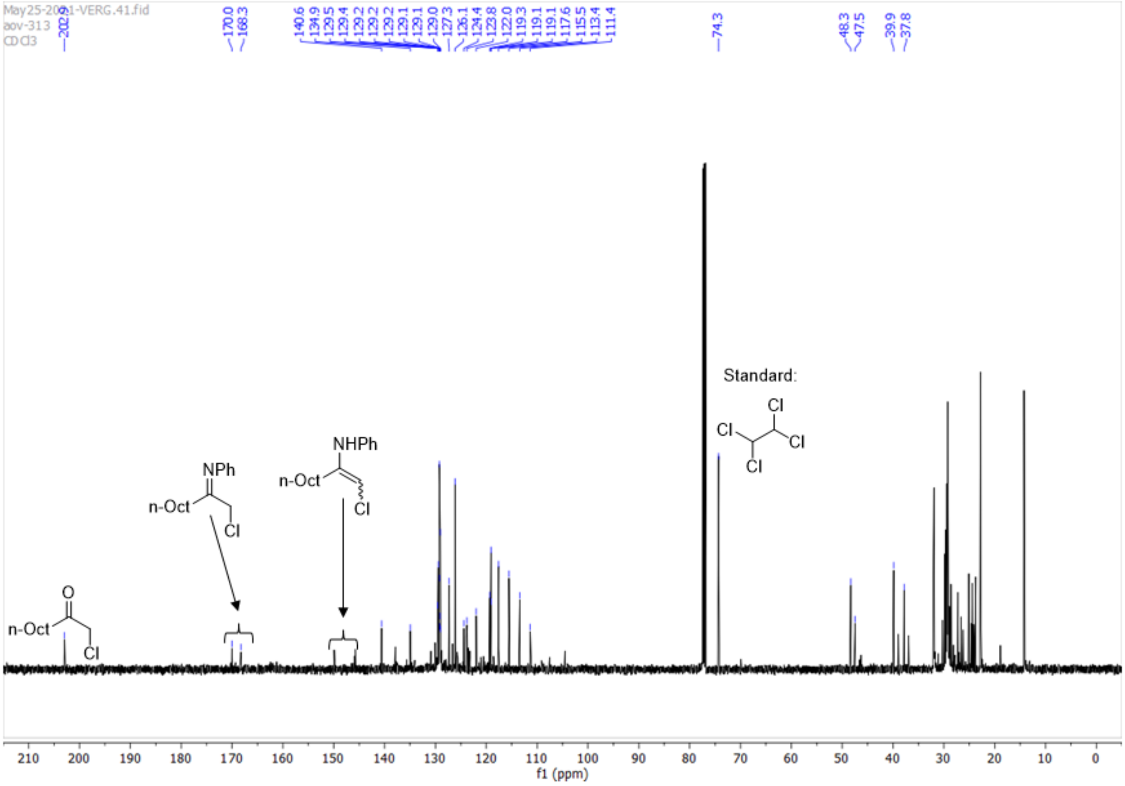

HSQC (500 MHz) NMR of crude **3ea** in $CDCl_3$

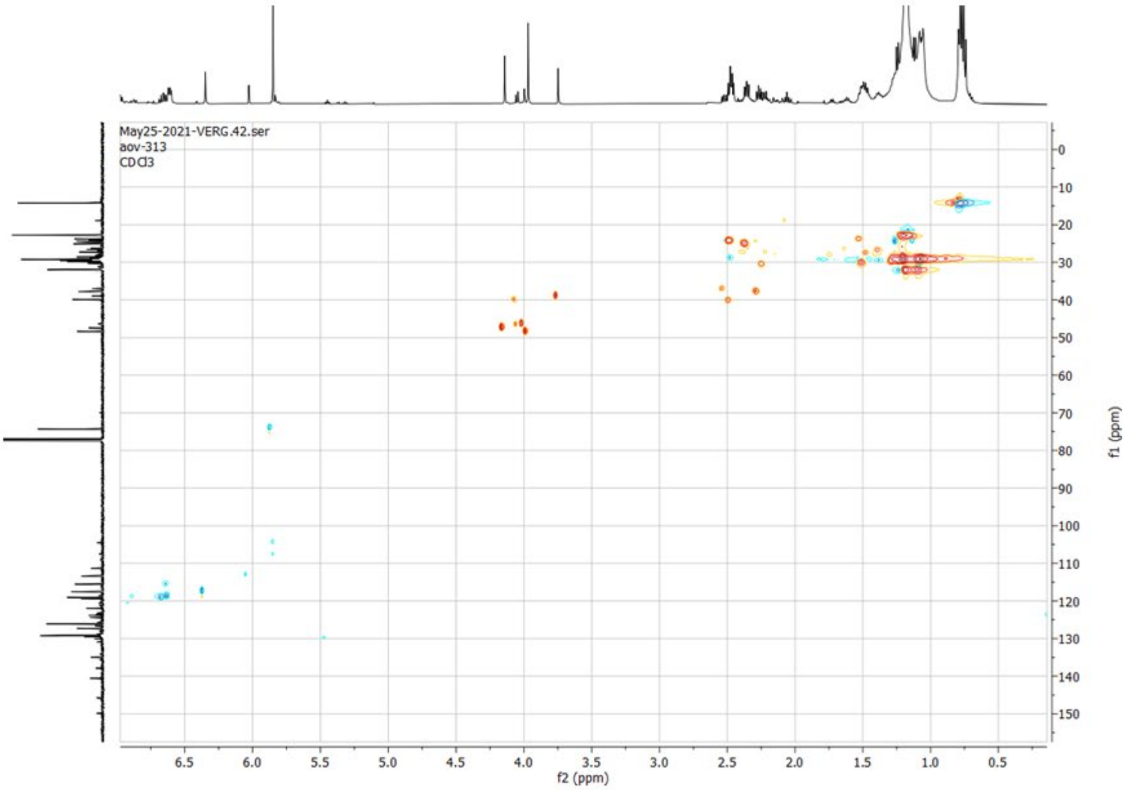

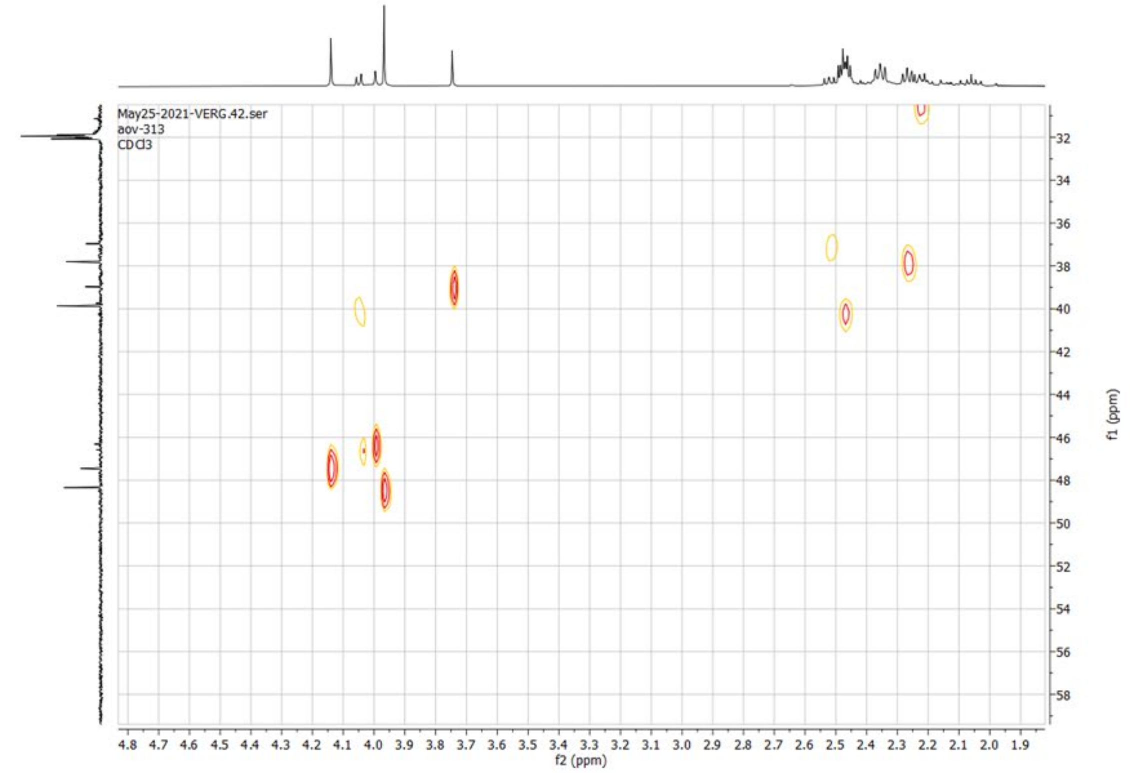

HSQC (500 MHz) NMR of crude **3ea** in $CDCl_3$

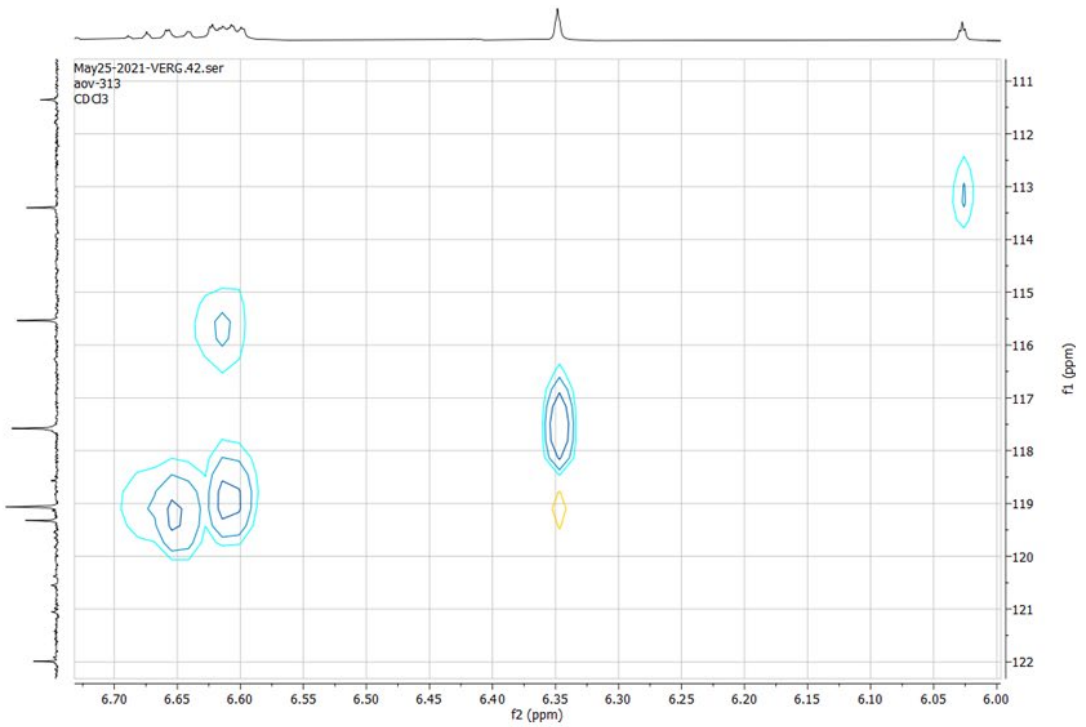

GC-MS of crude **3ea** in $CDCl_3$/DCM

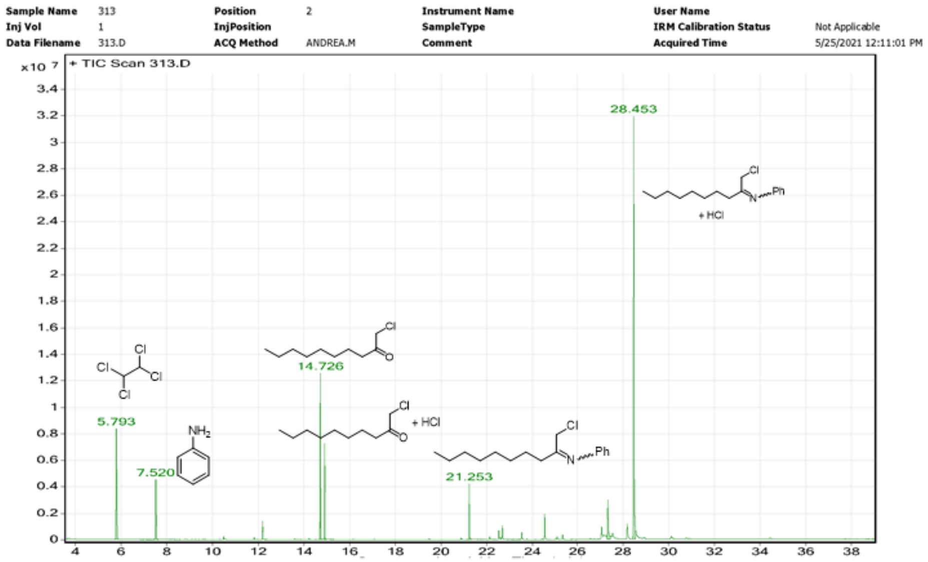

Peaks: + TIC Scan (313.D)

Peak	RT	Area	Area %	Area Sum %	Height	Width	FWHM
1	5.793	19544679.9	32.51	14.74	8367967.7	0.201	0.036
2	7.52	9289116.73	15.45	7.01	4528744.3	0.181	0.031
3	14.726	18686581.3	31.08	14.09	12556936.	0.122	0.023
4	14.914	10705745.7	17.81	8.08	7276128.2	0.123	0.023
5	21.253	6647045.02	11.06	5.01	4213724.1	0.129	0.024
6	27.321	7583476.68	12.61	5.72	2983080.1	0.149	0.03
7	28.45	60121004.6	100	45.35	31920465.	0.136	0.026

GC-MS of crude **3ea** in $CDCl_3$/DCM (cont.)

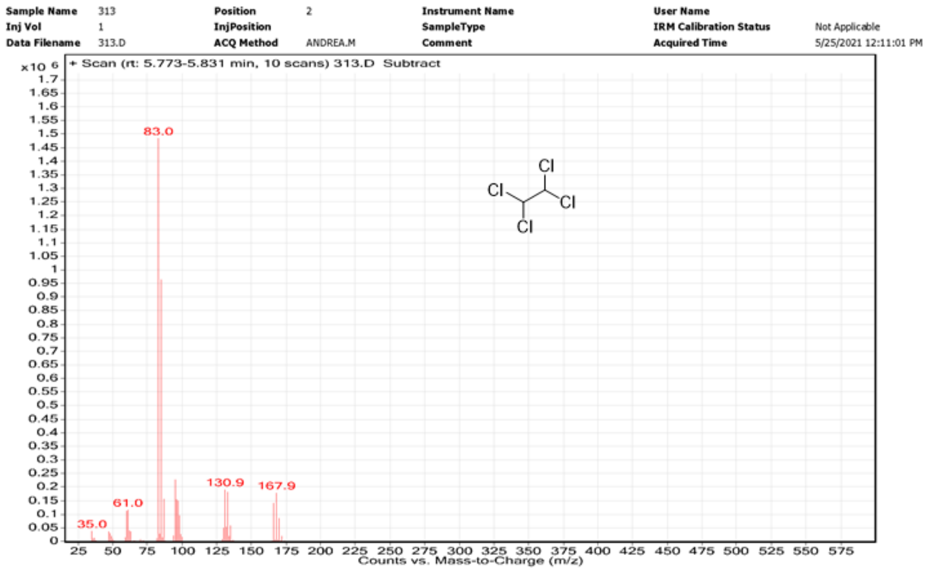

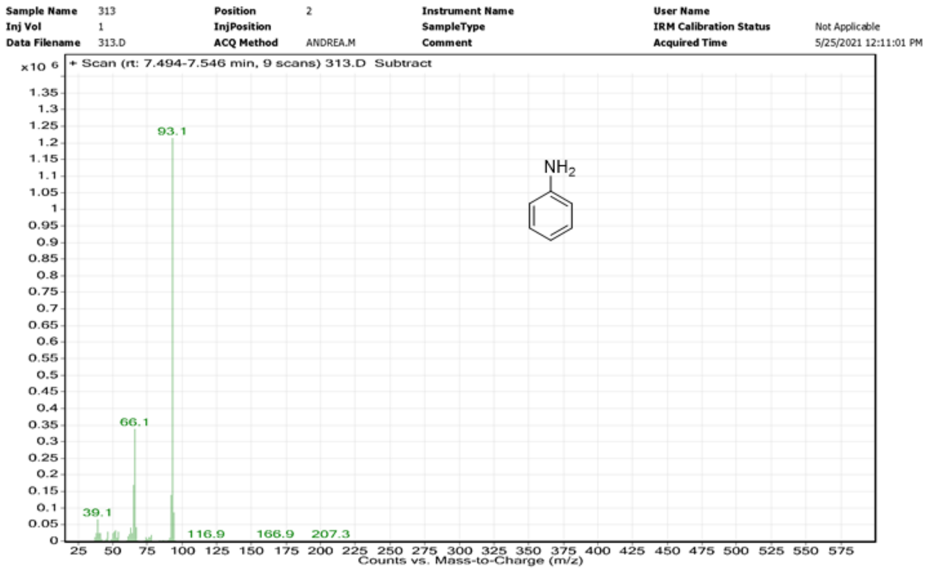

GC-MS of crude **3ea** in $CDCl_3$/DCM (cont.)

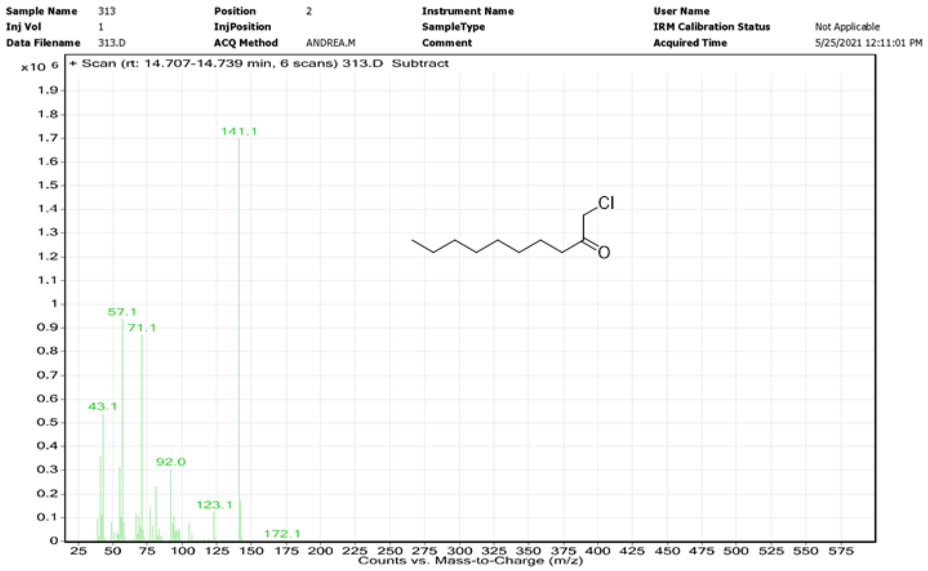

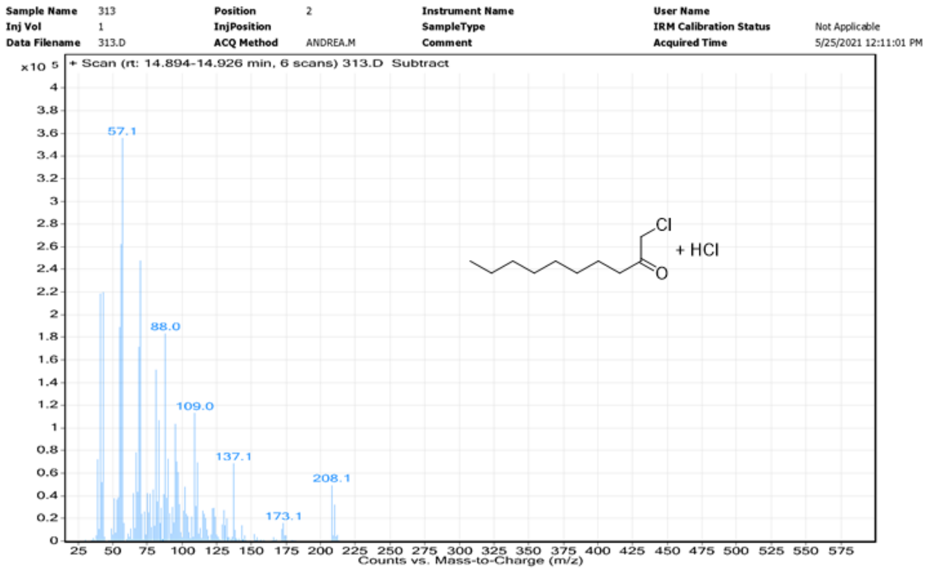

GC-MS of crude **3ea** in $CDCl_3$/DCM (cont.)

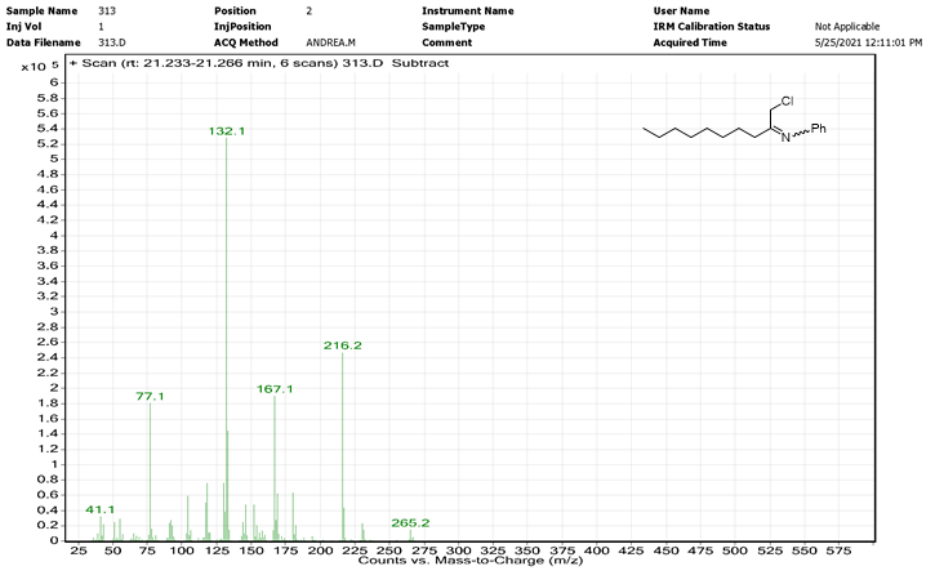

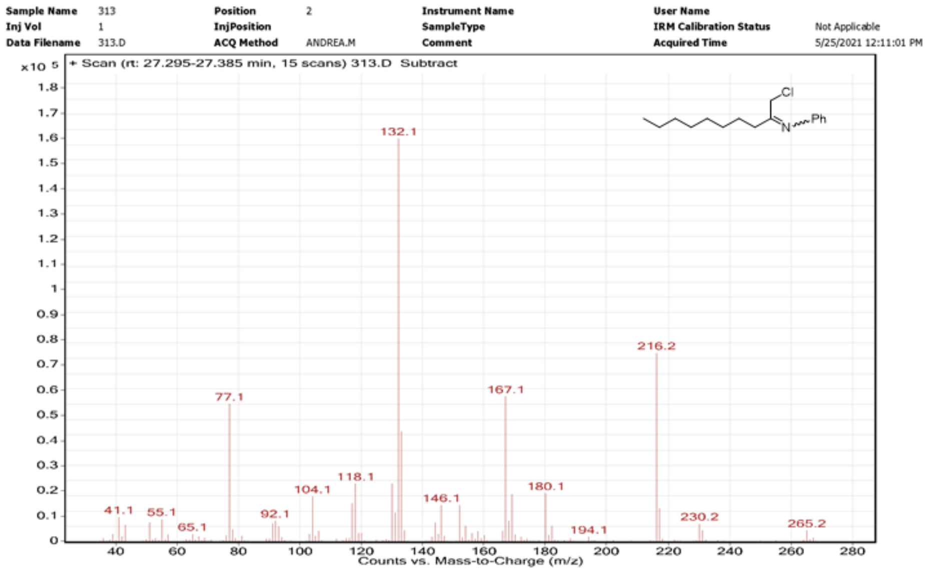

GC-MS of crude **3ea** in $CDCl_3$/DCM (cont.)

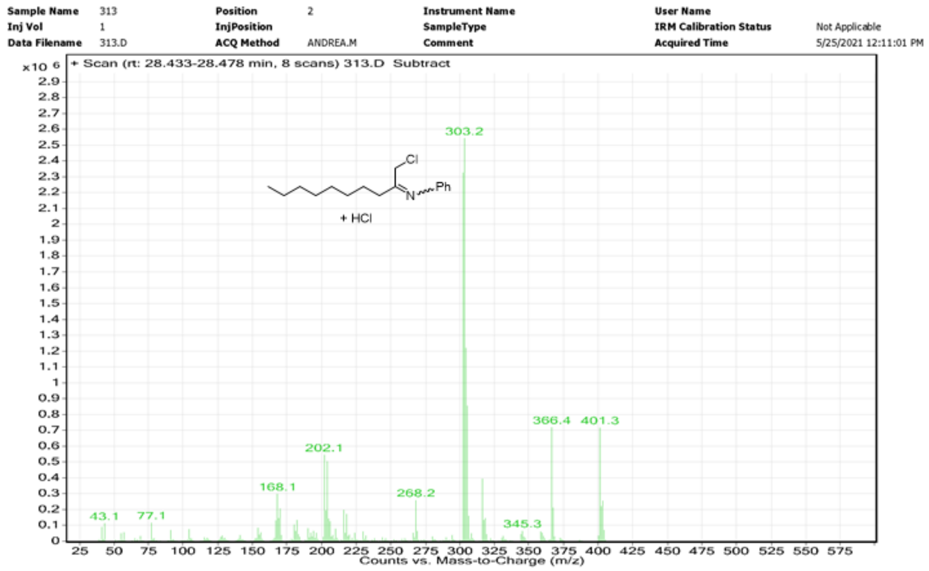

HRMS (+ESI) of crude **3ea**:

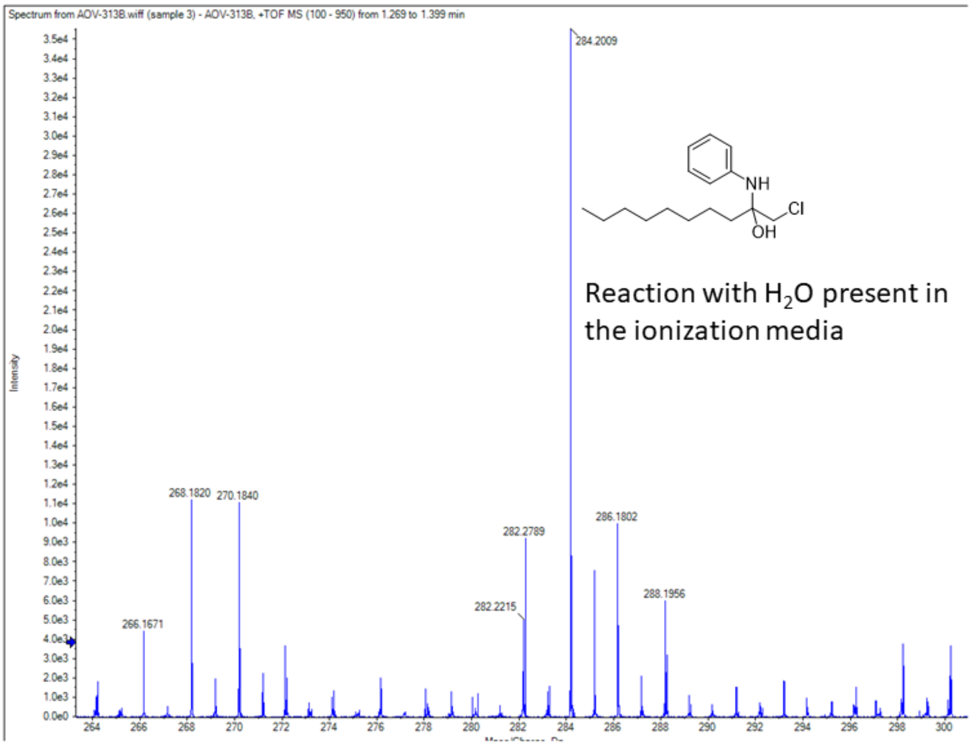

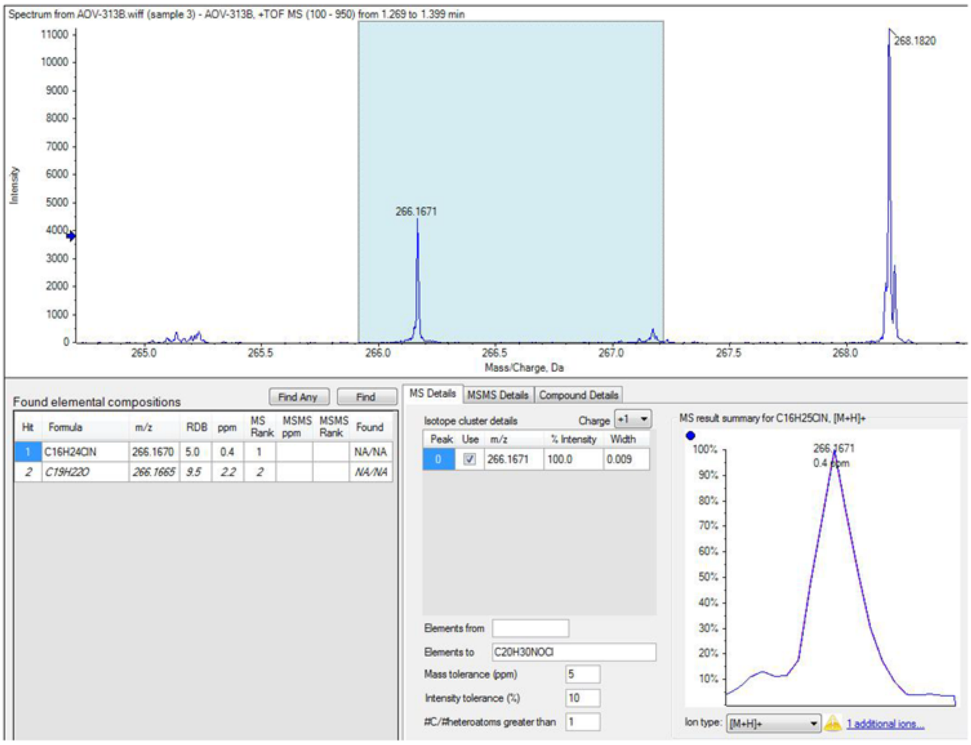

^{1}H (500 MHz) and $^{13}C\{^{1}H\}$ (125 MHz) NMR of crude **3bb** in $CDCl_3$

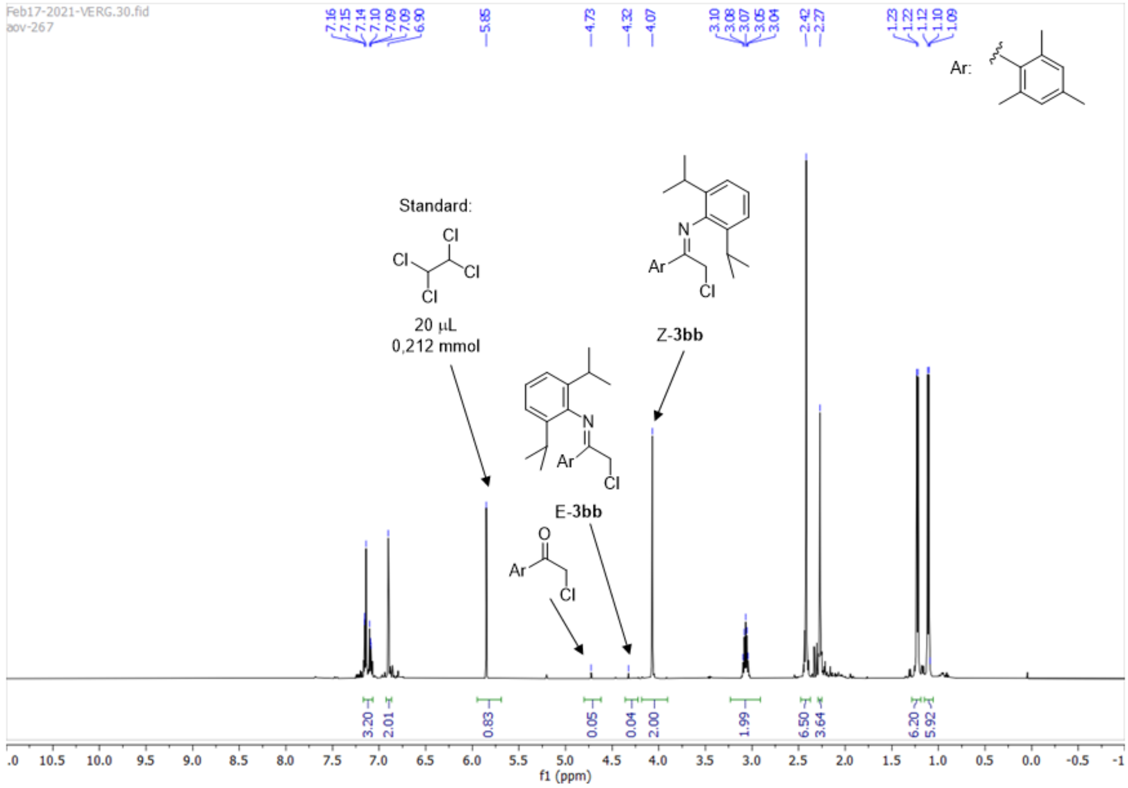

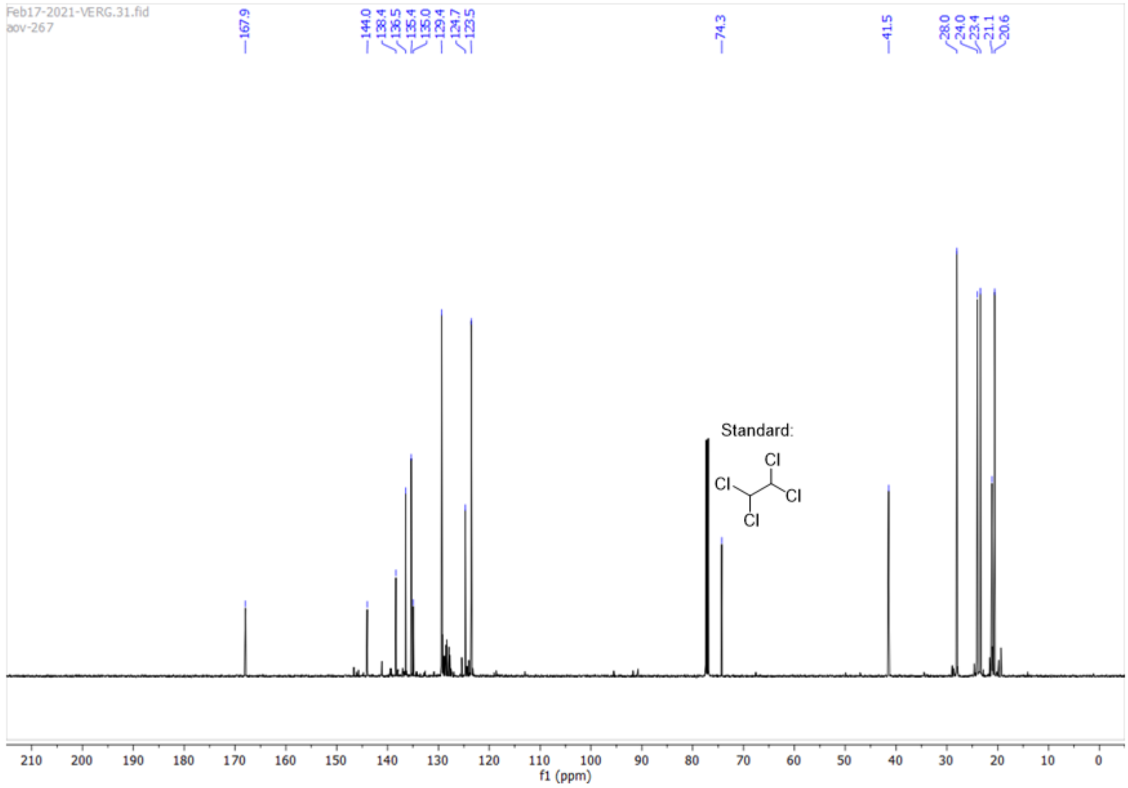

HRMS (+ESI) of crude **3bb**:

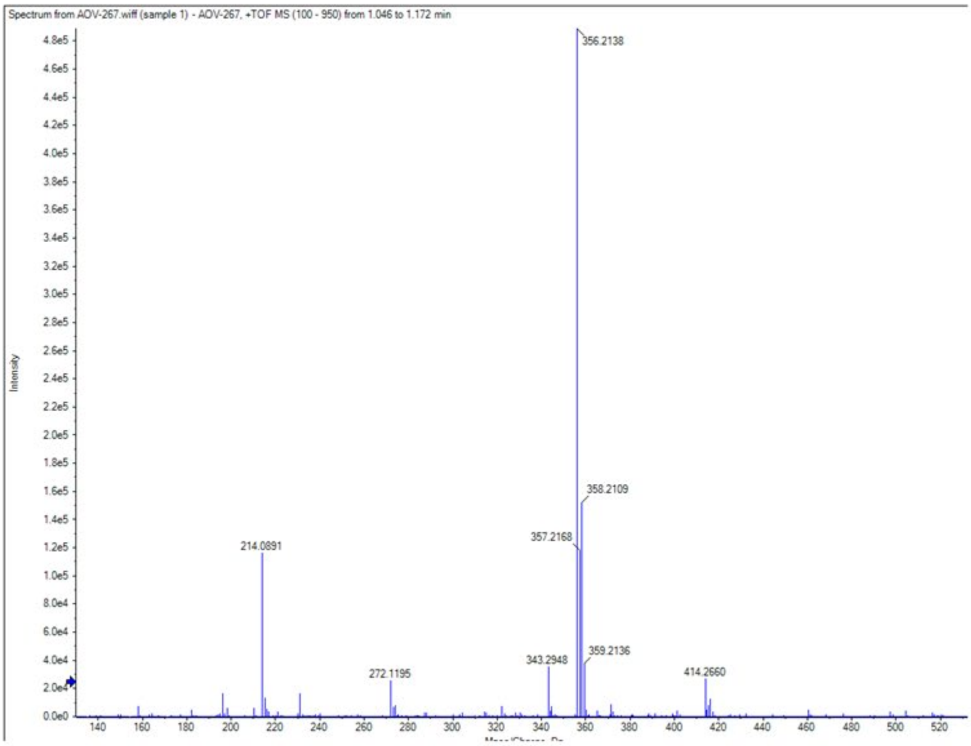

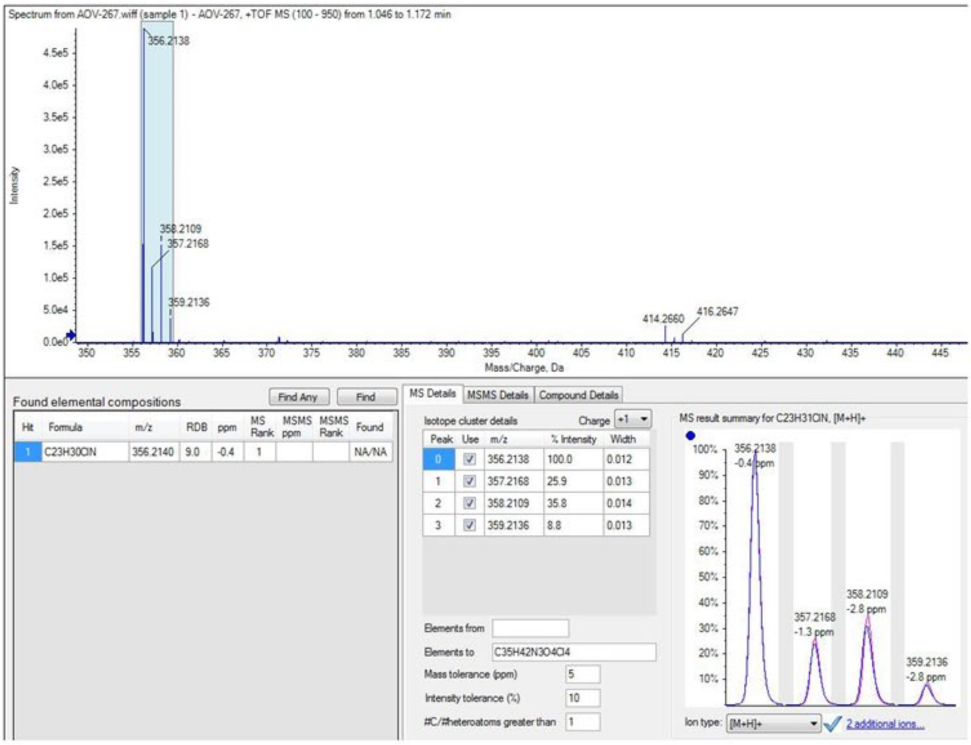

^{1}H (300 MHz) and $^{13}C\{^{1}H\}$ (75 MHz) NMR of crude **3cc** in $CDCl_3$

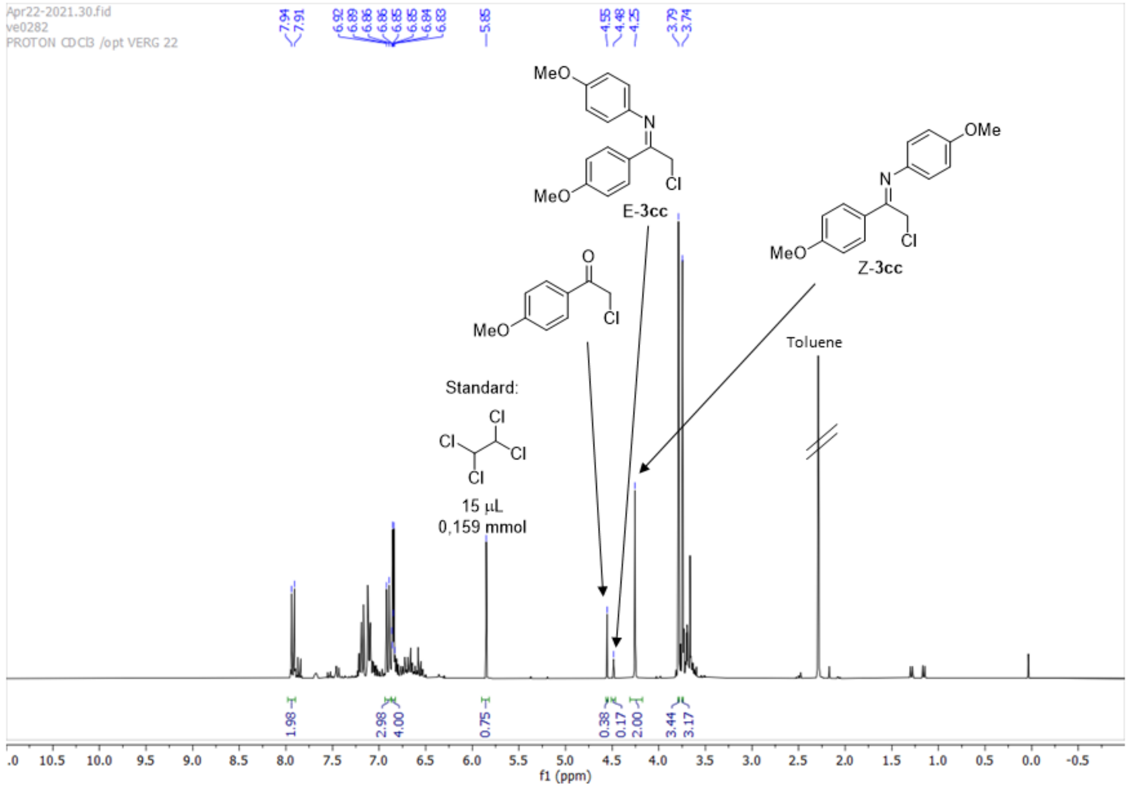

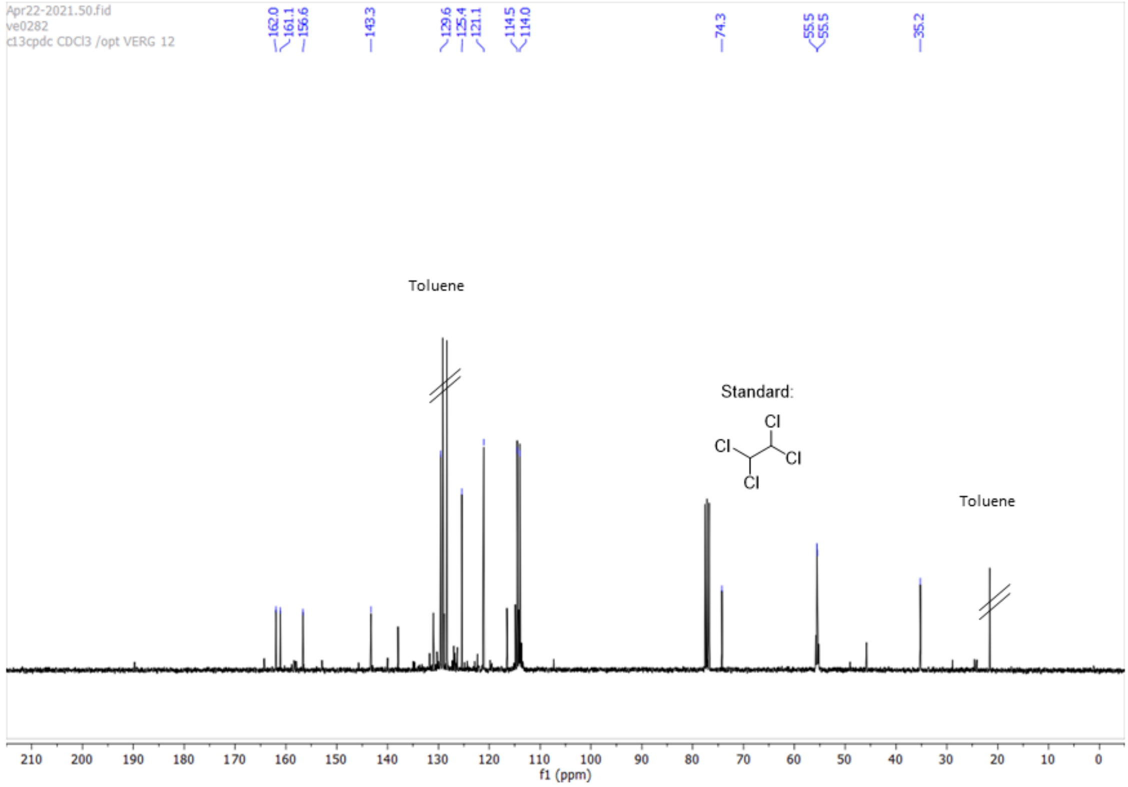

HRMS (+ESI) of crude **3cc**:

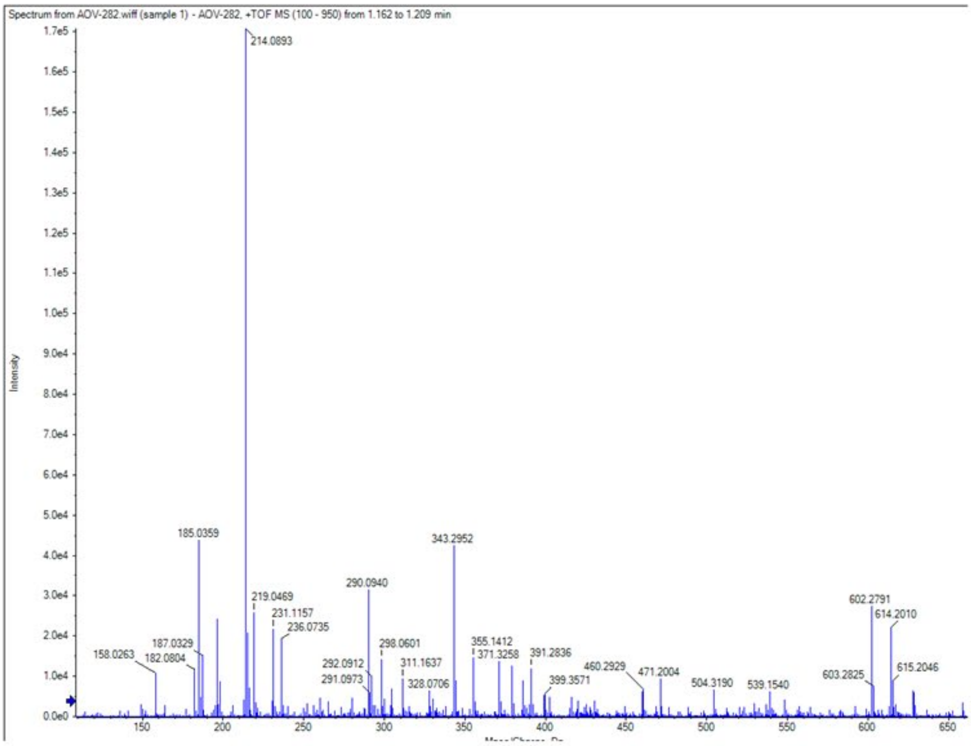

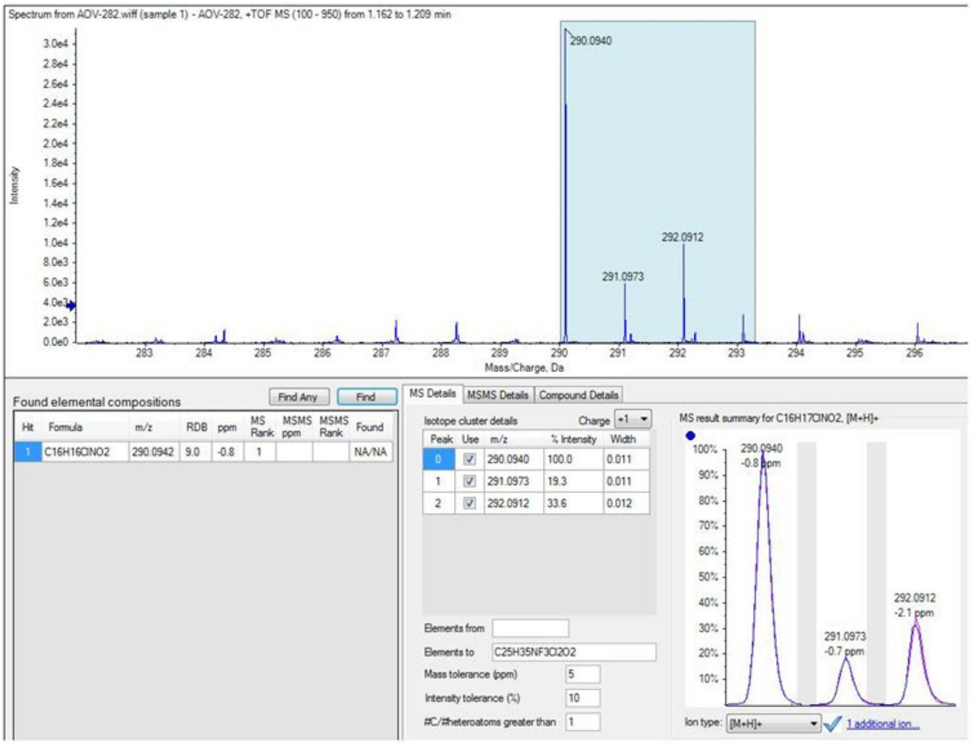

^{1}H (500 MHz) and $^{13}C\{^{1}H\}$ (125 MHz) NMR of crude **3cd** in $CDCl_3$

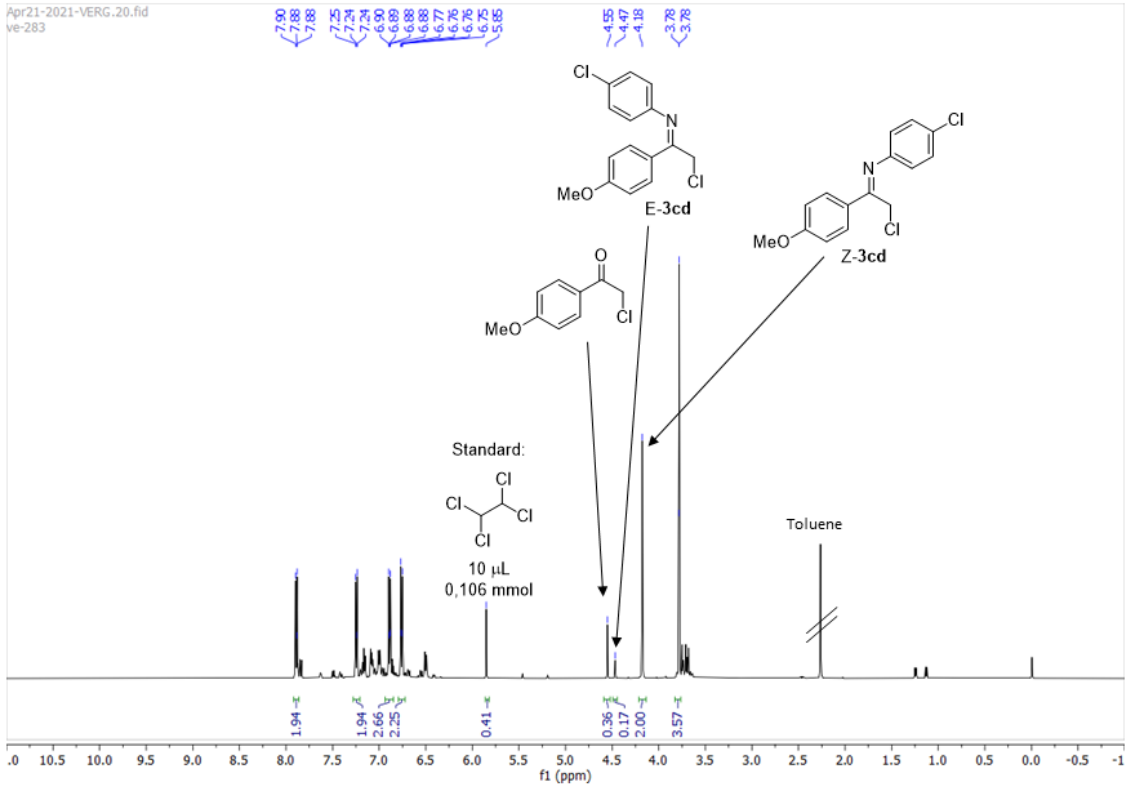

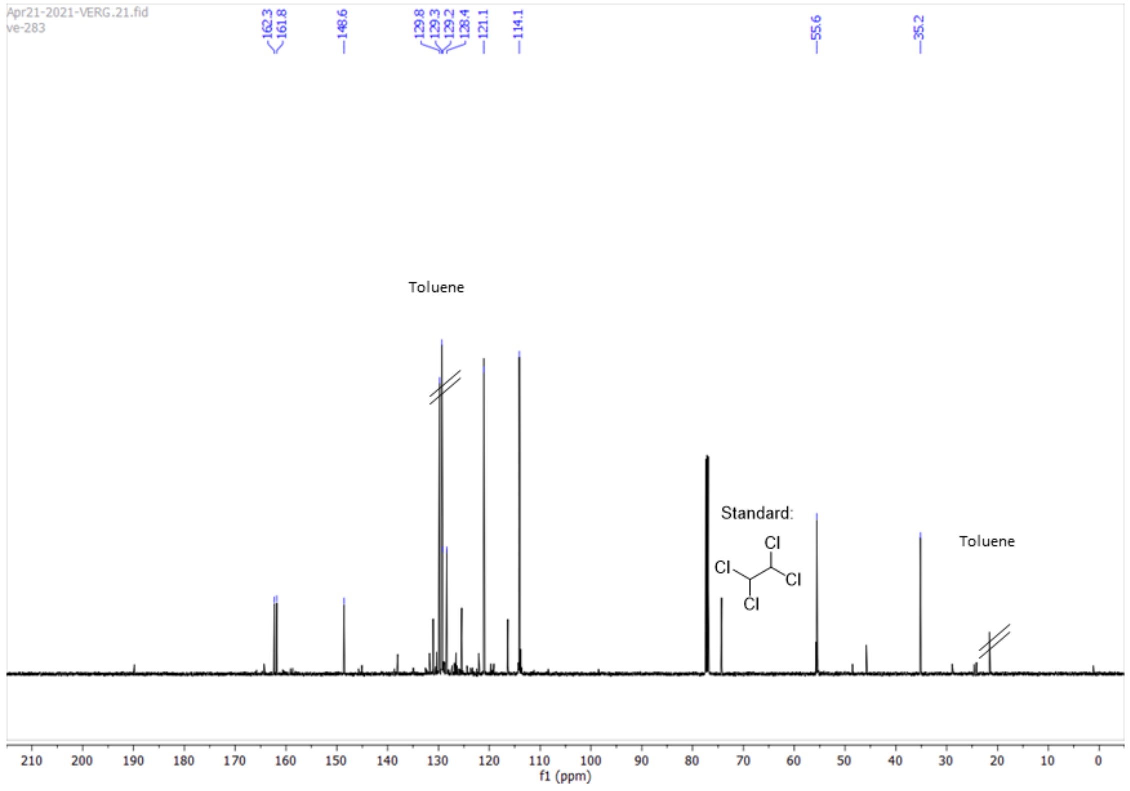

HRMS (+ESI) of crude **3cd**:

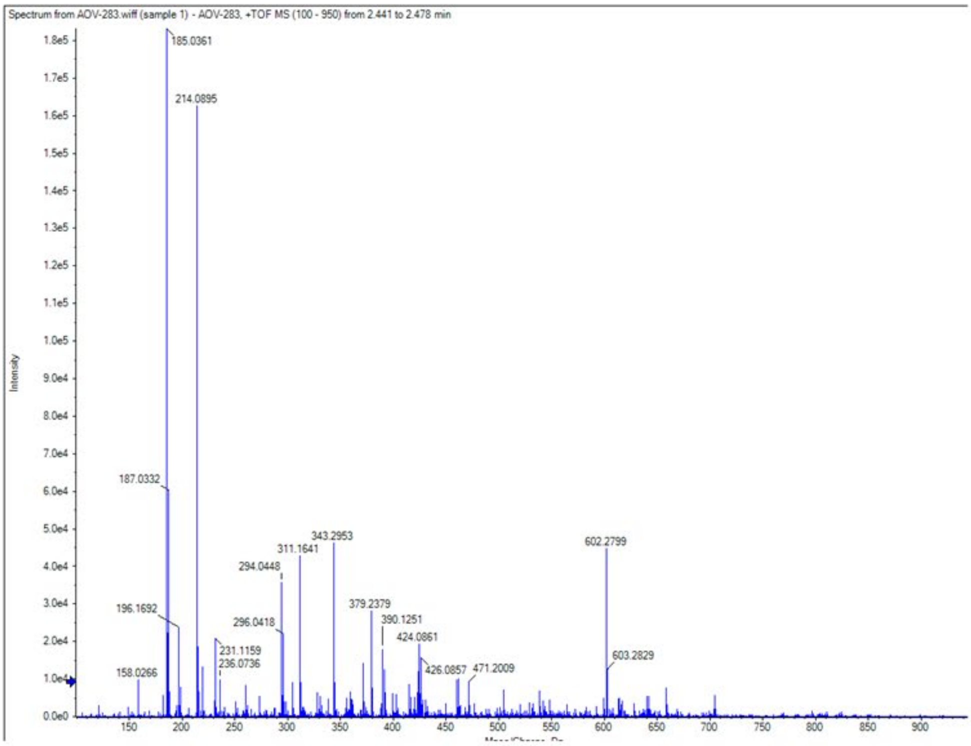

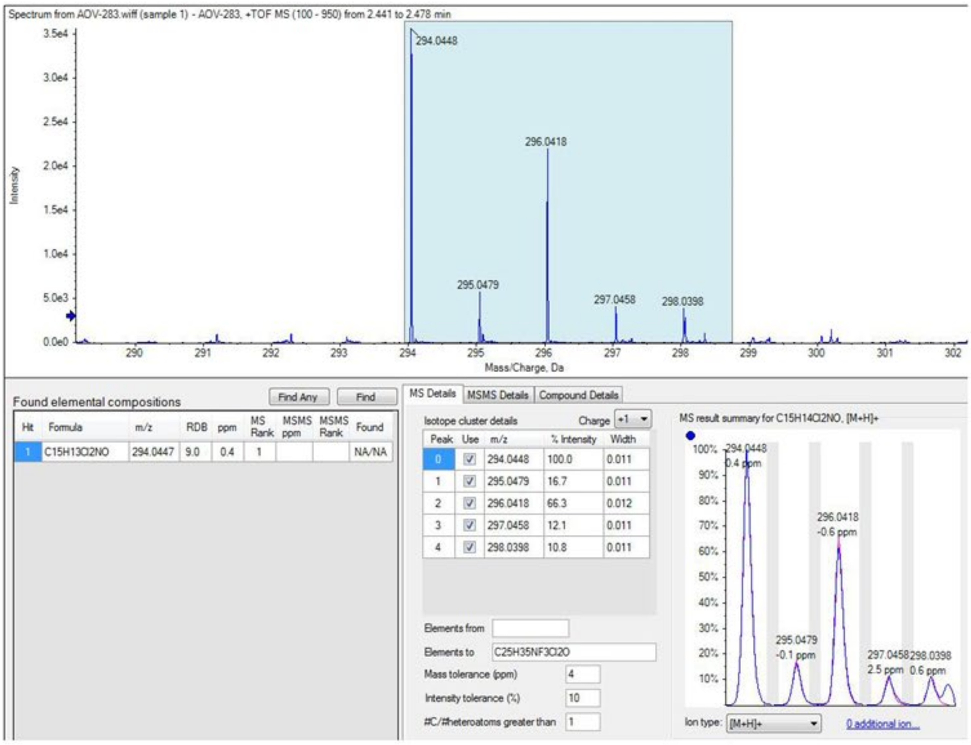

^1H (500 MHz) and ^{19}F (470 MHz) NMR of crude **3dc** in $CDCl_3$

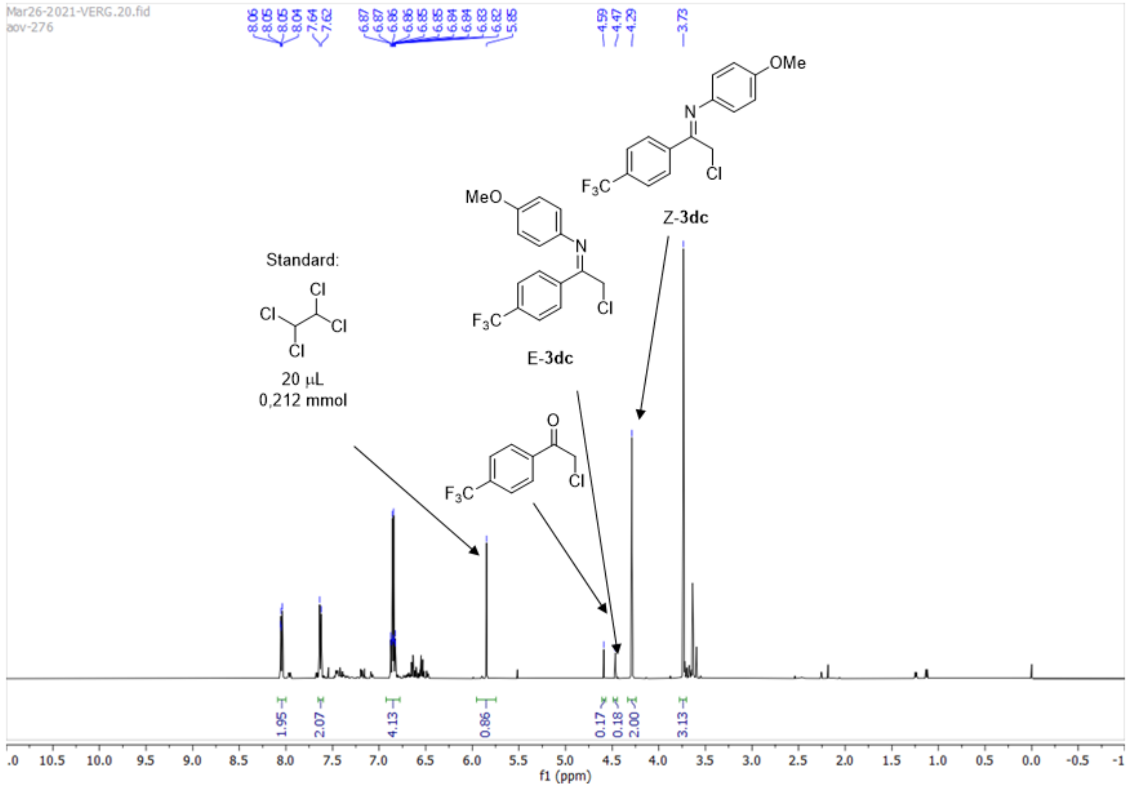

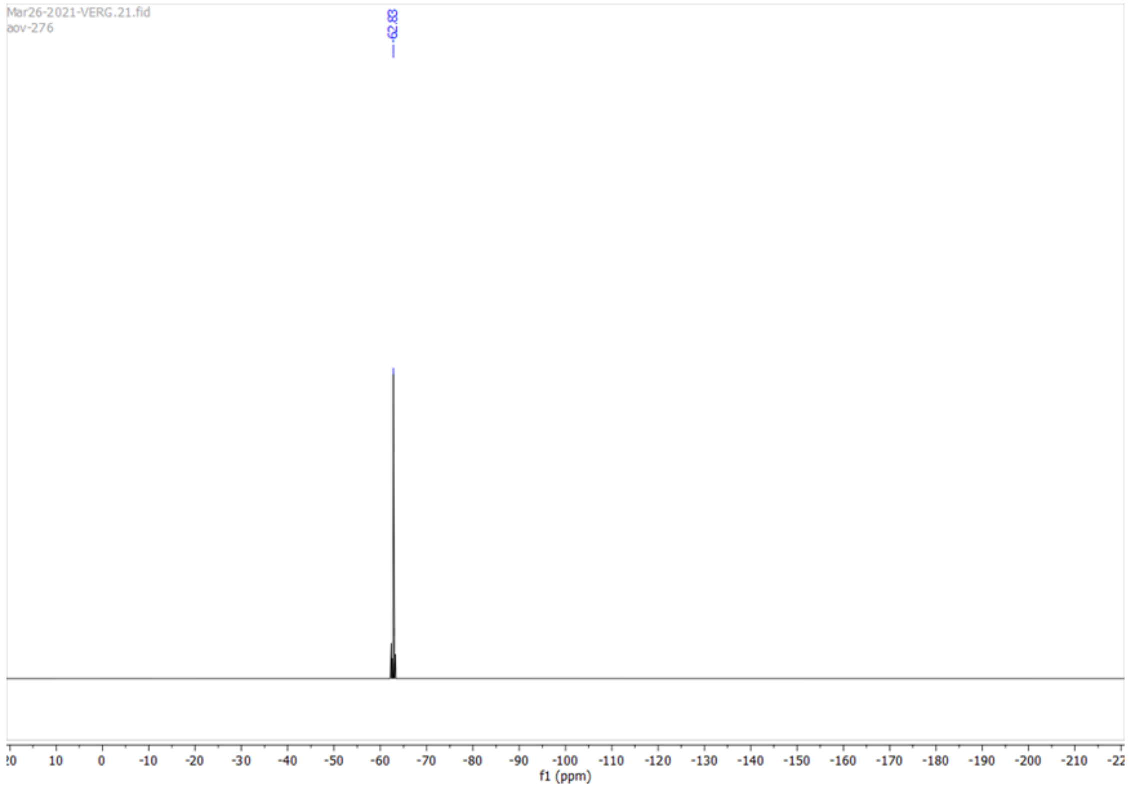

$^{13}C\{^1H\}$ (125 MHz) NMR of crude **3dc** in $CDCl_3$

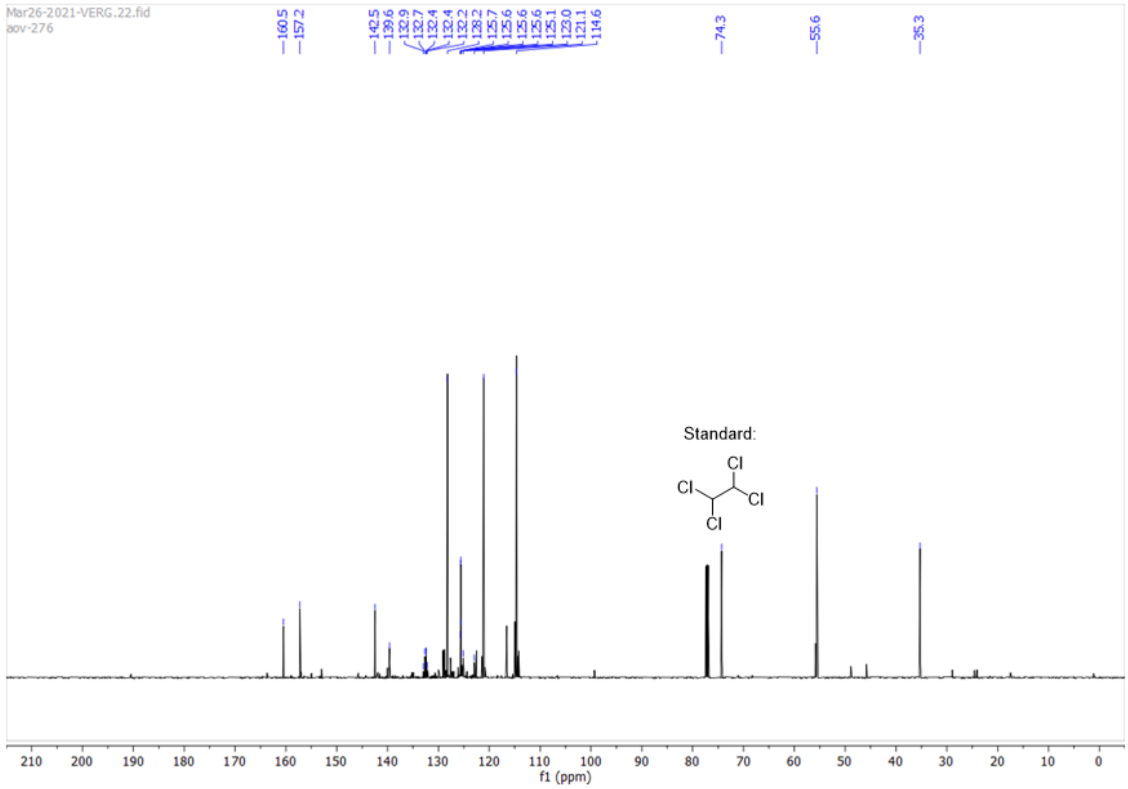

HRMS (+ESI) of crude **3dc**:

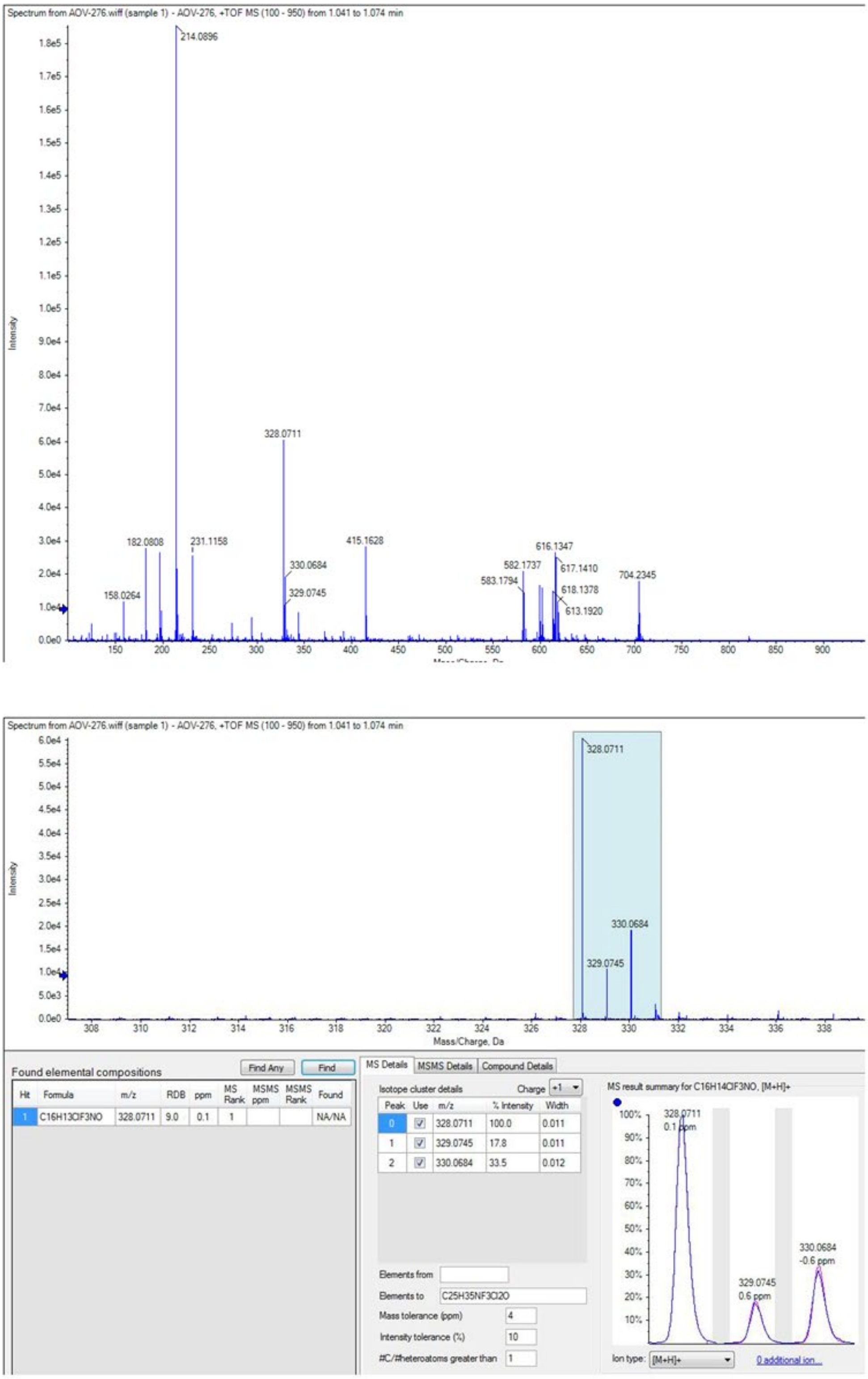

^{1}H (400 MHz) and ^{19}F (470 MHz) NMR of crude **3dd** in $CDCl_3$

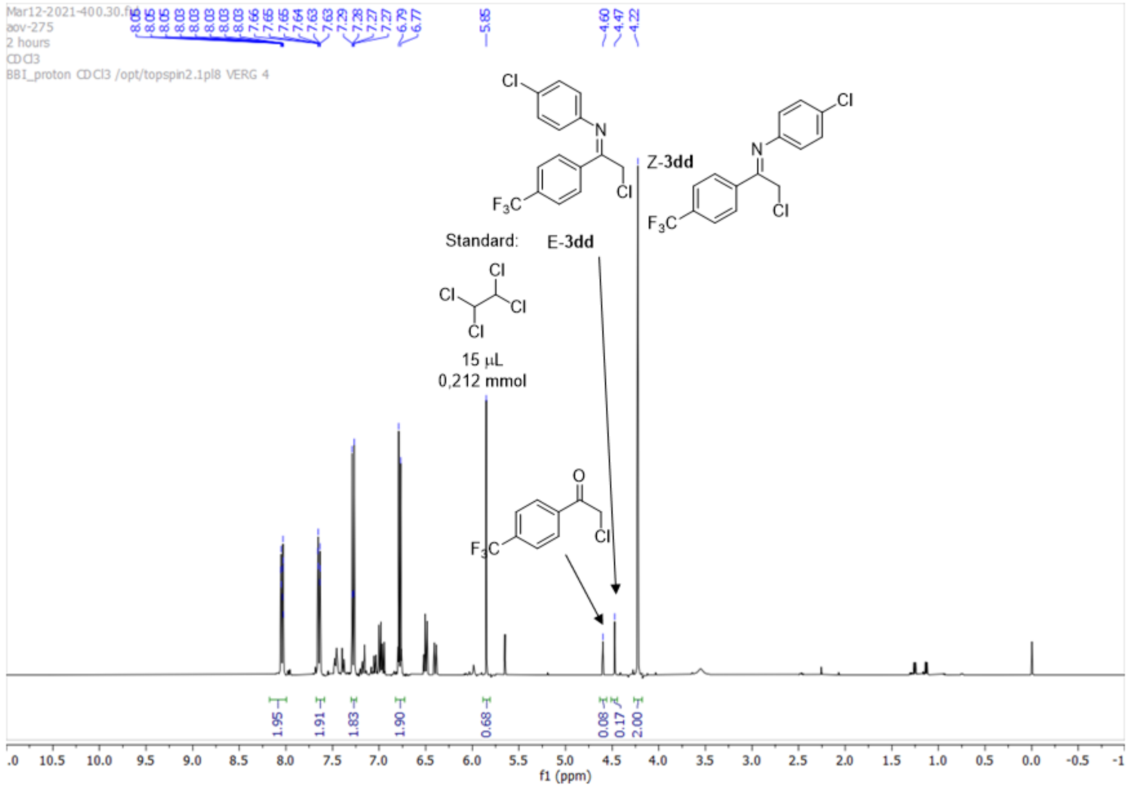

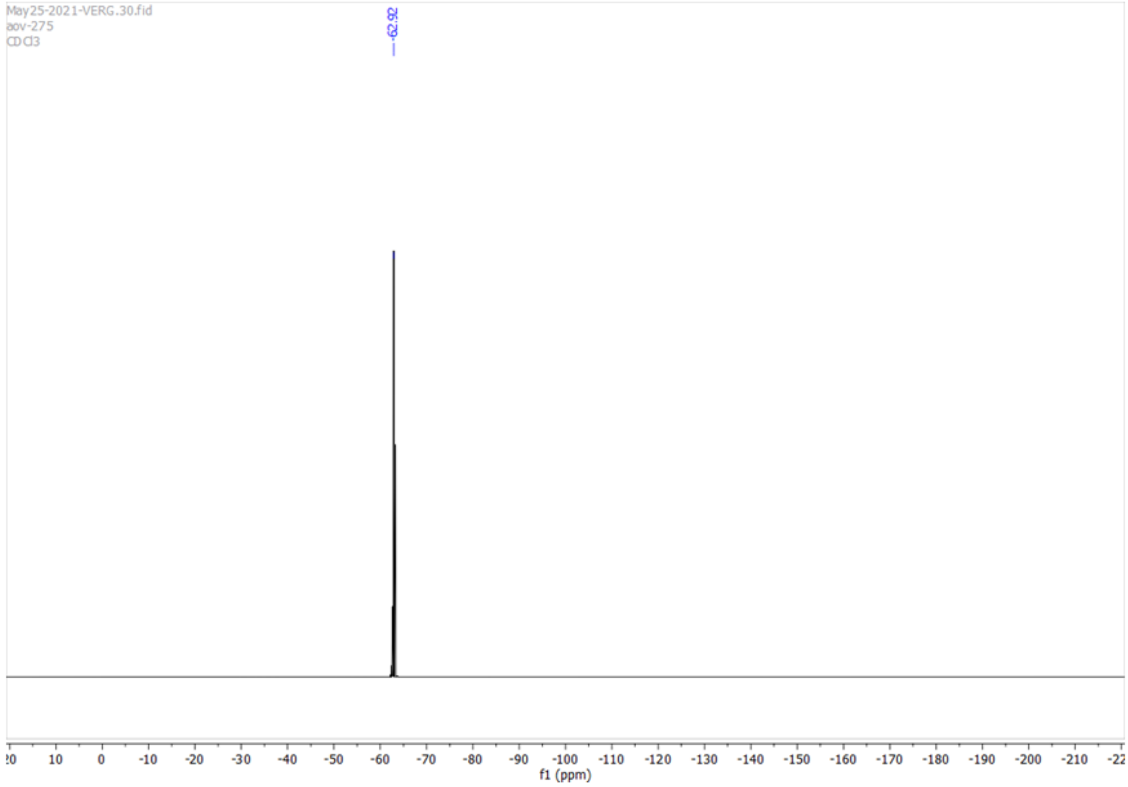

$^{13}C\{^1H\}$ (100 MHz) NMR of crude **3dd** in $CDCl_3$

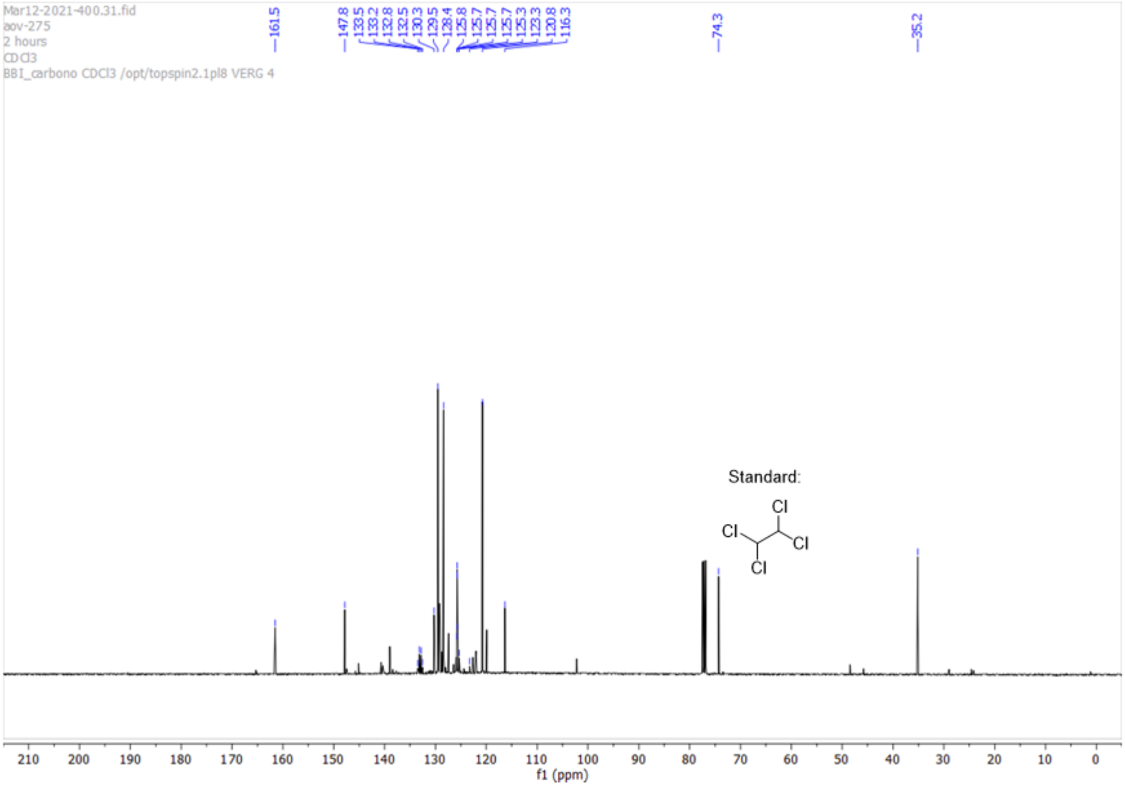

HRMS (+ESI) of crude **3dd**:

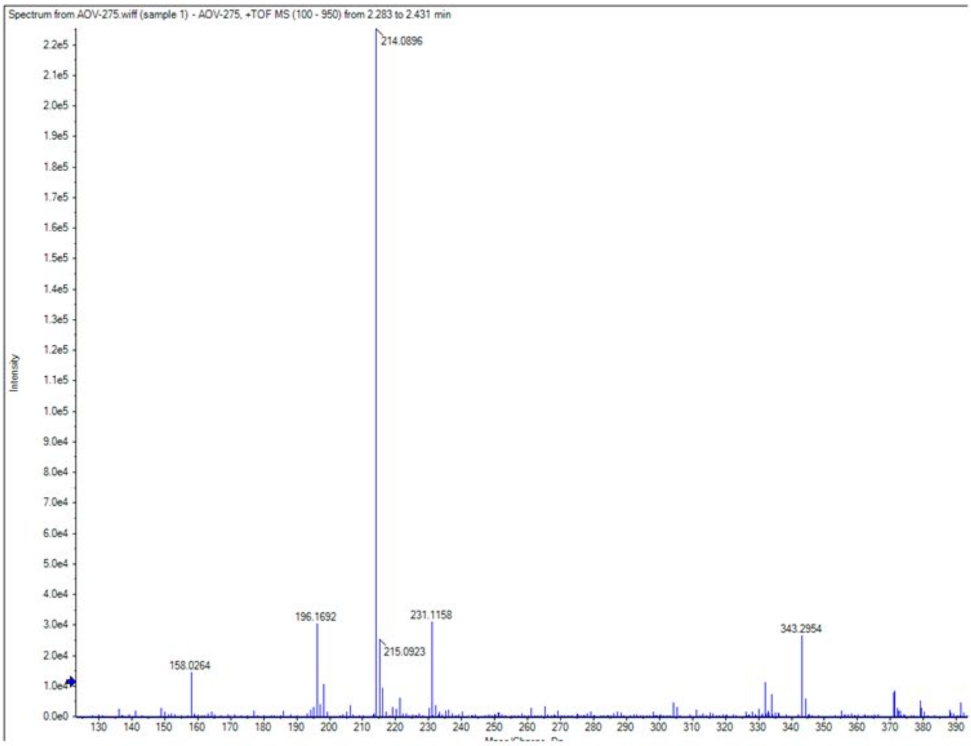

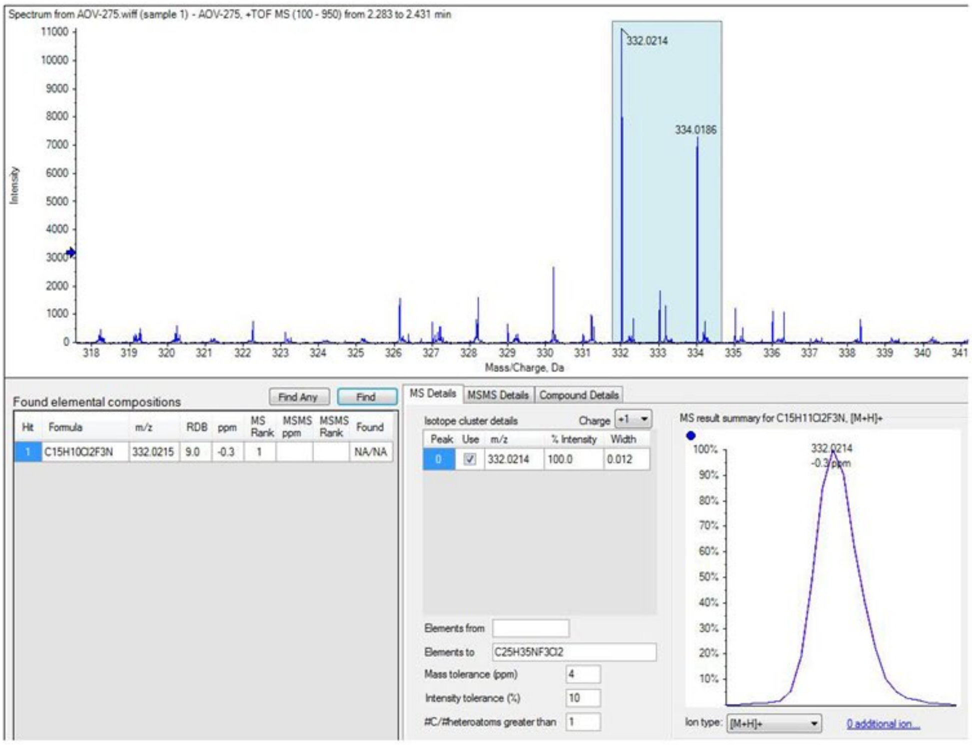

^{1}H (500 MHz) and $^{13}C\{^{1}H\}$ (125 MHz) NMR of crude **3eb** in $CDCl_3$

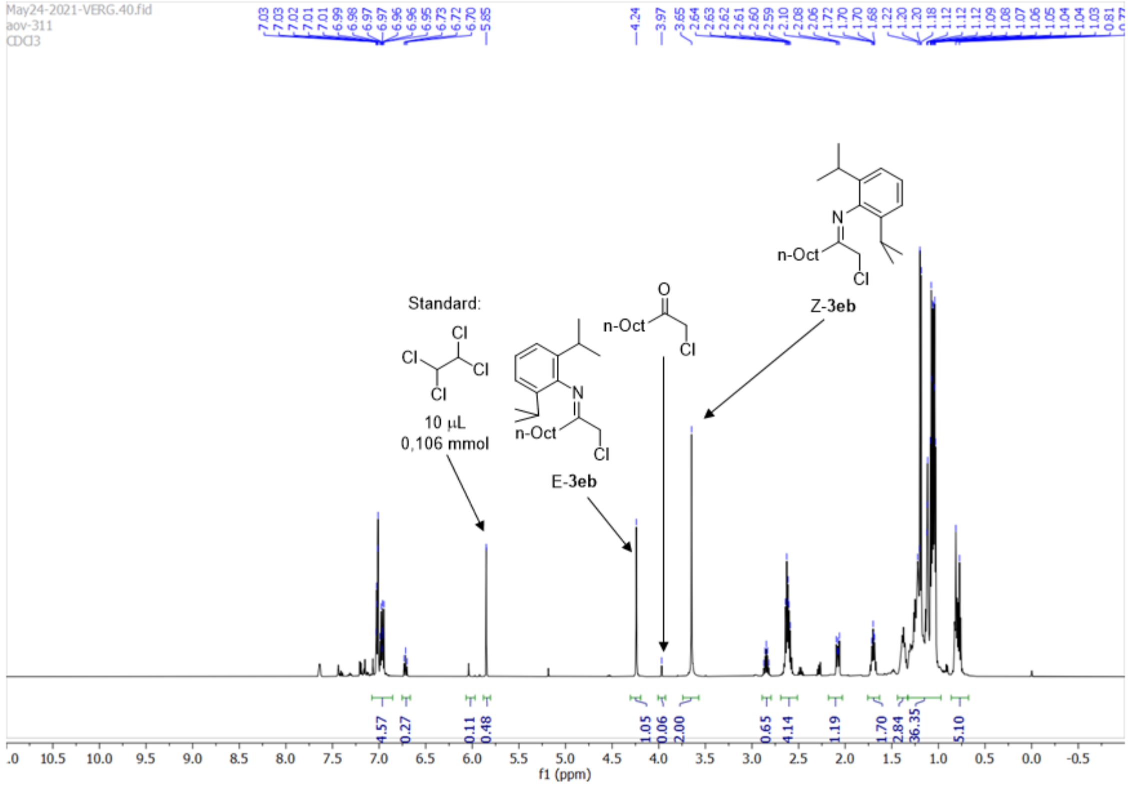

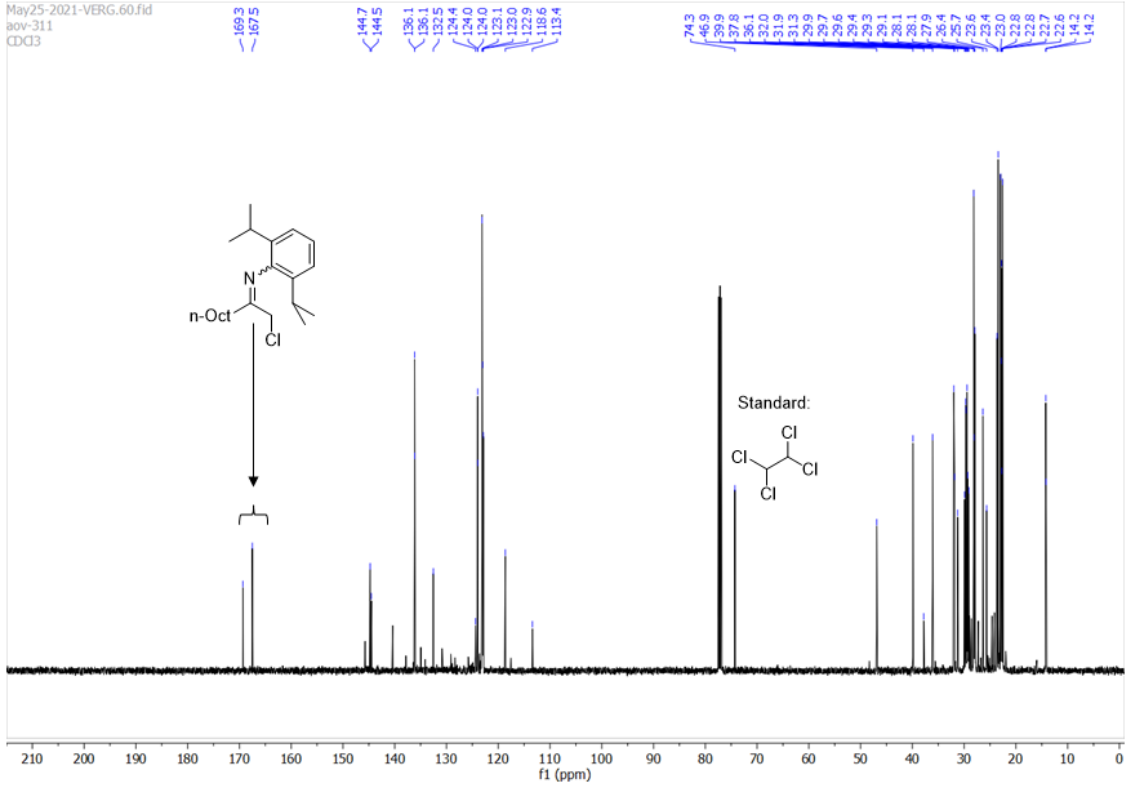

HSQC (500 MHz) NMR of crude **3eb** in $CDCl_3$

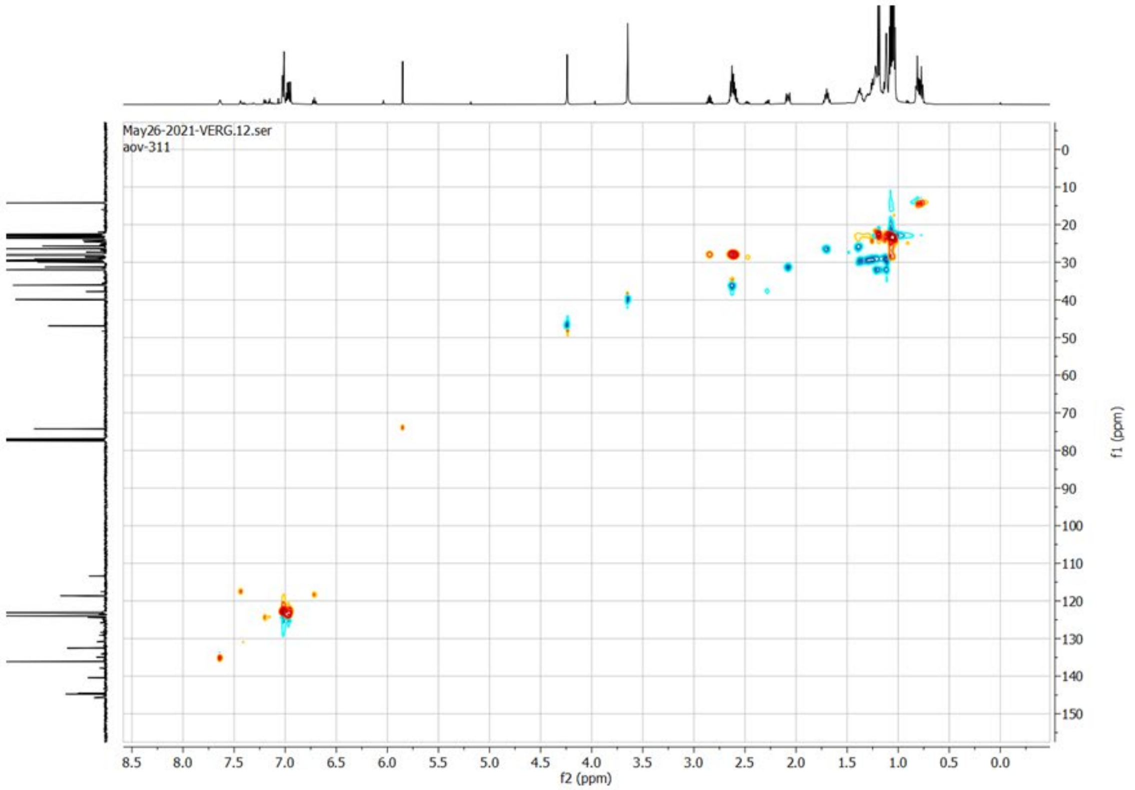

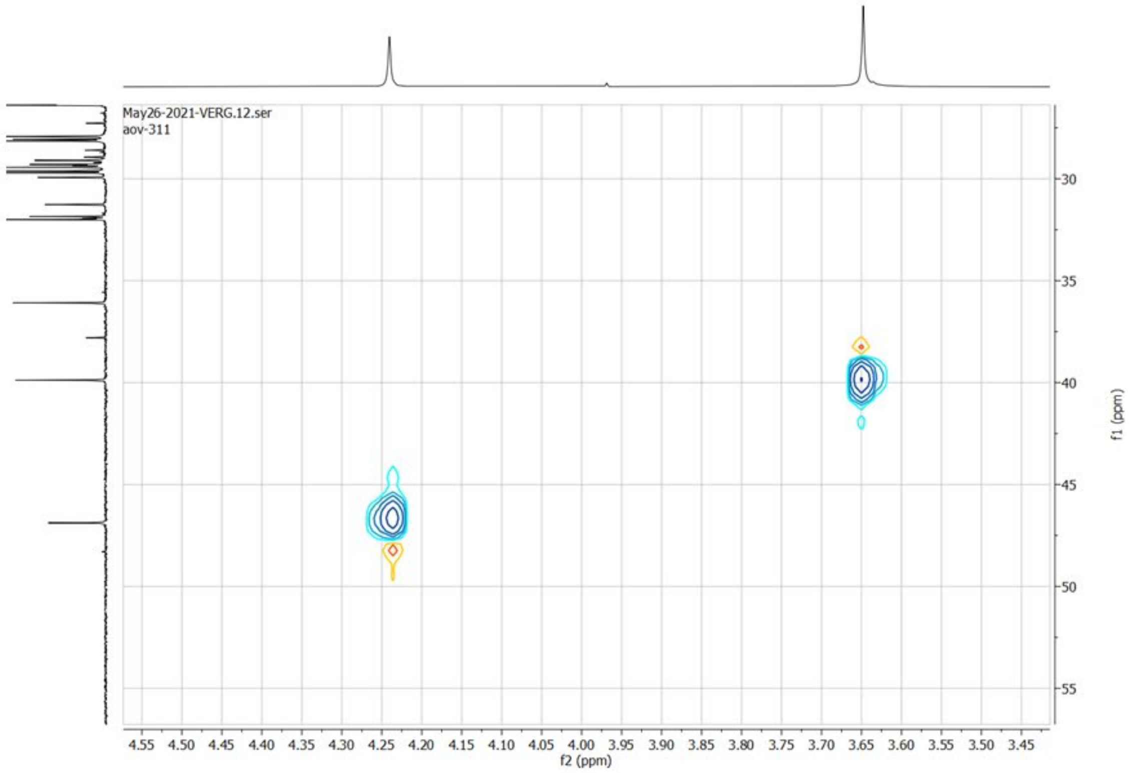

NOESY (500 MHz) NMR of crude **3eb** in $CDCl_3$

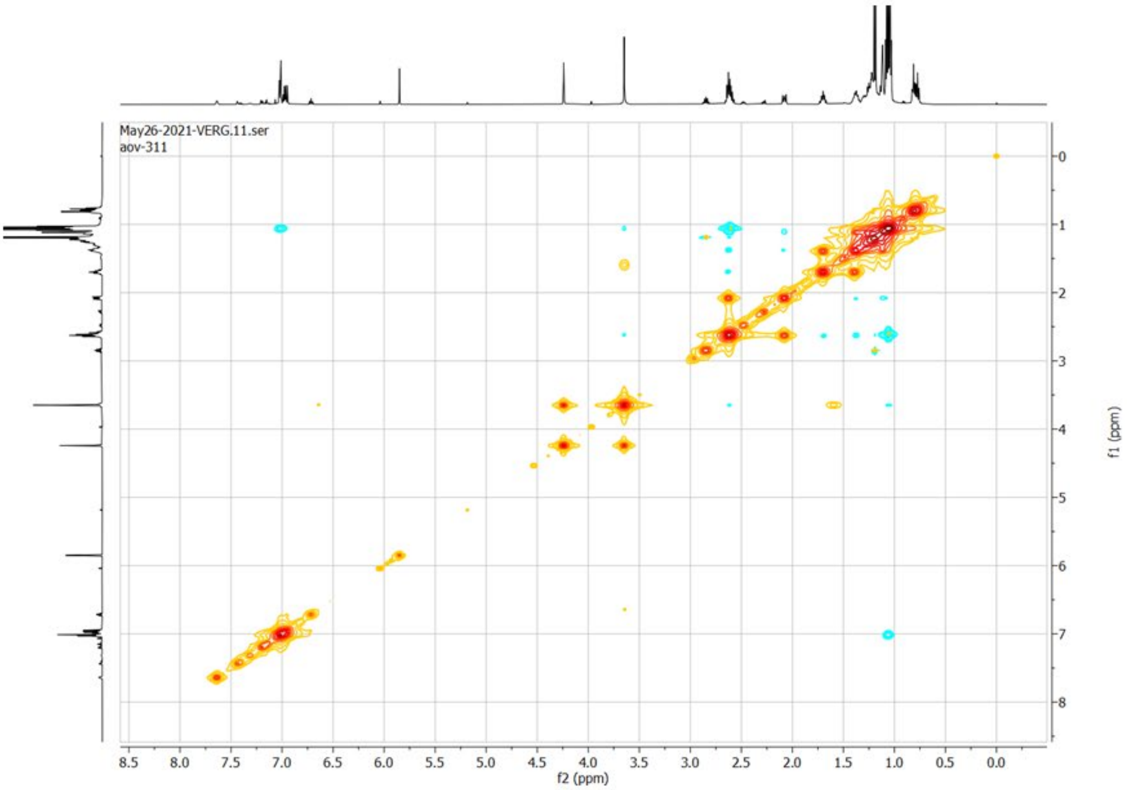

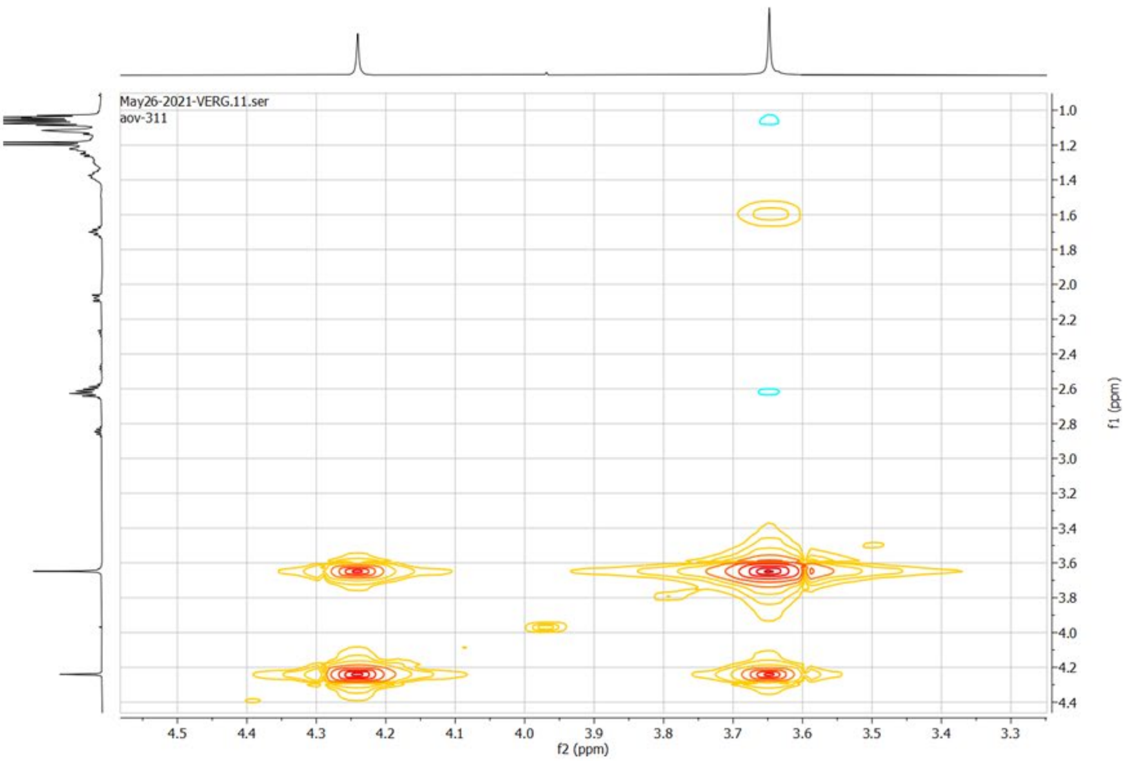

HRMS (+ESI) of crude **3eb**:

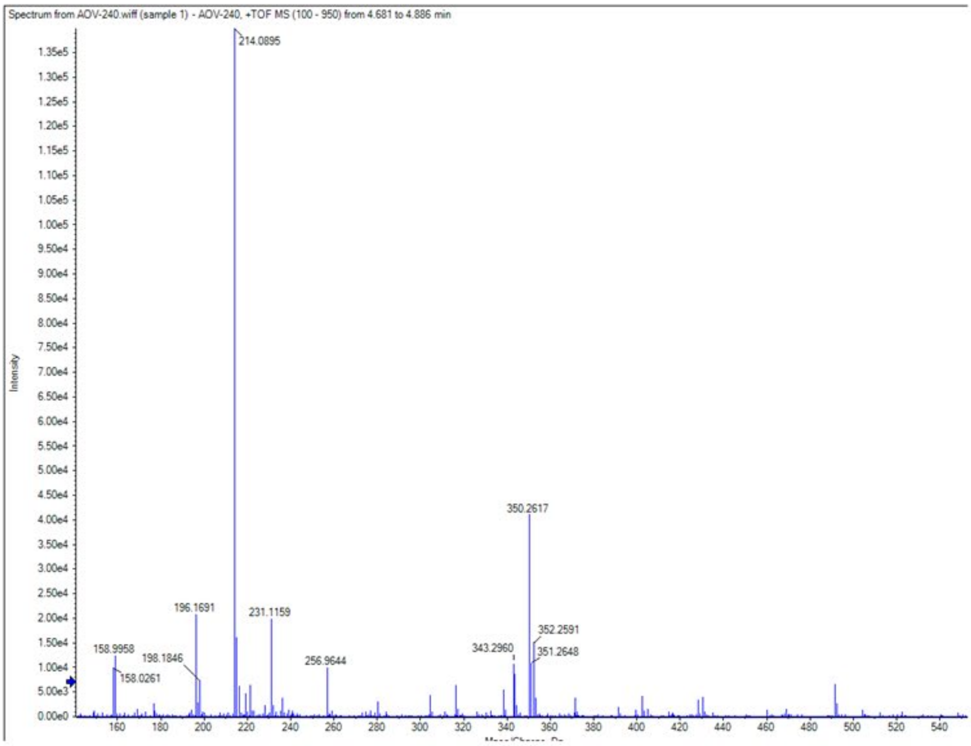

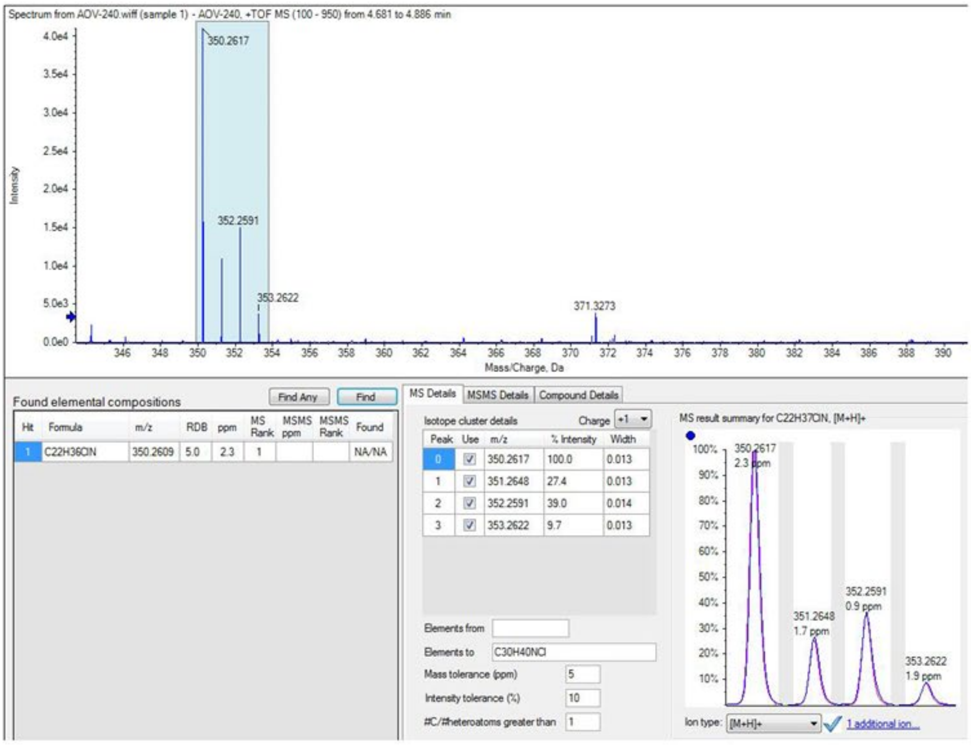

^1H (500 MHz) and $^{13}C\{^1H\}$ (125 MHz) NMRof crude **6aa** in $CDCl_3$

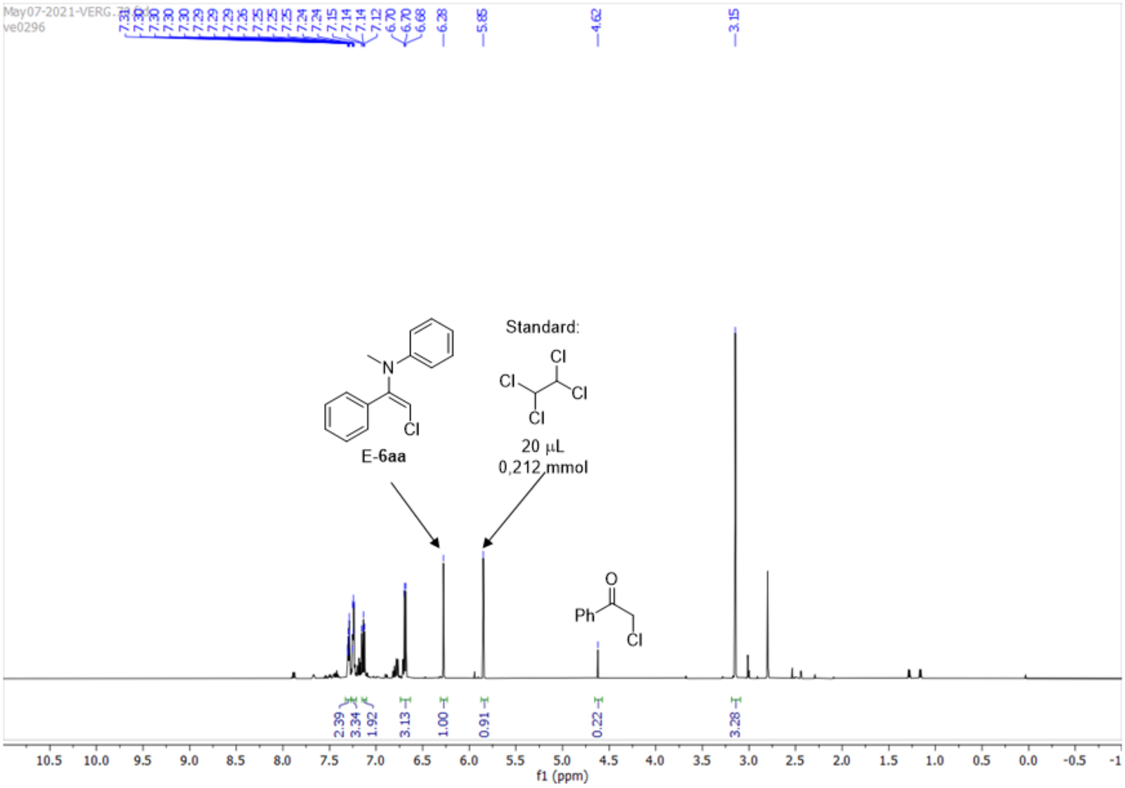

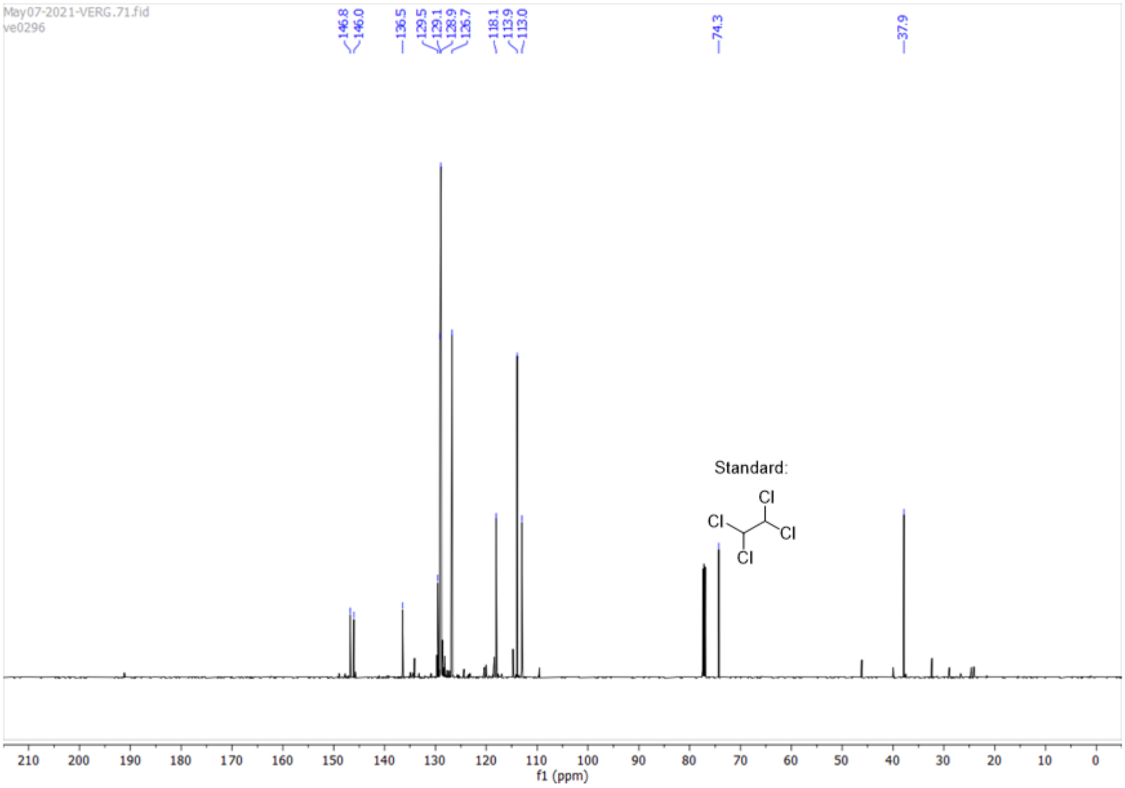

HSQC (500 MHz) NMR of crude **6aa** in $CDCl_3$

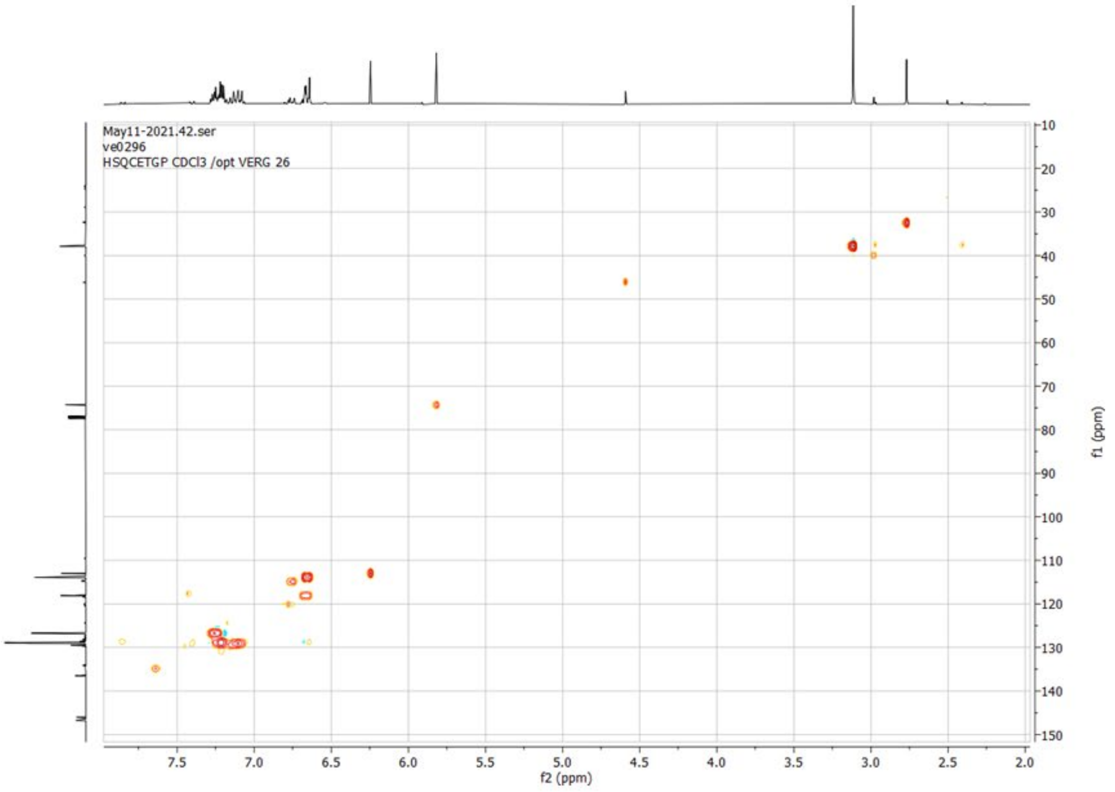

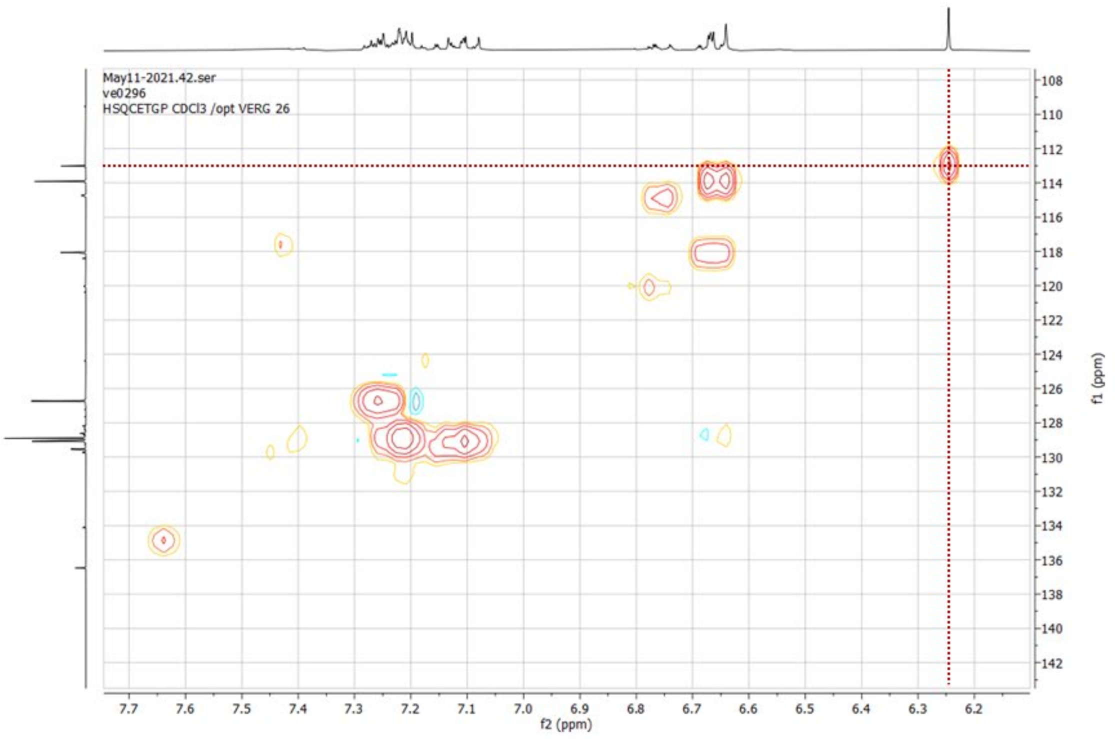

HRMS (+ESI) of crude **6aa**:

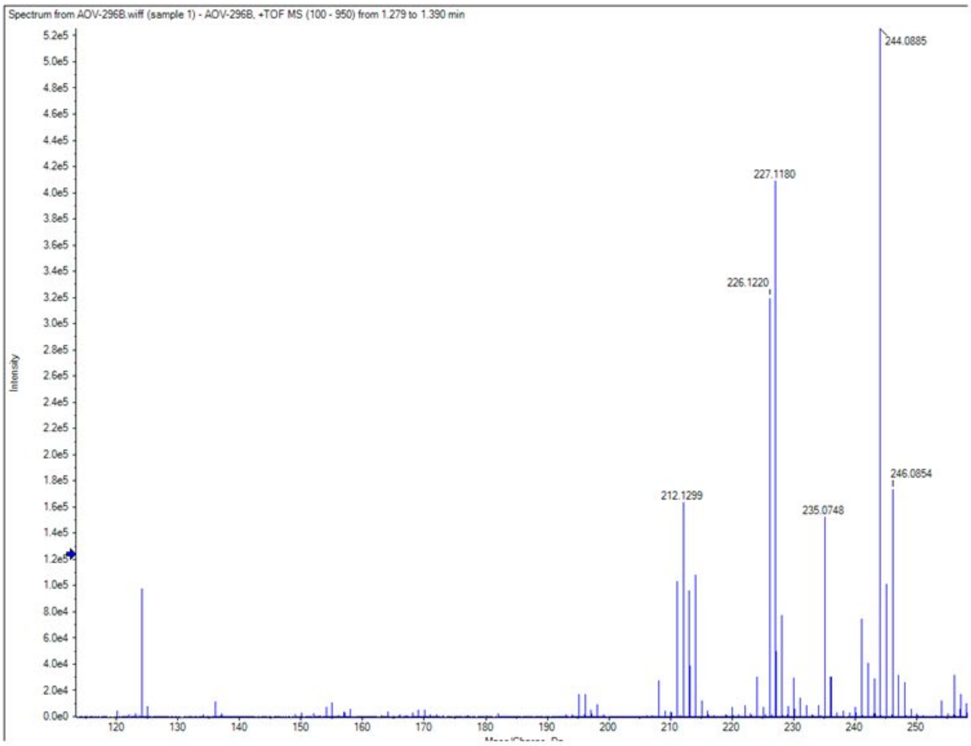

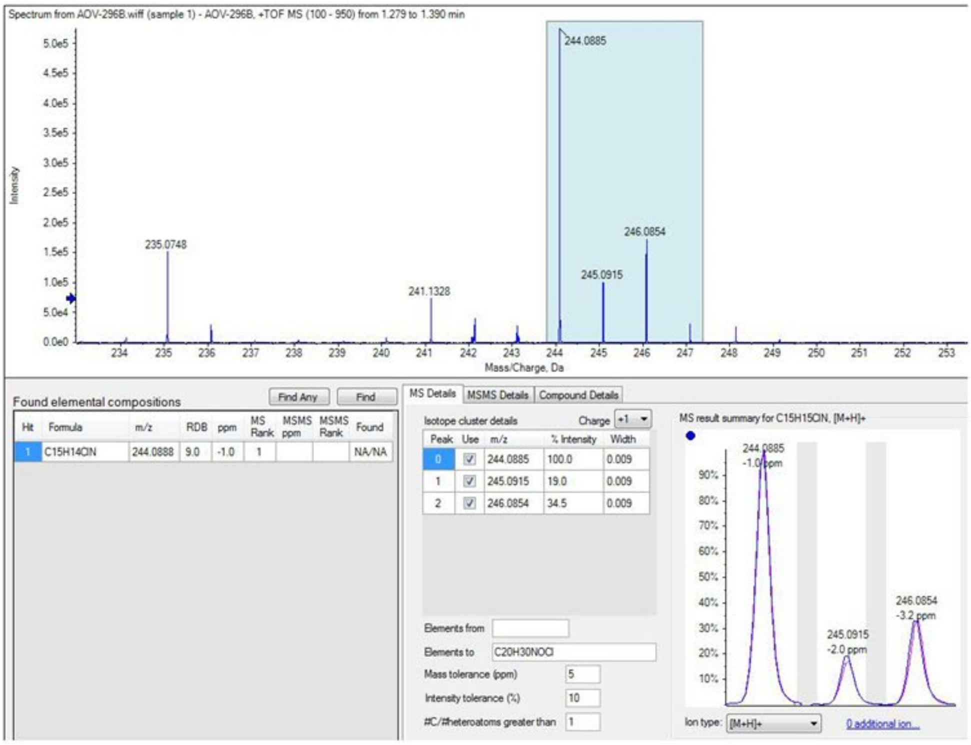

^1H (500 MHz) and $^{13}C\{^1H\}$ (125 MHz) NMR of crude **6ab** in $CDCl_3$

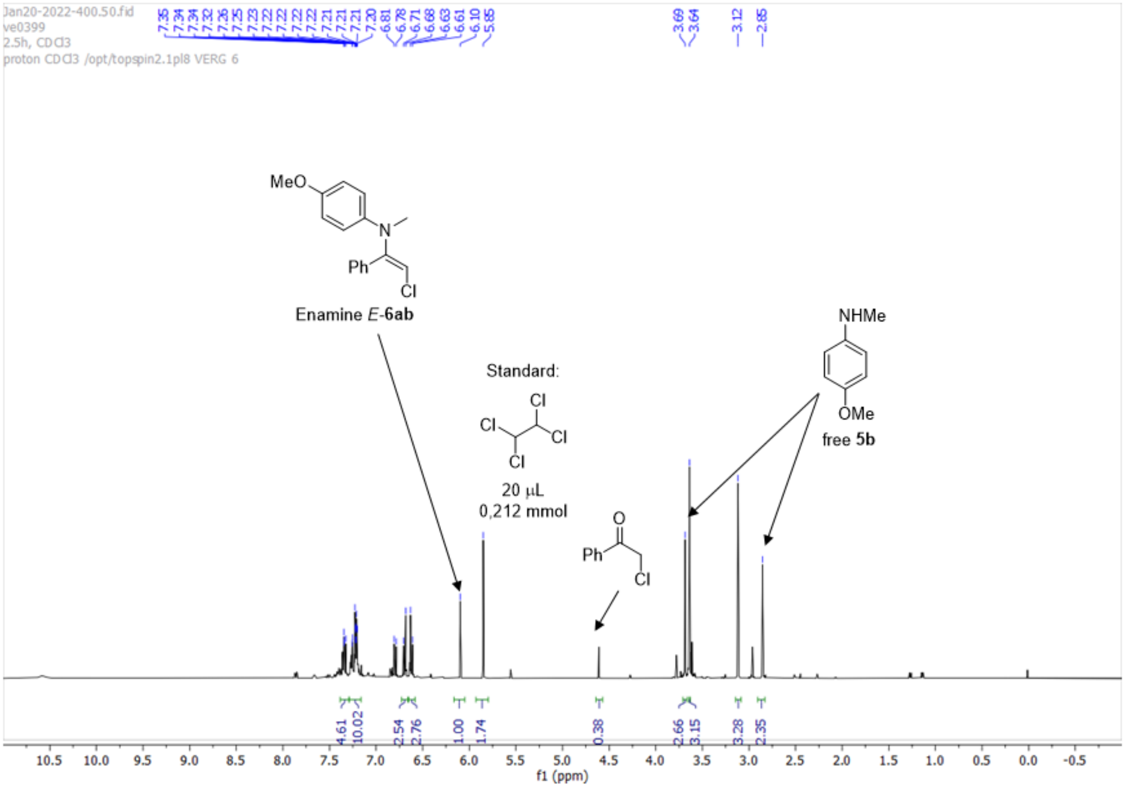

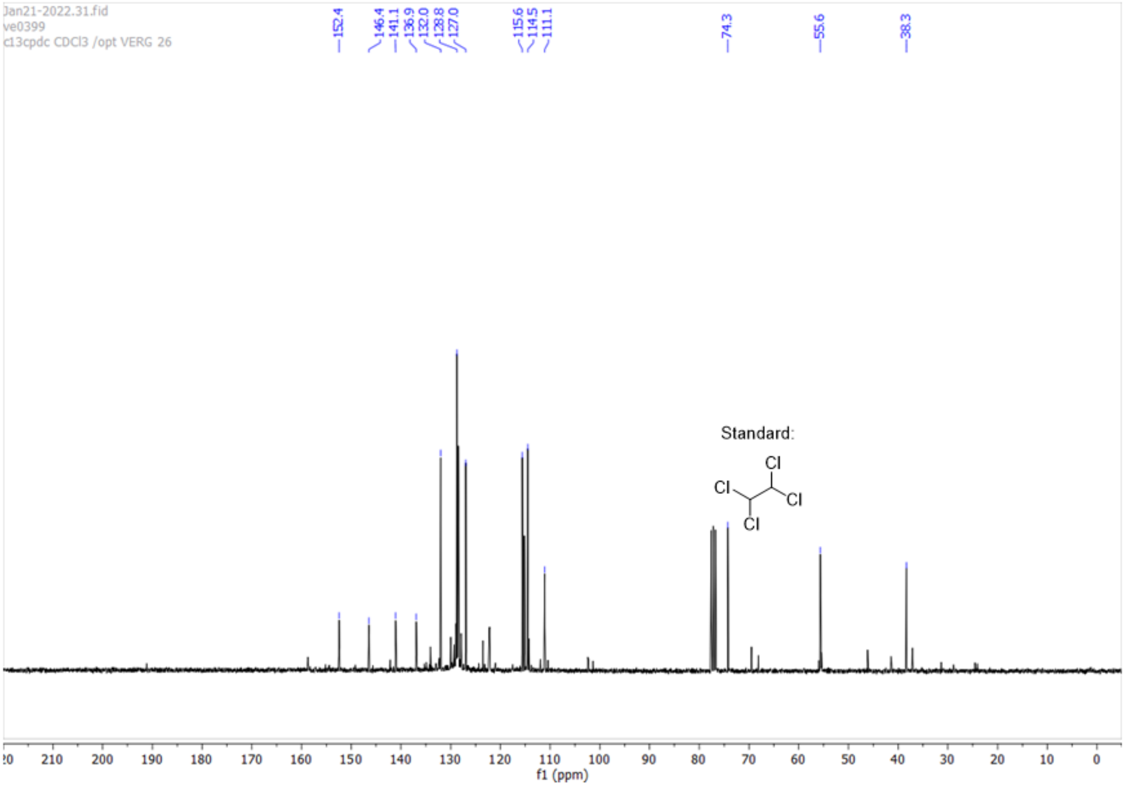

HSQC (500 MHz) of crude **6ab** in $CDCl_3$

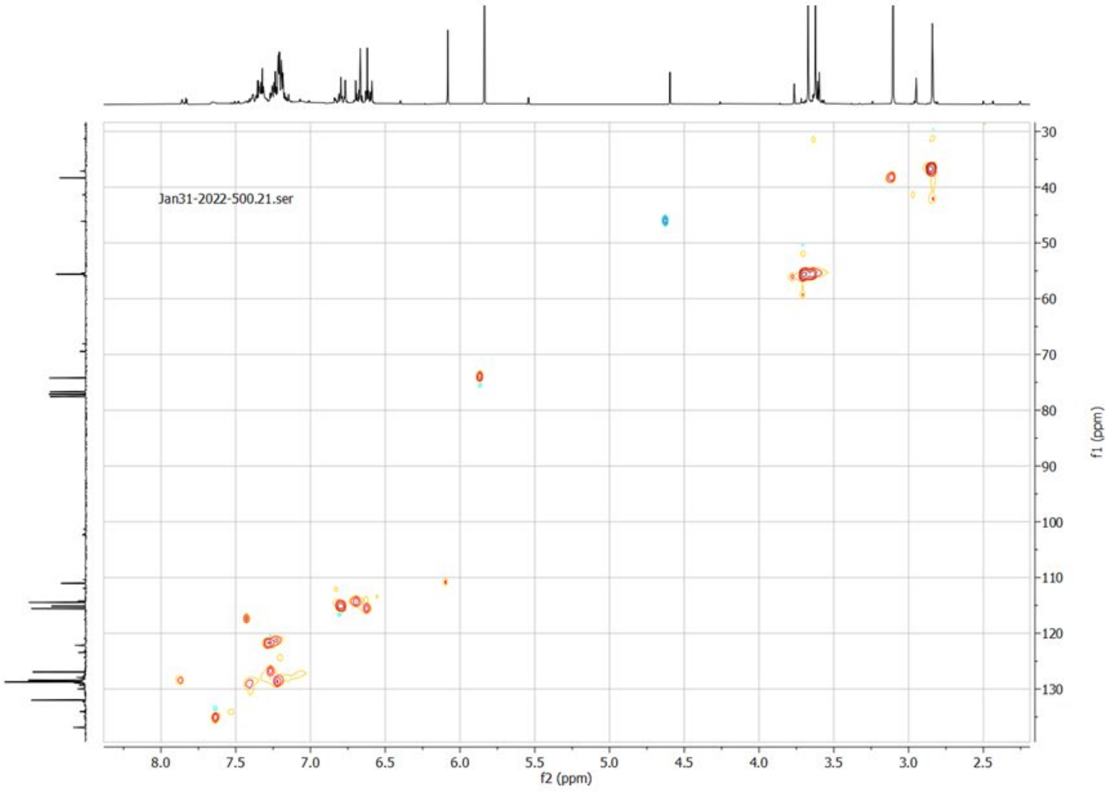

HRMS (+ESI) of crude **6ab**:

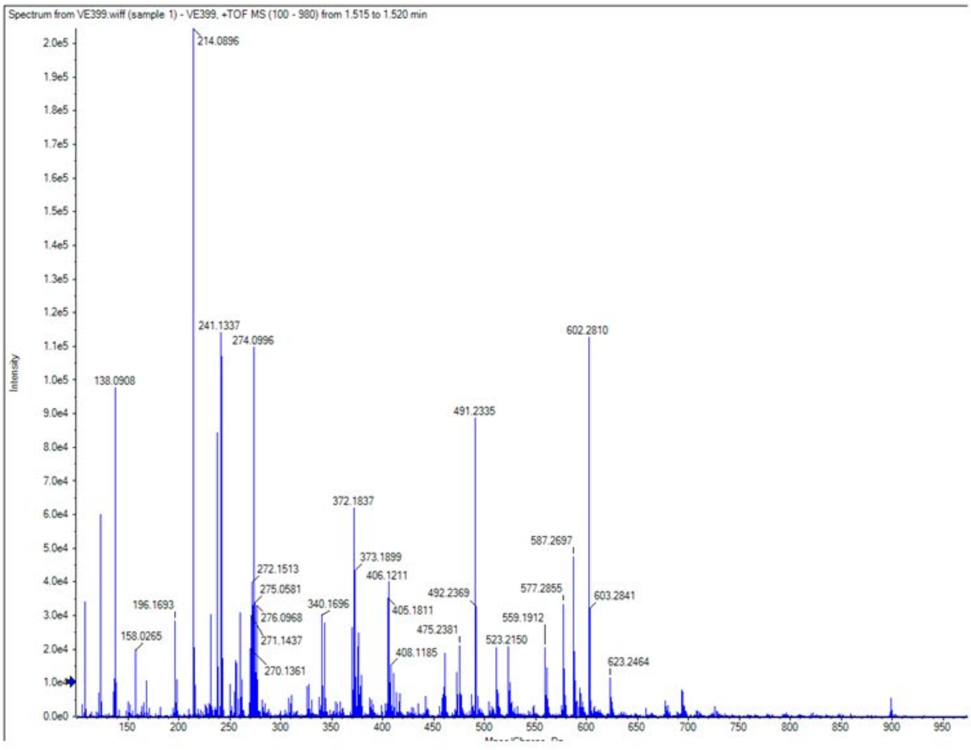

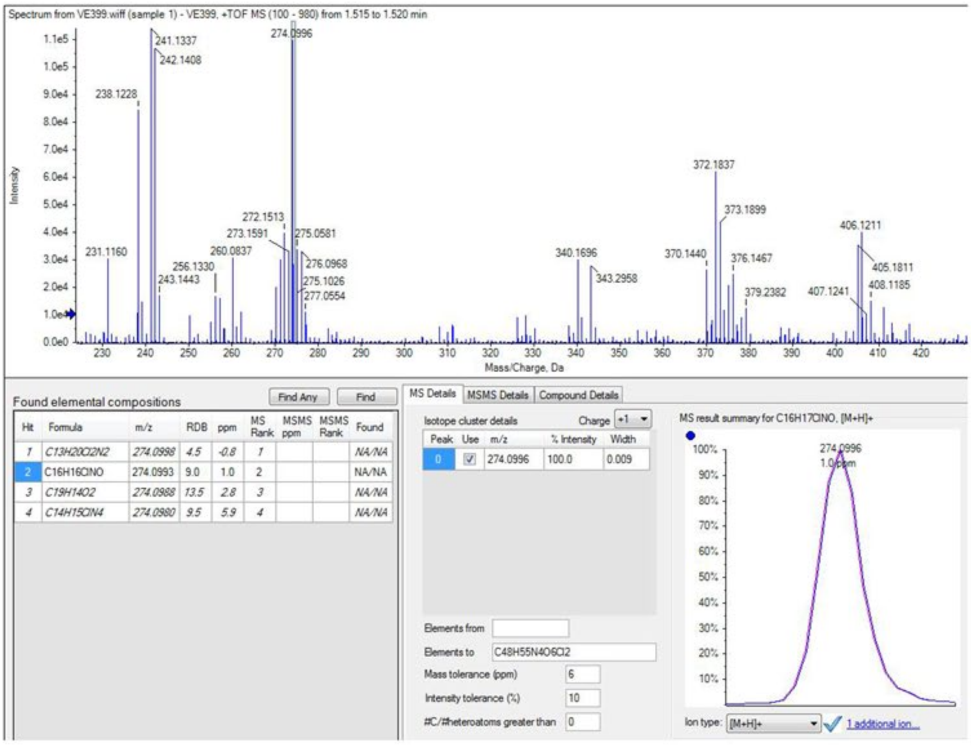

^{1}H (500 MHz) and $^{13}C\{^{1}H\}$ (125 MHz) NMR of crude **6ac** in $CDCl_3$

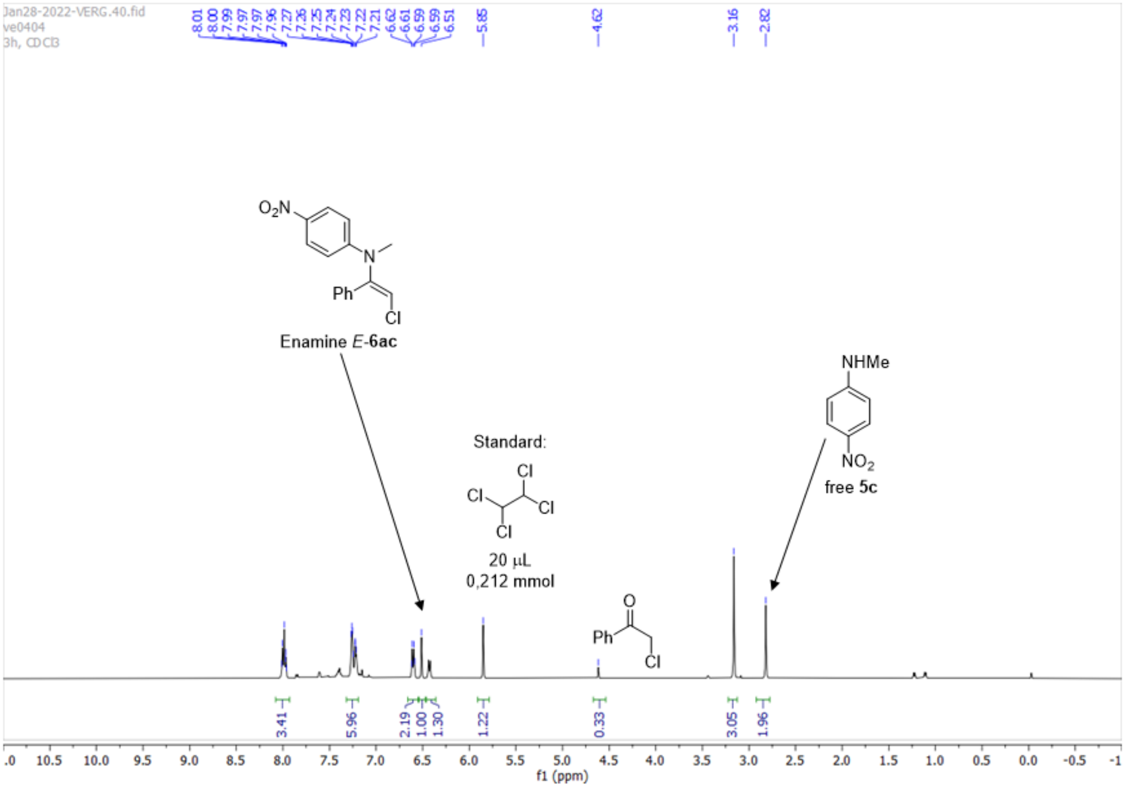

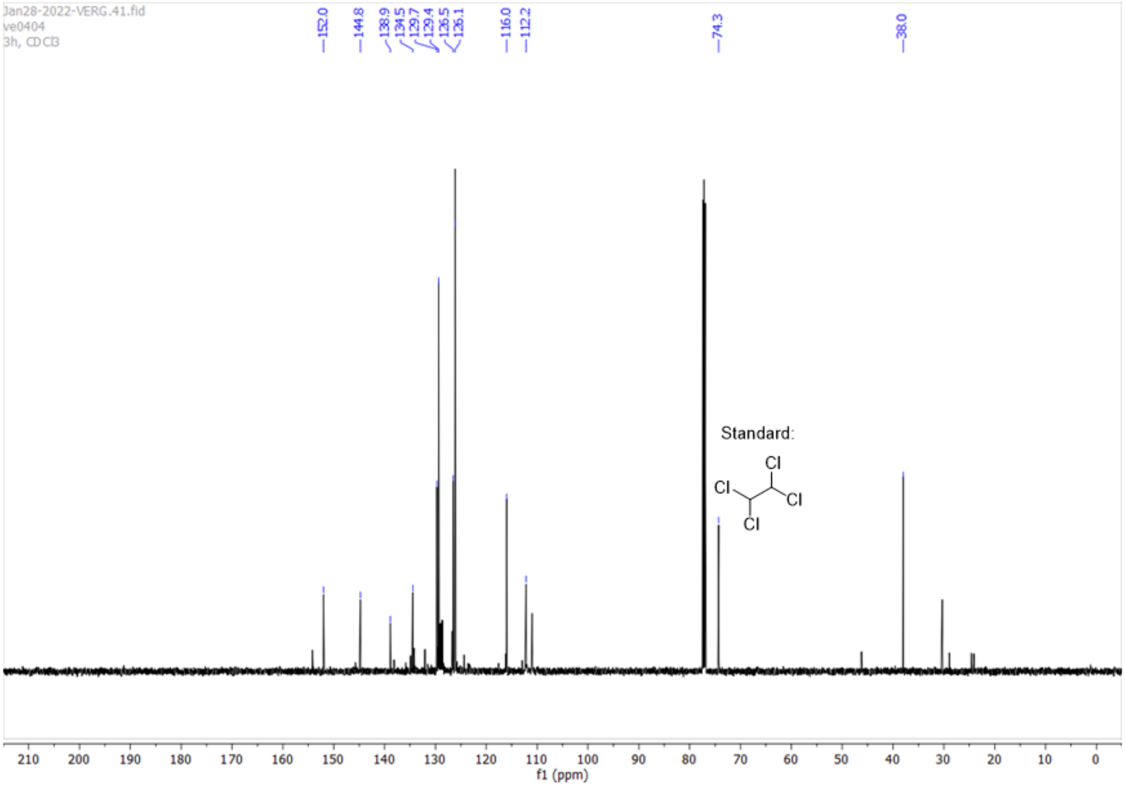

HSQC (500 MHz) of crude **6ac** in $CDCl_3$

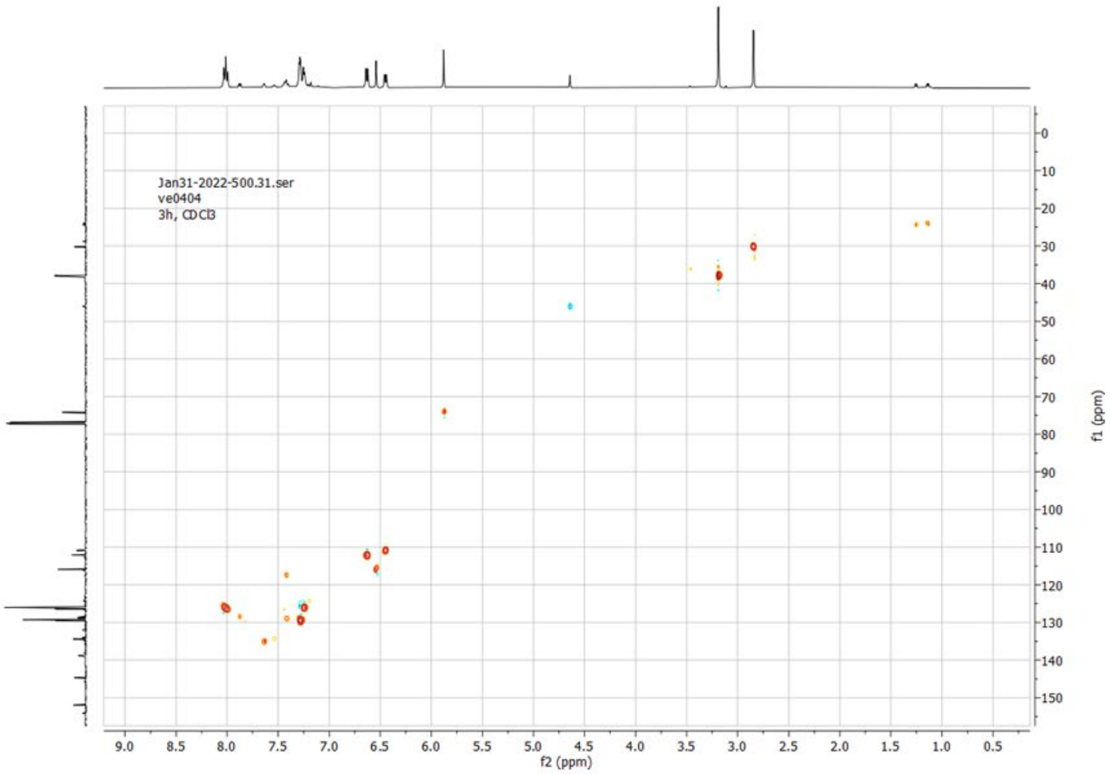

HRMS (+ESI) of crude **6ac**:

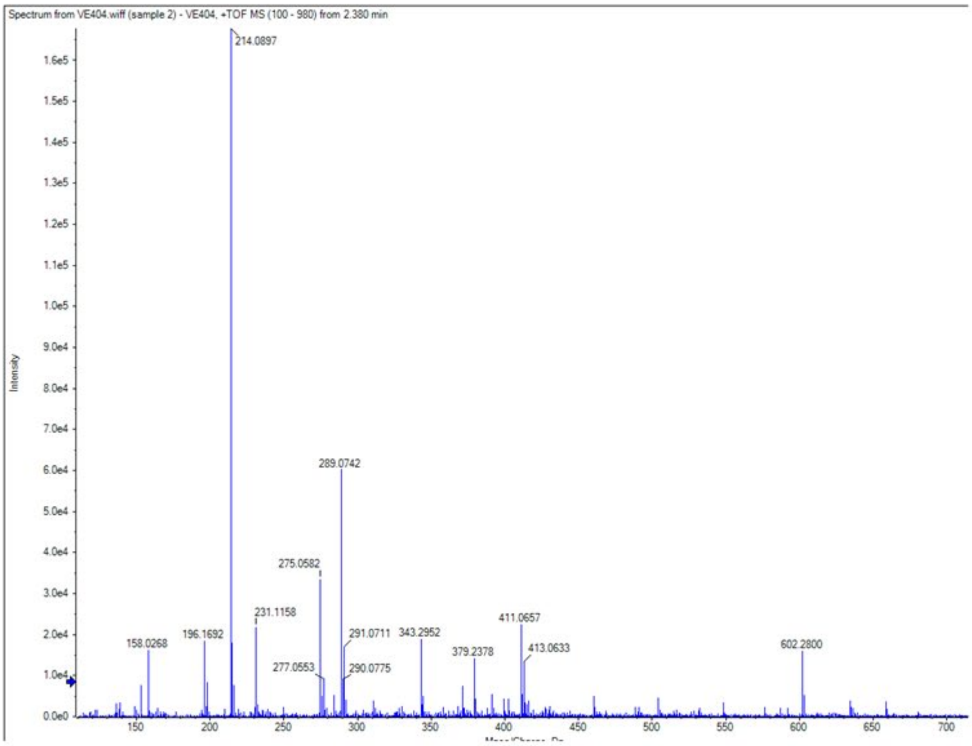

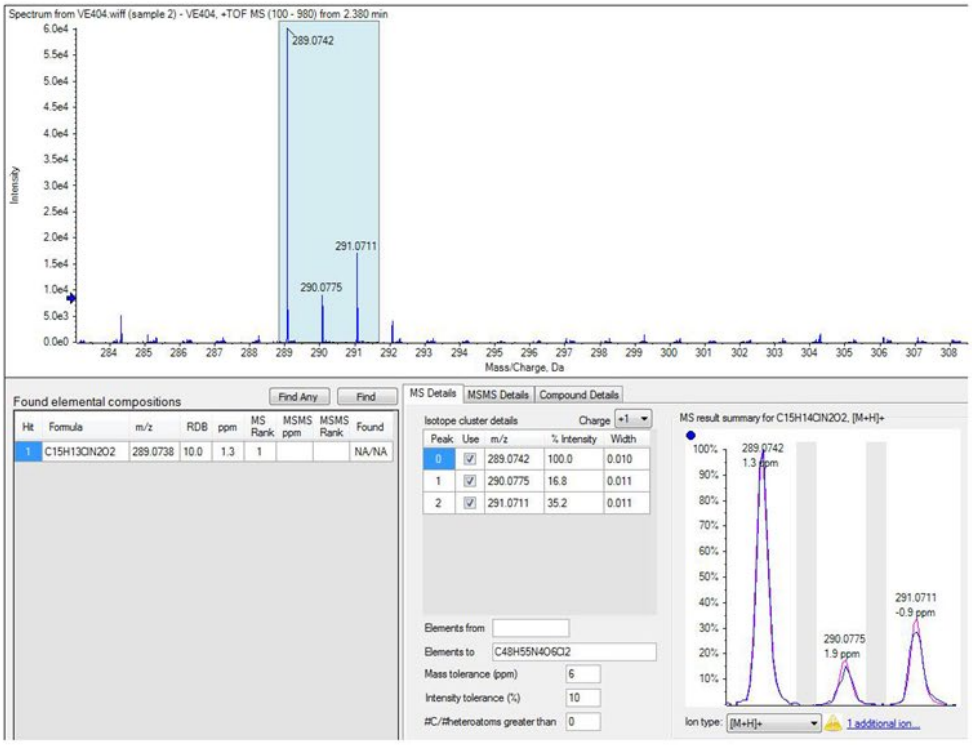

^1H (500 MHz) and $^{13}C\{^1H\}$ (125 MHz) NMR of crude **6ad** in $CDCl_3$

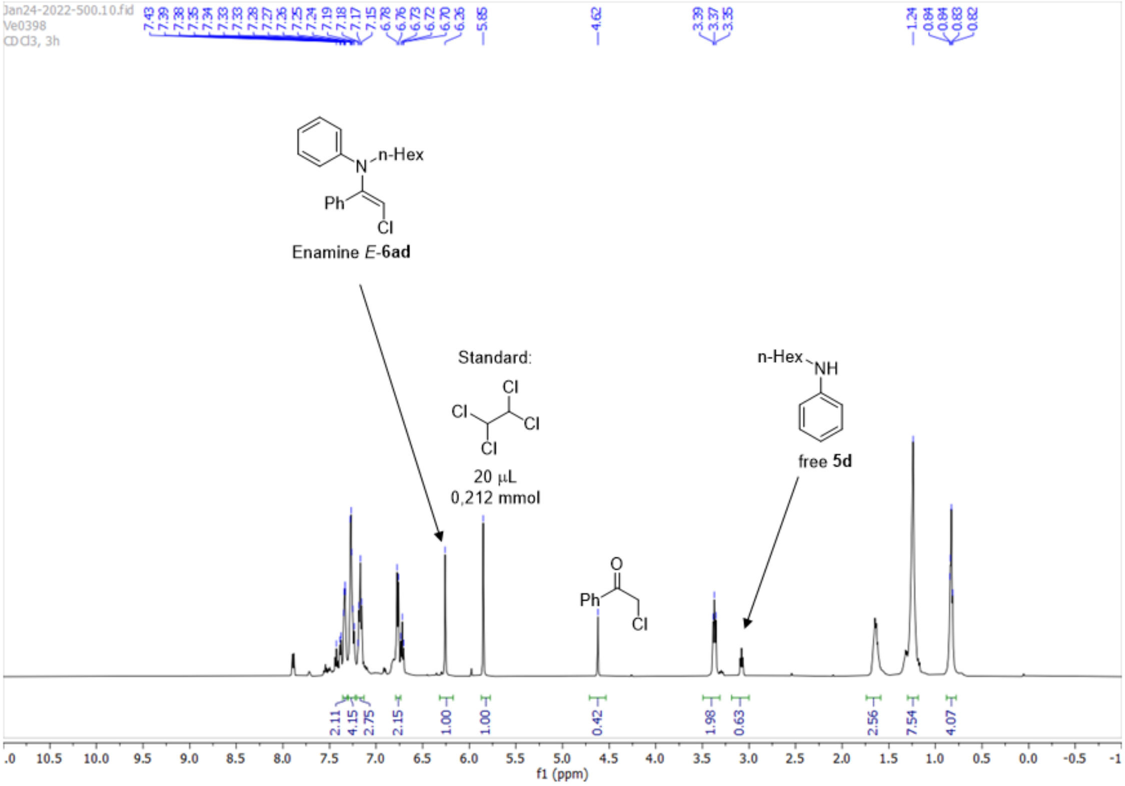

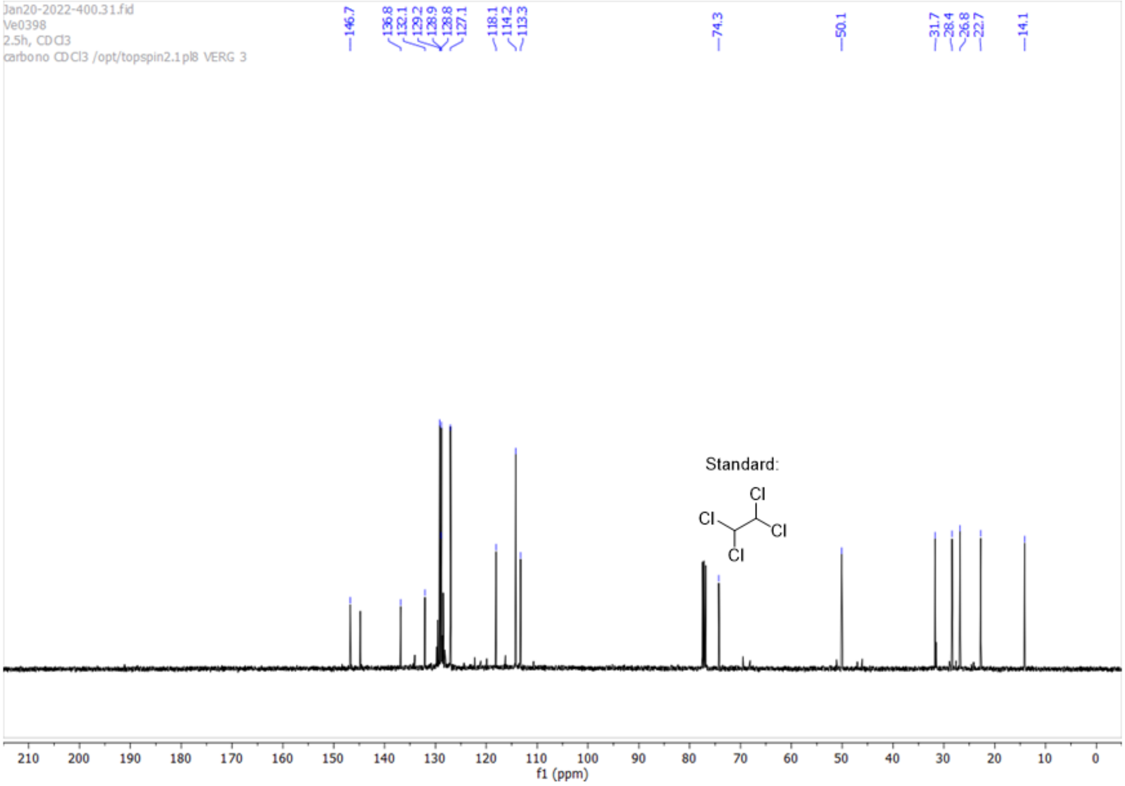

HSQC (500 MHz) of crude **6ad** in $CDCl_3$

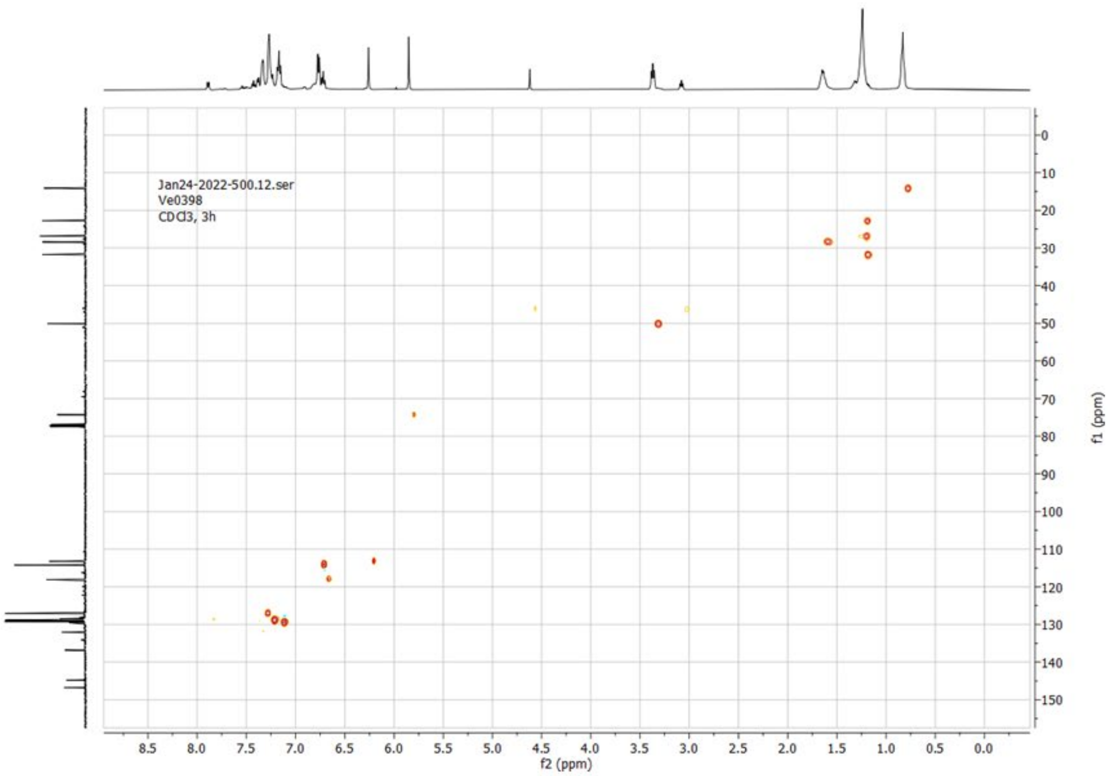

HRMS (+ESI) of crude **6ad**:

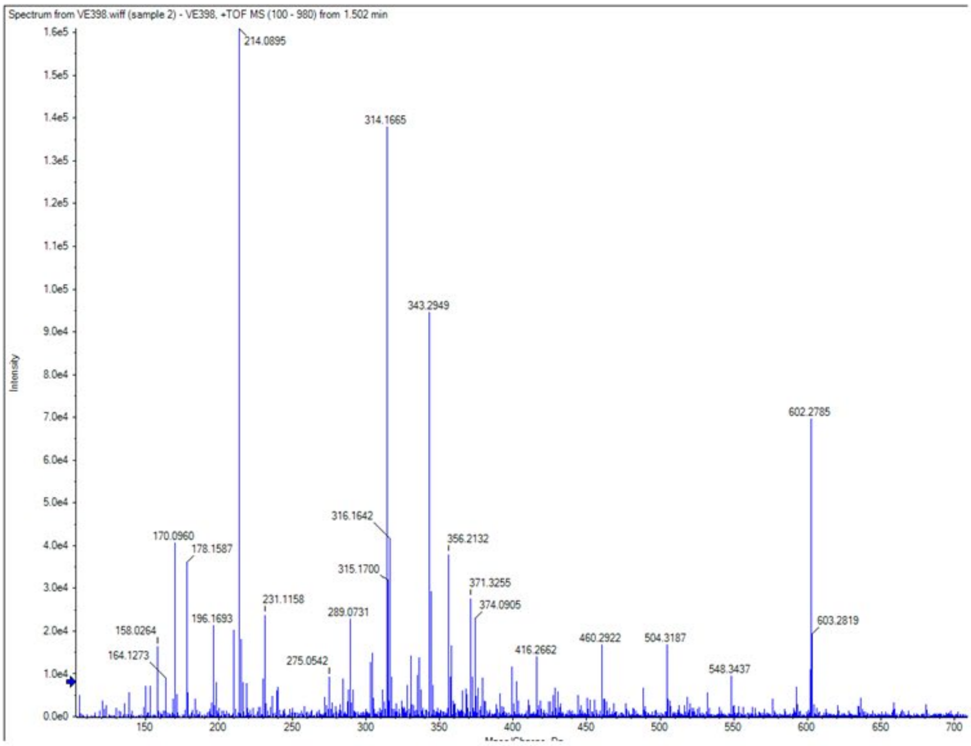

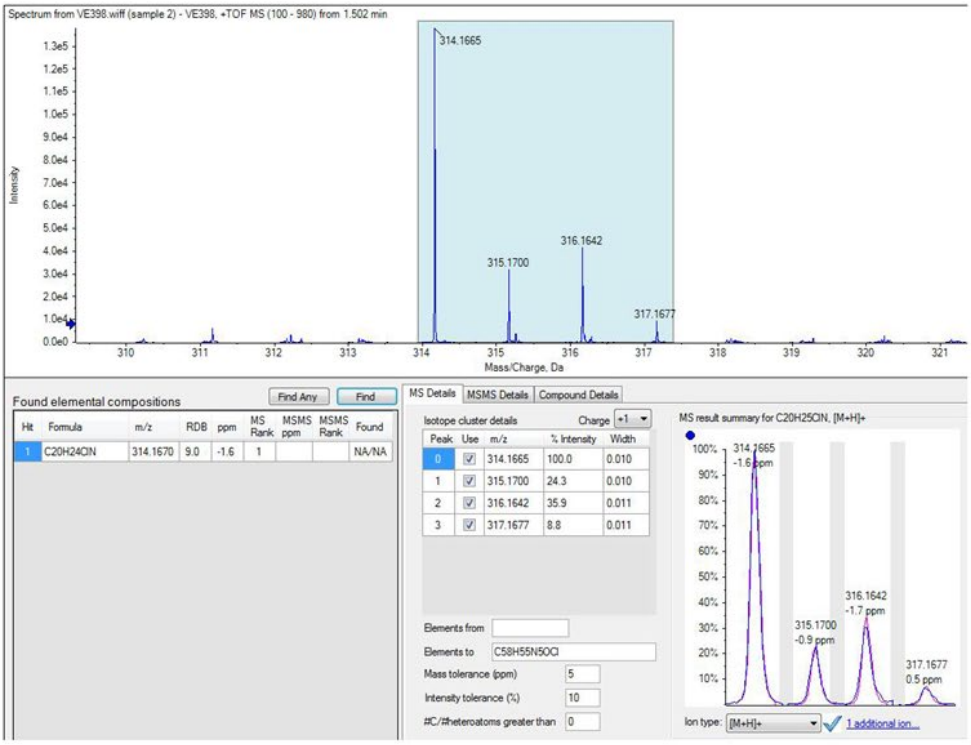

^{1}H (500 MHz) and ^{19}F (470 MHz) NMR of crude **6de** in $CDCl_3$

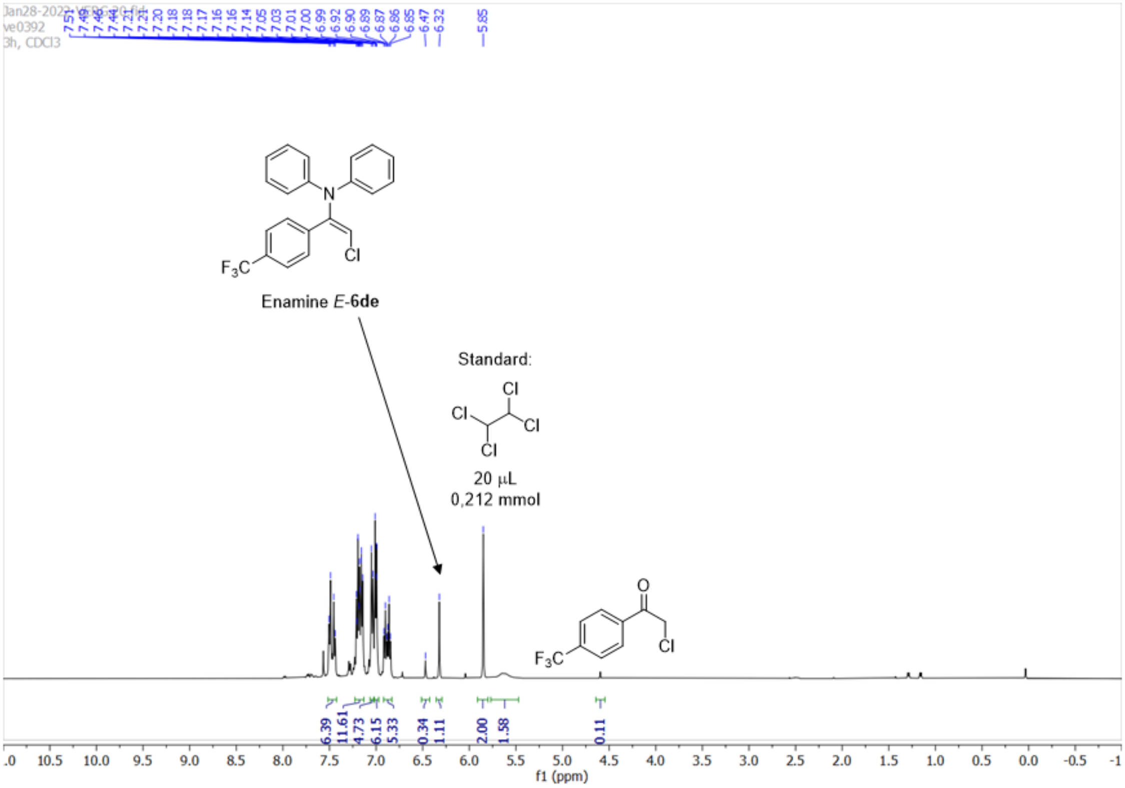

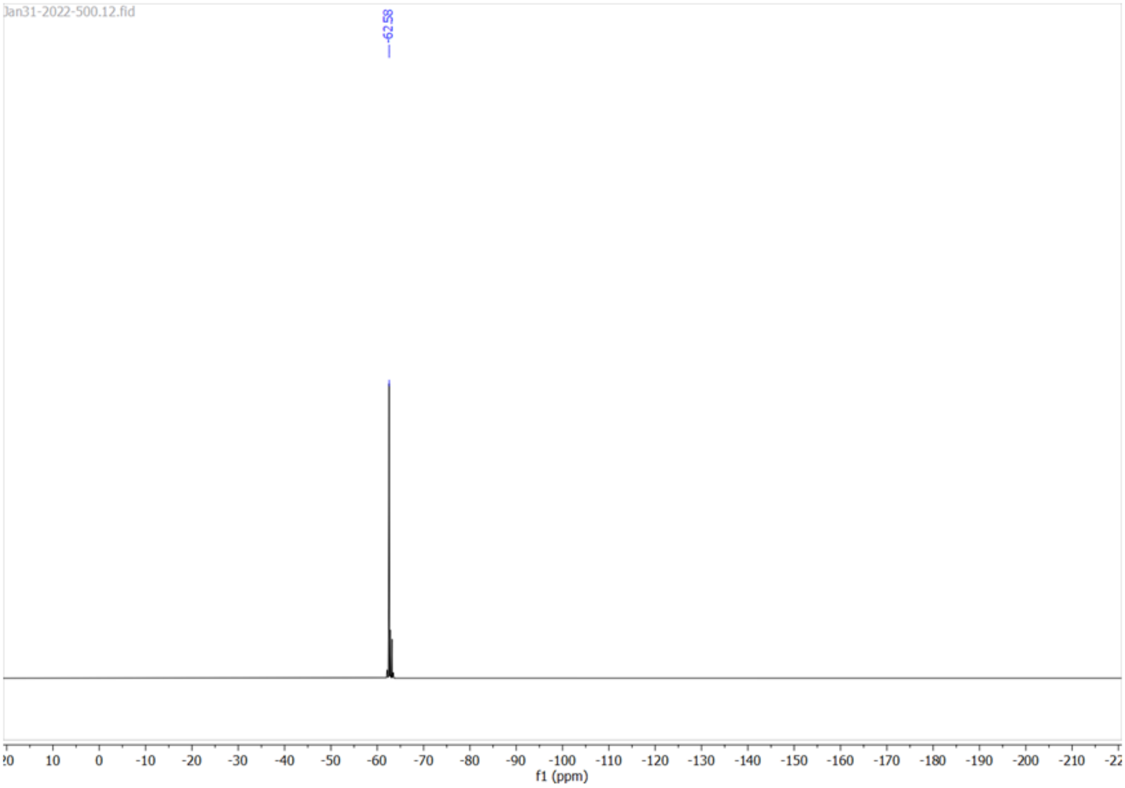

$^{13}C\{^{1}H\}$ (125MHz) of crude **6de** in $CDCl_3$

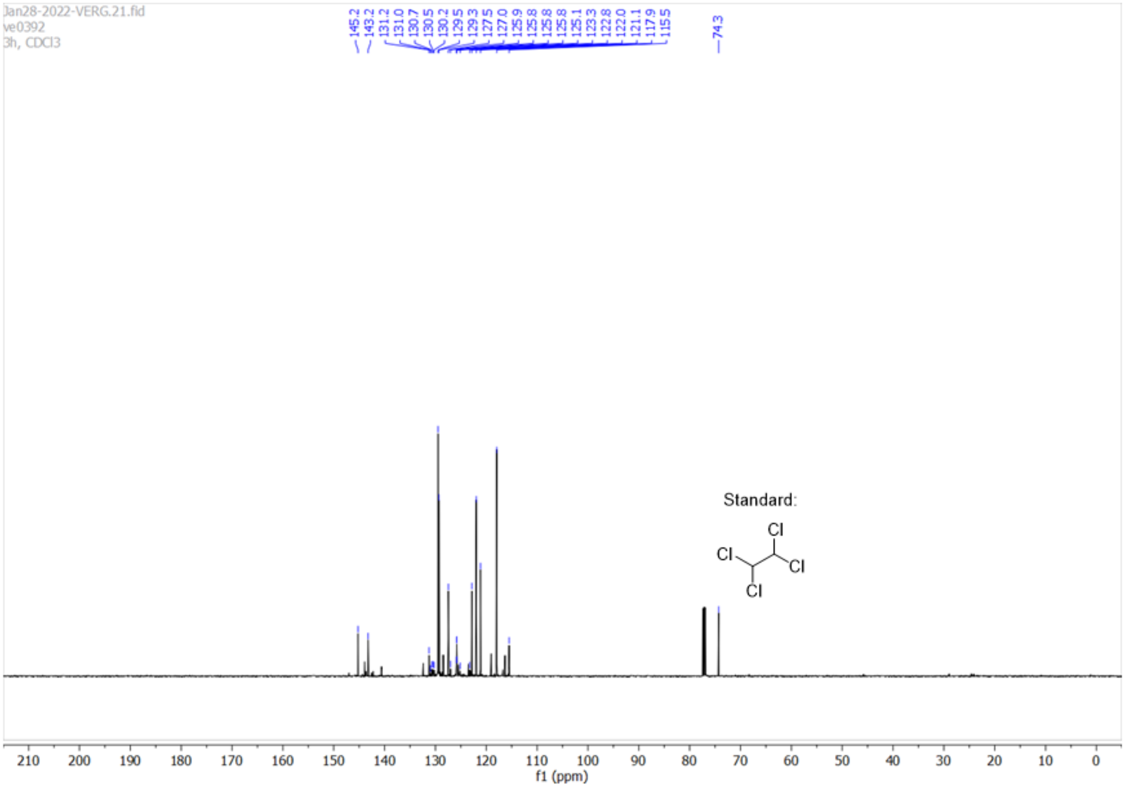

NOESY (500MHz) of crude **6de** in $CDCl_3$

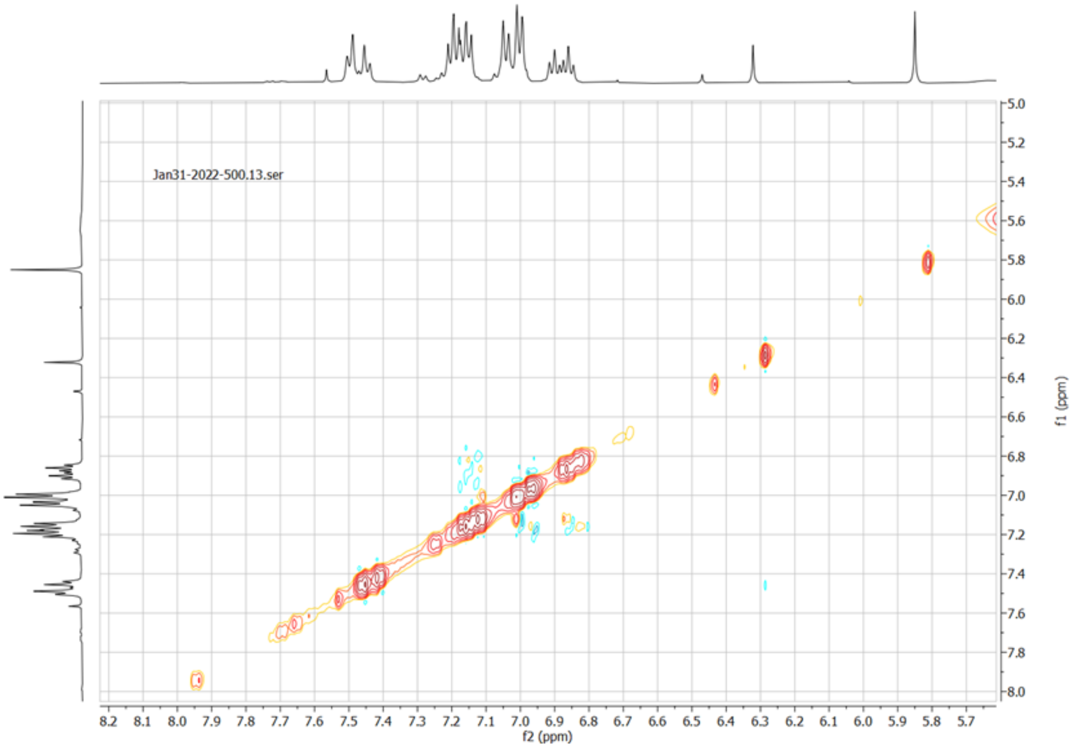

HRMS (+ESI) of crude **6de**:

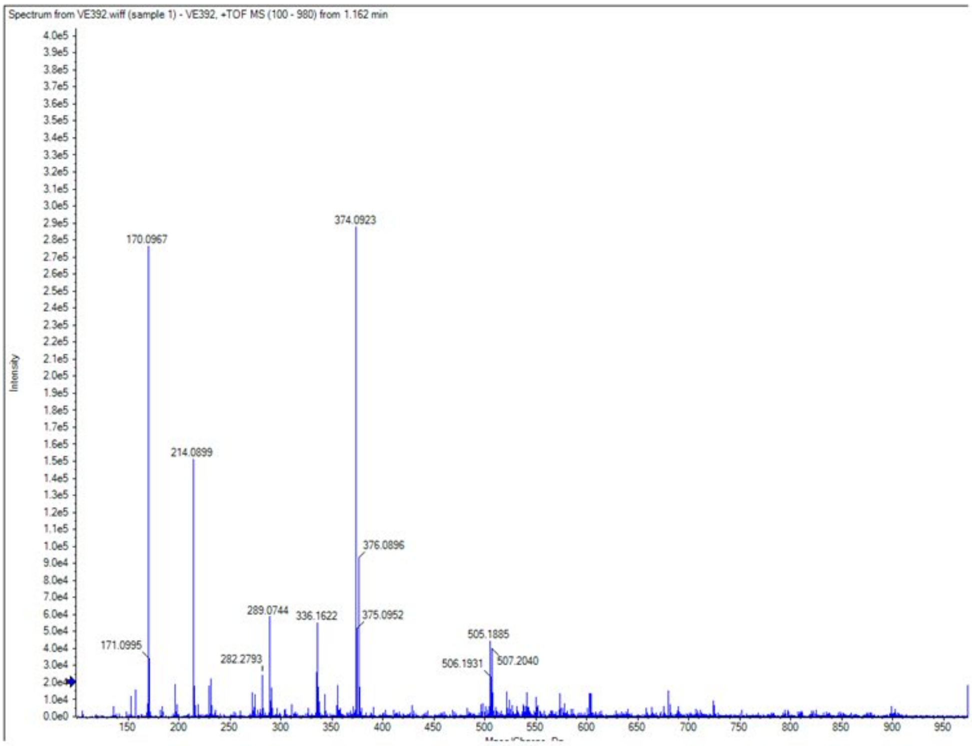

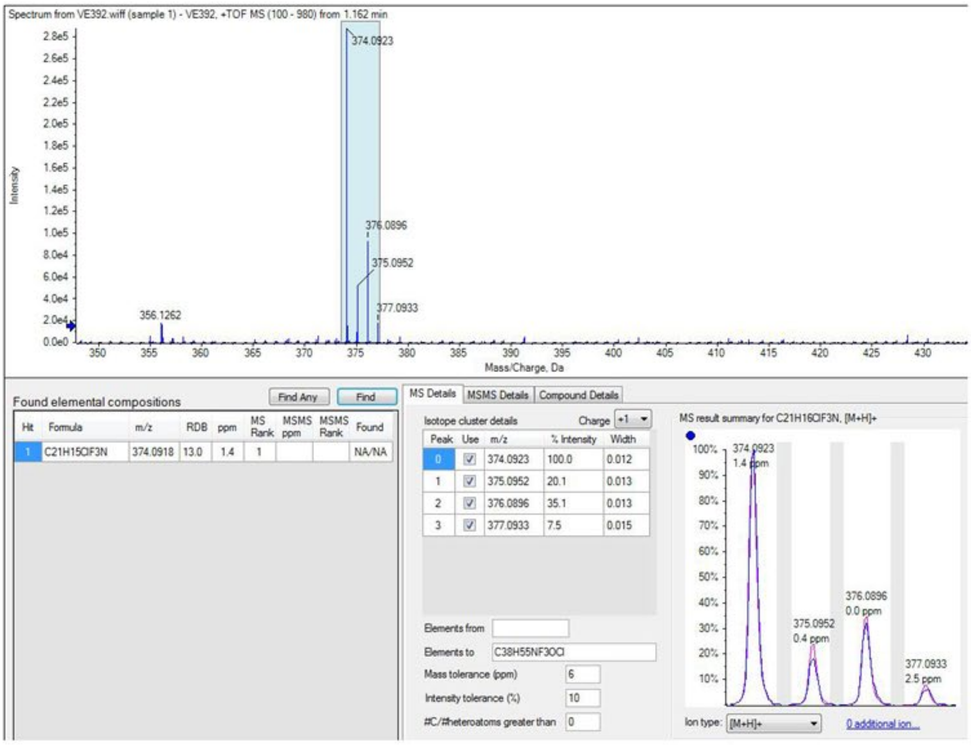

NMR (500 MHz) study of **3da** formation in toluene-d_8 at 80 °C:

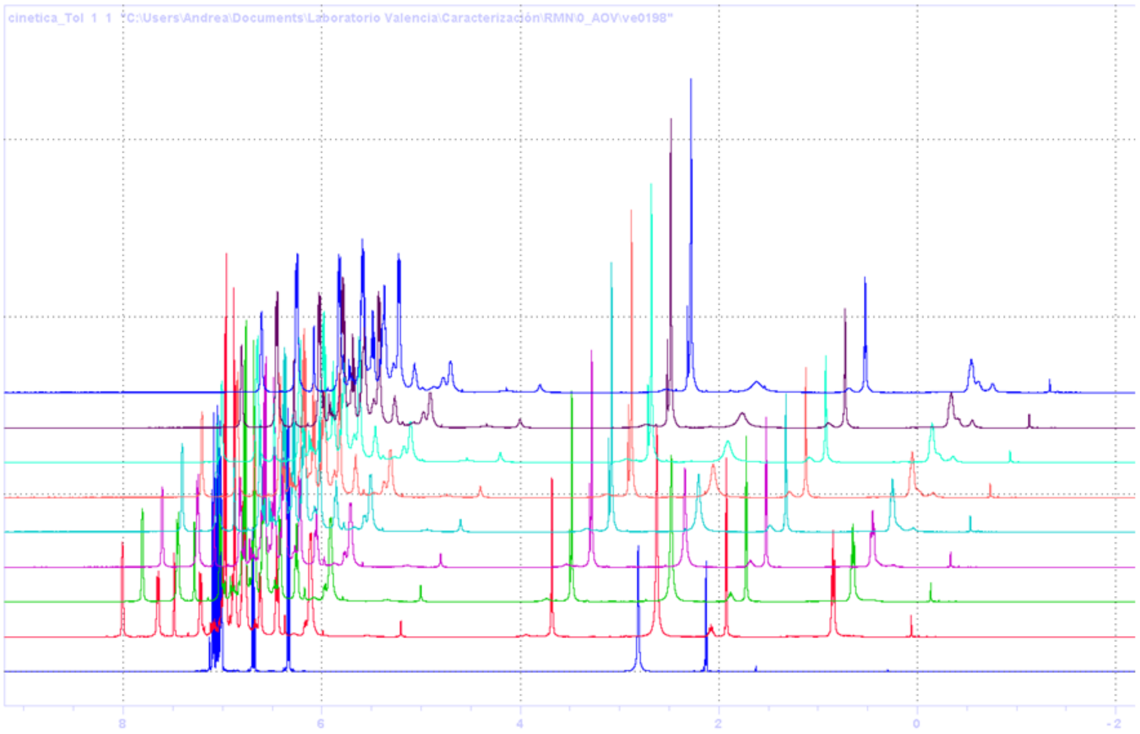

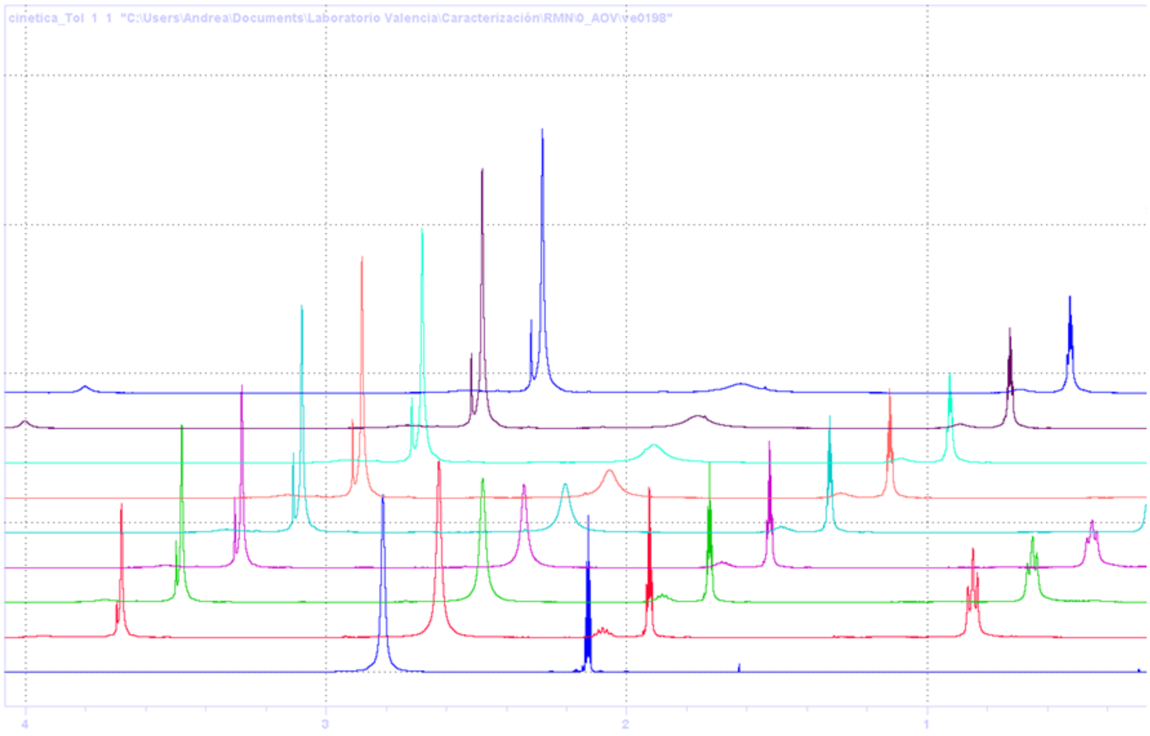

^1H (500 MHz) and $^{13}C\{^1H\}$ (125 MHz) NMR of **3da** in toluene-d_8 :

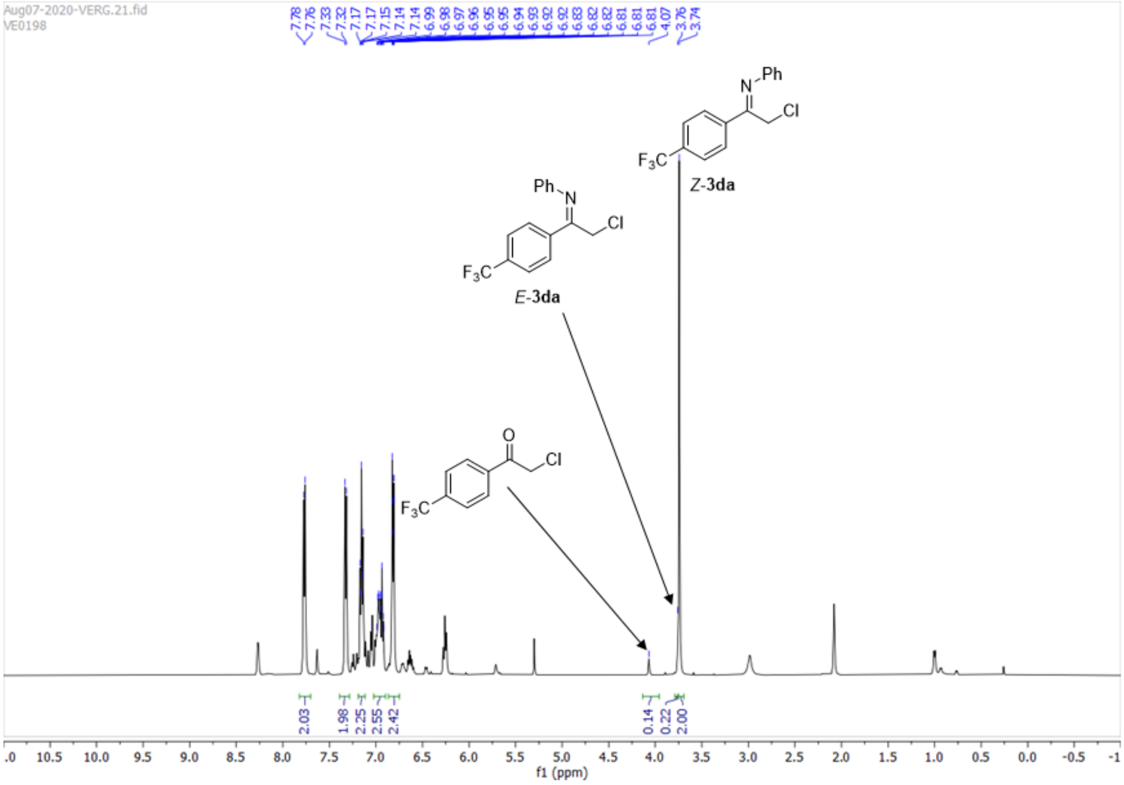

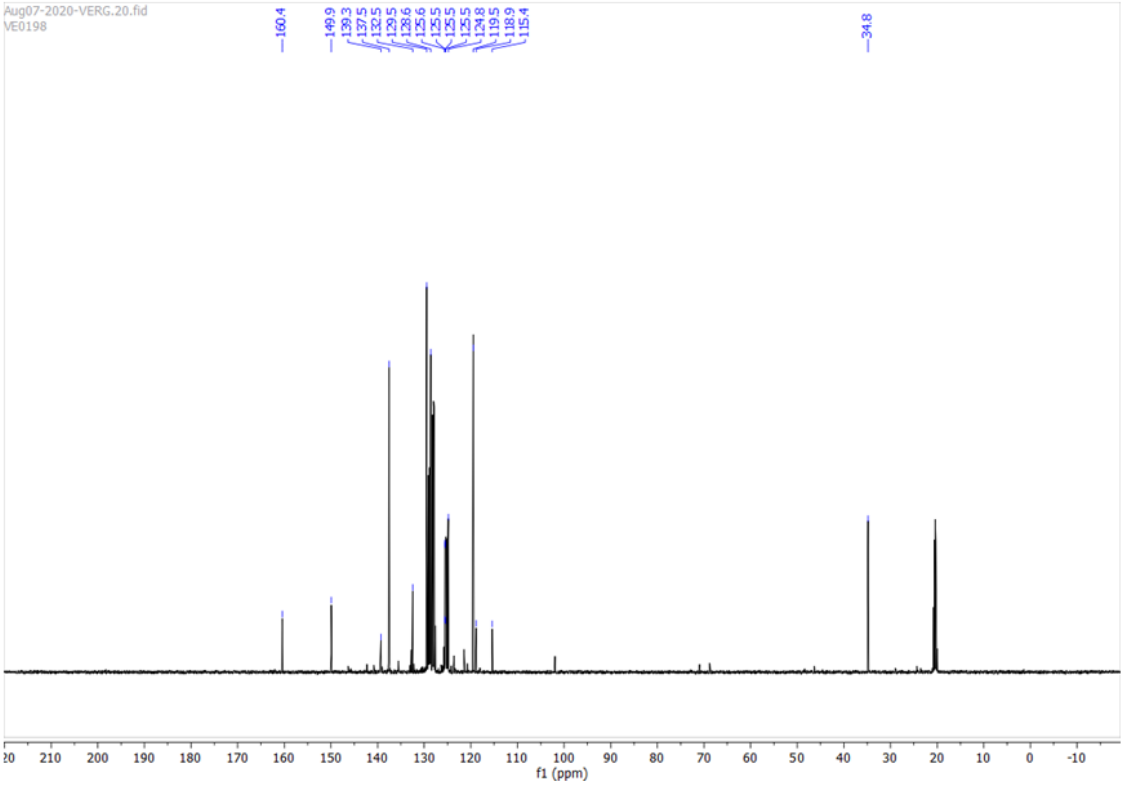

^{1}H (300 MHz) and $^{13}C\{^{1}H\}$ (75 MHz) NMR of **7aa** in $CDCl_3$

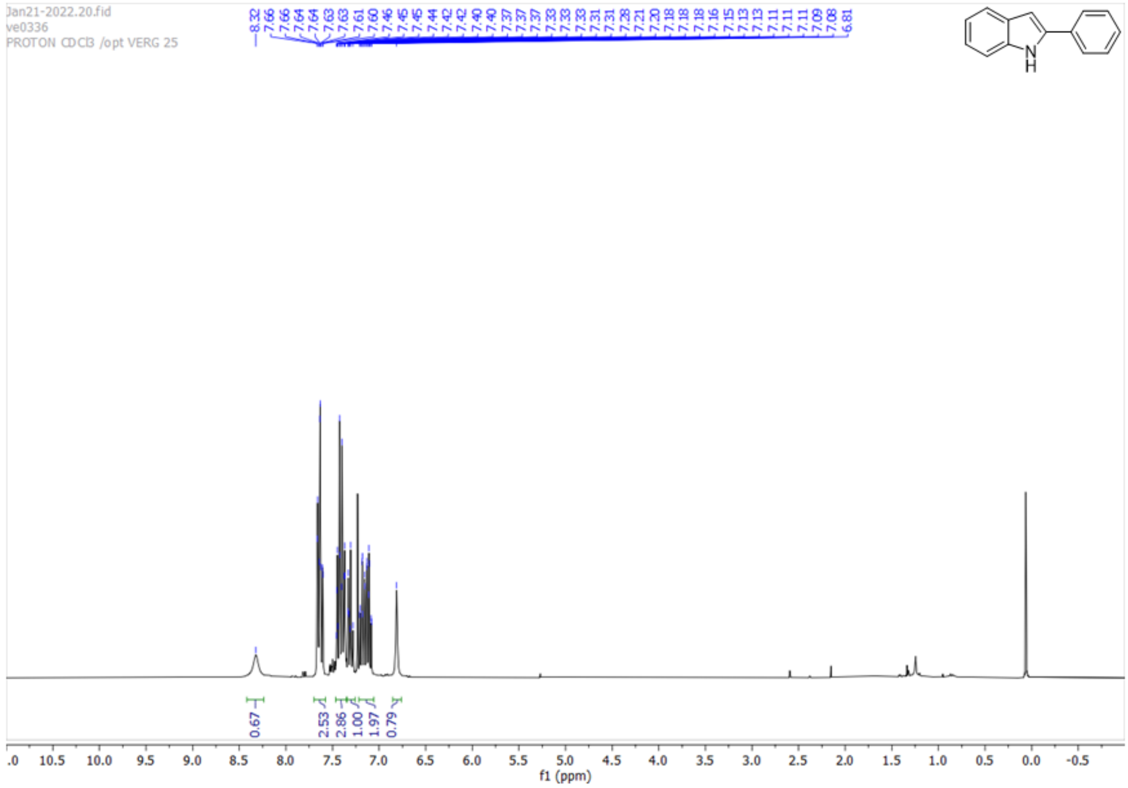

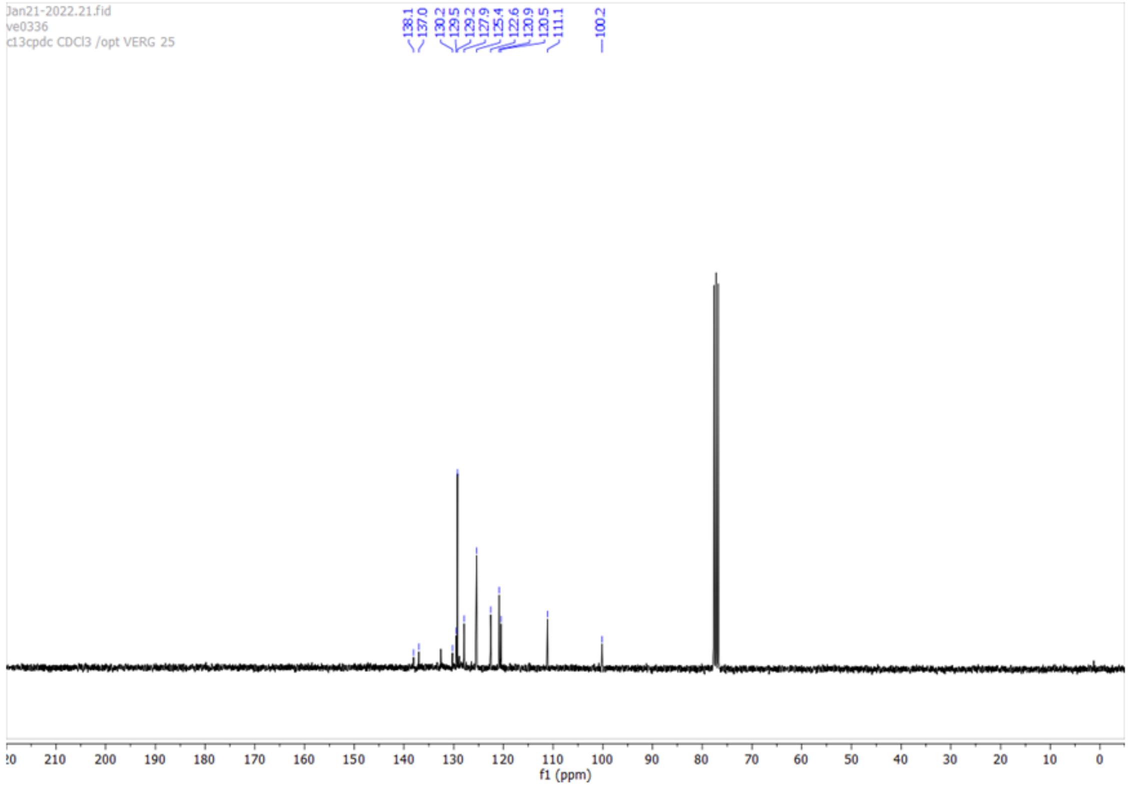

^1H (500 MHz) and $^{13}C\{^1H\}$ (125 MHz) NMR of **7ba** in $CDCl_3$

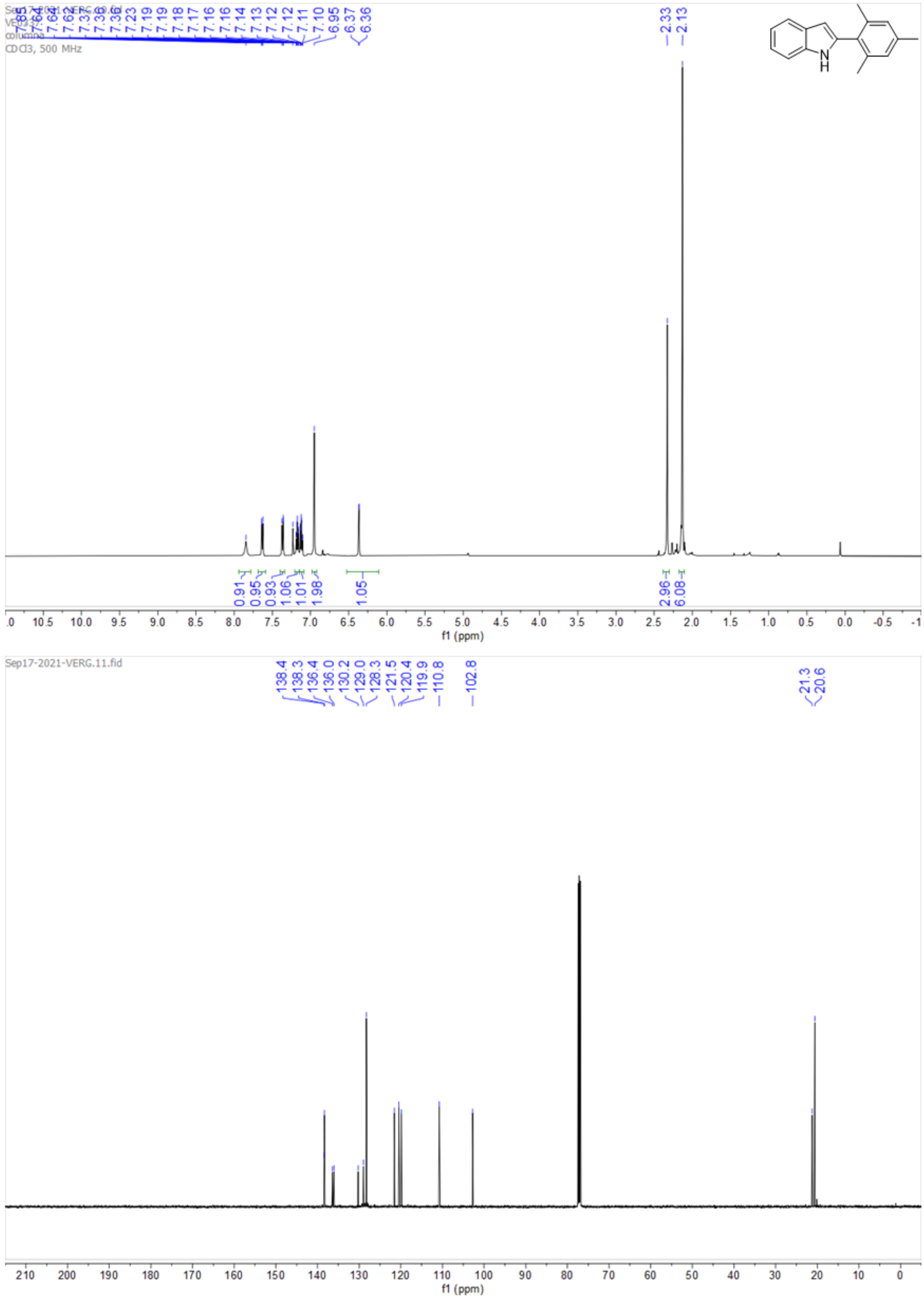

[1]H (500 MHz) and ^{13}C{^{1}H} (125 MHz) NMR of **7ea** in $CDCl_3$

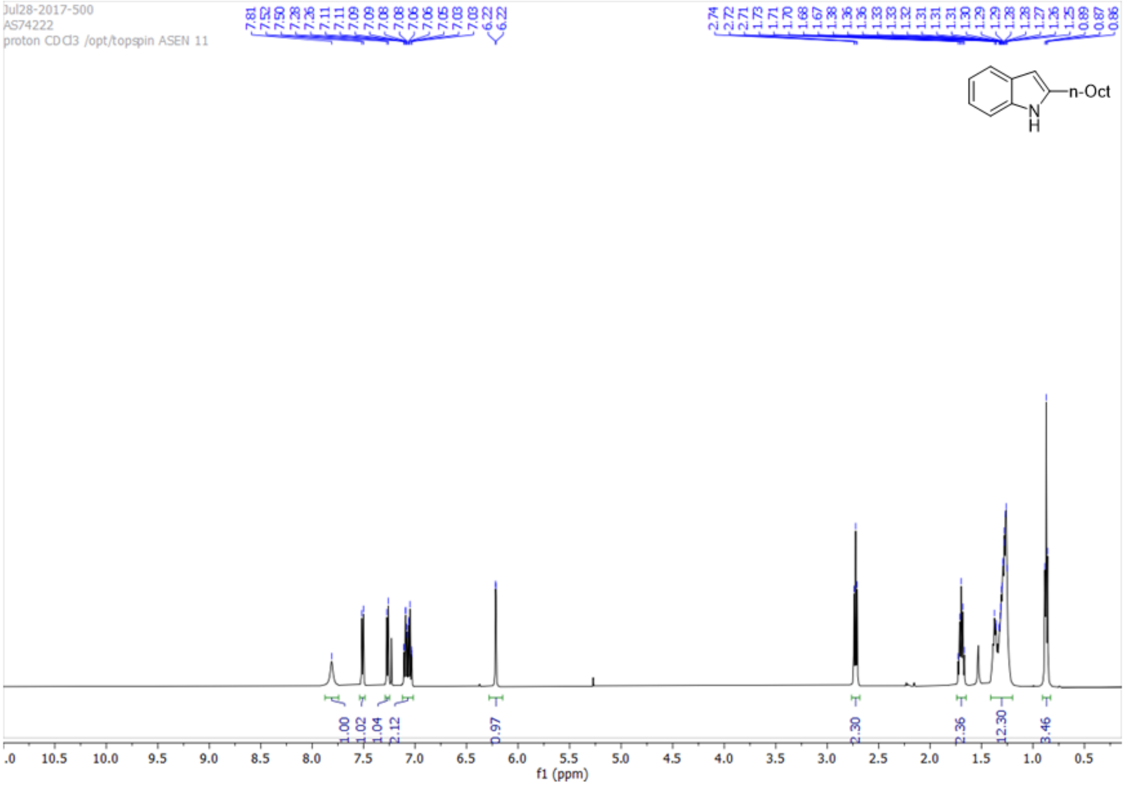

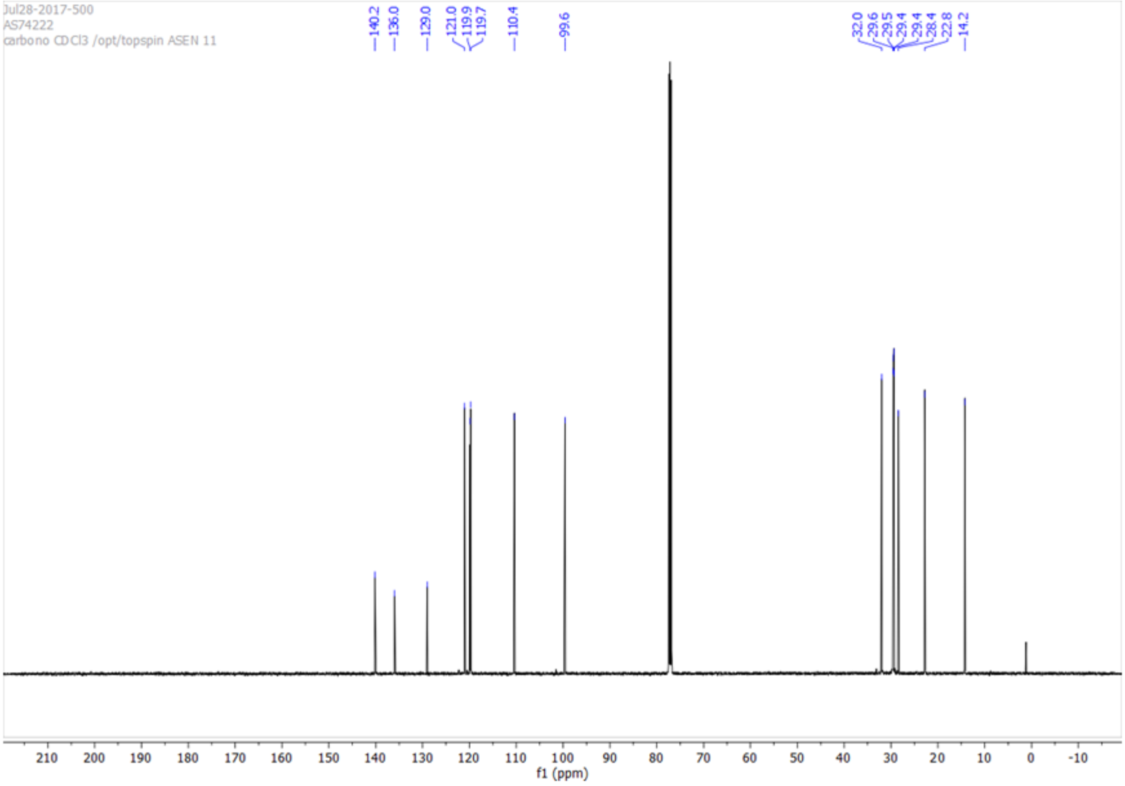

^1H (500 MHz) and $^{13}C\{^1H\}$ (125 MHz) NMR of **8aa** in $CDCl_3$

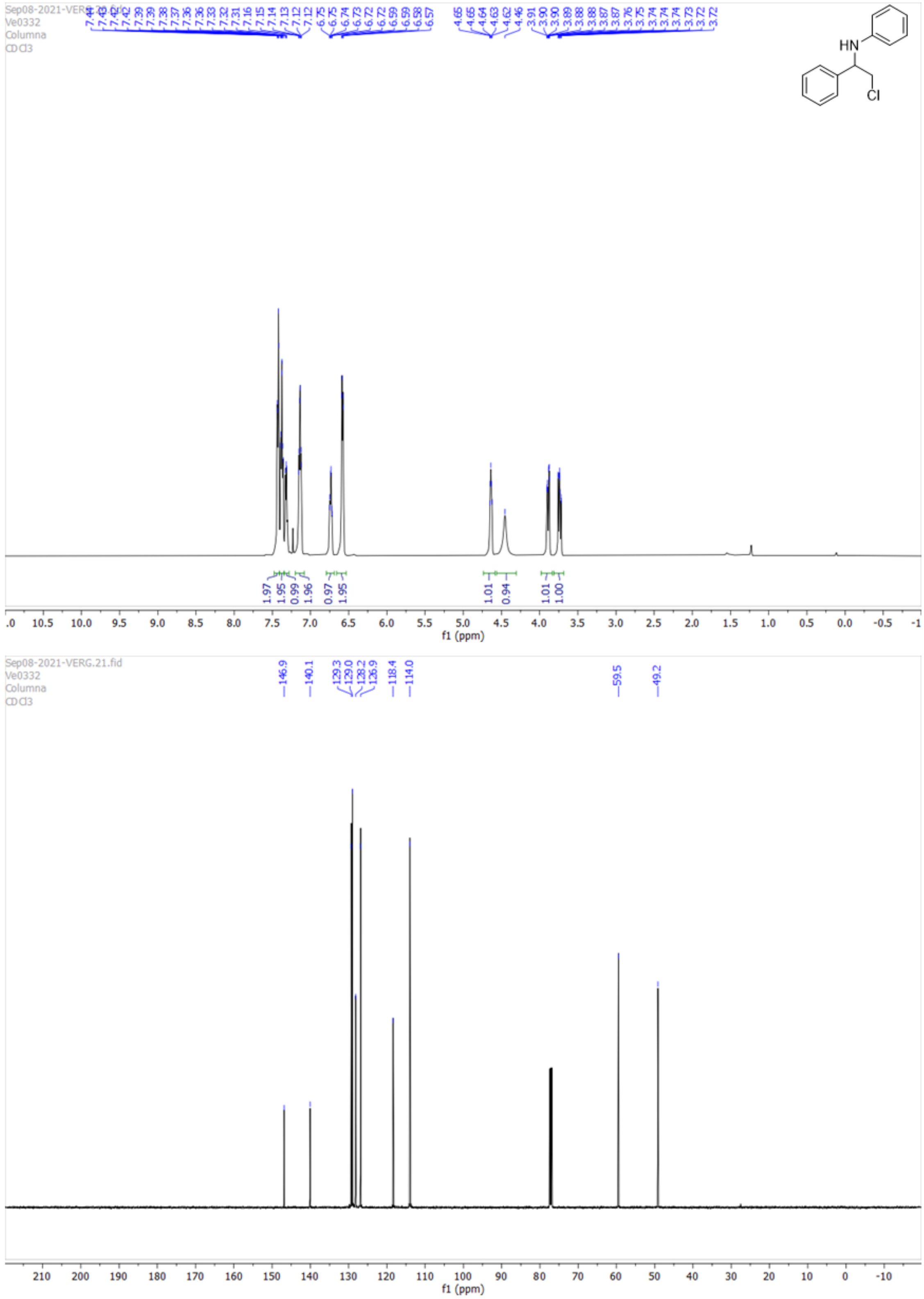

^{1}H (300 MHz) and $^{13}C\{^{1}H\}$ (75 MHz) NMRof **8ab** in $CDCl_3$

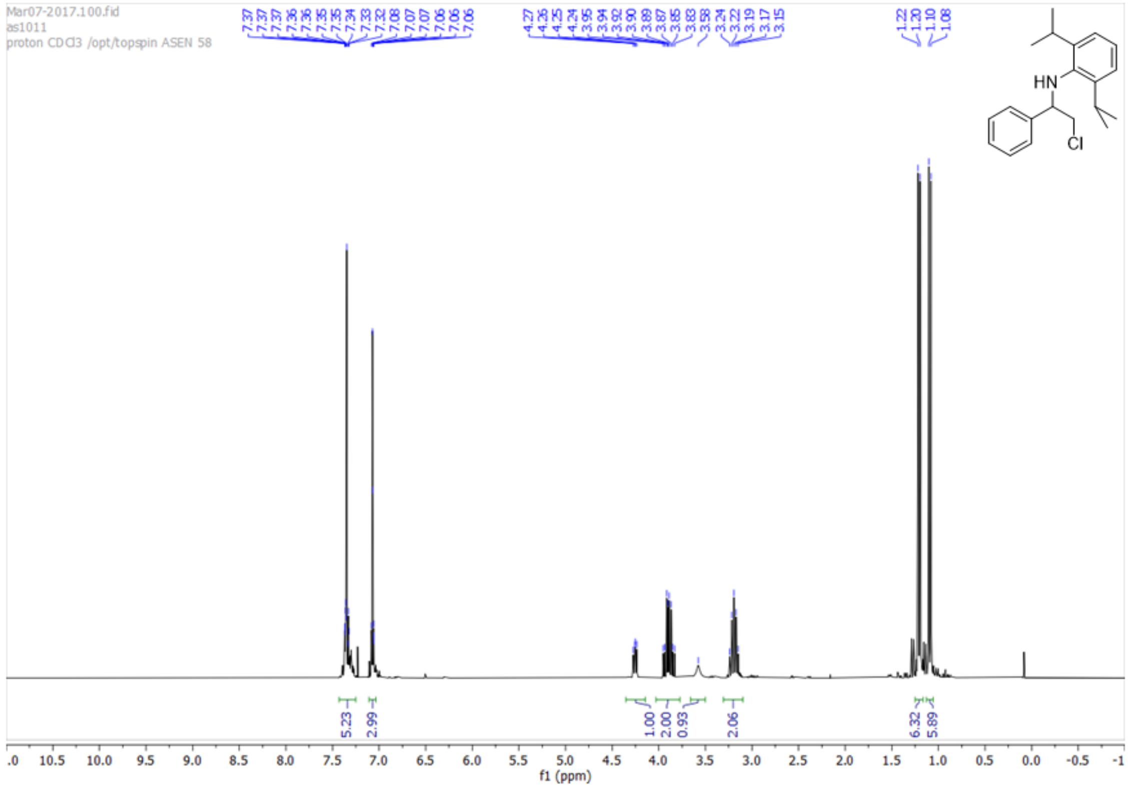

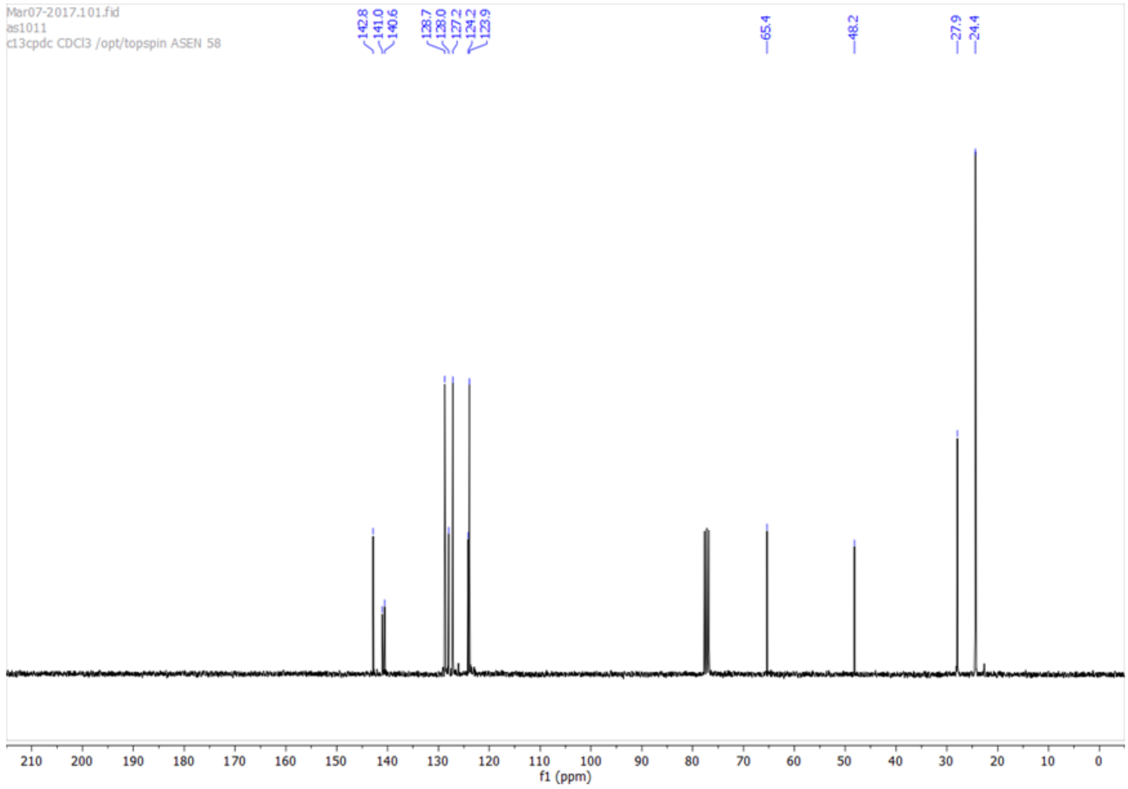

HRMS (+ESI) of **8ab**:

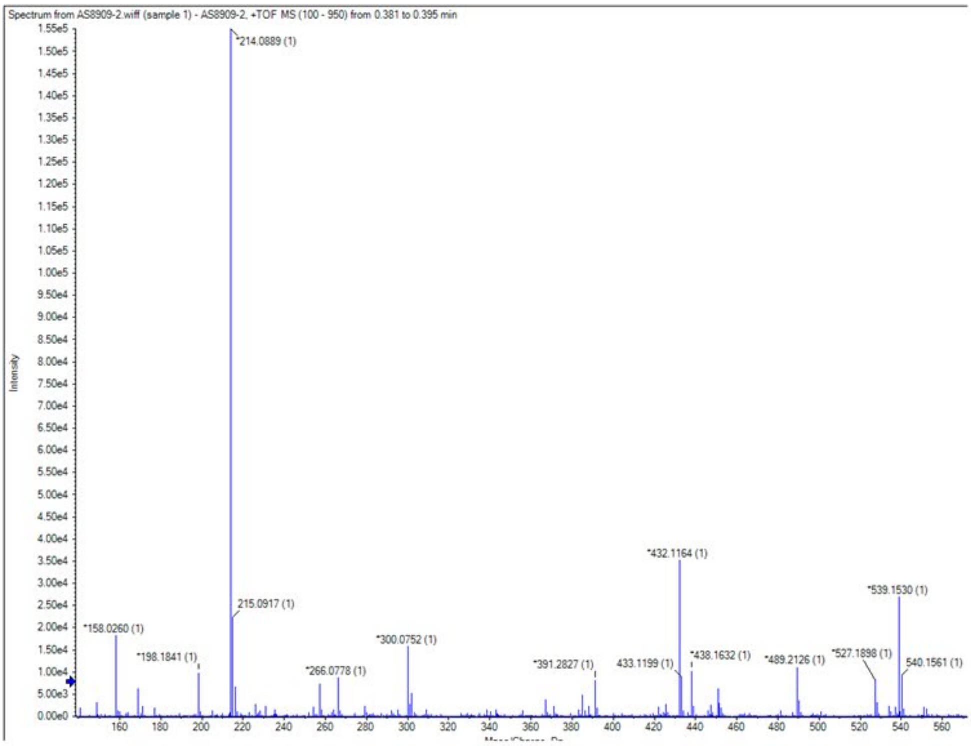

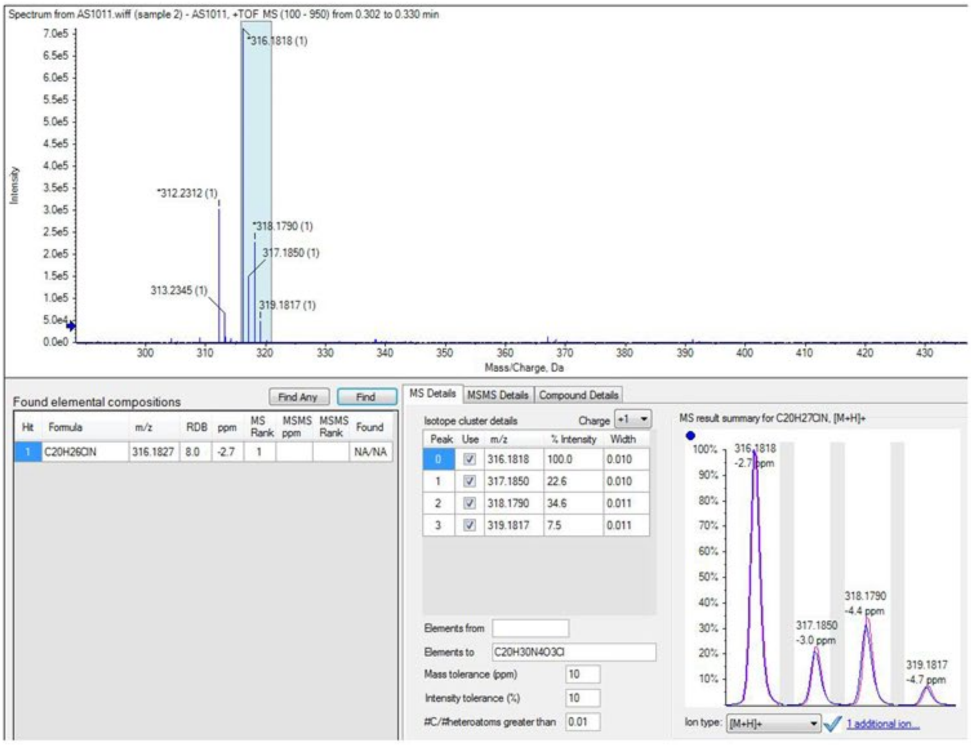

^1H (500 MHz) and $^{13}C\{^1H\}$ (125 MHz) NMRof **8ad** in $CDCl_3$

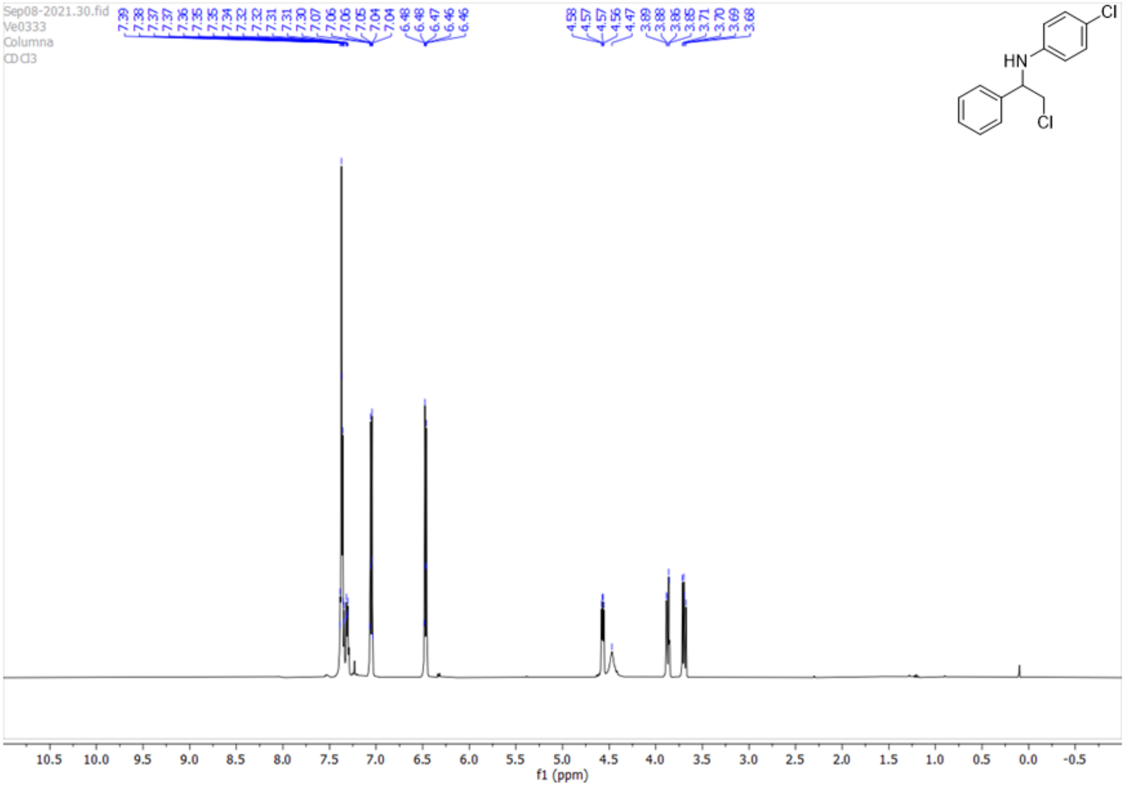

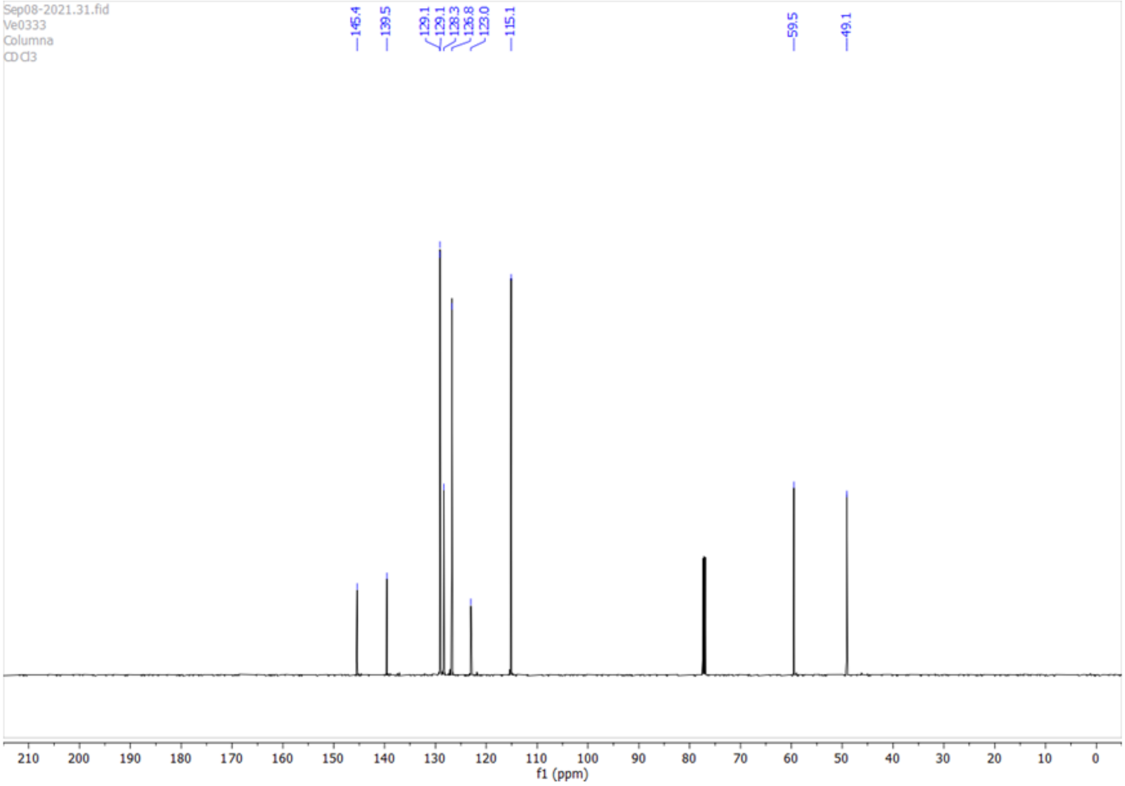

^{1}H (300 MHz) and $^{13}C\{^{1}H\}$ (75 MHz) NMR of **8ca** in $CDCl_3$

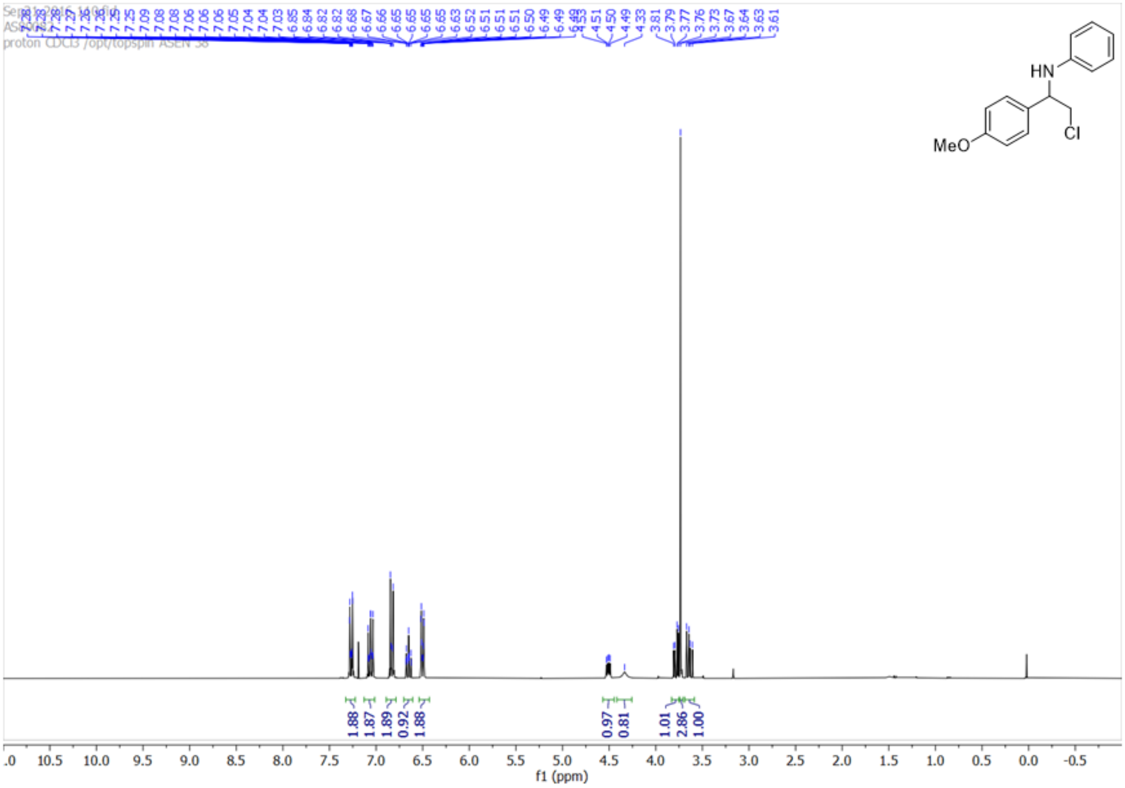

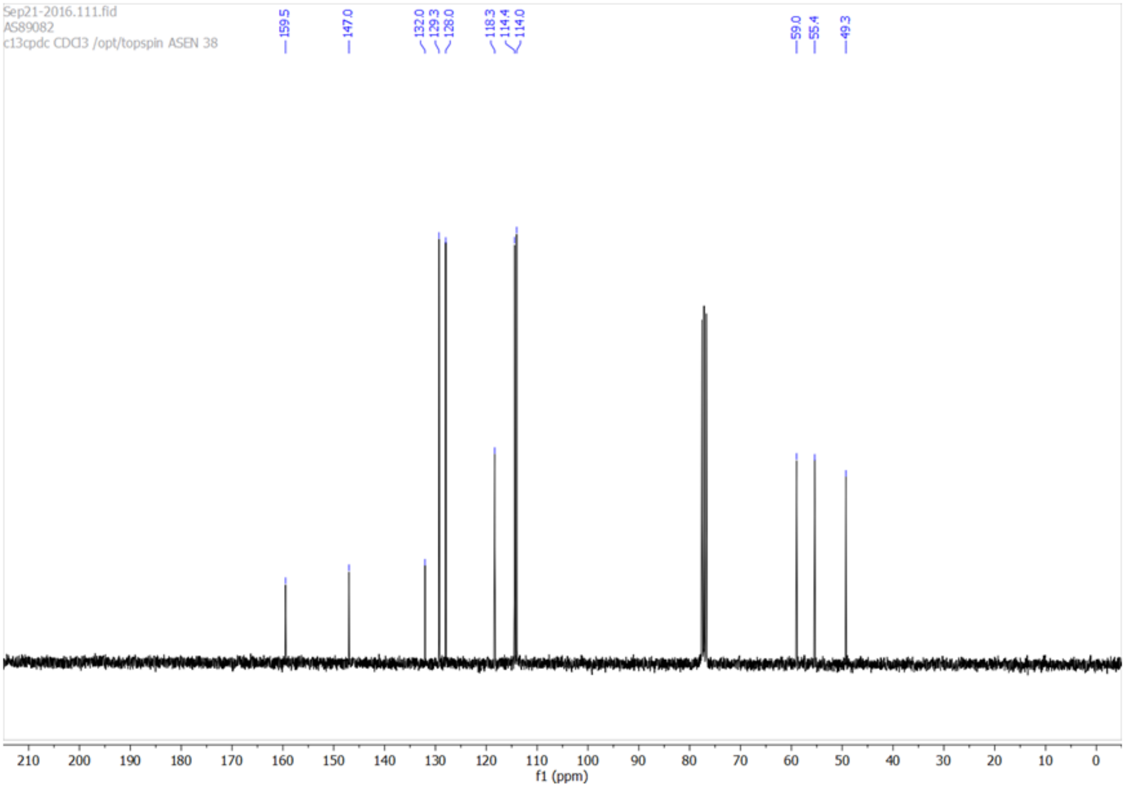

HRMS (+ESI) of **8ca**:

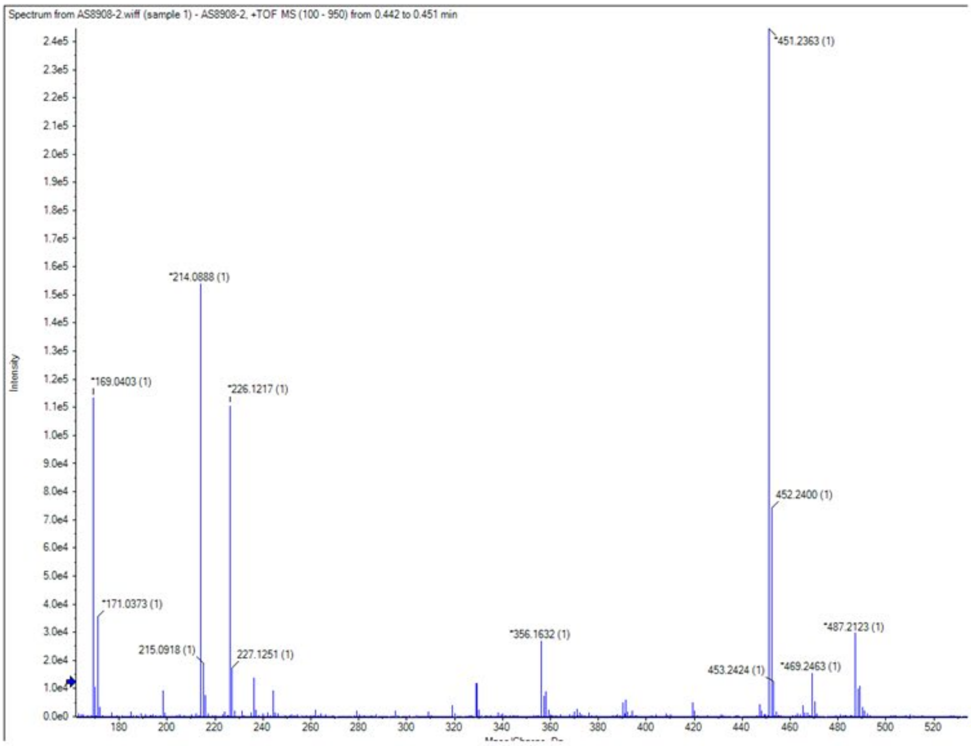

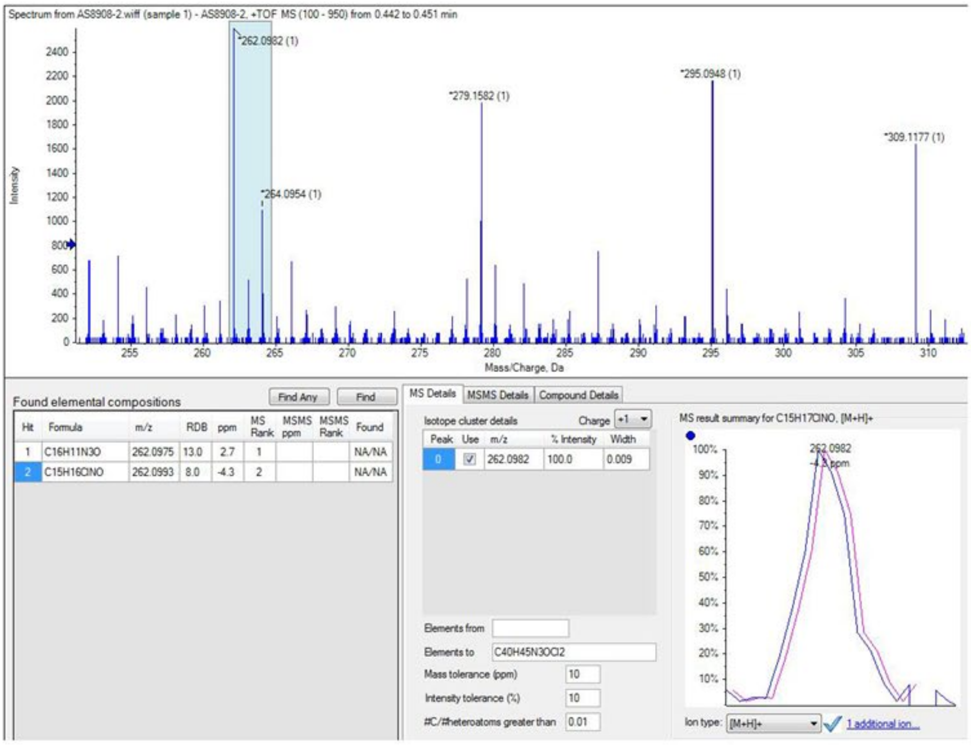

[1]H (300 MHz) and ^{19}F (282 MHz) NMR of **8da** in $CDCl_3$

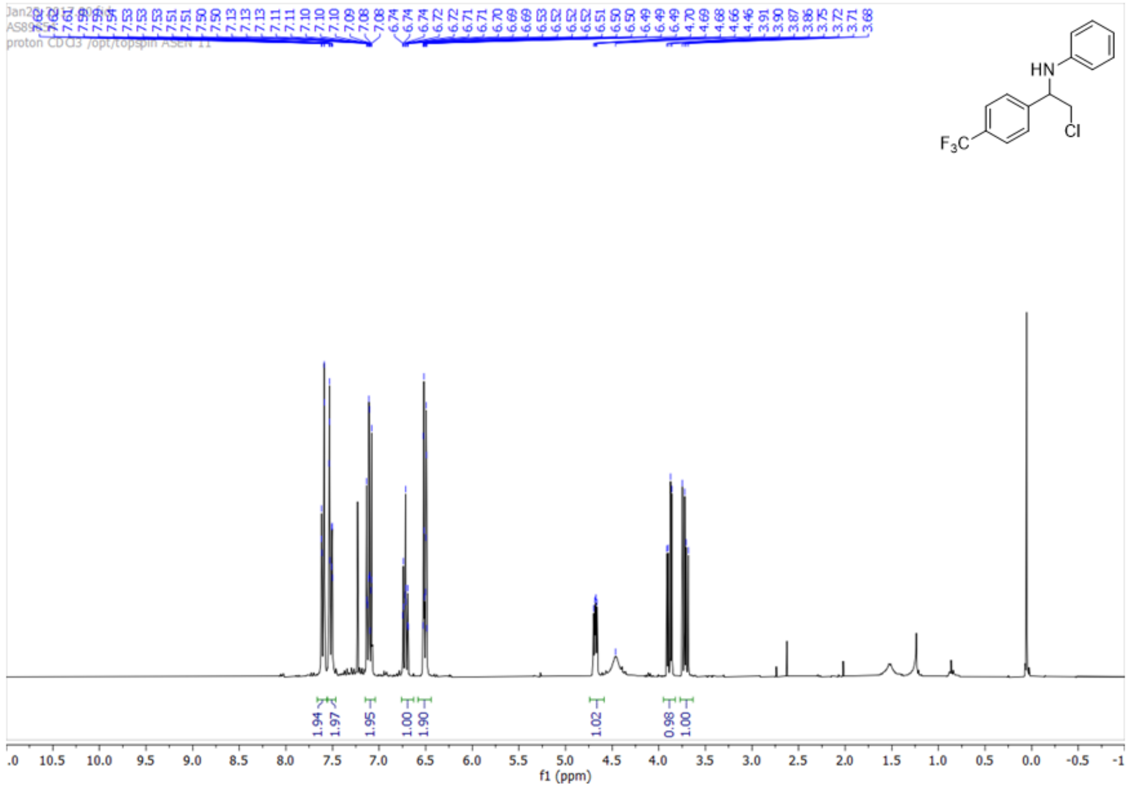

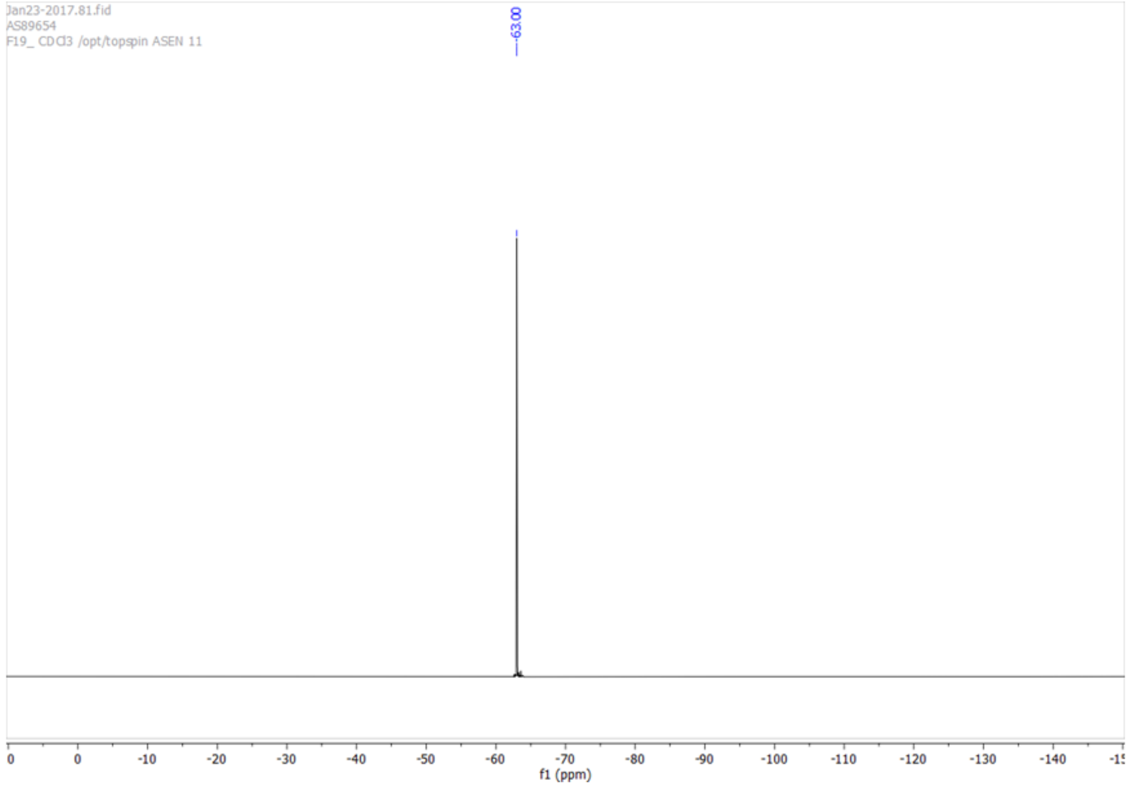

$^{13}C\{^{1}H\}$ (125 MHz) NMR of **8da** in $CDCl_3$

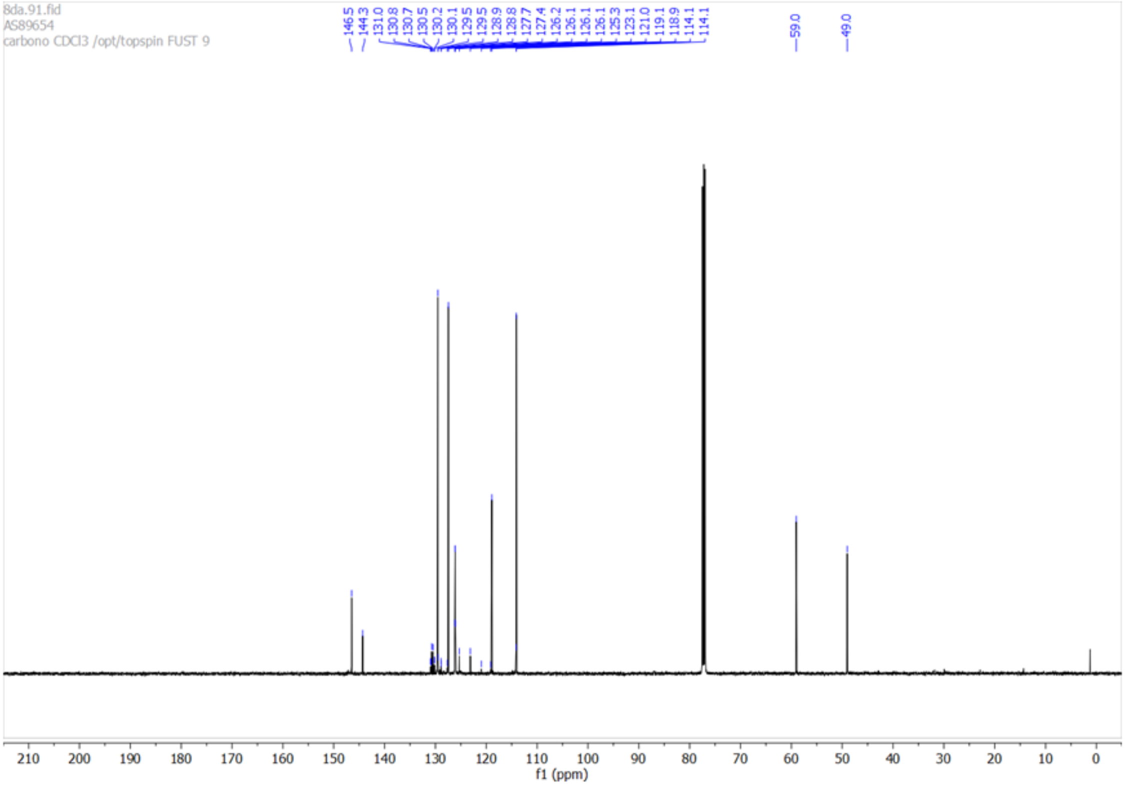

HRMS (+ESI) of **8da**:

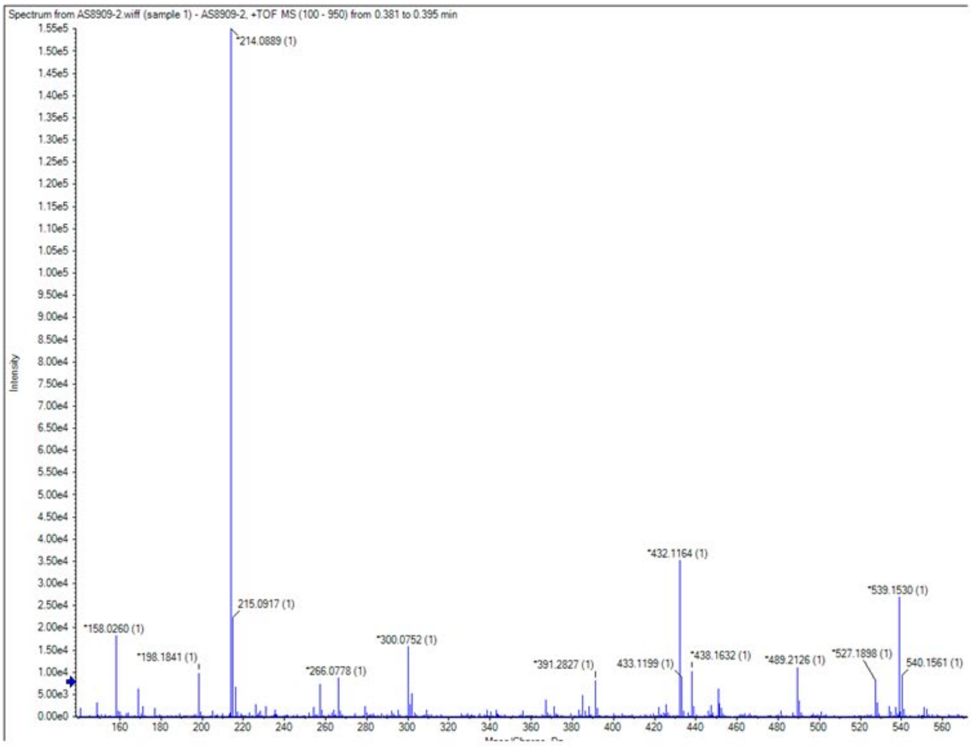

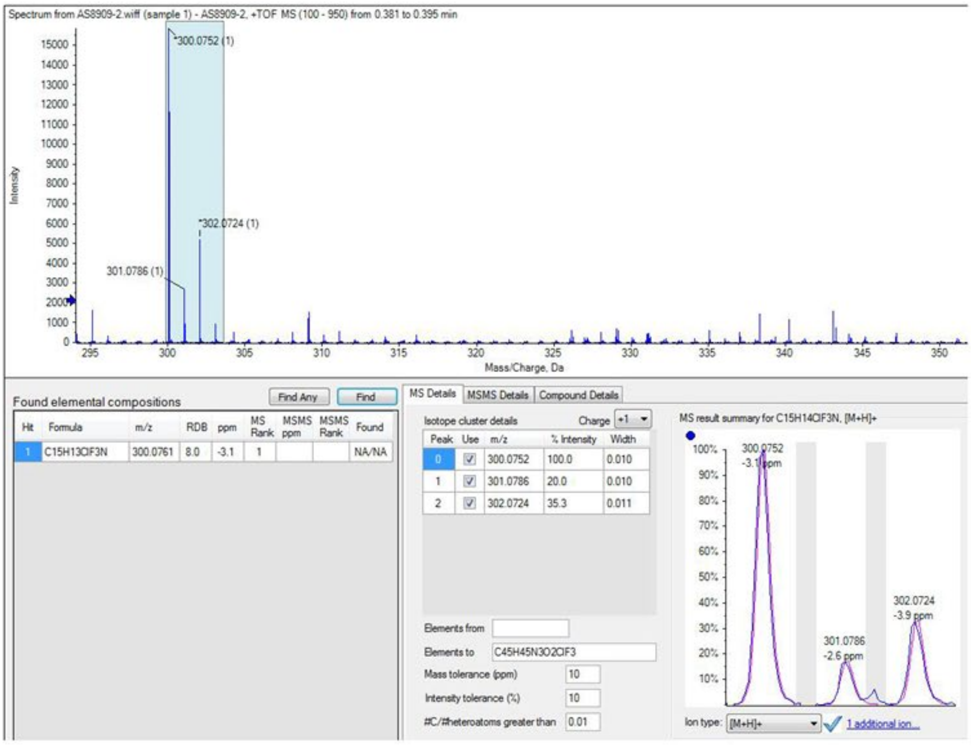

10. References

1. Adak, T.; Schulmeister, J.; Dietl, M. C.; Rudolph, M.; Rominger, F.; Hashmi, A. S. K. Gold-Catalyzed Highly Chemo- and Regioselective C-H Bond Functionalization of Phenols with Haloalkynes. *Eur. J. Org. Chem*. **2019**, 3867-3876. DOI: 10.1002/ejoc.201900653.
2. Shi, W.; Guan, Z.; Cai, P.; Chen. H. Highly Efficient and Recyclable Catalyst for the Direct Chlorination, Bromination and Iodination of Terminal Alkynes *J. Catal.* **2017**, *353*, 199-204. DOI: 10.1016/j.jcat.2017.07.019.
3. Bellezza, D.; Noverges, B.; Fasano, F.; Sarmiento, J. T.; Medio-Simón, M.; Asensio G. Palladium-Catalyzed C–C Ring Closure in α-Chloromethylimines: Synthesis of 1H-Indoles *Eur. J. Org. Chem*. **2019**, 1229-1235. DOI: 10.1002/ejoc.201801607.
4. Yue, G.; Wu, Y.; Wu, C.; Yin, Z.; Chen, H.; Wang, X.; Zhang, Z. Synthesis of 2-arylindoles by Suzuki coupling reaction of 3-bromoindoles with hindered benzoboronic acids. *Tetrahedron Lett*. **2017**, *58*, 666-669. DOI: 10.1016/j.tetlet.2017.01.014.
5. Malkov, A. V.; Stončius, S.; Kočovský, P. Enantioselective Synthesis of 1,2-Diarylaziridines by the Organocatalytic Reductive Amination of α-Chloroketones. *Angew. Chem. Int. Ed.* **2007**, *46*, 3722-3724. DOI: 10.1002/anie.200700165.
